# Knowledge, attitude, and practices towards rabies: A community survey in selected areas of KwaZulu-Natal Province, South Africa

**DOI:** 10.1371/journal.pone.0352279

**Published:** 2026-07-09

**Authors:** Mohube Titus Letsoalo, Daniel Nenene Qekwana, Christian A. Mbajiorgu, Ishmael Festus Jaja, James Wabwire Oguttu

**Affiliations:** 1 Department of Agriculture and Animal Health, College of Agriculture and Environmental Sciences, University of South Africa, Johannesburg, South Africa; 2 KwaZulu-Natal Department of Agriculture and Rural Development, Durban, KwaZulu-Natal, South Africa; 3 Department of Paraclinical Science, Faculty of Veterinary Science, University of Pretoria, Pretoria, South Africa; GCS Medical College, INDIA

## Abstract

Knowledge, attitude, and practices (KAP) studies have been widely used to assess gaps in KAP towards rabies. However, there is no evidence of baseline studies that have assessed KAP towards rabies among selected communities in KwaZulu-Natal Province, South Africa. A cross-sectional, questionnaire-based study design was adopted. Participants (n = 768) were selected using systematic random sampling. Data from respondents (≥18 years of age) were collected using a validated questionnaire and analysed using descriptive and inferential statistics. Overall, 26.95% (207/768) of the respondents could name the cause of rabies, while 53.52% (411/768) indicated that they would adhere to post-exposure prophylaxis (PEP), which can include, depending on the category of the bite wound, wound washing, and receiving a dose of human rabies immune globulin (HRIG) and rabies vaccine at the time of the first medical visit. Most respondents (90.10%, 692/768) would seek medical attention following a dog bite. Most dog owners (82.11%, 459/559) owned a vaccination certificate for their pet. Allowing dogs to roam without supervision was widely reported by the respondents. Dog ownership was the only factor significantly associated (p = 0.0001) with knowledge of rabies. Gaps in some aspects of knowledge about rabies were observed, which require awareness creation among residents of the study area, irrespective of whether they own pets.

## 1. Introduction

Domestic dogs play a significant role in the lives of humans. However, they pose the threat of transmitting rabies to humans [1]. Rabies is an important infectious viral disease [2,3] caused by lyssavirus, from the family *Rhabdoviridae* [4,5]. All mammals are susceptible to rabies virus infection, which is generally transmitted through the bite of a rabid animal [6].

Dog-mediated rabies infection remains a significant disease from a public health perspective [7] and annually it is responsible for an estimated 60 000 human deaths worldwide [8,9]. Of these, Asia has the highest burden of rabies, followed by Africa, making the two the most affected continents [9,10]. In Africa, over 90% of human rabies deaths are associated with dog bites [11,12]. n Asia, 31 539 human deaths attributed to rabies are reported annually, suggesting a high rate of rabies infection compared to other continents [13]. Furthermore, globally, around 15 million people seek rabies post-exposure prophylaxis (PEP) to minimise the risk of rabies infection following exposure to dog bites [14].

Once symptoms of rabies develop, victims usually succumb to the disease [4]. Moreover, the risk of developing symptoms is high if the victim fails to obtain PEP early following a dog bite [15]. In humans potentially exposed to the rabies virus, PEP usually includes thorough wound cleaning and a combination of treatment with rabies immunoglobulin and vaccination [16]. In the absence of appropriate healthcare interventions, untreated individuals are at increased risk of death. Moreover, the disease is reported to have the highest case fatality of any disease [11]. Details of the treatment prescribed for dog-bite victims to prevent the development of rabies have been described by Bishop et al (2010) [17]. In addition, the *WHO Expert Consultation on Rabies*, third edition, 2018, provides clear guidelines on the prevention of rabies in humans.

Rabies is a preventable disease, and vaccination of dogs through mass vaccination campaigns is one of the effective ways to control the disease at its source [18,19]. According to the World Health Organization (WHO), a minimum of 70% dog vaccination coverage can drastically reduce the number of human cases in countries where rabies is endemic [20]. However, in most developing countries, rabies continues to be a challenge resulting in high mortality in humans and animals [21]. This is despite the existence of effective human and animal rabies vaccines [11]. This could be attributed to inadequate efforts to control and prevent the spread of the disease in these countries [11,21].

In South Africa, an estimated 40 human rabies-related deaths resulting from dog bites occur yearly [16]. In view of this, there is a need for adequate measures to successfully prevent and control the spread of rabies within the dog population. This is especially true in KwaZulu-Natal (KZN) Province, which has the highest number of dog rabies cases in the country [22]. In addition, rabies is well established and remains endemic in the province [23]. This is supported by the recent increase in the number of laboratory confirmed positive animal cases in KZN, most of which have been confirmed in dogs [16,24]. Besides KZN Province, the Eastern Cape and Limpopo provinces in South Africa are considered to have high numbers of rabies cases [16].

Knowledge, attitude, and practices (KAP) studies constitute an important methodological approach for acquiring insights into the subject of concern [25]. Therefore, questions on the cause, source and mode of transmission, clinical signs, prevention practices and treatment measures are important in determining participants’ KAP regarding rabies [26]. In addition, for prevention and control measures for rabies to be effective, community knowledge on how to handle a suspected rabid dog [27], as well as the measures that must be adopted when bitten by a rabid dog, needs to be assessed [28].

Good KAP about rabies has been shown to reduce the spread of the disease and the fatalities associated with it [18]. Furthermore, KAP studies can also be used to identify gaps in KAP towards rabies, allowing for the design of targeted campaigns to fill those gaps. In view of this, the present study investigated the KAP in Embo and Verulam, located in the eThekwini District of the KwaZulu-Natal Province in South Africa.

## 2. Methods

### 2.1. Study area

The study was conducted within the eThekwini District in KwaZulu-Natal Province, South Africa. KwaZulu-Natal is one of the nine provinces in South Africa and is a multiracial community. The population of KZN is approximately 10 819 130, living in an area of 94 361 km². Its annual population growth is estimated to be 1.2% [29].

The eThekwini District is occupied by about 3.7 million people on a land area of approximately 2 555 km² [30]. Embo (Latitude: 29° 44’ 46” S, Longitude: 30° 46’ 03” E) is a rural area situated in the inland part of eThekwini in the western region of the district, with an estimated population of approximately 40 924 people and an estimated 8 184 households. Verulam (Latitude: 29° 38’ 40” S; Longitude: 31° 02’ 40” E), on the other hand, is an urban area located in the upper part of the northern region [30]. Verulam has an estimated 64 950 households (unpublished data, Verulam Environmental Health Manager) and approximately 184 114 occupants on a land area of 18.13 km^2^ [30].

### 2.2. Study design

A cross-sectional study design was adopted to investigate the KAP of community members towards rabies in the eThekwini District in KZN Province of South Africa.

### 2.3. Target population

The target population for this study was residents of Embo, a rural area, and Verulam, an urban area, aged 18 years and older. The study included people of different age groups, religious beliefs, and educational backgrounds. People of both sexes, male and female, and both pet owners and non-pet owners were also included in the study.

### 2.4. Sample size determination

The sample size for the two study areas, Embo and Verulam, was determined using the formula described by Guadu et al. [26]:


$$\begin{array}{c} N=\frac{\text{1}.\text{9}{\text{6}}^{\text{2}}\times Pexp(\text{1}-Pexp)}{(d{)}^{\text{2}}} \end{array}$$

*N* = sample size

*Pexp* = estimate or expected proportion of knowledge about rabies from the community was considered at 50%, given that there is no baseline study regarding KAP in the study area.

*d*^2^ = desired absolute precision (5%)

A total of 768 respondents, 384 from each of the two areas, were recruited and agreed to participate voluntarily in the study.

### 2.5. Sampling strategy

A systematic random sampling method was used to sample the two selected areas, Embo and Verulam. In both areas, a sampling interval was predetermined by dividing the estimated number of households in the area by the sample size before the selection of households commenced. The first household was selected randomly by allocating numbers to the first houses constituting the sampling interval, namely 21 houses in Embo and 169 houses in Verulam. A number was then randomly drawn from the list, and the house corresponding to the number selected was used as the starting point. Thereafter, successive households were selected following the predetermined sampling interval for each of the two areas, 169 and 21 in Verulam and Embo, respectively, until the computed sample size was reached. Streets were used as intersections, and houses on one side of the street were sampled. When the interviewers reached the end of the street, they crossed onto the opposite side and continued in the opposite direction. Thereafter, they moved to the next street, and the process was repeated until the required sample was reached. In cases where the household head was absent, an adult aged ≥18 years who resided in the home was interviewed [19]. If tthe household head or an adult who could complete the questionnaire was absent at the time of the interview, the next house was chosen.

### 2.6. Data collection

The questionnaire developed by Fenelon et al. [31] was adapted for this study (Supplementary material I). Data was collected using a structured questionnaire during face-to-face interviews. The questionnaire included the following sections: respondents’ demographic data and KAP regarding rabies.

The questionnaire was translated into the most widely-spoken local language (isiZulu) to cater for respondents who could not communicate in English. In addition, the questionnaire was pre-tested on fifteen participants from each area to test its fitness for purpose. Each interview session lasted 30–45 minutes and was conducted in English or IsiZulu. The questionnaire was administered from 01/08/2019 to 31/03/2021.

### 2.7. Data management and data analysis

#### 2.7.1. Data management.

Each questionnaire was reviewed carefully after each data collection session to ensure that it had been filled in correctly, and each response was then coded. All raw data were captured using Microsoft Excel 365 (Microsoft Corp., Redmond, WA, USA). The correct response to each of the knowledge questions was awarded a score of 1, while 0 was given for an incorrect answer. The knowledge score of each respondent was computed and presented as a percentage. Thereafter, a binary variable was created from the knowledge score using 60% as the cut-off for being knowledgeable (≥60%) or not knowledgeable (<60%), using the criteria described by Alam et al. (2021) [32].

#### 2.7.2. Data analysis.

Data were analysed using the Statistical Package for the Social Sciences (SPSS) version 27 (SPSS Inc., Chicago, IL, USA). Descriptive statistics, such as frequencies and percentages of all categorical variables, were computed and presented as graphs and tables. Inferential analysis involved first fitting a series of univariate binary logistic regression models to test for simple associations between the predictor variables and the outcome variable, knowledge score. Variables with a p-value ≤0.20 in the univariable model were included in the multivariable model. Significance in the multivariable model was assessed at p < 0.05.

A multivariable logistic regression model was then fitted to the data to assess the factors that were significantly associated with being knowledgeable about rabies. Using the backward selection method, variables to retain in the final regression model were identified. If the removal of a variable from the model resulted in a ≥ 20% change in the effect measure of any of the variables in the model, the variable was considered a confounder and was therefore retained in the final effects model, regardless of whether it was significantly associated with the outcome variable.

Models were compared using the log-likelihood ratio test. The model fit of the final effects model was tested using the Hosmer–Lemeshow test. Based on the results, there was good model fit (p > 0.05).

## 3. Ethical considerations

The State Veterinary Services of the KwaZulu-Natal Department of Agriculture and Rural Development granted approval to conduct the study. In addition, permission to carry out the study was granted by the Ethics Committee of the College of Agriculture and Environmental Sciences, University of South Africa (REC reference #: 2019/CAES_HREC/105) (Supplementary material II). The identities of the respondents were protected by ensuring that they remained anonymous. The objectives of the study were first explained to the respondents, and thereafter, each participant signed the consent form to indicate that they had voluntarily agreed to participate in the study. All respondents had the right to withdraw from the study at any time during the course of the study.

## 4. Results

### 4.1. Descriptive statistics

#### 4.1.1. Demographic profile of respondents.

An equal number of respondents (n = 384) was drawn from each of the identified areas, Embo and Verulam. Hence, a total of 768 community members were included in the study. The majority of the respondents were female (63.54%, 488/768), aged between 18 and 35 years (53.21%, 409/768), belonged to the Christian faith (47.66%, 366/768), and had not completed secondary education (35.81%, 275/768). Pet ownership was high (72.79%, 559/768) in the study area (Table 1).

**Table 1:** Demographic profile of people who agreed to participate in the study.

Variables	Level	Frequency (n)	Percentage %
Age (years)	18–35	409	53.26
	36–53	288	37.50
	54–71	64	8.33
	>71	7	0.91
Sex	Male	280	36.46
	Female	488	63.54
Residence	Verulam	384	50.00
	Embo	384	50.00
Own a pet dog and/or cat	Yes	559	72.79
	No	209	27.21
Religion	Christian	366	47.66
	African religion	140	18.23
	Hindu	204	26.56
	Muslim	31	4.04
	Other	27	3.52
Education	No education	43	5.60
	Completed primary education	101	13.15
	Secondary education not completed	275	35.81
	Completed matric/high school education	234	30.47
	Completed tertiary education	115	14.97

#### 4.1.2. Knowledge of rabies as a disease.

The overwhelming majority were not aware of the cause of rabies, with 50.52% (388/768) saying that they did not know the cause, 2.47% (19/768) saying it is caused by a chemical substance, and 10.03% (77/768) saying it is caused by insufficient intake of feed and water. Only 26.95% (207/768) of the participants were aware that rabies is a viral disease (Table 2).

**Table 2:** The results of the assessment of the knowledge of rabies among respondents.

Variables	Level	Frequency (n)	Percentage %
What is the cause of rabies?	Chemical substances	19	2.47
	Virus	207	26.95
	Insufficient intake of feed and water	77	10.03
	Psychological problem	77	10.03
	Don’t know	388	50.52
How can humans be infected with rabies?	Dog-bite	668	86.98
	Playing with the dog	15	1.95
	Feeding the dog	0	0
	Don’t know	85	11.07
What is the first thing to be done after a dog bite while at home?	Cover the wound with a bandage	96	12.50
	Cleanse the wound with soap and running water	411	53.52
	Apply any topical medication	134	17.45
	Don’t know	127	16.54
Can rabies be treated after the appearance of clinical signs?	Yes	387	50.39
	No	123	16.02
	Don’t know	258	33.59
When is it appropriate to receive an anti-rabies injection after being bitten by a dog suspected of having rabies?	Weeks later	27	3.52
	Immediately	601	78.26
	Months later	41	5.34
	Don’t know	99	12.89
Is vaccination of pets important for preventing human rabies?	Yes	611	79.56
	No	13	1.69
	Don’t know	144	18.75

Most respondents (86.98%, 668/768) identified dog bites as the most likely route for the transmission of rabies. With respect to the first thing to do after a dog bite while at home, slightly more than half of the respondents (53.52%, 411/768) indicated that they would clean the wound with soap and running water (Table 2).

Although half of the respondents (50.39%, 387/768) were not aware that rabies can be fatal after the appearance of clinical signs, most respondents (78.26%, 601/768) would immediately seek anti-rabies injections following a bite by a suspected rabid animal. Some respondents (12.89%, 99/768) did not know that they needed to get anti-rabies injections immediately. Some thought that it was appropriate to get the anti-rabies injection later (5.34%, 41/768), such as weeks or even months later (Table 2).

Most respondents (87.24%, 670/768) identified human clinics or hospitals as places where they could obtain anti-rabies injections if they were bitten by a suspected rabid dog (Table 3). Others identified veterinary clinics or veterinary hospitals (5.08%, 39/768) or supermarkets (1.30%, 10/768) as possible sources for anti-rabies injections following a dog bite.

**Table 3:** Proportion of respondents based on their responses to knowledge questions.

Knowledge variables	Level	Frequency (n)	Percentage %
At what age do pets (such as dogs or cats) start to receive their vaccination?	Immediately from birth	122	15.89
	From 3 months	324	42.19
	From 12 months	100	13.02
	Never	2	0.26
	Don’t know	220	28.65
Where can you obtain an anti-rabies injection if you are a victim of a dog bite?	Veterinary clinic or Hospital	39	5.08
	Any supermarket	10	1.30
	Human clinic or hospital	670	87.24
	Don’t know	49	6.38
Is it possible for rabies to be transmitted from animals to humans?	Yes	640	83.33
	No	30	3.91
	Don’t know	98	12.76
Is it advisable for someone to get anti-rabies treatment after a bite from a suspected rabid dog?	Yes	607	79.04
	No	25	3.26
	Don’t know	135	17.58
	Did not answer	1	0.13
If you are vaccinated against rabies after being bitten by a rabid dog, will that protect you from developing rabies?	Yes	551	71.74
	No	44	5.73
	Don’t know	170	22.14
	Did not answer	3	0.39

The results showed that 79.56% (611/768) of the respondents were aware of the importance of pet vaccination in controlling rabies among humans. Albeit few, there were respondents (1.69%, 13/768), who indicated that pet vaccination was not important for controlling rabies in humans.

Despite the high number of respondents (83.33%, 640/768) who were aware that rabies could be transmitted to humans from animals, up to 12.76% (98/768) of the study population did not know that rabies could be transmitted from animals to humans. In fact, some respondents were of the view that rabies could not be transmitted from animals to humans (3.91%, 30/768) (Table 3).

Most respondents (79.04%, 607/768) were aware of the need to seek immediate medical attention after a bite from a suspected rabid dog. However, when respondents were asked whether receiving a rabies vaccine after being bitten by a suspected rabid dog offered protection against the development of rabies, the number who responded in the affirmative dropped to 71.74% (551/768) (Table 3).

Less than half of the respondents (42.19%, 324/768) indicated that a 3-month-old dog was eligible to receive its initial rabies vaccination. This was followed by 28.65% (220/768) who did not know the age at which puppies can receive their initial rabies vaccination and 15.89% (122/768) who thought that puppies can receive their initial rabies vaccination immediately after birth (Table 3).

**Fig 1:**
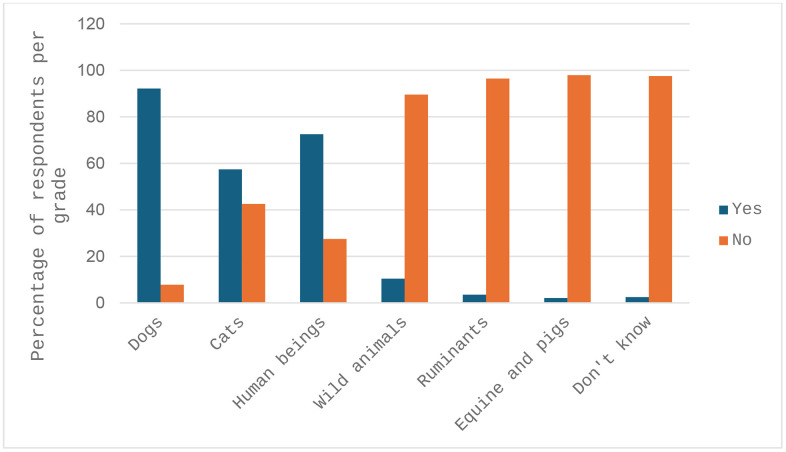
Proportion of respondents based on their responses to questions regarding animals susceptible to rabies.

Regarding the species that are susceptible to rabies, dogs (92.2%, 708/768) were the most frequently mentioned. Cats were mentioned by just over half of the respondents (57.42%, 441/768) (Fig 1).

**Fig 2:**
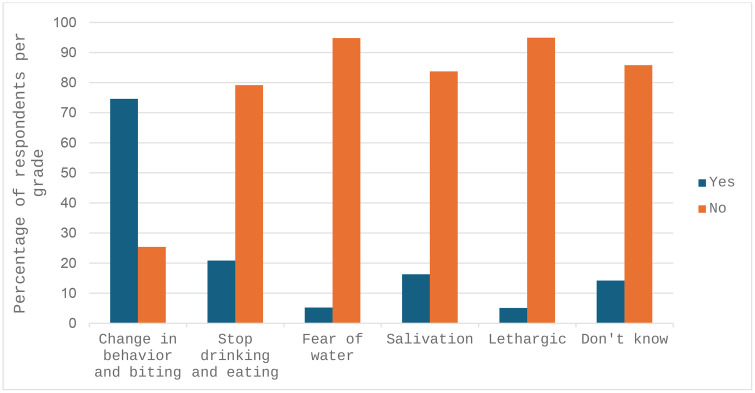
Percentage of respondents based on their responses to questions about the clinical signs of rabies in dogs.

Among the clinical signs that can be seen in a suspected rabid dog, change in behaviour, the inability of a dog to drink and eat and salivation were the most frequently mentioned (Fig 2).

#### 4.1.3. Attitudes and practices towards rabies.

As shown in Table 4, the majority of the respondents (81.77%, 628/768) indicated that it is necessary to receive an anti-rabies injection after a dog bite. Meanwhile, some respondents were not aware of, or did not think, that it was necessary to receive an anti-rabies injection after a dog bite.

**Table 4:** Results of the attitude and practices among respondents towards rabies as a disease.

Variables	Levels	Frequency (n)	Percentage %
Do you think it is necessary to receive an anti-rabies injection after a dog bite?	Yes	628	81.77
	No	24	3.13
	Don’t know	115	14.97
	Did not answer	1	0.13
What should someone who has been bitten by a suspected rabid dog do following a bite?	Nothing	30	3.91
	Leave the wound to heal	20	2.60
	Seek medical attention	692	90.10
	Purchase medication for the wound	25	3.26
	Did not answer	1	0.13
What should be done to a dog that has bitten someone?	Nothing	183	23.83
	Kill the dog	104	13.54
	Chase the dog away	77	10.03
	Quarantine the dog	403	52.47
	Did not answer	1	0.13
Do you think it is appropriate to destroy the dog if it is suspected of having rabies?	Yes	303	39.45
	No	265	34.51
	Don’t know	198	25.78
	Did not answer	2	0.26
Did you sterilise your pet?	Yes	149	26.65
	No	410	73.35
Do you have a copy of the vaccination certificate for your pet?	Yes	459	82.11
	No	100	17.89
How often do you vaccinate your dog?	Only once in a lifetime	30	5.37
	Never	8	1.43
	At different intervals	374	66.91
	Don’t know	147	26.30
Do you keep your dog in a fenced place?	Yes	446	79.79
	No	113	20.21
	Don’t have a pet	209	27.21
Do you ever allow your dog to go out of the yard unsupervised?	Yes	227	40.61
	No	332	59.39
	Don’t have a pet	209	27.21
Do you always vaccinate your dog when there are annual vaccination campaigns?	Yes	494	88.37
	No	65	11.62
	Don’t have a pet	209	27.21
Do you vaccinate your dog even though it is always restrained?	Yes	480	85.87
	No	79	14.13
	Don’t have a pet	209	27.21
How do you know if an animal has rabies?	When the animal is lazy	102	13.28
	You only know by laboratory results	137	17.84
	Wait until it suddenly dies	13	1.69
	If it bites people when confronted	272	35.42
	Don’t know	244	31.77
What do you do when you see a suspected rabid stray dog	Nothing	227	29.56
	Kill the dog and throw it away or bury it	80	10.42
	Chase the dog away	50	6.51
	Report it to the Department of Agriculture and/or the SPCA)	411	53.52

Regarding what should be done to a dog that has bitten someone, as shown in Table 4, just over half expressed the view that such an animal should be quarantined. The other respondents were of the view that nothing should be done to the dog or that such a dog should be chased away. Meanwhile, a small number (13.54%, 104/768) indicated that such a dog should be killed.

On the question of whether destroying a dog suspected of having rabies was appropriate, only 39.45% (303/768) agreed that euthanising a dog suspected of having rabies was the appropriate measure in such circumstances. Some respondents did not think that a dog should be euthanised because it was suspected to be rabid (Table 4).

High dog or pet ownership (72.79%, 559/768) was observed in the study area. However, among those who owned pets, very few (26.65%, 149/559) indicated that they had sterilised their pets.

In response to the question on how often dogs are vaccinated, as presented in Table 4 the overwhelming majority indicated that dogs are vaccinated at different intervals. Twenty-six percent (26.2%, 147/559) did not know the frequency with which dogs are vaccinated. A small number indicated that their dogs were vaccinated only once in their lifetime or that their dogs were never vaccinated.

The majority (82.22%, 459/559) of the respondents who owned pets produced a vaccination certificate as proof that their pets had been vaccinated against rabies. The remaining 17.89% (100/559) failed to show vaccination certificates for their pets (Table 4).

The majority of the respondents (85.87%, 480/559) who owned dogs indicated that they vaccinated their dogs even if the dogs were always restrained. On the contrary, a much smaller number of respondents did not think it was worthwhile vaccinating their dogs if they were always restrained (Table 4).

Fewer than half (40.61%, 227/559) of the respondents who owned dogs indicated that they allowed their dogs to go outside the yard unsupervised. On the contrary, a higher percentage (79.79%, 446/559) of the same dog owners indicated that they always kept their dogs in fenced premises to prevent them from going outside unsupervised (Table 4).

Most of the respondents who owned dogs indicated that they always vaccinated their dogs during annual vaccination campaigns (88.37%, 494/559). A very small number indicated that they did not always take their dogs for vaccination during rabies vaccination campaigns (Table 4).

As indicated in Table 4, very few respondents (17.84%, 137/768) were aware that only laboratory results can be used to declare an animal positive for rabies. On the contrary, a higher number of respondents were not aware of how to tell if an animal has rabies or believed that animals that bite people after confrontation should be presumed to be rabid (35.42%, 272/768).

When asked what they would do if they encountered a suspected rabid stray dog, just over half (53.52%, 411/768) said that they would report the dog as a suspected rabies case to the Department of Agriculture and Rural Development and/or the SPCA.

**Fig 3:**
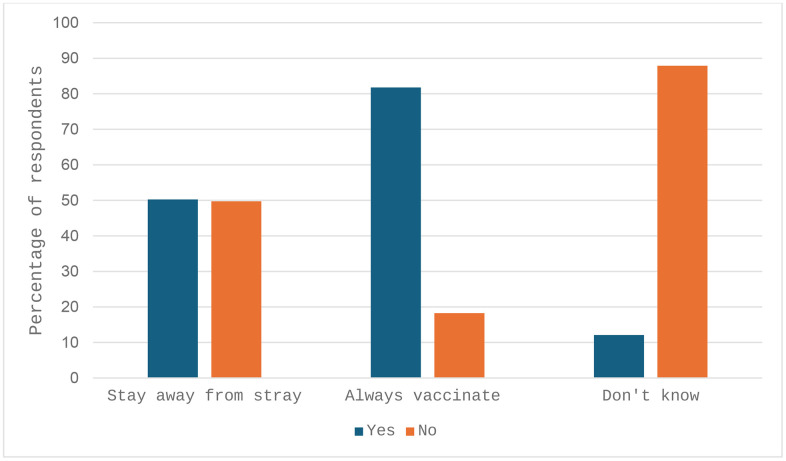
Bar graph showing the proportion of respondents based on their attitudes towards rabies in eThekwini District.

As shown in Fig 3, the overwhelming majority of respondents indicated that animals should always be vaccinated to avoid rabies outbreaks. Meanwhile, a few respondents identified avoiding contact with stray animals as a way to protect themselves from contracting rabies. It was surprising to note that some respondents did not know what to do to protect themselves from contracting rabies (Fig 3).

### 4.2. Inferential statistics

Age, pet/dog ownership, religion, and educational level were the only variables significantly associated with the outcome in the univariable analysis at a p-value of ≤0.20. These were thus included in the multivariable logistic regression model to identify the factors significantly associated with being knowledgeable about rabies.

Only two variables, owning a pet and age of the respondents, were retained in the final multivariable logistic regression model. Respondents who owned pets were approximately three times as likely to be knowledgeable about rabies as those who did not own pets, the reference group (AOR = 2.79; p = 0.0001) (Table 5).

**Table 5:** Results of the analysis of factors correlated with being knowledgeable about rabies^a^.

Variable	Coefficient estimate	Standard error	Adjusted Odds Ratio	95% CI		*p*-value
**Own Pet?**				**Lower**	**Upper**	
No	**Ref**					
Yes	1.03	0.26	2.79	1.72	4.74	0.0001
**Age (Years)**						
>71	**Ref**					
18-35	−0.07	1.10	0.93	0.15	18.01	0.9490
36-53	0.60	1.10	1.82	0.29	35.17	0.5870
54-71	0.27	1.14	1.32	0.19	26.36	0.8098

^a^ Only variables retained in the final model are shown.

Age was not a significant predictor of knowledge among the respondents (p > 0.05). However, it was retained in the final effects model because it proved to be a confounder.

## 5. Discussion

### 5.1. Knowledge of rabies as a disease

The results revealed that there was a widespread lack of knowledge about the cause of rabies. This was not expected, given the high percentage of people in the study area who could read and write. A population that can read and write is amenable to training, given that most training materials on rabies are in English. Considering the observed findings, there is a need to place emphasis on the cause of rabies during extension programmes in the study area to improve the proportion of people who know the cause of the disease.

If a dog licks an open wound, it is advisable to seek medical attention immediately. However, a suspected rabid animal licking an open wound was not considered important by the respondents in this study. This means that respondents are not likely to seek medical care, placing members of the community at a heightened risk of contracting rabies infection. In view of this, dogs licking open wounds need to be emphasised as a possible rabies transmission route. It should also be emphasised that, in the event of a suspected rabid dog licking an open wound, the victim should wash the wound immediately with soap and water.

The low number of participants, that is, just over half, who indicated that washing a dog bite wound with soap and running water was an important part of first aid that should be administered to dog bite victims to help prevent the development of rabies, is a serious public health concern. This is because failure to wash a dog bite wound immediately with soap and water as part of PEP allows the virus to enter the victim’s body, thus increasing the risk of the victim developing rabies.

A much lower proportion of people considered rabies to be a fatal disease than was reported by Bihon et al. [33] and Abedela and Teshome [4]. The latter observed that 84.6% (325/384) and 82% (123/150) of participants, respectively, were aware that it is not possible to cure rabies once clinical signs appear. This finding suggests that people in the study area are unlikely to seek medical care early enough following a dog bite. This kind of information ought to be central to the message passed on to the community.

Although 78% (601/768) of respondents in the current study were aware of the need to obtain anti-rabies treatment following a dog bite, from a public health point of view, this is still a source of concern. Everyone should know that receiving anti-rabies treatment immediately following a bite by a dog suspected of having rabies is important if rabies-related deaths are to be prevented. This is particularly important considering the high dog ownership observed in the study area. As a result, receiving the anti-rabies vaccine immediately following a dog bite is another area that needs to be emphasised as part of the extension message.

Studies conducted elsewhere have shown that the role of other animal species or wildlife in the spread of rabies is not well known. For example, in Kansas and Gondar Zuria District, 96.8% and 100% [34,35], respectively, of the respondents did not mention other animal species or wildlife besides dogs as playing a role in the spread of rabies. Similarly, in the present study, the role of other animal species or wildlife in the spread of rabies was mentioned less frequently. The widespread lack of knowledge of the role played by other animal species in the spread of rabies points to a gap in the education programmes to which communities are exposed in the study area. This therefore warrants emphasis on the role played by other animal species in the spread of rabies during community awareness programmes.

Vaccination of pets against rabies enhances herd immunity and helps interrupt the transmission chain of the rabies virus among animals, as well as between animals and humans. Therefore, the high number of respondents who were aware of the importance of vaccinating dogs to control rabies in this study is encouraging. That notwithstanding, compared to places such as Jabalpur, central India [36], a lower proportion of respondents in the current study viewed vaccination of dogs as being important in protecting humans. In view of this, there is a need to implement measures that can help increase the number of people who understand the importance of vaccinating pets.

Rabies campaigns in South Africa target puppies from 3 months of age and older as part of the eradication scheme within the country. This could explain the high proportion of people who were in favour of vaccinating their pets at 3 months. The high number of people who were in favour of their pets being vaccinated at 3 months could also be attributed to the rigour with which rabies eradication campaigns are conducted in the study area. Furthermore, compared to the low numbers of people who were willing to vaccinate their dogs at an early age in Rwanda (20.6%, 22/107) and the Democratic Republic of Congo (14.7%, 60/407) [7,37], the findings of the present study attest to the impact of rabies prevention campaigns in the study area.

It is encouraging to note the high number of respondents (87.24%) who preferred to be treated at a human clinic or hospital following exposure to a dog bite. However, the fact that some respondents indicated that they did not know where to go for treatment following exposure, or that they would get treatment from a veterinary clinic, veterinary hospital, or supermarket, means that more effort is needed to ensure that everyone knows the right place to go for treatment following exposure. This is important if the area is to realise the goal of eliminating dog-mediated rabies deaths in humans.

Considering the fact that rabies is endemic in the region where the study was conducted, it was encouraging to note that most respondents were aware that rabies can be transmitted from animals to humans. This is consistent with findings from a study done in the Tigray region of Ethiopia, where 89.5% indicated that humans were likely to contract rabies from animals [38]. At the same time, it is concerning to note that some respondents were not aware that they could contract rabies from dogs. This places such people at a high risk of contracting rabies. Therefore, to realise the zero rabies-related deaths in humans in line with the WHO 2030 strategy, urgent attention to this matter is needed.

The importance of obtaining anti-rabies vaccination following a bite by a suspected rabid dog was widely known among the respondents. This contrasts with what was observed in Odisha, India [39] and Ethiopia [34], both known to be endemic for rabies, where only 14.9% (50/336) and 38.8% (55/400), respectively, were aware of the importance of receiving anti-rabies vaccination following exposure to a dog bite. This further demonstrates the strong impact of the rabies control programme implemented by the authorities in the study area.

While a change in the behaviour of the dog, including biting people, was widely reported by participants as a major clinical sign of rabies, paralysis was not commonly cited as a clinical sign of rabies. This means that dogs presenting with paralysis are likely to be overlooked as suspected rabies cases. Therefore, if a rabid dog showing paralytic signs were to bite a person, such a person would be unlikely to seek PEP, which increases the risk of contracting rabies among the respondents.

### 5.2. Attitude and practices of community members towards rabies

Only 51% (204/400) of respondents in a study conducted in Berhampur, Odisha, India, a country that accounts for 36% of the world’s rabies deaths, were willing to obtain anti-rabies treatment following a dog bite [39]. On the contrary, a high proportion of respondents in the present study were willing to receive the anti-rabies vaccine following a dog bite. This is encouraging because a high number of people in a community who are willing to receive the anti-rabies vaccine has the potential to diminish the risk of members of the community developing rabies following exposure to bites by a rabid dog.

A healthy attitude among study participants towards seeking medical attention following exposure to a dog bite was observed in the present study. This is evidenced by the many respondents (90.10%, 692/768) who were willing to seek medical attention following exposure to bites by rabid dogs. Compared to what was reported in South Gondar, Ethiopia, where the overwhelming majority (79.4%, 305/384) were not in favour of visiting healthcare centres following dog bites [33], the results of the present study are encouraging and point to the effectiveness of the extension programs in the study area.

Isolating animals or placing them under quarantine if they display abnormal behaviour, such as biting someone, is very important in preventing rabies outbreaks. This is because of its role in the early detection of rabies outbreaks, which contributes to the protection of members of the community and their dogs from contracting rabies. Therefore, the high number of respondents in this study who favoured quarantining their animals if they displayed abnormal behaviour is a significant finding from a public health point of view.

Destroying a suspected rabid dog was not widely supported by the respondents in this study. This contrasts with what has been observed in other rabies-endemic areas, such as South Gondar (64.3%, 247/384) and Kombolcha (84.1%, 323/384), where a higher percentage were willing to kill dogs suspected of having rabies [5,33]. It is therefore clear that more needs to be done in the study area to improve the attitude of the population towards putting down dogs that are suspected of being rabid.

The low sterilisation rate reported in the present study suggests that conditions in the area are conducive to the development of a large population of breeding dogs. If an area has many dogs that are not neutered, it could end up with many dogs roaming in the area, especially during the mating season. Therefore, attention must be given to educating dog owners about the importance of neutering their dogs. This is important considering that poor sterilisation rates among dogs have also been reported by other researchers elsewhere, which shows that low sterilisation rates, despite the associated dangers, is a common problem that needs attention. For example, a study conducted in Sri Lanka by Ubeyratne et al. [40] reported that up to 86.41% (674/780) of the respondents had not sterilised their pets.

The vaccination coverage in the study area, measured by the number of respondents who had valid vaccination cards, was above 70%. According to WHO, this is the minimum vaccination coverage needed to increase herd immunity in dogs to a level that prevents rabies virus transmission among dogs or animals and eventual transmission to humans [41]. This is a positive finding worth noting, and it suggests that the risk of humans contracting rabies from dogs in the study area is minimal. Nonetheless, more effort is needed to increase the vaccination coverage in the area to cover all dogs.

Vaccination of pets following guidelines provided by the State was widely supported in the study area, as evidenced by the high number of respondents (75.91%, 583/768) who indicated a willingness to vaccinate their dogs following state guidelines. This is an important finding because adhering to repeated and regular rabies vaccination helps boost the immunity levels of pets and thus minimises outbreaks of rabies.

While, in the study by Shirsuphal [42], 84% (56/67) of participants did not let their dogs go outside the homestead, very few participants in the present study indicated that they kept their dogs in an area that was fenced off and did not allow dogs to go outside the compound. This observation is a serious public health concern because, when domestic dogs are allowed to roam freely, they frequently encounter stray animals that might be rabid. This heightens the risk of these dogs contracting the rabies virus. Therefore, confinement of dogs to minimise encounters between domestic dogs and stray dogs or wild animals in the study area needs to be improved.

Regularly presenting dogs for vaccination during vaccination campaigns has been widely adopted, as shown by the very high proportion (88.37%, 494/559) of dog owners in the present study who were willing to have their dogs regularly vaccinated. This clearly contrasts with what has been observed in the Philippines and South Gondar, where only 64% and 69.8%, respectively, consistently vaccinated their dogs [27,36]. This is a welcome finding and could be attributed to the regular farmers’ days organised in the study area to educate and carry out rabies vaccination campaigns. The observed high number of people, willing to consistently present their dogs for vaccination in the present study, explains the more than 70% vaccination coverage recommended by WHO [41] that was recorded.

Given that the study area is endemic for rabies, it is concerning that some people did not present their dogs for vaccination during each campaign. This may be due to individuals being unavailable because of work or other commitments, while others were unable to handle their dogs and therefore did not present them for vaccination. The responsible authority needs to consider this and design innovative ways to reach such dogs.

It is evident that presenting the carcass of a suspected rabid dog to the laboratory to confirm whether the dog had rabies is not well appreciated in the study area. This is supported by the few respondents in the study who indicated that they would present the carcass of a suspected animal to the laboratory to confirm whether it had rabies. However, the number of people who would take the carcass of a dead dog to the laboratory for confirmatory diagnosis in the present study is higher than the 2% (22/1056) in the Democratic Republic of Congo study who indicated that they would take the carcass of a suspected rabid animal to the veterinary services for laboratory testing [37]. Although comparatively, the findings observed in this study are encouraging, more effort is required to ensure that carcasses of animals suspected of having rabies are submitted for laboratory confirmation. This is necessary for early outbreak detection and improving surveillance efforts.

The number of participants who indicated that they would report a suspected rabies-positive case to the authorities was extremely low. In fact, only 53.52% (411/768) of the respondents in the area indicated that they would report a suspected rabies case. Areas that are far from places where suspected cases can be reported are likely to experience low levels of compliance and should therefore be targeted during campaigns to emphasise the need to report suspected rabies cases.

### 5.3. Factors significantly associated with knowledge of rabies

The finding that respondents who owned pets had significantly higher odds of being knowledgeable about rabies than respondents who did not own pets is consistent with the findings by Abdela et al. [6]. The latter also observed that respondents who owned dogs had significantly (*p* = 0.029) higher odds of obtaining a good KAP score than those who did not own dogs. Similar results were observed among residents of the Abuja municipal area, where it was found that people who owned dogs were 7.8 times more likely to have better knowledge of rabies than those who had no dogs [21]. The phenomenon of dog owners being more knowledgeable about rabies, could be attributed to the fact that people who own pets are more likely to receive information during rabies campaigns when they take their dogs for vaccination.

## 6. Conclusion and recommendations

The study area has attained vaccination coverage of over 70%, which, according to the WHO, is the level needed to break the rabies virus transmission chain among dogs and prevent transmission to humans. However, the identified knowledge gaps have the potential to negate the benefits the community stands to gain from the high vaccination coverage. The identified knowledge gaps include poor knowledge of the cause of rabies, how one can protect oneself from contracting rabies, precautionary measures to adopt following exposure to rabies, and how to protect susceptible animals from being exposed to rabies. Measures such as erecting fences around homesteads to restrict animal movements and neutering pets to control the dog population need to be emphasised during awareness programmes.

The study identified owning a dog as the only socio-demographic factor that predicted a high knowledge score for rabies. In light of this, information dissemination needs to be all-inclusive to reach non-pet owners, who also constitute a population at risk. On a positive note, the study demonstrated that there was widespread knowledge of some aspects, such as the role of dog bites in spreading rabies in the study area, which suggests that people are likely to seek medical attention in the event of a dog bite. In addition, a good attitude towards receiving PEP following a dog bite and reporting a suspected case was widespread in the study.

To bridge the gaps identified in this study, the implementation of global strategies, such as awareness and vaccination campaigns, needs to be stepped up. Information to sensitise people can be accessed through government-distributed pamphlets, by watching the If Only I Knew video, and by consulting Government Veterinary Services (GVS). In addition, programmes to reinforce the positive aspects identified in this study should be implemented continuously. This will help improve control and prevention efforts directed towards minimising and eradicating rabies outbreaks, including associated human fatalities. A One Health approach that involves collaboration between veterinary services, other public health institutions, and local authorities to expand and strengthen rabies awareness should be adopted in the study areas to achieve the 2030 goal of rabies elimination in rabies-endemic areas.

## 7. Limitations

This study was subject to the limitations of cross-sectional surveys, such as recall bias. Furthermore, the study was limited to Verulam and Embo in eThekwini District; hence, the results cannot be generalised to other areas in South Africa. The study was conducted during the lockdown due to the COVID-19 pandemic, when the movement of people was restricted and people were reluctant to meet strangers, which could have led to selection bias. This thus calls for caution when interpreting the results. Nonetheless, this study presents baseline information on knowledge, attitudes, and practices towards rabies, which was previously unavailable in the study area. It also provides baseline information upon which future research can be developed.

## Supporting information

S1 File This is the Supplementary material I. This is The questionnaire.
(PDF)

S2 File This is the Supplementary material II. This is The Ethics approval certificate.
(PDF)
