# Supplementary material for: Knowledge, attitude, and practices towards rabies: A community survey in selected areas of KwaZulu-Natal Province, South Africa
Source: PLoS One. 2026 Jul 9;21(7):e0352279. doi: 10.1371/journal.pone.0352279 (PMC13349125; doi:10.1371/journal.pone.0352279)
Supplement: S1 File — This is The questionnaire. (PDF) [file pone.0352279.s001.pdf]

QUESTIONNAIRE

RABIES IN TWO COMMUNITIES IN ETHEKWINI DISTRICT IN KWAZULU-NATAL PROVINCE OF SOUTH AFRICA: KNOWLEDGE, ATTITUDE AND PRACTICES (KAP).

Aim

The aim of the study is to assess the knowledge and attitude of pet-owners and non-pet owners on rabies disease and also investigate practices associated with rabies in rural and urban communities in eThekwini District in KwaZulu-Natal Province of South Africa.

1. **Demographic data**

   1.1 What is your age?
   1. 18-35
   2. 36-50
   3. 51-70
   4. Above 70

   1.2 Sex of the respondent?
   1. Male
   2. Female

   1.3 Where is your residential setting?
   1. Verulam (urban)
   2. Embo (rural)

   1.4 Do you have a pet dog and/or cat?
   1. Yes
   2. No

   1.5 What is your religion?
   1. Christian
   2. African religion
   3. Hindu
   4. Muslim
   5. Other

   1.6 What is your educational level?
   1. No education
   2. Completed primary
   3. Secondary not completed
   4. Completed Matric
   5. Tertiary completed

2. **Knowledge questions**

   2.1 What is the cause of rabies disease?
   1. Chemical substances
   2. Virus
   3. Insufficient intake of feed and water

4. Psychological problem
5. Don’t know

2.2 How can human beings become infected with rabies?
1. Dog-bite
2. Playing with the dog
3. Feeding the dog
4. Don’t know

2.3 What is the first thing to do after dog-bite while at home?
1. Cover the wound with bandage
2. Cleanse the wound with soap and running water
3. Apply any topical medication
4. Don’t know

2.4 Can rabies be cured beyond appearance of clinical signs?
1. Yes
2. No
3. Don’t know

2.5 When is it appropriate to receive anti-rabies injection after being bitten by a dog suspected of having rabies?
1. Later
2. Immediately
3. Any time
4. Don’t know

2.6 Which of the following are susceptible to rabies?
1. Dogs
2. Cats
3. Human beings
4. Wild animals
5. Ruminants
6. Equine and pigs
7. Don’t know

2.7 Is vaccination of pets important for preventing human disease?
1. Yes
2. No
3. Don’t know

2.8 At what age do pets (dogs or cats) start to receive their vaccination?
1. Immediately from birth
2. From 3 months, again within 12 months, annually or every 3 years.
3. From 12 months, again every 3 years
4. Never
5. Don’t know

2.9 Where can you obtain anti-rabies injections if you are a victim of dog bite?
1. Veterinary clinic or Hospital
2. Any supermarket
3. Human clinic or Hospital

4. Don’t know

2.10 Is it possible for rabies to be transmitted from animals to humans?
1. Yes
2. No
3. Don’t know

2.11 Is it advisable for someone to get anti-rabies after a bite from a suspected rabid dog?
1. Yes
2. No
3. Don’t know

2.12 If you are vaccinated against rabies after being bitten by a rabid dog, will that protect you from developing rabies?
1. No
2. Yes
3. Don’t know

2.13 Which clinical signs can make you to suspect rabies in dogs?
1. Change in behaviour and biting
2. Stop eating and drinking
3. Fear of water
4. Salivating
5. Laziness/Lethargic/Paralysis
6. Don’t know

3. Attitude towards animal bite

3.1 Do you think it is necessary to receive anti-rabies injection after a dog bite?
1. Yes
2. No
3. Don’t know

3.2 What should someone who has been bitten by a suspect rabid dog do following a bite?
1. Nothing
2. Cleanse the wound
3. Seek medical attention
4. Purchase medication for the wound
5. Report to a traditional healer

3.3 What should be done to dog that has bitten someone?
1. Nothing
2. Kill the dog
3. Chase the dog away
4. Quarantine the dog and observe for 7-14 days to see if it dies and also inform the Department of Agriculture (Veterinary services)/ Private veterinarian/Society for the Prevention of Cruelty to Animals (SPCA)

3.4 Do you think it is appropriate to put-down the dog if it is suspected to have rabies?

1. Yes
2. No
3. Don’t know

4. **Practices of safety towards rabies**

4.1 Did you sterilise your pet?
1. No
2. Yes
3. Don’t have

4.2 Do you have a copy of certificate of vaccination for your pet, to produce?
1. Yes
2. No
3. Don’t have a pet

4.3 How often can you vaccinate your dog?
1. Only once in a lifetime
2. Never
3. Different interval
4. Don’t know

4.4 How do you keep safe from contracting rabies?
1. Staying away from stray animals
2. Always vaccinate your pets
3. Don’t know

4.5 Do you keep your dog in a fenced place?
1. Yes
2. No
3. Don’t have

4.6 Do you ever allow your dog to go out of the yard unsupervised?
1. Yes
2. No
3. Don’t have

4.7 Do you always vaccinate your dog when there are ongoing campaigns?
1. Yes
2. No
3. Don’t have

4.8 Do you vaccinate your dog even though it is always restrained?
1. No
2. Yes
3. Don’t have

4.9 How do you know if an animal has rabies?
1. When the animal is lazy
2. You only know if you send the animal to the Laboratory
3. Wait until it suddenly dies
4. If it bites people when confronted
5. Don’t know

4.10 What do you do when you see a suspect rabies stray dog?

1. Nothing
2. Kill the dog and throw or bury
3. Chase the dog away
4. Report to the Department of Agriculture/Society for the Prevention of Cruelty to Animals (SPCA).
