# Supplementary material for: Knowledge, attitude, and practices towards rabies: A community survey in selected areas of KwaZulu-Natal Province, South Africa
Source: PLoS One. 2026 Jul 9;21(7):e0352279. doi: 10.1371/journal.pone.0352279 (PMC13349125; doi:10.1371/journal.pone.0352279)
Supplement: S2 File — This is The Ethics approval certificate. (PDF) [file pone.0352279.s002.pdf]

UNISA-CAES HEALTH RESEARCH ETHICS COMMITTEE

Date: 11/06/2019

NHREC Registration # : REC-170616-051
REC Reference # : 2019/CAES_HREC/105
Name : Mr MT Letsoalo
Student #: 51084473

Dear Mr Letsoalo

Decision: Ethics Approval from 06/06/2019 to 31/05/2020

Researcher(s): Mr MT Letsoalo
51084473@mylife.unisa.ac.za

Supervisor (s): Prof JW Oguttu
joguttu@unisa.ac.za; 011-471-3353

Prof CA Mbajiorgu
mbajica@unisa.ac.za; 011-471-3590

Working title of research:

Rabies in rural and urban communities in Ethekwini municipality in Kwazulu-Natal Province of South Africa: Knowledge, attitude and practice (KAP)

Qualification: MSc Agriculture

Thank you for the application for research ethics clearance by the Unisa-CAES Health Research Ethics Committee for the above mentioned research. Ethics approval is granted for a one-year period. After one year the researcher is required to submit a progress report, upon which the ethics clearance may be renewed for another year.

Due date for progress report: 31 May 2020

*The **minimal risk application** was **reviewed** by the UNISA-CAES Health Research Ethics Committee on 06 June 2019 in compliance with the Unisa Policy on Research Ethics and the Standard Operating Procedure on Research Ethics Risk Assessment.*

The proposed research may now commence with the provisions that:

1. The researcher(s) will ensure that the research project adheres to the values and principles expressed in the UNISA Policy on Research Ethics.
2. Any adverse circumstance arising in the undertaking of the research project that is relevant to the ethicality of the study should be communicated in writing to the Committee.
3. The researcher(s) will conduct the study according to the methods and procedures set out in the approved application.
4. Any changes that can affect the study-related risks for the research participants, particularly in terms of assurances made with regards to the protection of participants' privacy and the confidentiality of the data, should be reported to the Committee in writing, accompanied by a progress report.
5. The researcher will ensure that the research project adheres to any applicable national legislation, professional codes of conduct, institutional guidelines and scientific standards relevant to the specific field of study. Adherence to the following South African legislation is important, if applicable: Protection of Personal Information Act, no 4 of 2013; Children's act no 38 of 2005 and the National Health Act, no 61 of 2003.
6. Only de-identified research data may be used for secondary research purposes in future on condition that the research objectives are similar to those of the original research. Secondary use of identifiable human research data require additional ethics clearance.
7. No field work activities may continue after the expiry date. Submission of a completed research ethics progress report will constitute an application for renewal of Ethics Research Committee approval.

Note:
The reference number ***2019/CAES_HREC/105*** *should be clearly indicated on all forms of communication with the intended research participants, as well as with the Committee.*

Yours sincerely,

Prof MA Antwi
Chair of UNISA-CAES Health REC
E-mail: antwima@unisa.ac.za
Tel: (011) 670-9391

Prof MJ Linington
Executive Dean : CAES
E-mail: lininmj@unisa.ac.za
Tel: (011) 471-3806

University of South Africa
Preller Street, Muckleneuk Ridge, City of Tshwane
PO Box 392 UNISA 0003 South Africa
Telephone: +27 12 429 3111 Facsimile: +27 12 429 4150
www.unisa.ac.za
