# Supplementary material for: Knowledge, attitude, and practices towards rabies: A community survey in selected areas of KwaZulu-Natal Province, South Africa
Source: PLoS One. 2026 Jul 9;21(7):e0352279. doi: 10.1371/journal.pone.0352279 (PMC13349125; doi:10.1371/journal.pone.0352279)
Supplement: Attachment — Submitted filename: Response to Reviewers.pdf [file pone.0352279.s005.pdf]

Response to editor

Please delete "prospective" from line 18, "of the respondents." from line 23 and "treatment" from line 25.

Response: corrected as advised

PEP includes wound washing, a dose of human rabies immune globulin (HRIG), and rabies vaccine given at the time of the first medical visit, and two booster doses. Please correct the statement at line 25.

Response: Corrected as advised-see lines 25-27

Please delete "rate" from line 56 as it is a proportion.

Response: "rate" deleted from line 58

Please replace all "Univariate" and "multivariate" with "univariable" and "multivariable" throughout the manuscript.

Response: corrected as advised.

Line 160: Please delete "binary".

Response: Corrected as advised-see line 162

Please mention the software used for the logistic regression analysis. Lines 318-322: Univariable results are not final, so please don't describe them.

Response: The following statement has been added under data analysis (lines 156-157)

Data was analysed using the Statistical Package for Social Science (SPSS) Version 27 (SPSS for Windows, version 27, SPSS, Inc, Chicago, Illinois, the USA).

Results of univariable regression have been removed as advised.
