# Supplementary material for: Knowledge, attitude, and practices towards rabies: A community survey in selected areas of KwaZulu-Natal Province, South Africa
Source: PLoS One. 2026 Jul 9;21(7):e0352279. doi: 10.1371/journal.pone.0352279 (PMC13349125; doi:10.1371/journal.pone.0352279)
Supplement: Attachment — Submitted filename: PONE-D-23-27745_R3_reviewerComments.pdf [file pone.0352279.s007.pdf]

PLOS ONE

Knowledge, attitude and practices towards rabies: A community survey in selected areas of KwaZulu-Natal Province, South Africa

--Manuscript Draft--

**Manuscript Number:**	PONE-D-23-27745R3
**Article Type:**	Research Article
**Full Title:**	Knowledge, attitude and practices towards rabies: A community survey in selected areas of KwaZulu-Natal Province, South Africa
**Short Title:**	Knowledge, attitude and practices towards rabies
**Corresponding Author:**	James Wabwire Wabwire Oguttu, PhD<br>University of South Africa<br>Johannesburg, Gauteng SOUTH AFRICA
**Keywords:**	Rabies vaccination; rabies disease; rabies awareness; dog-bite; post-exposure prophylaxis
**Abstract:**	Background: Knowledge, attitude, and practices (KAP) studies have been widely used to assess gaps towards rabies. However, there is no evidence of baseline studies that have assessed the KAP towards rabies among selected communities in KwaZulu-Natal Province, South Africa.<br>Methods: A prospective cross-sectional questionnaire-based study design was adopted for this study. Participants (n=768) were selected using systematic random sampling. Data from respondents (≥18 years) was collected using a pretested questionnaire and analysed using descriptive and inferential statistics. Significance was set at α≤0.05.<br>Results: Twenty seven percent (26.95%) of the respondents of the respondents could name the cause of rabies, while just over half (53.52%) of the respondents indicated that they would adhere to post exposure prophylaxis (PEP) treatment which included washing the wound with soap and running water. Most respondents (90.10%) mentioned that they would seek medical attention following a dog bite. Only 59.77% of respondents were able to produce a vaccination certificate for their pets. Allowing dogs to roam without supervision was widely reported by the respondents. Dog ownership was the only factor significantly associated with knowledge of rabies.<br>Conclusion: Lack of knowledge of the cause of rabies and the need to receive PEP immediately after exposure to a dog bite was widespread in the study area. However, gaps in some aspects related to knowledge about rabies were observed that require awareness creation for dog owners, including those who do not own pets.
**Order of Authors:**	James Wabwire Wabwire Oguttu, PhD
	Mohube Titus Letsoalo, Diploma (Animal Health), BTech (Animal Health)
	Nenene Daniel Qekwana, PhD
	Christian A Mbajiorgu, PhD
	Jaja Ishmael Festus, PhD
**Response to Reviewers:**	We have revised the manuscript and provided a point by point explanation of how we responded to the Academic Editor's comment and suggestions. We have also formated the paper according to the templated for PLOSONE. If the paper is accepted for publication we will make the data available on the institutional repository.
**Additional Information:**	
**Question**	**Response**
**Financial Disclosure**<br><br>Enter a financial disclosure statement that describes the sources of funding for the work included in this submission. Review	The authors received no specific funding for this work.

Powered by Editorial Manager® and ProduXion Manager® from Aries Systems Corporation

the submission guidelines for detailed requirements. View published research articles from *PLOS ONE* for specific examples.<br><br>This statement is required for submission and **will appear in the published article** if the submission is accepted. Please make sure it is accurate.<br><br>**Unfunded studies**<br>Enter: *The author(s) received no specific funding for this work.*<br><br>**Funded studies**<br>Enter a statement with the following details:<br>• Initials of the authors who received each award<br>• Grant numbers awarded to each author<br>• The full name of each funder<br>• URL of each funder website<br>• Did the sponsors or funders play any role in the study design, data collection and analysis, decision to publish, or preparation of the manuscript?<br>• **NO** - Include this sentence at the end of your statement: *The funders had no role in study design, data collection and analysis, decision to publish, or preparation of the manuscript.*<br>• **YES** - Specify the role(s) played.<br><br>* typeset	
**Competing Interests**<br><br>Use the instructions below to enter a competing interest statement for this submission. On behalf of all authors, disclose any competing interests that could be perceived to bias this work—acknowledging all financial support and any other relevant financial or non-financial competing interests.<br><br>This statement is required for submission and **will appear in the published article** if the submission is accepted. Please make sure it is accurate and that any funding sources listed in your Funding Information later in the submission form are also	The authors have declared that no competing interests exist.

declared in your Financial Disclosure statement.<br><br>View published research articles from *PLOS ONE* for specific examples.<br><br>**NO authors have competing interests**<br><br>Enter: *The authors have declared that no competing interests exist.*<br><br>**Authors with competing interests**<br><br>Enter competing interest details beginning with this statement:<br><br>*I have read the journal's policy and the authors of this manuscript have the following competing interests: [insert competing interests here]*<br><br>* typeset	
**Ethics Statement**<br><br>Enter an ethics statement for this submission. This statement is required if the study involved:<br><br>• Human participants<br>• Human specimens or tissue<br>• Vertebrate animals or cephalopods<br>• Vertebrate embryos or tissues<br>• Field research<br><br>Write "N/A" if the submission does not require an ethics statement.<br><br>General guidance is provided below. Consult the submission guidelines for detailed instructions. **Make sure that all information entered here is included in the Methods section of the manuscript.**	The Ethics committee of the College of Agriculture and Environmental Science, University of South Africa approved this study (REC Reference #: 2019/CAES_HREC/105). Written consent was onbtained from each participant before completing the questionnaire.

**Format for specific study types**<br><br>**Human Subject Research (involving human participants and/or tissue)**<br>• Give the name of the institutional review board or ethics committee that approved the study<br>• Include the approval number and/or a statement indicating approval of this research<br>• Indicate the form of consent obtained (written/oral) or the reason that consent was not obtained (e.g. the data were analyzed anonymously)<br><br>**Animal Research (involving vertebrate animals, embryos or tissues)**<br>• Provide the name of the Institutional Animal Care and Use Committee (IACUC) or other relevant ethics board that reviewed the study protocol, and indicate whether they approved this research or granted a formal waiver of ethical approval<br>• Include an approval number if one was obtained<br>• If the study involved *non-human primates*, add *additional details* about animal welfare and steps taken to ameliorate suffering<br>• If anesthesia, euthanasia, or any kind of animal sacrifice is part of the study, include briefly which substances and/or methods were applied<br><br>**Field Research**<br><br>Include the following details if this study involves the collection of plant, animal, or other materials from a natural setting:<br>• Field permit number<br>• Name of the institution or relevant body that granted permission	
**Data Availability**<br><br>Authors are required to make all data underlying the findings described fully available, without restriction, and from the time of publication. PLOS allows rare exceptions to address legal and ethical concerns. See the PLOS Data Policy and FAQ for detailed information.	Yes - all data are fully available without restriction

A Data Availability Statement describing where the data can be found is required at submission. Your answers to this question constitute the Data Availability Statement and **will be published in the article**, if accepted.<br><br>**Important:** Stating 'data available on request from the author' is not sufficient. If your data are only available upon request, select 'No' for the first question and explain your exceptional situation in the text box.<br><br>Do the authors confirm that all data underlying the findings described in their manuscript are fully available without restriction?	
**Describe where the data may be found in full sentences. If you are copying our sample text, replace any instances of XXX with the appropriate details.**<br><br>• If the data are **held or will be held in a public repository**, include URLs, accession numbers or DOIs. If this information will only be available after acceptance, indicate this by ticking the box below. For example: *All XXX files are available from the XXX database (accession number(s) XXX, XXX.*).<br>• If the data are all contained **within the manuscript and/or Supporting Information files**, enter the following: *All relevant data are within the manuscript and its Supporting Information files.*<br>• If neither of these applies but you are able to provide **details of access elsewhere**, with or without limitations, please do so. For example:<br><br>*Data cannot be shared publicly because of [XXX]. Data are available from the XXX Institutional Data Access / Ethics Committee (contact via XXX) for researchers who meet the criteria for access to confidential data.*<br><br>*The data underlying the results presented in the study are available from (include the name of the third party*	The data are held in a public repository, Figshare. The DOI link is referred to below:<br><br>https://doi.org/10.25399/UnisaData.26110657.v1

Powered by Editorial Manager® and ProduXion Manager® from Aries Systems Corporation

and contact information or URL).<br>• This text is appropriate if the data are owned by a third party and authors do not have permission to share the data.<br><br>* typeset	
Additional data availability information:	

Powered by Editorial Manager® and ProduXion Manager® from Aries Systems Corporation

Manuscript
Click here to access/download;Manuscript;Manuscript.docx

Knowledge, attitude, and practices towards rabies: a community survey in selected areas of KwaZulu-Natal Province, South Africa.

Mohube Titus Letsoalo[1,2¶], Daniel Nenene Qekwana[3&], Christian. A. Mbajiorgu[1&], Ishmael Festus Jaja[1&], James Wabwire Oguttu[1¶*]

[1]Department of Agriculture and Animal Health, College of Agriculture and Environmental Sciences, University of South Africa, Johannesburg, South Africa

[2]KwaZulu-Natal Department of Agriculture and Rural Development, Durban, KZN, South Africa

[3]Department of Paraclinical Science, Faculty of Veterinary Science, University of Pretoria, Pretoria, South Africa.

*Correspondence

Email: joguttu@unisa.ac.za

Abstract

Knowledge, attitude, and practices (KAP) studies have been widely used to assess gaps towards rabies. However, there is no evidence of baseline studies that have assessed the KAP towards rabies among selected communities in KwaZulu-Natal Province, South Africa. A cross-sectional questionnaire-based study design was adopted for this study. Participants (n=768) were selected using systematic random sampling. Data from respondents (≥18 years) was collected using a pretested questionnaire and analysed using descriptive and inferential statistics. Significance was set at $\alpha \leq 0.05$. Twenty seven percent (26.95%) of the respondents could name the cause of rabies, while just over half (53.52%) of the respondents indicated that they would adhere to post exposure prophylaxis (PEP) which, includes wound washing, a dose of human rabies immune globulin (HRIG), and rabies vaccine given at the time of the first medical visit, and two booster doses . Most respondents (90.10%) mentioned that they would seek medical attention following a dog bite. Only

59.77% of respondents were able to produce a vaccination certificate for their pets. Allowing dogs to roam without supervision was widely reported by the respondents. Dog ownership was the only factor significantly associated with knowledge of rabies. Lack of knowledge of the cause of rabies and the need to receive PEP immediately after exposure to a dog bite was widespread in the study area. However, gaps in some aspects related to knowledge about rabies were observed that require awareness creation for dog owners, including those who do not own pets.

Keywords: Rabies, vaccination, rabies disease, rabies awareness, dog-bite, post-exposure prophylaxis, PEP, Risk factors

1. INTRODUCTION

Domestic dogs play a significant role in the lives of humans. But on the other hand, they pose the greatest threat of transmitting rabies to humans [9]. Rabies is an important infectious viral disease [4], caused by the lyssavirus, from the family of *Rhabdoviridae* [16, 20]. All warm-blooded mammals are susceptible to the rabies virus infection generally transmitted through the bite of a rabid animal [29].

Dog mediated rabies infection remains a significant disease from a public health perspective [33] and annually it is responsible for an estimated 60 000 human deaths worldwide [22, 28]. Of these, Asia has the highest burden of rabies, followed by Africa. Making the two the most affected continents. In Africa, over 90% of human rabies deaths are associated with dog bites [33, 42]. In Asia alone, approximately 31,539 human deaths are reported annually suggesting a high burden of rabies in the continent ([3]. Furthermore, globally, around 15 million people seek rabies post-exposure prophylaxis (PEP) due to the threat of rabies infection following exposure [30].

Once symptoms of rabies develop, victims usually succumb to the disease [2]. This is especially true if the victim fails to obtain PEP early following a dog bite [14]. In humans potentially exposed to the rabies virus, PEP usually includes thorough wound cleaning and a combination of treatment with rabies immunoglobulin and vaccination [44]. Without proper health care, the loss of life of untreated victims is inevitable. Moreover, the disease is reported to have the highest case fatality of any disease [33].

Rabies is a preventable disease, and vaccination of dogs through mass campaigns is one of the effective ways to control the disease at its source [22]. According to the World Health Organization (WHO), a minimum of 70% dog vaccination coverage can drastically reduce the number of human cases within countries where rabies is endemic [26]. However, in most developing countries, rabies continues to be a challenge resulting in high mortalities in humans and animals [25]. This is despite existence of effective human and animal rabies vaccines [33]. This could be attributed to inadequate efforts to control and prevent the spread of the disease in these countries [14].

In South Africa, an estimated 40 human rabies related deaths from dog bite contacts occur yearly [44]. This points to the fact that measures needed to successfully prevent and control the spread of rabies within the dog population are inadequate. This is especially true in the province of KwaZulu-Natal (KZN), which has the highest cases of dog rabies in the country [23]. In addition, rabies is well established and remains endemic in the province [34]. This is supported by the recent increase in the number of laboratory confirmed positive animal cases in KZN [44]. Moreover, most of such cases of rabies have been confirmed in dogs [23]. Besides KZN Province, the Eastern Cape and Limpopo provinces are also considered by the Department of

Agriculture, Land Reform and Rural Development (DALLRD) as having high numbers of rabies.

Knowledge, attitude and practices studies are significant in obtaining information on the subject of concern [32]. Therefore, questions on the cause, source and mode of transmission, clinical signs and prevention practices and treatment measures are important in determining participants' knowledge, attitude and practices (KAP) regarding rabies [21]. In addition, knowledge of how to handle a suspected rabid dog is important [10], and measures that must be adopted when bitten by a rabid dog, need to be assessed if measures to prevent and control rabies are to be effective [45].

In fact, good knowledge, attitude and practices about rabies have been shown to reduce the spread of the disease and the fatalities associated with the disease [16]. In view of this, the present study investigated the KAP of communities in two areas (Embo and Verulam) of KwaZulu-Natal Province of South Africa.

2. METHODS

2.1 Study area

The study was conducted within eThekwini District in KwaZulu-Natal Province of South Africa. Kwazulu-Natal is one of the nine provinces in South Africa and is a multi-racial community. The population of KZN is approximately 10,819,130 living on an area of 94,361 km². Its annual population growth is estimated to be 1, 2 % [24].

The eThekwini District is occupied by about 3.7 million people on a land division of approximately 2,555 km² [15]. Embo (Latitude: 29° 54' 34'' S, Longitude: 30° 40' 29'' E) is a rural area situated in the inlands of eThekwini in the western region of the district, with an estimated population size of approximately 40,924 people, and an estimated 8,184 households. Verulam (Latitude: -29° 38' 59.99'' S; Longitude: 31° 02'

60.00" E) on the other hand is an urban area located in the upper part of the northern region [15]. Verulam has an estimated 64,950 households (Unpublished data, Verulam Environmental Health Manager), and approximately 184,114 occupants on a land of 18.13 km² ([15].

2.2 Study design

A cross-sectional study design was adopted to investigate the knowledge, attitude and practices of community members towards rabies in eThekwini District in KZN province of South Africa.

2.3 Target population

The target population for this study was the residents of Embo (a rural area) and Verulam (an urban area). The study included people of different age groups, religious beliefs, and educational backgrounds. People of both sex (male or female) and both pet owners and non-pet owners were also included in the study.

2.4 Sampling strategy

A systematic random sampling method was used to sample from the two selected areas (Embo and Verulam). In both areas, a sampling interval was predetermined before the selection of households commenced. The first household was selected randomly, thereafter, the successive households were selected following the adopted interval for each of the areas until the number required was reached. However, if an adult was absent at the time of interviewing, the next house with an adult (≥18 years) was chosen to be interviewed [40].

2.5 Sample size determination

This sample size from the two study areas (Embo and Verulam) was determined using the formula described by Gaudu et al. [21] :

$$N = \frac{1.96^2 \times Pexp(1 - Pexp)}{(d)^2}$$

N= Sample size

P_{exp}- Estimate or expected proportion of knowledge about rabies from community was considered at 50%, given that there is no baseline study regarding KAP in the study area.

d^2- Desired absolute precision (5%).

A total of 768 respondents (384 from each of the two areas) were recruited and agreed to voluntarily participate in the study.

2.6 Data collection

The questionnaire developed by Fenelon et al. [17] was adapted for this study. Data was collected using a structured questionnaire during face-to-face interviews. The questionnaire had the following sections: respondents' demographic data, Knowledge of rabies, Attitude towards rabies and Practices.

The questionnaire was translated into the local language (isiZulu) that is most widely spoken to cater for respondents who could not communicate in English. In addition, the questionnaire was pre-tested on fifteen participants from each area to test its fitness for purpose. Each interview session lasted 30-45 minutes and was conducted in English or IsiZulu. Administration of the questionnaire took place from 01/08/2019 to 31/03/2021.

2.7 Data management and data analysis

2.7.1 Data management

Each questionnaire was reviewed carefully after each data collection session to ensure that it was filled in correctly, and afterwards each question was coded. All raw data was immediately captured into the computer using Microsoft Excel 365

(Microsoft Corp., Redmond 137, USA). The correct response to each of the questions on knowledge was awarded a score of 1, while 0 was given for the incorrect answer. Thereafter, the total score for each respondent was computed and presented as a percentage (knowledge score). A binary variable was then created from the knowledge score (knowledgeable=1 and not knowledgeable=0).

2.7.2 Data analysis

Data was analysed using the Statistical Package for Social Science (SPSS) Version 27 (SPSS for Windows, version 27, SPSS, Inc, Chicago, Illinois, the USA). Descriptive statistics such as frequencies and percentages of all categorical variables were computed and presented in graphs and tables. Inferential analysis involved first fitting a series of univariate binary logistic regression models to test for simple associations between the predictor variables and the outcome variable (Knowledge). Variables with alpha level ≤ 0.20 in the univariable model were included in the multivariable model. Significance in the multivariable model was assessed at $p < 0.05$.

A multivariable logistic regression model was then fit to the data to assess the factors that were significantly associated with being knowledgeable about rabies. Using the backward selection method, variables to retain in the final regression model were identified. If removal of a variable from the model resulted in a ≥20% change in the effects measure of any of the variables in the model, the variable was considered a confounder and was thus retained in the final effects model, regardless of whether it was significantly associated with the outcome variable or not.

Models were compared using the Log likelihood ratio test. The model fit of the final effects model was tested using a Hosmer-Lemeshow test. Based on the results, there was a good model fit ($p > 0.05$).

3. Ethical consideration

The State Veterinary services of KwaZulu-Natal Department of Agriculture and rural development granted approval to conduct the study. In addition, permission to carry out the study was granted by the Ethics committee of the College of Agriculture and Environmental Science, University of South Africa (REC Reference #: 2019/CAES_HREC/105). The identity of the respondents was protected by ensuring that they remained anonymous. The objectives of the study were first explained to the respondents, and thereafter each participant signed the consent form to indicate that they voluntarily accepted to participate in the study. All respondents had the right to withdraw from the study at any time during the study. Findings of this study will be disseminated to the residents of the study areas during rabies vaccination campaigns and presented at conferences to the broader scientific community and researchers.

4. RESULTS

4.1 Descriptive statistics

4.1.1 Demographic profile of respondents

An equal number of respondents (384) was drawn from each of the identified areas (Embo and Verulam). Therefore, a total of 768 community members were invited to participate in the study.

Majority of the respondents were females (63.54%), aged between 18 – 35 years (53.21%), belonged to the Christian faith (47.66%), and had not completed secondary education (35.81%). There was a high pet ownership (72.79%) in the study area (Table 1).

Table 1: **Demographic profile of people who agreed to participate in the study**

Variables	Level	Frequency (n)	Percentage %	95% Confidence Interval	
				Lower interval	Upper interval
Age	18 – 35	409	53.26	49.65	56.83
	36 -53	288	37.50	34.06	41.03
	54 - 71	64	8.33	6.48	10.52
	>71	7	0.91	0.37	1.87
Sex	Male	280	36.46	33.05	39.97
	Female	488	63.54	60.03	66.95
Residence	Verulam	384	50.00	46.40	53.60
	Embo	384	50.00	46.40	53.60
Own a pet dog and/ or cat	Yes	559	72.79	69.49	75.91
	No	209	27.21	24.09	30.51
Religion	Christian	366	47.66	44.07	51.26
	African religion	140	18.23	15.56	21.15
	Hindu	204	26.56	23.47	29.84
	Muslim	31	4.04	2.76	5.68
	Other	27	3.52	2.33	5.07
Education	No education	43	5.60	4.08	7.47
	Completed primary	101	13.15	10.84	15.75
	Secondary not completed	275	35.81	32.41	39.31
	Completed matric/High school Education	234	30.47	27.23	33.86
	Tertiary completed	115	14.97	12.52	17.70

4.1.2 Knowledge of rabies disease

Results of the assessment of knowledge of rabies are presented in Table 2 and Table 3. Half (50.52%; n= 388) of respondents indicated that they did not know the cause of rabies, with only 26.95% (n=207) of the participants indicating that rabies is caused by a virus. Although in the minority, it was interesting to note that some respondents (2.47%, n=19) thought that rabies is caused by a chemical substance.

Table 2: The results of the assessment of the knowledge of rabies among respondents

Variables	Level	Frequency (n)	Percentage %
What is the cause of rabies?	Chemical substances	19	2.47
	Virus	207	26.95
	Insufficient intake of feed and water	77	10.03
	Psychological problem	77	10.03
	Don’t know	388	50.52

How can human be infected with rabies?	Dog-bite	668	86.98
	Playing with the dog	15	1.95
	Feeding the dog	0	0
	Don't know	85	11.07
What is the first thing to be done after dog-bite while at home?	Cover the wound with bandage	96	12.50
	Cleanse the wound with soap and running water	411	53.52
	Apply any topical medication	134	17.45
	Don't know	127	16.54
Can rabies be treated beyond appearance of clinical signs?	Yes	387	50.39
	No	123	16.02
	Don't know	258	33.59
When is it appropriate to receive anti-rabies injection after being bitten by a dog suspected of having rabies?	Later after weeks	27	3.52
	Immediately	601	78.26
	Any time after months	41	5.34
	Don't know	99	12.89
Is vaccination of pets important for preventing human rabies?	Yes	611	79.56
	No	13	1.69
	Don't know	144	18.75

Dog-bite was identified by most respondents (86.98%, n=668) as the likely rout for the transmission of rabies. In response to the question on the first thing to be done after a dog-bite while at home, slightly over half of the respondents (53.52%, n=411) indicated that they would clean the wound with soap and running water (Table 2).

Although 50.39% (n=387) of respondents were not aware that rabies can be fatal beyond appearance of clinical signs. It was also noted that most respondents (78.26%, n=601) would immediately seek the provision of anti-rabies injections following a bite by a suspect rabid animal. However, 12.89% (n=99) did not know that they should get anti-rabies injections immediately, while 5.34% (n=41) indicated that it could be given later; say after weeks or even months later (3.52%, n=27).

Majority of respondents indicated that human clinics or hospitals (87.24%, n=670) are the places they can obtain anti-rabies injection in case they were bitten by a suspected rabid dog. A few indicated the veterinary clinic or veterinary hospital (5.08%, n=39) or supermarkets (1.30%, n=10) as possible sources where anti-rabies injections could be obtained following a dog bite.

The results show that 79.56% (n=611) of the respondents were aware that vaccination of pets is important for controlling rabies among humans. This was followed by 18.75% (n=144) who did not know that vaccination of pets is important to prevent humans contracting the disease. Meanwhile, a very small percentage (1.69% (n=13) indicated that vaccination of pets was not important for controlling rabies in humans.

Although a high number of respondents (83.33%, n=640) were aware that rabies could be transmitted to humans from animals, up to 12.76% (n=98) of the study population, expressed ignorance about rabies being transmitted from animals to humans. In addition, some respondents said that rabies could not be transmitted from animals to humans. However, these were very few (3.91%, n=30) (Table 3).

Most respondents (79.04%, n=607) were aware the need for someone to seek medical attention after a bite from a suspected rabid dog. However, the number of positive responses slightly dropped to 71.74% (n=551) when respondents were asked if vaccination against rabies after being bitten by a suspected rabid dog offers protection against development of rabies (Table 3).

Less than half (42.19%; n=324) of the respondents indicated that a 3-month-old dog was eligible to receive the start-up rabies vaccination. This was followed by 28.65% (n=220) who did not know the age at which puppies can get their start-up rabies vaccination and 15.89% (n=122) who thought that puppies can get their start-up rabies vaccination immediately after birth (Table 3).

Table 3: The results of the assessment of the knowledge of rabies

Knowledge variables	Level	Frequency (n)	Percentage %
At what age do pets (dogs or cats) start to receive their vaccination?	Immediately from birth	122	15.89
	From 3 months	324	42.19
	From 12 months	100	13.02

	Never	2	0.26
	Don’t know	220	28.65
Where can you obtain anti-rabies injection if you are a victim of dog bite?	Veterinary clinic or Hospital	39	5.08
	Any supermarket	10	1.30
	Human clinic or Hospital	670	87.24
	Don’t know	49	6.38
Is it possible for rabies to be transmitted from animals to humans?	Yes	640	83.33
	No	30	3.91
	Don’t know	98	12.76
Is it advisable for someone to get anti-rabies after a bite from a suspected rabid dog?	Yes	607	79.04
	No	25	3.26
	Don’t know	135	17.58
	Did not answer	1	0.13
If you are vaccinated against rabies after being bitten by a rabid dog, will that protect you from developing rabies?	Yes	551	71.74
	No	44	5.73
	Don’t know	170	22.14
	Did not answer	3	0.39

In response to the question on the species that are “susceptible to rabies?”. Most respondents (92,2%) mentioned dogs. This was followed by 72,53% who indicated that human beings could contract rabies and just over half (57,42%) who mentioned cats were susceptible to rabies (Fig 1).

Fig 1: Distribution of respondents by responses to questions regarding animals susceptible to rabies.

In response to the question on clinical signs that can make them suspect rabies in dogs, most respondents mentioned (74,61%) signs such as change in behaviour, followed by the inability of a dog to drink and eat (20,83%), and salivation (16,28%) (Fig 2).

Fig 2: The percentage of respondents based on how they responded to the question about the clinical signs of rabies in dogs.

4.1.3 Attitude and practices towards the disease rabies

Results of the attitudes and practices towards rabies are summarised below. Majority of respondents (81.77%, n=628) indicated that it was necessary to receive anti-rabies injection after a dog bite. This was followed by 4.97%; n=115) who did not know that it was necessary to receive anti-rabies, and 3.13% (n=24) did not think that it was necessary to receive anti-rabies injection after a dog bite.

With regards to what should be done to a dog that has bitten someone, 52.47% (n=403) were of the view that such animal should be quarantined. This was followed by 23.83% (n=183) who were of the view that noting should be done to the dog and 13.54% (n=104) and 10.03% (n=77) who indicated that the dog should be killed or chased away respectively.

On the question of whether putting-down a dog suspected of having rabies was appropriate, only 39.45% (n=303), agreed that putting down the dog was the appropriate measure in such circumstances. This was followed by 34.51% (n=265) who did not think that putting down a dog in such circumstances was appropriate, and 25.78% (n=198) who did not know if putting down a dog in such circumstances was appropriate (Table 4).

Under thirty percent (27.21%; n=209) of the study population did not have pets. But among those who owned pets, slightly over half (53.39%, n=410) did not sterilise their pets.

In response to the question how often their dogs get vaccinated 75.91% (n=583) vaccinate their dogs at different intervals. This was followed by 19.14% (n=147), who were not aware of the frequency with which their dogs get vaccinated. Some respondents vaccinated their dogs only once in its lifetime (3.91%; n=30), or never

vaccinate their dogs (1.04%; n=8) (Table 4). Meanwhile, only 59.77% (n=459) of the respondents were able to produce the vaccination certificate as proof that their pets had been vaccinated against rabies.

Sixty-three percent (62.50%; n=408) of the respondents were of the view that vaccination of pets (dog or cat) was important even if the pet is always restrained. In contrast, 10.29% (n=79) did not think it was worthwhile vaccinating their dogs if they are always restraint.

With regards to allowing dogs going out of the yard unsupervised, 43.23% (n=332) did not allow their dogs to go out of the yard unsupervised, while 43.23% (n=332) indicated that they let their dogs go out of the yard unsupervised. But regarding keeping dogs in a fenced place, just over half (58.07%; n=446) indicated that they kept their dogs in a fenced premises to prevent the dogs from going outside unsupervised. The majority (64.32%, n=494) of respondents indicated that they always vaccinated their pets during annual vaccination campaigns, while 8.46% (n=65), indicated that they did not always take their dogs for vaccination during the rabies vaccination campaigns (Table 4).

As indicated in Table 4 below, only 17.84% (n=137) respondents were aware that only laboratory results can be used to declare the animal positive for rabies. However, a higher number (35.42%; n=272) indicated that animals that bite people after confrontation, should be presumed to be rabid. In response to the question what to do when a suspect rabies stray dog is encountered, just over half (53.52%; n=411) said that they would report the dog as a rabies suspect to the department of Agriculture/ or SPCA.

Table 4: Results of the attitude and practices among respondents towards rabies disease

Variables	Levels	Frequency (n)	Percentage %
Do you think it is necessary to receive anti-rabies injection after a dog bite?	Yes	628	81.77
	No	24	3.13
	Don’t know	115	14.97
	Did not answer	1	0.13
What should someone who has been bitten by a suspect rabid dog do following a bite?	Nothing	30	3.91
	Leave the wound to heal	20	2.60
	Seek medical attention	692	90.10
	Purchase medication for the wound	25	3.26
	Did not answer	1	0.13
What should be done to dog that has bitten someone?	Nothing	183	23.83
	Kill the dog	104	13.54
	Chase the dog away	77	10.03
	Quarantine the dog	403	52.47
	Did not answer	1	0.13
Do you think it is appropriate to put-down the dog if it is suspected to have rabies?	Yes	303	39.45
	No	265	34.51
	Don’t know	198	25.78
	Did not answer	2	0.26
Did you sterilise your pet?	Yes	149	19.40
	No	410	53.39
	Don’t have	209	27.21
Do you have a copy of certificate of vaccination for your pet, to produce?	Yes	459	59.77
	No	100	13.02
	Don’t have a pet	209	27.21
How often can you vaccinate your dog?	Only once in a lifetime	30	3.91
	Never	8	1.04
	Different interval	583	75.91
	Don’t know	147	19.14
Do you keep your dog in a fenced place?	Yes	446	58.07
	No	113	14.71
	Don’t have	209	27.21
Do you ever allow your dog to go out of the yard unsupervised?	Yes	227	29.56
	No	332	43.23
	Don’t have	209	27.21
Do you always vaccinate your dog when there are annual vaccination campaigns?	Yes	494	64.32
	No	65	8.46
	Don’t have a pet	209	27.21
Do you vaccinate your dog even though it is always restrained?	Yes	480	62.50
	No	79	10.29
	Don’t have	209	27.21
How do you know if an animals has rabies?	When the animal is lazy	102	13.28
	You only know by laboratory results	137	17.84
	Wait until it suddenly dies	13	1.69
	If it bites people when confronted	272	35.42
	Don’t know	244	31.77
What do you do when you see a suspect rabies stray dog?	Nothing	227	29.56
	Kill the dog and throw or bury	80	10.42
	Chase the dog away	50	6.51
	Report to Department of Agriculture/ SPCA)	411	53.52

Respondents were asked a question on how they keep safe from contracting rabies. Majority of respondents (81,77%) indicated they always vaccinate their animals to avoid rabies. Slightly over half (50,26%) of the respondents indicated that they would stay away from stray animals to avoid contracting rabies. Only 12,11% did not know what should be done to remain safe from rabies (Fig 3).

Figure 3: A bar graph showing the proportion of respondents based on their attitude towards rabies in eThekwini District.

4.2 Inferential statistics

Age, owning a pet, religion and educational level were the only variables that were significantly associated with the outcome in the univariable analysis at a generous P-value of ≤ 0.2. These were thus included in the multivariable logistic regression model to identify the factors that were significantly associated with being knowledgeable about rabies.

Only two variables (owning a pet and age of the respondents) were retained in the final multivariable logistic regression model. The respondents who owned pets compared to those who did not, were 3 times (AOR = 2.788; p-value=0.0001) as likely to be knowledgeable about rabies compared to those who did not own pets (reference group) (Table 5).

Age was not a significant (p-value >0.05) predictor of knowledge among the respondents. However, it was retained in the final effects model because it was a confounder.

Table 5: The results of multivariable analysis showing factors that were associated with being knowledge about rabies

Variable	Coefficient estimate	Standard error	Adjusted Odds Ratio	95 % CI		P-value
**Own Pet?**				Lower	Upper	
No	**Ref**					
Yes	1.0254	0.2570	2.788	1.721	4.737	0.0001
**Age**						
>71	**Ref**					
18-35	-0.0706	1.1034	0.932	0.149	18.009	0.9490
36-53	0.5991	1.1030	1.821	0.291	35.170	0.5870
54-71	0.2741	1.1388	1.315	0.191	26.364	0.8098

5. DISCUSSION

This is the first study to investigate the knowledge, attitude, and practices toward rabies disease among respondents in the study area. Few respondents were aware that rabies is caused by a virus. Dog bites was identified by a large proportion of the respondents as the main transmission mode for rabies to humans. But on the other hand, just over half of the respondents interviewed mentioned that washing of the wound following a dog bite with soap and running water was important for preventing rabies disease. Very few respondents were aware that licking of an open wound by a suspected rabid dog is one of ways in which rabies spreads from dogs to humans. Similarly, a very low number of respondents in the present study indicated that they knew that rabies disease was fatal. However, many respondents were aware of the importance of receiving anti-rabies vaccine immediately after being bitten by a dog suspected of having rabies to protect the bite victims from developing rabies. Only pet ownership was significantly associated with a high knowledge score for rabies.

5.1 Demographic profile of respondents

This study found that over half of respondents (53.26%) were 18-35 years of age. This the age group that is likely to be more active when it comes to pet management in the homes. Given that more females (63.54%) agreed to participate in the study compared to males, it is possible that females take more keen interest in matters that relate to their pets. However, this observation contrasts with findings by

Bihon et al., [6] and Abdela and Teshome [2], who observed that male respondents constituted 71.4% and 59.3% of the study population respectively.

The findings of the present study suggest a high dog/pet ownership in the study area. For example, in comparison to the 72.79% who owned dogs in the present study, only 53% of the respondents owned dogs in a study done in Shirsuphal in western India [39]. Findings from the present study also contrast with those from a study conducted in Cameroon in which less than half of the respondents owned pets [9].

In this study, just below half (47.66%) of respondents were Christians, contrary to findings of studies conducted in Kombolcha and Dedo districts which reported Muslims proportion of 56.8% and 94.8% respectively [1, 20]. The observed difference could be attributed to differences in the dominant religions in the different study areas.

The number of people who had not completed secondary education (35.81%) or had completed matric (high school) (30.47%) in the study population was lower than the number of people who had formal education. However, this is much higher than what Abdela et al. [1] reported. The later observed that half of their study population (51.1%) did not have a formal education. Similarly, the number of people with education higher than primary schooling in the current study was higher than the 52,5% reported in the survey done in Berhampur, Odisha that had only primary school level education [41]. The high number of people who could read and write in the present study is an encouraging finding because it points to a target population that is amiable to training given that a lot of training material on rabies is in English.

5.2 Knowledge of the disease rabies

The very low number (26.95%; n=207) of respondents who were aware that a virus was the agent responsible for rabies, is in line with results from the study

conducted in Dessie City, where only 18% (n=25) of respondents regarded a virus to be the cause of rabies [19]. However, in a study conducted in Kombolcha, Ethiopia, a higher number (76.6%; n=294) of respondents were aware that rabies is caused by a virus [20]. This difference suggests a need to improve on the training of residents in the study area and particularly make it clear what causes rabies.

A very high number of respondents (86.98%) who mentioned dog bite as a mode of transmission for rabies to humans is an encouraging finding from a public health point of view. This aligns with findings from a study by Fesseha et al. [18], which reported almost a similar proportion (85%) of respondents who mentioned that rabies virus was transferred through a bite from an infected animal. However, a slightly lower number from the Philippines (79.5%) than those observed in the present study, were aware that dog bites play a crucial role in the transfer of rabies to people [5]. Similarly, a study conducted in Haiti also observed that 75.6% of respondents knew that rabies can be transmitted via a dog bite [17]. The findings of this study shows that the knowledge about the transmission of rabies in the study is comparable to that observed in other developing countries such as Ethiopia and the Philippines. On the other hand, results of this study indicate more people than what has been reported in other countries were knowledgeable about the transmission of rabies.

Licking of an open wound by a dog as mode of transmitting rabies, was mentioned by a mere 11.46% of the respondents in the present study. Similarly, a very low (1.8%) number of community members in a study conducted in Haiti were aware that rabies could be transmitted to people through contact of saliva with an open wound [17].

While most respondents mentioned biting as one of the ways the virus gains entry to the victim, licking of the open wound by the suspected animal was not

considered important in the spread of rabies. This suggest that if the dog licked humans in the study area, they would probably not attempt to seek medical care. This would hence put them at risk of rabies infection.

Just over half (53.52%) of this study's respondents considered washing the wound with soap and running water as the first aid to prevent rabies. This low number, is of concern, given the importance of post exposure prophylaxis that includes washing the wound immediately with soap and water to prevent infection gaining entry to the victim. In contrast, a higher number (92.4%), of respondents from the study in and around South Gondor in Northwest Ethiopia indicated that washing the wound with soap was the first measure to be implement following a bite by a suspected rabid dog before seeking other preventative measures [20]. Findings reported here also contrasted with those reported by Bihon et al [6], who observed that a very low number of respondents (8.9%) regarded wound washing as an immediate response to prevent rabies. In view of the findings of the present study, while the number of people who acknowledge that washing the bite wound was important as the first step in the prevention of rabies is higher than what Bihon et al. [6] observed in South Gondor. Therefore, there is room for improvement on understanding how to prevent rabies in case of a bite among residents in the study area.

In this study, only 16% of respondents considered rabies a fatal disease. This figure is much lower than the findings from Bihon et al [6] and Abedela and Teshome [2], who observed that 84.6% and 82% of people, respectively, were aware that rabies cannot be cured once clinical signs appear. The fact that only a few respondents in this study were aware that rabies is not treatable, is significant and suggests people are most likely not to seek medical care early. It is critical that this kind of information

be included in campaigns to educate the community about the seriousness of the disease rabies.

A considerable number of respondents (78%) in the present study knew that they need to obtain anti-rabies vaccine immediately after being bitten by dog suspected of having rabies. A slightly lower response (65.2%) was reported in Dedo district of Jimma zone, Ethiopia [1]. Even a much lower number was reported by Abdela and Teshome [2] who observed that only 32.7% of the respondents in their study indicated that anti-rabies should be administered immediately after exposure. Although a much higher number of respondents in this study were aware of the importance of receiving anti-rabies vaccines immediately after being bitten by a suspect dog, there is room for improvement to bring the number closer to 100%. The authors believe that everyone should know that receiving anti-rabies treatment immediately after one has been bitten by a dog suspected of having rabies is necessary to eradicate rabies related deaths. The difference observed between the different studies suggest a difference in the effectiveness of extension services in the different study areas. It is evident from the findings reported here that there is room for improvement in the study area when it comes to the importance of receiving anti-rabies vaccine immediately following a dog bite.

Lack of knowledge among the general public on the role other species besides dogs play in the spread of rabies, seems to be widespread. For example, although in this study a large number (90%) of community members mentioned dogs as being susceptible to rabies, other animal species were mentioned less frequently. Similarly, a high proportion of respondents in other studies conducted in Kansas (96.8%) and Gondar Zuria District (100%), largely pointed to dogs as being the one that is susceptible to rabies [11, 27]. Only a study conducted in Harare Metropolitan reported

few respondents (37.59%) who identified dogs as the only animal susceptible to rabies [36]. The widespread lack of knowledge of the role played by other species besides dogs in the spread of rabies, points to a gap in the education programmes communities are exposed to. Therefore, there is a need to place emphasis on the role other species play in the spread of rabies during extension to communities.

The over three quarters (79.75%) of the respondents in this study who were aware that vaccinating pets was significant to keep humans safe from rabies, is consistent with findings of other studies. For example, most respondents (68%) from Jamma also believed that vaccination against rabies in dogs helps prevent the disease in humans [38]. Dubey et al. [12] reported a slightly higher proportion of respondents who believed that dog vaccination was a good practice in a study conducted in Jabalpur central India. The high number of respondents who were aware of the importance of vaccinating dogs to control rabies is good news. This is because vaccination of pets creates a barrier of diseases shared among pets and humans given that they occupy the same space.

The slightly less than half (42.19%) of the respondents in the present study who favoured vaccinating their pets at 3 months old, was a bit higher than the finding of a study conducted in Kigali city, Rwanda, were only 20.6% of respondents vaccinated their puppies by the age 3 months [31]. On the other hand, Bouaddi et al. [7] reported a much lower percentage of respondents (14.7%) who favoured having their dogs vaccinated at 3 months of age. However, in the Democratic Republic of Congo up to 14% of respondents believed that young puppies should not be vaccinated [27]. The differences observed in studies conducted elsewhere and the present study, the higher figures observed in this study, could be attributed to the rigour with which rabies eradication campaigns are conducted in the study area. In fact, in South Africa, rabies

campaigns target puppies from 3 months of age and older as part of the eradication scheme aimed at eliminating rabies within the country.

The very high number of respondents (87.24%) in this study who preferred to be treated from a human clinic or hospital after exposure to a dog bite, is a welcome observation. It is important for people to know where they can be treated following exposure for effective rabies prevention. This result is consistent with what Edukugho et al. [14] reported. The latter observed that more than 86% of respondents would report to the hospital or see a doctor after being bitten by dogs. However, a report by Fesseha et al. [18], observed that only 55.6% of respondents would seek medical attention after a dog bite. This low number of people who would seek medical care reported by Fesseha et al. [18] in comparison to what was observed in the present study, suggests that the education of the public regarding how to treat exposed people is very impactful in the study area.

Although up to 83.33% of the respondents in this study knew that rabies can be transmitted from animals to humans, the number that did not know, even though small, cannot be ignored. However, these findings agree with those of studies done in Munesa district and Tigray regions of Ethiopia, in which 88.7% and 89.5%, respectively, indicated that humans were likely to contract rabies from animals [2,13].

The high number of respondents (79.04%) in the present study who were aware of the importance of obtaining anti-rabies vaccination following a bite from a suspected rabid dog, contrasts with findings from Odisha, India [41] and Ethiopia [11], which reported low numbers of respondents (14% and 38.8% respectively) who were aware of the importance of receiving anti-rabies vaccination after being bitten by a dog. This could be attributed to differences in the proximity to health care facilities in the different study areas. In instances where health facilities are readily accessed, although some

of the bite victims may go to a health facility with the intention of getting anti-rabies, they also end up receiving wound treatment. This could contribute to the high numbers seeking medical care observed in some studies as opposed to others.

The 71.74% of the respondents in the present study that were aware that if vaccinated against rabies following a bite by a rabid dog, a person is protected from developing rabies, is lower than the 87% of respondents from a study conducted in Abuja, Nigeria, that had visited health care facilities to obtain treatment following a dog bite [14]. Nonetheless, the high numbers of people in this study, who were aware that receiving the anti-rabies vaccine is important in preventing the disease in humans, further points to the effectiveness of the education campaign in the study area. This contrasts with finds of another study conducted in the Tigray region of Ethiopia, in which just over half (54.9%) of respondents preferred to go to traditional healers instead of hospitals following a dog bite incident [13].

It was encouraging to observe that majority of the respondents (74.61%) in this study were aware that a change in behaviour including dogs biting people were major clinical sign of rabies. This is in line with the findings reported in Dedo district, Southwest Ethiopia [1], Gondor Zuria, Ethiopia [11] and Gondar, Northwest [6], where up to 50.4%, 63.5% and 75.8% of the respondents respectively, knew that behaviour change is one of the signs of rabies.

On the other hand, paralysis as a clinical sign of rabies did not feature predominantly. Therefore, paralysis was likely to be overlooked as a sign of rabies by the study population. This is worth noting because, when such people get bitten by a rabid dog even if it showed paralytic signs, they are likely not to seek post exposure prophylaxis.

5.3 Attitude and practices of community members towards rabies

The considerable proportion of respondents (81.77%) in this study who showed a willingness to receive anti-rabies vaccine to help prevent the disease in case of dog bite, is a positive finding. This implies that people are willing to obtain anti-rabies treatment as soon as possible after exposure to prevent rabies, given that currently there is no available treatment once the disease has developed. The number of people willing to received anti-rabies in the current study was higher than 51% from Berhampur, Odisha, in India, that reported were willing to obtain anti-rabies as a control measure rabies following a dog bite, a country that is also endemic for rabies, and accounts for 36% of the world's rabies deaths [41]. An even lower percentage (38.8%) of people from Gondar Zuria District, Ethiopia, were willing to obtain anti-rabies in response to dog bite [11]. Just like India and South Africa, Ethiopia is endemic to rabies. The difference observed with respect to the number of people with a positive attitude towards receiving anti-rabies vaccine in the different countries that are endemic to rabies, could be attributed to the nature of education programmes implemented in the different countries. It could also be attributed to the differences in the level of education in the different study areas.

The overwhelming majority of respondents (90.10%) in this study were willing to seek medical attention following exposure to a dog bite victim. This is consistent with results of a survey done in Kombolcha, Ethiopia where 89.3% of respondents would seek medical care if they were bitten by an animal suspected to have rabies [20]. However, results from the present study widely contrasted with findings of a survey conducted in and around South Gondar, Ethiopia, in which it was observed that 79.4% of respondents were not in favor of visiting health care centers following dog bites [6].

The 52.47% of respondents who indicated that they would allow their dogs to be quarantined for observation after biting someone, contrasts with 69% of the respondents in a study from Kigali City, Rwanda, who were not in favor of isolating their animals if they bit people [31]. However, the number of people willing to have their dogs quarantined in the present study were far higher than those reported in a study conducted in Cameroon, where only 2.6% favored confining their dogs for observation when they start behaving unusually, including biting people [9].

While a low percentage of respondents (39.45%) in the present study that indicated that they would put down their dogs if they were suspected to be rabid, many more, either did not think that putting down the dog suspected of rabies was appropriate or did not know what to do with such animals (60,29%. This sharply contrasts with results of studies conducted in South Gondar and Kombolcha where 64.3% and 84.1%, respectively, indicated that they would kill the dog suspected to have rabies [6,20]. The low numbers of people willing to have their animals put down if they are suspected to be rabid in this study, could be influenced by the belief that rabies can be treated. It could also be influenced by religious belief among certain ethnic or religious groups that an animal should die naturally without human interference.

The sterilization rate in the study area was low with just over half (53.39%) of the respondents in the study population having not sterilised their pets. The low sterilization rate of pets suggests that many dogs in the study area can breed. The problem with this, is the potential to increase the risk of rabies due to the resultant high number of dogs in the study area. The larger the population of dogs in the area, the more dogs are likely to roam freely, especially during mating season when they want to reproduce. This is why, dog population control is likely to curtail the spread of rabies.

The number of people willing to sterilise their pets in the present study contrasts with the numbers reported by a study conducted in Sri Lanka by Ubeyratne et al. [43], who reported that up to 86.41% had not sterilised their pets. But findings of the present study are consistent with those of a study conducted in El Jadida, Morocco, that reported that just under half (45%) of respondents did not believe in the practice of sterilizing pets [7].

A copy of the rabies vaccination certificate was requested from respondents as a confirmation of pets in the study areas having been vaccinated. A vaccination certificate was recorded as valid if the pet had received the rabies vaccine the previous year or in the same year the study was conducted. Only 58.77% of the respondents in the present study were in position of a vaccination certificate as proof of vaccination of their pets. This finding is suggests that the vaccination coverage in the study area is below the 70% vaccination coverage recommended by the World Health Organization (WHO) [7] to prevent rabies outbreaks in humans. This finding is a serious public health concern because it shows that the area is susceptible to experiencing a rabies outbreak. However, findings reported here, contrast with reports of studies conducted elsewhere. For example, in the El Jadida Region, Morocco [7], it was observed that 76.6% of the respondents had rabies vaccination certificate for their pets. Ntampaka et al. [31], observed that the majority (81.8%) of respondents in their study had vaccinated their animals to prevent them from getting rabies and 81.2% of the respondents from Southern Wollo, Ethiopia indicated that vaccination was significant to maintain a society free from rabies [20].

However, a low vaccination coverage, has also been observed in other parts of the continent. For example, Costa et al. [9], reported a low vaccination coverage (30.8%) of pets in their study conducted in Cameroon. It is evident that the study area

is doing better than other areas parts of other countries. But that notwithstanding, there is room for improvement in the study area to ensure that the vaccination coverage is at the level recommended by the WHO.

The 75.91% of respondents in this study that showed a willingness to vaccinate their pets following guidelines provided by the State to vaccinate dogs, is higher than the findings reported in El Jadida, Morocco, where only 67.8% of the respondents appreciated annual dog vaccination [7]. An even a lower response was observed among respondents in Northwest Ethiopia, where only 28.1% knew that dogs should be vaccinated repeatedly to prevent rabies [6]. Adhering to repeated and regular rabies vaccination helps boost the immune level of pets and thus minimize outbreaks of rabies.

The 50.26% in this study who mentioned that to avoid getting rabies from animals, one must avoid any contact with stray dogs, sharply contrasts with the 81.77% who advocated for animals getting vaccinated. The 50.26% in the present study also contrasts with only 33.3% of respondents in Kansas who expressed the view that avoiding contact with animals one is not familiar with, is key to preventing rabies [37].

Over half (58.07%) of respondents in the present study, kept their dogs behind a fence, which is far less than the number of respondents (84%) in Shirsuphal who kept their dogs in a fenced place to limit them from going outside the households [39]. On the other hand, a lower percentage of respondents (25%) in Filipinos kept their animals in an enclosed environment to prevent them from roaming freely [10]. Results reported here contrast with those in Bobo Dioulasso, Burkina Faso, where up to 85% of the participants kept their animals in closed places to prevent them from roaming in the streets [35]. Keeping dogs from contact with other stray animals limits the chance

of rabies infection. Therefore, the lack of fences around homes with dogs increases the contact of animals which may lead to the rapid spread of rabies. Thus, confinement of dogs is very important and needs to be improved on in the study area.

Over one quarter (29.56%) of respondents in the present study indicated that they occasionally allowed their dogs to move freely out of the yard unsupervised. However, this figure is lower than what was reported by Costa et al. [9] and Corfmat et al. [8], who reported a significantly higher percentage of respondents (61% and 59%, respectively), who indicated that they occasionally allowed their dogs to move out of the yard freely. Dogs that move freely risk contracting the rabies virus from other infected animals.

Just as was observed that 64% of the respondents in the Filipinos study consistently vaccinated their dogs to prevent rabies [10], up to 64.32% of respondents in the current study regularly presented their dogs for vaccination during vaccination campaigns. Similarly, a study conducted in south Gondar also reported that 69.8% of respondents showed commitment to dog vaccination [6]. However, although the number of people who consistently vaccinated their dogs in the present study was high, it was below the recommended 70% vaccination coverage by WHO [7]. The low number could be because some people are not available during the campaigns due to work or other commitments, while others are unable to handle their dogs and end up not presenting their dogs for vaccination. It is also possible that the high number of dogs that are not confined that was observed in this study explains the low numbers of people who consistently present their dogs for vaccination. This is because such dogs are likely not to be available to be immunized during the time of the vaccination campaign.

It was encouraging to note that 62.50% of respondents in the present study indicated that dog vaccination is necessary even for dogs that are kept in closed yards all the time. This finding has potential to boost vaccination as a tool to drive elimination off dog-mediated human rabies.

A very lower response (17.84%) was noted with respect to submitting the carcass of suspected animals to the laboratory to confirm if it had rabies. The results of the study conducted in the Democratic Republic of the Congo about submitting carcasses for confirmation of rabies, was worse, with only 2% of respondents indicating that they would take the carcass of a suspected rabid animal to veterinary services for laboratory testing [27]. Effort is needed to increase awareness of the need to submit carcasses of animals that have died for laboratory confirmation. This has the potential to assist in early outbreak detection and improving surveillance efforts.

A mere 53.52% of the respondents in the present study who would report animals suspected of being rabies positive to the authorities, contrasts with a higher percentage (73%) of respondents from Shirsuphal village in western India, who would report the presence of rabid dogs to the municipal authorities if they ever encountered a case of rabies [40]. However, in Debark Worenda, Ethiopia 84.1% of the respondents said that they would kill the animal if they suspected it had rabies [45]. In the study by Mbilo et al [27], only 20% of respondents indicated that they would alert the veterinary services if they recognized a suspected rabid animal. This completely contrasts with the findings of the present study, and this difference could be attributed to the impact of the rabies eradication campaigns in the different areas. Behavioral change in a suspected rabid animal was reported by a higher number of respondents, while other signs of rabies were mentioned less. These findings are of public health importance in that respondents are likely to report to the authorities when they observe

behavior change, which is an important sign of rabies. However, studies show that aggressive animals are likely to become tame when rabid. As a result, it might be difficult to identify when the animal is a stray.

5.4 Factors significantly associated with knowledge

The finding that respondents who owned pets had significantly higher odds (AOR: 2.788; P=0.0001) of being knowledgeable about rabies in comparison to respondents who did not own pets, is consistent with the findings by Abdela et al. [1]. The later also observed that respondents who owned dogs had significantly (P=0.029) higher odds of obtaining a good KAP score than those who did not own dogs. Similar results were observed among residents of Abuja municipal area, where it was found that people who owned dogs were 7.8 times more likely to have a better knowledge of rabies than those who had no dogs [14]. This is explained by the fact that people who own pets are more likely to receive information during rabies campaigns when they take their dogs for vaccination.

6. CONCLUSION AND RECOMMENDATIONS

There are knowledge gaps with respect to certain aspects such as rabies being a viral disease, how one can protect oneself from contracting rabies, precautionary measures to adopt following exposure to rabies and how to protect susceptible animals from being exposed to rabies. The role of measures such erecting fences around homesteads to restrict animal movements and neutering of pets to control dog population need to be emphasized during extension. The low number of people who had vaccination certificates, although worth noting, needs further investigate to establish whether respondents do not keep certificates, or if it is a true reflection of the status of the vaccination coverage.

The study identified that dog ownership was the only socio-demographic factor that was a significant predictor of knowledge on rabies. In view of this, information dissemination needs to be inclusive to include non-pet owners in the area who also constitute the population at risk. On a positive side, the study demonstrated that there was widespread knowledge on some aspects like knowledge of the role of dog bites in the spreading of rabies in the study area, which suggests that people are likely to seek medical attention in case of a dog bite incidence. In addition, a good attitude towards receiving PEP following a dog bite and reporting a suspected cases were widespread in the study.

To bridge the gaps in knowledge that were identified in this study, implementation of global strategies such as awareness and vaccination campaigns need to be stepped up. Information to sensitize people can be found from government distributed pamphlets or by watching “If Only I Knew” video and by also consulting Government Veterinary Services (GVS). In addition, programs to enforce the positive aspects identified in this study should continuously be implemented. This will help improve the control and prevention efforts directed towards minimizing and eradicating rabies outbreaks including associated human fatalities. A one health approach that involves collaboration between veterinary services with other public health institutions and local authorities to expand and strengthen rabies awareness should be adopted in the study areas to achieve 2030 goal of rabies elimination in rabies endemic areas.

7. Limitations

This study was subject to limitations of cross-sectional surveys such as recall biases. Furthermore, the study was limited to Verulam and Embo in eThekwini District; hence, the results cannot be generalised to other areas in South Africa. The study was conducted during the lockdown due to the COVID-19 pandemic, when movement of

people was restricted and people were reluctant to meet strangers, which could have led to selection bias, which thus calls for caution when interpreting the results. Nonetheless, this study presents baseline information on the knowledge, attitude, and practices towards rabies, which was previously unavailable in the study area. It also provides baseline information upon which future research can be developed.

8. ACKNOWLEGEMENTS

The authors would like to appreciate the University of South Africa for partially funding this study through the Post-graduate Bursary, as well as the participants for consenting to participate in this study, and their cooperation throughout the study. We would like to acknowledge the Department of Agriculture and Rural Development, Veterinary services section, KZN, for permitting us to carry-out the study within eThekwini district. We would also like to extend gratitude to UNISA ethics committee for approving this study to be conducted.

REFERENCES

1. Abdela, N., Midekso, B., Jabir, J. and Abdela, W. (2017) ‘Knowledge, attitudes and practices towards rabies in Dedo district of Jimma zone, southwestern Ethiopia: A community based cross - sectional study’, 9(May), pp. 61–71. Available at: https://doi.org/10.5897/IJMMS2017.1302.
2. Abdela, N. and Teshome, E. (2017) ‘Community based cross-sectional study on knowledge, attitudes and practices towards rabies in Munesa District, Arsi Zone, Southeastern Ethiopia’, *Journal of Public Health and Epidemiology*, 9(6), pp. 161–170. Available at: https://doi.org/10.5897/jphe2017.0931.
3. Ali, A., Ahmed, E.Y. and Sifer, D. (2014) ‘A Study on Knowledge, Attitude and Practice of rabies among residents in Addis Ababa, Ethiopia’, *Ethiopian*

Veterinary Journal, 17(2), p. 19. Available at: https://doi.org/10.4314/evj.v17i2.2.

4. Ba, M.F., Kane, N.M., Diallo, M.K.K., Bassoum, O., Boh, O.K., Mboup, F.Z.M. *et al.* (2021) 'Knowledge, attitudes and practices on rabies among human and animal health professionals in senegal', *Pathogens*, 10(10), pp. 1–15. Available at: https://doi.org/10.3390/pathogens10101282.
5. Barroga, T.R.M., Basitan, I.S., Lobete, T.M., Bernales, R.P., Gordoncillo, M.J.N., Lopez, E.L. *et al.* (2018) 'Community Awareness on Rabies Prevention and Control in Bicol, Philippines: Pre- and Post-Project Implementation', *Tropical Medicine and Infectious Disease*, 3(1), pp. 1–14. Available at: https://doi.org/10.3390/tropicalmed3010016
6. Bihon, A., Meresa, D. and Tesfaw, A. (2020) 'Rabies: Knowledge, Attitude and Practices in and Around South Gondar, North West Ethiopia', *Diseases*, 8(1), p. 5. Available at: https://doi.org/10.3390/diseases8010005
7. Bouaddi, K., Bitar, A., Bouslikhane, M., Ferssiwi, A., Fitani, A. and Mshelbwala, P.P. (2020) 'Knowledge, attitudes, and practices regarding rabies in El jadida region, Morocco', *Veterinary Sciences*, 7(1). Available at: https://doi.org/10.3390/vetsci7010029
8. Corfmat, J. Gibson, A.D., Mellanby, R.J, Watson, W., Appupillai, M., Yale, G. *et al.* (2022) 'Community attitudes and perceptions towards free-roaming dogs in Goa, India', *Journal of Applied Animal Welfare Science*, 00(00), pp. 1–17. Available at: https://doi.org/10.1080/10888705.2021.2014839.
9. Costa, G.B., Gilbert, A., Monroe, B., Blanton, J., Ngam, S.N., Recuenco, S. *et al.* (2018) 'The influence of poverty and rabies knowledge on healthcare

seeking behaviors and dog ownership, Cameroon', *PLoS ONE*, 13(6). Available at: https://doi.org/10.1371/journal.pone.0197330.

10. Davlin, S.L. Lapiz, S.M., Miranda, M.E. and Murray, K.O. (2014) 'Knowledge, attitudes, and practices regarding rabies in Filipinos following implementation of the Bohol Rabies Prevention and Elimination Programme', *Epidemiology and Infection*, 142(7), pp. 1476–1485. Available at: https://doi.org/10.1017/S0950268813002513.
11. Digafe, R.T., Kifelew, L.G. and Mechesso, A.F. (2015) 'Knowledge, attitudes and practices towards rabies: Questionnaire survey in rural household heads of Gondar Zuria District, Ethiopia', *BMC Research Notes*, 8(1). Available at: https://doi.org/10.1186/s13104-015-1357-8.
12. Dubey, S., Singh, R.V., Gupta, B., Gupta, D.K., Nyak, A., Rai, A. *et al.* (2022) 'Study on rabies control and prevention using knowledge attitude and practice in Jabalpur central India', 11(7), pp. 2353–2359.
13. Ebuy, Y., Alemayehu, T., Reda, M., Berhe, M. and Bsrat, A. (2019) 'Community Knowledge, Attitude and Practice on Rabies, Incidence in Humans and Animals and Risk Factors to Rabies in Selected Districts of Tigray Region, Ethiopia', *Nigerian Veterinary Journal*, 40(2), pp. 147–163.
14. Edukugho, A.A., Umoh, J.U., Diem, M., Ajani, O., Uba, B., Okeke, L. *et al.* (2018) 'Knowledge, attitudes and practices towards rabies prevention among residents of Abuja municipal area council, Federal Capital Territory, Nigeria', *Pan African Medical Journal*, 31, pp. 1–11. Available at: https://doi.org/10.11604/pamj.2018.31.21.15120.
15. eThekwini Municipality (2017) Integrated Development Plan (IDP) Annual Review - *2017/2018*; Durban eThekwini Municipality; 2017.

16. Fazeli, M., Golahdouz, M., Bashar, R., Bafrrani, M.A., Doosti-Irani, A., Pourhossein, B. *et al.* (2018) ‘Design and Validation of a Persian Knowledge, Attitude, and Practice Questionnaire for Rabies (PKAP-Rabies) in the General Population of Iran’, *Journal of Medical Microbiology and Infectious Diseases*, 6(2), pp. 67–71. Available at: https://doi.org/10.29252/jommid.6.2.3.67
17. Fenelon, N., Dely, P., Katz, M.A., Schaad, N.D., Dismer, A., Moran, D. *et al.* (2017) ‘Knowledge, attitudes and practices regarding rabies risk in community members and healthcare professionals: Pétionville, Haiti, 2013’, *Epidemiology and Infection*, 145(8), pp. 1624–1634. Available at: https://doi.org/10.1017/S0950268816003125
18. Fesseha, H. and Hadgu, A. (2020) ‘Assessment of Knowledge, Attitude and Practices on Rabies among Communities of Atsbi Wonberta District, Tigray, Northern Ethiopia’, *Current Research in Agriculture and Farming*, 1(1), pp. 21–31. Available at: https://doi.org/10.18782/2582-7146.104
19. Gebeyaw, S. and Teshome, D. (2016) ‘Study on Community Knowledge, Attitude and Practice of Rabies in and Around Dessie City’, *Austin Journal of Veterinary Science & Animimal Husbandry*, 3(1), p. 5.
20. Gebremeskel, K.A., Tanga, B.M., Getachew, A. and Eshetu, Y. (2019) ‘Assessment of public knowledge, attitude and practices towards rabies in the community of Kombolcha, Southern Wollo, Amhara Reginal State, Ethiopia’, *Journal of Public Health and Epidemiology*, 11(1), pp. 38–48. Available at: https://doi.org/10.5897/jphe2017.0965
21. Guadu, T., Shite, A., Chanie, M., Bogale, B. and Fentahun, T. (2014) ‘Assessment of Knowledge, Attitude and Practices about Rabies and

Associated Factors: In the Case of Bahir Dar Town', *Global Veterinaria*, 13(3), pp. 348–354. Available at: https://doi.org/10.5829/idosi.gv.2014.13.03.8579.

22. Hampson, K., Coudeville, L., Lembo, T., Sambo, M., Kieffer, A., Attlan, M. *et al*. (2015) 'Estimating the Global Burden of Endemic Canine Rabies', *Plos Neglected Tropical Diseases,* 9(4): e0003709. Available at: https://doi.org/10.1371/journal.pntd.0003709
23. Hergert, M., LeRoux, K. and Nel, L.H. (2016) 'Risk factors associated with nonvaccination rabies status of dogs in KwaZulu-Natal, South Africa', *Veterinary Medicine: Research and Reports*, Volume 7, pp. 75–83. Available at: https://doi.org/10.2147/vmrr.s103859
24. Hergert, M. and Nel, L.H. (2013) 'Dog Bite Histories and Response to Incidents in Canine Rabies-Enzootic KwaZulu-Natal, South Africa', *PLoS Neglected Tropical Diseases*, 7(4), pp. 1–11. Available at: https://doi.org/10.1371/journal.pntd.0002059
25. Kiratitana-olan, K., Chaisowwong, W., Thongkorn, K. and Kreausukon, K. (2021) 'Keys to Good Knowledge, Attitude and Practice for Rabies Prevention in Disease-Free Communities in Thailand', *Veterinary Intergrative Sciences,* 19(3): 407-422
26. Lankester, F.J., Wouters, P.A.W.M., Czupryna, A., Palmer, G.H., Mzimbiri, I., Cleaveland, S. *et al.* (2016) 'Thermotolerance of an inactivated rabies vaccine for dogs', *Vaccine*, 34(46), pp. 5504–5511. Available at: https://doi.org/10.1016/j.vaccine.2016.10.015
27. Mbilo, C., Kabongo, J., Pyana, P.P., Nlonda, L., Nzita, R.W., Luntadila, B. *et al.* (2019) 'Dog ecology, bite incidence, and disease awareness: A cross-sectional survey among a rabies-affected community in the democratic

republic of the congo’, *Vaccines*, 7(3). Available at: https://doi.org/10.3390/vaccines7030098

28. Morters, M.K., McNabb, S., Horton, D.L., Fooks, A.R., Schoeman, J.P., Whay, H.R. *et al.* (2015) ‘Effective vaccination against rabies in puppies in rabies endemic regions’, *Veterinary Record*, 177(6), p. 150. Available at: https://doi.org/10.1136/vr.102975
29. Ngah Osoe Bouli, F.P., Awah-Ndukum, J., Tejiokem, C.M. and Mingoas Kilekoung, J. (2018) ‘Knowledge, attitudes and practices (KAP) evaluation regarding rabies in the Centre, Adamawa and North Regions of Cameroon’, *Journal of Animal Science and Veterinary Medicine*, 3(6), pp. 178–183. Available at: https://doi.org/10.31248/jasvm2018.114
30. Ngugi, J.N., Maza, A.K., Omolo, O.J. and Obonyo, M. (2018) ‘Epidemiology and surveillance of human animal-bite injuries and rabies post-exposure prophylaxis, in selected counties in Kenya, 2011-2016’, *BMC Public Health*, 18(1). Available at: https://doi.org/10.1186/s12889-018-5888-5
31. Ntampaka, P., Nyaga, P.N., Niragire, F., Gathumbi, J.F. and Tukei, M. (2019) ‘Knowledge, attitudes and practices regarding rabies and its control among dog owners in Kigali city, Rwanda’, pp. 1–16. Available at: https://doi.org/10.1371/journal.pone.0210044
32. Pal, P., Yawongsa, A., Bhusal, T.N., Bashyal, R. and Rukkwamsuk, T. (2021) ‘Knowledge, attitude, and practice about rabies prevention and control: A community survey in Nepal’, *Veterinary World*, 14(4), pp. 923–942. Available at: https://doi.org/10.14202/vetworld.2021.933-942
33. Rine, R.C., Dogara, G.S. and Pam, M.D. (2017) ‘Knowledge, Attitude and Practice of Rabies in and Around Lafia Metropolis, Nigeria’, *Central African*

Journal of Public Health, 3(3), pp 27-33. Available at: https://doi.org/10.11648/j.cajph.20210303.11

34. Sabeta, C.T., Weyer, J., Geertsma, P., Mohale, D., Miyen, J., Blumberg, L.H. *et al.* (2013) 'Emergence of rabies in the Gauteng Province, South Africa: 2010-2011', *Journal of the South African Veterinary Association*, 84(1), pp. 2010–2011. Available at: https://doi.org/10.4102/jsava.v84i1.923
35. Savadogo, M., Tialla, D., Ouattara, B., Dahourou, L.D., Ossebi, W., Ilboudo, S.G. *et al.* (2021) 'Factors associated with owned-dogs' vaccination against rabies: A household survey in Bobo Dioulasso, Burkina Faso', *Veterinary Medicine and Science*, 7(4), pp. 1096–1106. Available at: https://doi.org/10.1002/vms3.468
36. Spargo, R.M., Coetzer, A., Makuvadze, F.T., Chikerema, S.M., Chiwerere, V., Bhara, E. *et al*. (2021) 'Knowledge, attitudes and practices towards rabies: A survey of the general population residing in the Harare Metropolitan Province of Zimbabwe' *PLos ONE* 16(1): e0246103. Available at: https://doi.org/10.1371/journal.pone.0246103
37. Straily, A. and Trevino-Garrison, I. (2017) 'Knowledge, Attitudes and Practices of Law Enforcement Officers on Rabies and Animal Control Issues in Kansas', pp. 111–117. Available at: https://doi.org/10.1111/zph.12287
38. Tandon, S., Kotwal, S.K., Malik, M.A., Singh, M., Kumar, D., Shafiq, M. *et al.* (2017) 'A Community Based Survey on Rabies Control and Prevention using KAP in', (December), pp. 1019–1028. Available at: https://doi.org/10.5958/2277-940X.2017.00153.X
39. Tiwari, H.K., Robertson, I.D., O'Dea, M. and Vanak, A.T. (2019) 'Knowledge, attitudes and practices (KAP) towards rabies and free roaming dogs (FRD) in

Panchkula district of north India: A cross sectional study of urban residents', *PLoS Neglected Tropical Diseases*, 13(4), pp. 1–17. Available at: https://doi.org/10.1371/journal.pntd.0007384

40. Tiwari, H.K., O'Dea, M., Robertson, I.D., and Vanak, A.T. (2019) 'Knowledge, attitudes and practices (KAP) towards rabies and free-roaming dogs (FRD) in Shirsuphal village in western India: A community based cross-sectional study', *PLoS Neglected Tropical Diseases*, 13(1). Available at: https://doi.org/10.1371/journal.pntd.0007120

41. Tripathy, R.M., Satapathy, S.P. and Karmee, N. (2017) 'Assessment of knowledge, attitude and practice regarding rabies and its prevention among construction workers: a cross-sectional study in Berhampur, Odisha', *International Journal of Research in Medical Sciences*, 5(9), p. 3971. Available at: https://doi.org/10.18203/2320-6012.ijrms20173964

42. Tschopp, R., Bekele, S. and Aseffa, A. (2016) 'Dog Demography, Animal Bite Management and Rabies Knowledge-Attitude and Practices in the Awash Basin, Eastern Ethiopia', *PLoS Neglected Tropical Diseases*, 10(2). Available at: https://doi.org/10.1371/journal.pntd.0004471

43. Ubeyratne, K., Srikitjakarn, L., Pfeiffer, D., Kong, F., Sunil-Chandra, N., Chaisowwong, W. *et al.* (2020) 'A Knowledge, Attitudes and Practices (KAP) survey on canine rabies prevention and control in four rural areas of Sri Lanka', *Transboundary and Emerging Diseases*, (November), pp. 1–15. Available at: https://doi.org/10.1111/tbed.13940

44. Weyer, J., Dermaux-Msimang, V., Grobbelaar, A., LeRoux, C., Moola, N., Paweska, J. *et al.* (2020) 'Epidemiology of human rabies in South Africa, 2008

- 2018’, *South African Medical Journal*, 110(9), pp. 877–881. Available at: https://doi.org/10.7196/SAMJ.2020.v110i9.14324

45. Yalemebrat, N., Bekele, T. and Melaku, M. (2016) ‘Journal of Veterinary Medicine and Animal Health Assessment of public knowledge, attitude and practices towards rabies in Debark Woreda, North Gondar, Ethiopia’, *Journal of Veterinary Medicine and Animal Health*, 8(11), pp. 183–192. Available at: https://doi.org/10.5897/JVMAH2016.0504

Supporting information

S1 Fig. Fig 1: Distribution of respondents by responses to questions regarding animals susceptible to rabies.

S2 Fig. Fig 2: The percentage of respondents based on how they responded to the question about the clinical signs of rabies in dogs.

S3 Fig. Fig 3: A bar graph showing the proportion of respondents based on their attitude towards rabies in eThekwini District

Click here to access/download;Figure;Fig 2.docx

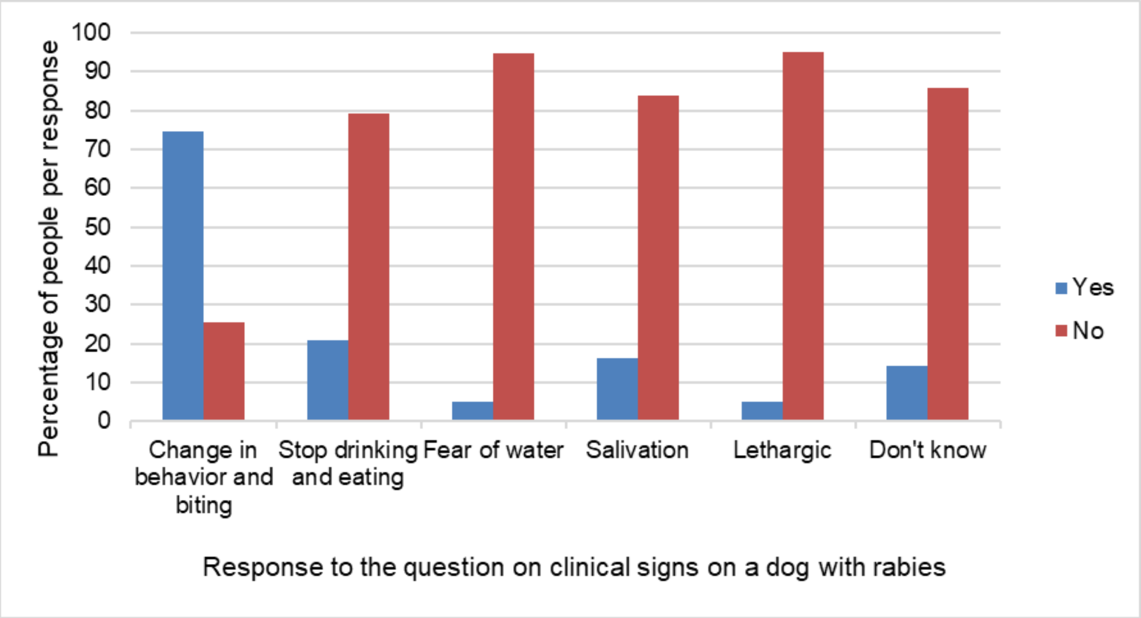

Figure 3 Click here to access/download;Figure;Fig 3.docx

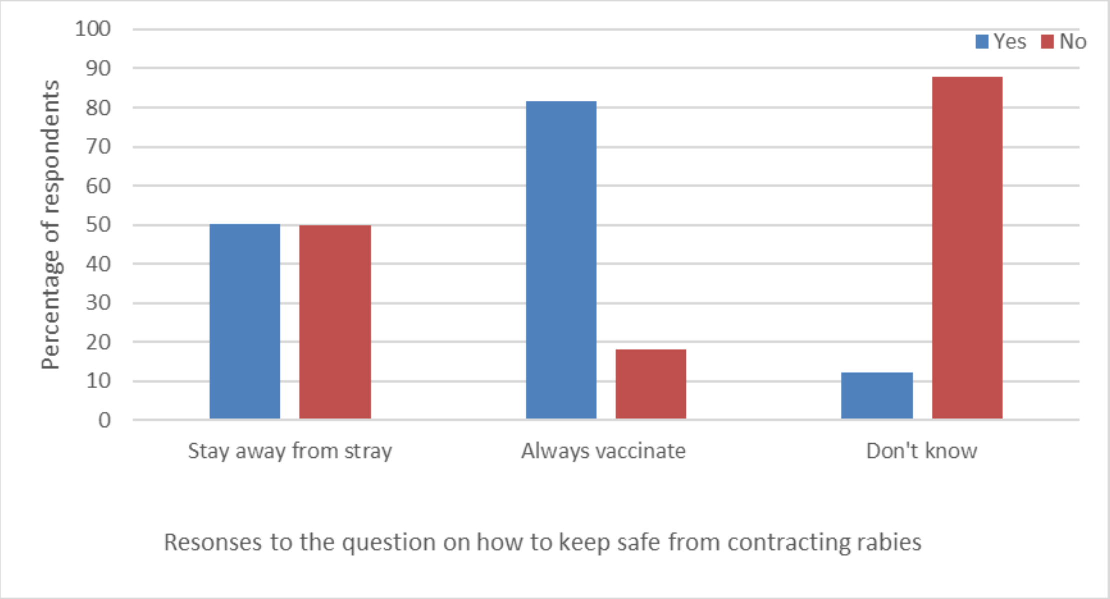

Figure 1

Click here to access/download;Figure;Fig 1.docx

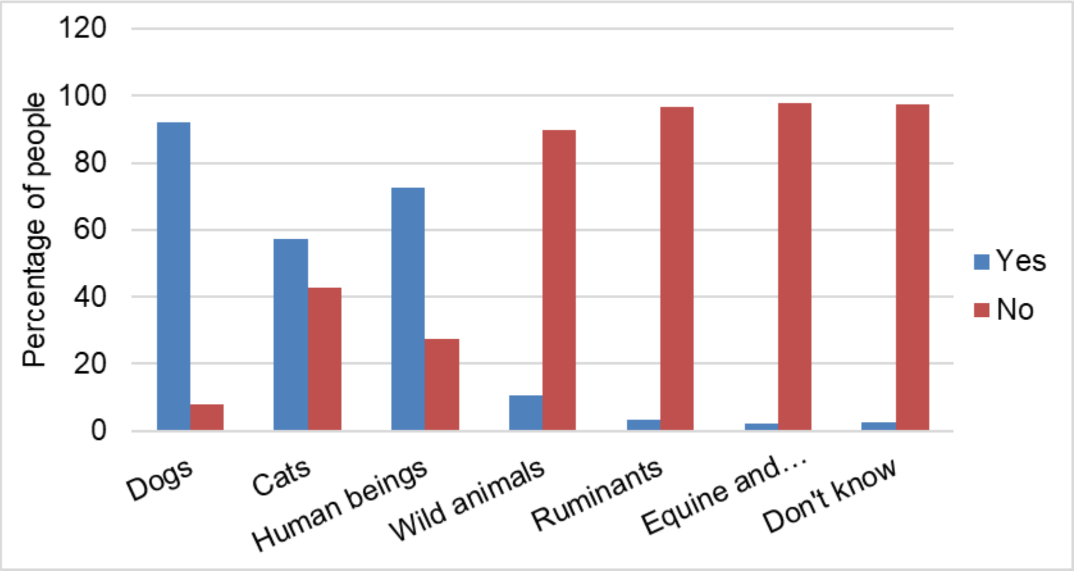

Click here to access/download
Supporting Information
Ref no 2019_CAES_HREC_105.pdf

***Knowledge, attitude, and practices towards rabies: a community survey in selected areas of KwaZulu-Natal Province, South Africa*.**

Mohube Titus Letsoalo[1,2¶], Daniel Nenene Qekwana[3&], Christian. A. Mbajiorgu[1&], Ishmael Festus Jaja[1&], James Wabwire Oguttu[1¶]*

[1]Department of Agriculture and Animal Health, College of Agriculture and Environmental Sciences, University of South Africa, Johannesburg, South Africa

[2]KwaZulu-Natal Department of Agriculture and Rural Development, Durban, KZN, South Africa

[3]Department of Paraclinical Science, Faculty of Veterinary Science, University of Pretoria, Pretoria, South Africa.

*Correspondence

Email: joguttu@unisa.ac.za

Abstract

Knowledge, attitude, and practices (KAP) studies have been widely used to assess gaps towards rabies. However, there is no evidence of baseline studies that have assessed the KAP towards rabies among selected communities in KwaZulu-Natal Province, South Africa. A ~~prospective~~ cross-sectional questionnaire-based study design was adopted for this study. Participants (n=768) were selected using systematic random sampling. Data from respondents (≥18 years) was collected using a pretested questionnaire and analysed using descriptive and inferential statistics. Significance was set at α≤0.05. Twenty seven percent (26.95%) of the respondents ~~of the respondents~~ could name the cause of rabies, while just over half (53.52%) of the respondents indicated that they would adhere to post exposure prophylaxis (PEP) ~~treatment~~ which, ~~included~~ includes wound washing, a dose of human rabies immune globulin (HRIG), and rabies vaccine given at the time of the first medical visit, and two booster doses ~~washing the wound with soap and running water~~. Most respondents

(90.10%) mentioned that they would seek medical attention following a dog bite. Only 59.77% of respondents were able to produce a vaccination certificate for their pets. Allowing dogs to roam without supervision was widely reported by the respondents. Dog ownership was the only factor significantly associated with knowledge of rabies. Lack of knowledge of the cause of rabies and the need to receive PEP immediately after exposure to a dog bite was widespread in the study area. However, gaps in some aspects related to knowledge about rabies were observed that require awareness creation for dog owners, including those who do not own pets.

Keywords: Rabies, vaccination, rabies disease, rabies awareness, dog-bite, post-exposure prophylaxis, PEP, Risk factors

1. INTRODUCTION

Domestic dogs play a significant role in the lives of humans. But on the other hand, they pose the greatest threat of transmitting rabies to humans [9]. Rabies is an important infectious viral disease [4], caused by the lyssavirus, from the family of *Rhabdoviridae* [16, 20]. All warm-blooded mammals are susceptible to the rabies virus infection generally transmitted through the bite of a rabid animal [29].

Dog mediated rabies infection remains a significant disease from a public health perspective [33] and annually it is responsible for an estimated 60 000 human deaths worldwide [22, 28]. Of these, Asia has the highest burden of rabies, followed by Africa. Making the two the most affected continents. In Africa, over 90% of human rabies deaths are associated with dog bites [33, 42]. In Asia alone, approximately 31,539 human deaths are reported annually suggesting a high burden of rabies in the continent ([3]. Furthermore, globally, around 15 million people seek rabies post-exposure prophylaxis (PEP) due to the threat of rabies infection following exposure [30].

Once symptoms of rabies develop, victims usually succumb to the disease [2]. This is especially true if the victim fails to obtain PEP early following a dog bite [14]. In humans potentially exposed to the rabies virus, PEP usually includes thorough wound cleaning and a combination of treatment with rabies immunoglobulin and vaccination [44]. Without proper health care, the loss of life of untreated victims is inevitable. Moreover, the disease is reported to have the highest case fatality ~~rate~~ of any disease [33].

Rabies is a preventable disease, and vaccination of dogs through mass campaigns is one of the effective ways to control the disease at its source [22]. According to the World Health Organization (WHO), a minimum of 70% dog vaccination coverage can drastically reduce the number of human cases within countries where rabies is endemic [26]. However, in most developing countries, rabies continues to be a challenge resulting in high mortalities in humans and animals [25]. This is despite existence of effective human and animal rabies vaccines [33]. This could be attributed to inadequate efforts to control and prevent the spread of the disease in these countries [14].

In South Africa, an estimated 40 human rabies related deaths from dog bite contacts occur yearly [44]. This points to the fact that measures needed to successfully prevent and control the spread of rabies within the dog population are inadequate. This is especially true in the province of KwaZulu-Natal (KZN), which has the highest cases of dog rabies in the country [23]. In addition, rabies is well established and remains endemic in the province [34]. This is supported by the recent increase in the number of laboratory confirmed positive animal cases in KZN [44]. Moreover, most of such cases of rabies have been confirmed in dogs [23]. Besides KZN Province, the Eastern Cape and Limpopo provinces are also considered by the Department of

Agriculture, Land Reform and Rural Development (DALLRD) as having high numbers of rabies.

Knowledge, attitude and practices studies are significant in obtaining information on the subject of concern [32]. Therefore, questions on the cause, source and mode of transmission, clinical signs and prevention practices and treatment measures are important in determining participants' knowledge, attitude and practices (KAP) regarding rabies [21]. In addition, knowledge of how to handle a suspected rabid dog is important [10], and measures that must be adopted when bitten by a rabid dog, need to be assessed if measures to prevent and control rabies are to be effective [45].

In fact, good knowledge, attitude and practices about rabies have been shown to reduce the spread of the disease and the fatalities associated with the disease [16]. In view of this, the present study investigated the KAP of communities in two areas (Embo and Verulam) of KwaZulu-Natal Province of South Africa.

2. METHODS

2.1 Study area

The study was conducted within eThekwini District in KwaZulu-Natal Province of South Africa. Kwazulu-Natal is one of the nine provinces in South Africa and is a multi-racial community. The population of KZN is approximately 10,819,130 living on an area of 94,361 km². Its annual population growth is estimated to be 1, 2 % [24].

The eThekwini District is occupied by about 3.7 million people on a land division of approximately 2,555 km² [15]. Embo (Latitude: 29° 54' 34'' S, Longitude: 30° 40' 29'' E) is a rural area situated in the inlands of eThekwini in the western region of the district, with an estimated population size of approximately 40,924 people, and an estimated 8,184 households. Verulam (Latitude: -29° 38' 59.99'' S; Longitude: 31° 02'

60.00" E) on the other hand is an urban area located in the upper part of the northern region [15]. Verulam has an estimated 64,950 households (Unpublished data, Verulam Environmental Health Manager), and approximately 184,114 occupants on a land of 18.13 km² ([15].

2.2 Study design

A cross-sectional study design was adopted to investigate the knowledge, attitude and practices of community members towards rabies in eThekwini District in KZN province of South Africa.

2.3 Target population

The target population for this study was the residents of Embo (a rural area) and Verulam (an urban area). The study included people of different age groups, religious beliefs, and educational backgrounds. People of both sex (male or female) and both pet owners and non-pet owners were also included in the study.

2.4 Sampling strategy

A systematic random sampling method was used to sample from the two selected areas (Embo and Verulam). In both areas, a sampling interval was predetermined before the selection of households commenced. The first household was selected randomly, thereafter, the successive households were selected following the adopted interval for each of the areas until the number required was reached. However, if an adult was absent at the time of interviewing, the next house with an adult (≥18 years) was chosen to be interviewed [40].

2.5 Sample size determination

This sample size from the two study areas (Embo and Verulam) was determined using the formula described by Gaudu et al. [21] :

$$N = \frac{1.96^2 \times Pexp(1 - Pexp)}{(d)^2}$$

N= Sample size

P_{exp}- Estimate or expected proportion of knowledge about rabies from community was considered at 50%, given that there is no baseline study regarding KAP in the study area.

d^2- Desired absolute precision (5%).

A total of 768 respondents (384 from each of the two areas) were recruited and agreed to voluntarily participate in the study.

2.6 Data collection

The questionnaire developed by Fenelon et al. [17] was adapted for this study. Data was collected using a structured questionnaire during face-to-face interviews. The questionnaire had the following sections: respondents' demographic data, Knowledge of rabies, Attitude towards rabies and Practices.

The questionnaire was translated into the local language (isiZulu) that is most widely spoken to cater for respondents who could not communicate in English. In addition, the questionnaire was pre-tested on fifteen participants from each area to test its fitness for purpose. Each interview session lasted 30-45 minutes and was conducted in English or IsiZulu. Administration of the questionnaire took place from 01/08/2019 to 31/03/2021.

2.7 Data management and data analysis

2.7.1 Data management

Each questionnaire was reviewed carefully after each data collection session to ensure that it was filled in correctly, and afterwards each question was coded. All raw data was immediately captured into the computer using Microsoft Excel 365

(Microsoft Corp., Redmond 137, USA). The correct response to each of the questions on knowledge was awarded a score of 1, while 0 was given for the incorrect answer. Thereafter, the total score for each respondent was computed and presented as a percentage (knowledge score). A binary variable was then created from the knowledge score (knowledgeable=1 and not knowledgeable=0).

2.7.2 Data analysis

Data was analysed using the Statistical Package for Social Science (SPSS) Version 27 (SPSS for Windows, version 27, SPSS, Inc, Chicago, Illinois, the USA). Descriptive statistics such as frequencies and percentages of all categorical variables were computed and presented in graphs and tables. Inferential analysis involved first fitting a series of univariate binary logistic regression models to test for simple associations between the predictor variables and the outcome variable (Knowledge). Variables with alpha level ≤ 0.20 in the ~~univariate~~ univariable model were included in the ~~multivariate~~ multivariable model. Significance in the multivariable model was assessed at $p > 0.05$.

A multivariable ~~binary~~ logistic regression model was then fit to the data to assess the factors that were significantly associated with being knowledgeable about rabies. Using the backward selection method, variables to retain in the final regression model were identified. If removal of a variable from the model resulted in a ≥20% change in the effects measure of any of the variables in the model, the variable was considered a confounder and was thus retained in the final effects model, regardless of whether it was significantly associated with the outcome variable or not.

Models were compared using the Log likelihood ratio test. The model fit of the final effects model was tested using a Hosmer-Lemeshow test. Based on the results, there was a good model fit ($p > 0.05$).

3. Ethical consideration

The State Veterinary services of KwaZulu-Natal Department of Agriculture and rural development granted approval to conduct the study. In addition, permission to carry out the study was granted by the Ethics committee of the College of Agriculture and Environmental Science, University of South Africa (REC Reference #: 2019/CAES_HREC/105). The identity of the respondents was protected by ensuring that they remained anonymous. The objectives of the study were first explained to the respondents, and thereafter each participant signed the consent form to indicate that they voluntarily accepted to participate in the study. All respondents had the right to withdraw from the study at any time during the study. Findings of this study will be disseminated to the residents of the study areas during rabies vaccination campaigns and presented at conferences to the broader scientific community and researchers.

4. RESULTS

4.1 Descriptive statistics

4.1.1 Demographic profile of respondents

An equal number of respondents (384) was drawn from each of the identified areas (Embo and Verulam). Therefore, a total of 768 community members were invited to participate in the study.

Majority of the respondents were females (63.54%), aged between 18 – 35 years (53.21%), belonged to the Christian faith (47.66%), and had not completed secondary education (35.81%). There was a high pet ownership (72.79%) in the study area (Table 1).

Table 1: **Demographic profile of people who agreed to participate in the study**

Variables	Level	Frequency (n)	Percentage %	95% Confidence Interval	
				Lower interval	Upper interval
Age	18 – 35	409	53.26	49.65	56.83
	36 -53	288	37.50	34.06	41.03
	54 - 71	64	8.33	6.48	10.52
	>71	7	0.91	0.37	1.87
Sex	Male	280	36.46	33.05	39.97
	Female	488	63.54	60.03	66.95
Residence	Verulam	384	50.00	46.40	53.60
	Embo	384	50.00	46.40	53.60
Own a pet dog and/ or cat	Yes	559	72.79	69.49	75.91
	No	209	27.21	24.09	30.51
Religion	Christian	366	47.66	44.07	51.26
	African religion	140	18.23	15.56	21.15
	Hindu	204	26.56	23.47	29.84
	Muslim	31	4.04	2.76	5.68
	Other	27	3.52	2.33	5.07
Education	No education	43	5.60	4.08	7.47
	Completed primary	101	13.15	10.84	15.75
	Secondary not completed	275	35.81	32.41	39.31
	Completed matric/High school Education	234	30.47	27.23	33.86
	Tertiary completed	115	14.97	12.52	17.70

4.1.2 Knowledge of rabies disease

Results of the assessment of knowledge of rabies are presented in Table 2 and Table 3. Half (50.52%; n= 388) of respondents indicated that they did not know the cause of rabies, with only 26.95% (n=207) of the participants indicating that rabies is caused by a virus. Although in the minority, it was interesting to note that some respondents (2.47%, n=19) thought that rabies is caused by a chemical substance.

Table 2: The results of the assessment of the knowledge of rabies among respondents

Variables	Level	Frequency (n)	Percentage %
What is the cause of rabies?	Chemical substances	19	2.47
	Virus	207	26.95
	Insufficient intake of feed and water	77	10.03
	Psychological problem	77	10.03
	Don’t know	388	50.52

How can human be infected with rabies?	Dog-bite	668	86.98
	Playing with the dog	15	1.95
	Feeding the dog	0	0
	Don't know	85	11.07
What is the first thing to be done after dog-bite while at home?	Cover the wound with bandage	96	12.50
	Cleanse the wound with soap and running water	411	53.52
	Apply any topical medication	134	17.45
	Don't know	127	16.54
Can rabies be treated beyond appearance of clinical signs?	Yes	387	50.39
	No	123	16.02
	Don't know	258	33.59
When is it appropriate to receive anti-rabies injection after being bitten by a dog suspected of having rabies?	Later after weeks	27	3.52
	Immediately	601	78.26
	Any time after months	41	5.34
	Don't know	99	12.89
Is vaccination of pets important for preventing human rabies?	Yes	611	79.56
	No	13	1.69
	Don't know	144	18.75

Dog-bite was identified by most respondents (86.98%, n=668) as the likely rout for the transmission of rabies. In response to the question on the first thing to be done after a dog-bite while at home, slightly over half of the respondents (53.52%, n=411) indicated that they would clean the wound with soap and running water (Table 2).

Although 50.39% (n=387) of respondents were not aware that rabies can be fatal beyond appearance of clinical signs. It was also noted that most respondents (78.26%, n=601) would immediately seek the provision of anti-rabies injections following a bite by a suspect rabid animal. However, 12.89% (n=99) did not know that they should get anti-rabies injections immediately, while 5.34% (n=41) indicated that it could be given later; say after weeks or even months later (3.52%, n=27).

Majority of respondents indicated that human clinics or hospitals (87.24%, n=670) are the places they can obtain anti-rabies injection in case they were bitten by a suspected rabid dog. A few indicated the veterinary clinic or veterinary hospital (5.08%, n=39) or supermarkets (1.30%, n=10) as possible sources where anti-rabies injections could be obtained following a dog bite.

The results show that 79.56% (n=611) of the respondents were aware that vaccination of pets is important for controlling rabies among humans. This was followed by 18.75% (n=144) who did not know that vaccination of pets is important to prevent humans contracting the disease. Meanwhile, a very small percentage (1.69% (n=13) indicated that vaccination of pets was not important for controlling rabies in humans.

Although a high number of respondents (83.33%, n=640) were aware that rabies could be transmitted to humans from animals, up to 12.76% (n=98) of the study population, expressed ignorance about rabies being transmitted from animals to humans. In addition, some respondents said that rabies could not be transmitted from animals to humans. However, these were very few (3.91%, n=30) (Table 3).

Most respondents (79.04%, n=607) were aware the need for someone to seek medical attention after a bite from a suspected rabid dog. However, the number of positive responses slightly dropped to 71.74% (n=551) when respondents were asked if vaccination against rabies after being bitten by a suspected rabid dog offers protection against development of rabies (Table 3).

Less than half (42.19%; n=324) of the respondents indicated that a 3-month-old dog was eligible to receive the start-up rabies vaccination. This was followed by 28.65% (n=220) who did not know the age at which puppies can get their start-up rabies vaccination and 15.89% (n=122) who thought that puppies can get their start-up rabies vaccination immediately after birth (Table 3).

Table 3: The results of the assessment of the knowledge of rabies

Knowledge variables	Level	Frequency (n)	Percentage %
At what age do pets (dogs or cats) start to receive their vaccination?	Immediately from birth	122	15.89
	From 3 months	324	42.19
	From 12 months	100	13.02

	Never	2	0.26
	Don’t know	220	28.65
Where can you obtain anti-rabies injection if you are a victim of dog bite?	Veterinary clinic or Hospital	39	5.08
	Any supermarket	10	1.30
	Human clinic or Hospital	670	87.24
	Don’t know	49	6.38
Is it possible for rabies to be transmitted from animals to humans?	Yes	640	83.33
	No	30	3.91
	Don’t know	98	12.76
Is it advisable for someone to get anti-rabies after a bite from a suspected rabid dog?	Yes	607	79.04
	No	25	3.26
	Don’t know	135	17.58
	Did not answer	1	0.13
If you are vaccinated against rabies after being bitten by a rabid dog, will that protect you from developing rabies?	Yes	551	71.74
	No	44	5.73
	Don’t know	170	22.14
	Did not answer	3	0.39

In response to the question on the species that are “susceptible to rabies?”. Most respondents (92,2%) mentioned dogs. This was followed by 72,53% who indicated that human beings could contract rabies and just over half (57,42%) who mentioned cats were susceptible to rabies (Fig 1).

Fig 1: Distribution of respondents by responses to questions regarding animals susceptible to rabies.

In response to the question on clinical signs that can make them suspect rabies in dogs, most respondents mentioned (74,61%) signs such as change in behaviour, followed by the inability of a dog to drink and eat (20,83%), and salivation (16,28%) (Fig 2).

Fig 2: The percentage of respondents based on how they responded to the question about the clinical signs of rabies in dogs.

4.1.3 Attitude and practices towards the disease rabies

Results of the attitudes and practices towards rabies are summarised below. Majority of respondents (81.77%, n=628) indicated that it was necessary to receive anti-rabies injection after a dog bite. This was followed by 4.97%; n=115) who did not know that it was necessary to receive anti-rabies, and 3.13% (n=24) did not think that it was necessary to receive anti-rabies injection after a dog bite.

With regards to what should be done to a dog that has bitten someone, 52.47% (n=403) were of the view that such animal should be quarantined. This was followed by 23.83% (n=183) who were of the view that noting should be done to the dog and 13.54% (n=104) and 10.03% (n=77) who indicated that the dog should be killed or chased away respectively.

On the question of whether putting-down a dog suspected of having rabies was appropriate, only 39.45% (n=303), agreed that putting down the dog was the appropriate measure in such circumstances. This was followed by 34.51% (n=265) who did not think that putting down a dog in such circumstances was appropriate, and 25.78% (n=198) who did not know if putting down a dog in such circumstances was appropriate (Table 4).

Under thirty percent (27.21%; n=209) of the study population did not have pets. But among those who owned pets, slightly over half (53.39%, n=410) did not sterilise their pets.

In response to the question how often their dogs get vaccinated 75.91% (n=583) vaccinate their dogs at different intervals. This was followed by 19.14% (n=147), who were not aware of the frequency with which their dogs get vaccinated. Some respondents vaccinated their dogs only once in its lifetime (3.91%; n=30), or never

vaccinate their dogs (1.04%; n=8) (Table 4). Meanwhile, only 59.77% (n=459) of the respondents were able to produce the vaccination certificate as proof that their pets had been vaccinated against rabies.

Sixty-three percent (62.50%; n=408) of the respondents were of the view that vaccination of pets (dog or cat) was important even if the pet is always restrained. In contrast, 10.29% (n=79) did not think it was worthwhile vaccinating their dogs if they are always restraint.

With regards to allowing dogs going out of the yard unsupervised, 43.23% (n=332) did not allow their dogs to go out of the yard unsupervised, while 43.23% (n=332) indicated that they let their dogs go out of the yard unsupervised. But regarding keeping dogs in a fenced place, just over half (58.07%; n=446) indicated that they kept their dogs in a fenced premises to prevent the dogs from going outside unsupervised. The majority (64.32%, n=494) of respondents indicated that they always vaccinated their pets during annual vaccination campaigns, while 8.46% (n=65), indicated that they did not always take their dogs for vaccination during the rabies vaccination campaigns (Table 4).

As indicated in Table 4 below, only 17.84% (n=137) respondents were aware that only laboratory results can be used to declare the animal positive for rabies. However, a higher number (35.42%; n=272) indicated that animals that bite people after confrontation, should be presumed to be rabid. In response to the question what to do when a suspect rabies stray dog is encountered, just over half (53.52%; n=411) said that they would report the dog as a rabies suspect to the department of Agriculture/ or SPCA.

Table 4: Results of the attitude and practices among respondents towards rabies disease

Variables	Levels	Frequency (n)	Percentage %
Do you think it is necessary to receive anti-rabies injection after a dog bite?	Yes	628	81.77
	No	24	3.13
	Don't know	115	14.97
	Did not answer	1	0.13
What should someone who has been bitten by a suspect rabid dog do following a bite?	Nothing	30	3.91
	Leave the wound to heal	20	2.60
	Seek medical attention	692	90.10
	Purchase medication for the wound	25	3.26
	Did not answer	1	0.13
What should be done to dog that has bitten someone?	Nothing	183	23.83
	Kill the dog	104	13.54
	Chase the dog away	77	10.03
	Quarantine the dog	403	52.47
	Did not answer	1	0.13
Do you think it is appropriate to put-down the dog if it is suspected to have rabies?	Yes	303	39.45
	No	265	34.51
	Don't know	198	25.78
	Did not answer	2	0.26
Did you sterilise your pet?	Yes	149	19.40
	No	410	53.39
	Don't have	209	27.21
Do you have a copy of certificate of vaccination for your pet, to produce?	Yes	459	59.77
	No	100	13.02
	Don't have a pet	209	27.21
How often can you vaccinate your dog?	Only once in a lifetime	30	3.91
	Never	8	1.04
	Different interval	583	75.91
	Don't know	147	19.14
Do you keep your dog in a fenced place?	Yes	446	58.07
	No	113	14.71
	Don't have	209	27.21
Do you ever allow your dog to go out of the yard unsupervised?	Yes	227	29.56
	No	332	43.23
	Don't have	209	27.21
Do you always vaccinate your dog when there are annual vaccination campaigns?	Yes	494	64.32
	No	65	8.46
	Don't have a pet	209	27.21
Do you vaccinate your dog even though it is always restrained?	Yes	480	62.50
	No	79	10.29
	Don't have	209	27.21
How do you know if an animals has rabies?	When the animal is lazy	102	13.28
	You only know by laboratory results	137	17.84
	Wait until it suddenly dies	13	1.69
	If it bites people when confronted	272	35.42
	Don't know	244	31.77
What do you do when you see a suspect rabies stray dog?	Nothing	227	29.56
	Kill the dog and throw or bury	80	10.42
	Chase the dog away	50	6.51
	Report to Department of Agriculture/ SPCA)	411	53.52

Respondents were asked a question on how they keep safe from contracting rabies. Majority of respondents (81,77%) indicated they always vaccinate their animals to avoid rabies. Slightly over half (50,26%) of the respondents indicated that they would stay away from stray animals to avoid contracting rabies. Only 12,11% did not know what should be done to remain safe from rabies (Fig 3).

Figure 3: A bar graph showing the proportion of respondents based on their attitude towards rabies in eThekwini District.

4.2 Inferential statistics

~~4.2.1 Results of the univariate analysis~~

~~The results of the univariate analysis are presented in Table 5. There was a significant association between age (p = 0.0037), owning a pets (p =0.0001), religion (p =0.0806), and education (p-value= 0.1258) at generous p-value ≤0.20.~~

~~There was no significance association between sex (p =0.7496) and the location (p = 0.7158) and the outcome variable (Table 5).~~

~~Table 5. The results of the univariate analysis showing the demographic profile of the respondents based on whether they were knowledgeable or not~~

~~Variable~~	~~Level~~	~~[a]Not knowledgeable (%)~~	~~[b]Knowledgeable~~	~~P-value~~
~~Age~~	~~18-35~~	~~85.09~~	~~14.91~~	~~0.0037~~
	~~36-53~~	~~73.96~~	~~26.04~~	
	~~54-71~~	~~79.69~~	~~20.31~~	
	~~>71~~	~~85.71~~	~~14.29~~	
~~Sex~~	~~Male~~	~~81.07~~	~~18.93~~	~~0.7496~~
	~~Female~~	~~80.12~~	~~19.88~~	
~~Residence~~	~~Verulam~~	~~79.95~~	~~20.05~~	~~0.7158~~
	~~Embo~~	~~80.99~~	~~19.01~~	
~~Do you own a pet?~~	~~Yes~~	~~76.74~~	~~23.26~~	~~<0.0001~~
	~~No~~	~~90.43~~	~~9.57~~	
~~Religion~~	~~Christian~~	~~81.42~~	~~18.58~~	~~0.0806~~
	~~African~~	~~77.86~~	~~22.14~~	
	~~Hindu~~	~~80.39~~	~~19.61~~	

	~~Muslim~~	~~67.74~~	~~32.26~~	
	~~Other~~	~~96.30~~	~~3.70~~	
~~Educational level~~	~~No education~~	~~83.72~~	~~16.28~~	~~0.1258~~
	~~Completed primary~~	~~86.14~~	~~13.86~~	
	~~Secondary not complete~~	~~82.18~~	~~17.82~~	
	~~Completed matric~~	~~79.06~~	~~20.94~~	
	~~Tertiary completed~~	~~73.04~~	~~26.96~~	

~~4.2.2 Results of the multivariate analysis~~

Age, owning a pet, religion and educational level were the only variables that were significantly associated with the outcome in the univariable analysis at a generous P-value of ≤ 0.2. These were thus included in the multivariable logistic regression model to identify the factors that were significantly associated with being knowledgeable about rabies.

Only two variables (owning a pet and age of the respondents) were retained in the final ~~multivariate~~ multivariable ~~binary~~ logistic regression model. The respondents who owned pets compared to those who did not, were 3 times (AOR = 2.788; p-value=0.0001) as likely to be knowledgeable about rabies compared to those who did not own pets (reference group) (Table ~~6~~5).

Age was not a significant (p-value >0.05) predictor of knowledge among the respondents. However, it was retained in the final effects model because it was a confounder.

Table ~~5~~6: The results of ~~multivariate~~ multivariable analysis showing factors that were associated with being knowledge about rabies

Variable	Coefficient estimate	Standard error	Adjusted Odds Ratio	95 % CI		P-value
**Own Pet?**				Lower	Upper	
No	**Ref**					
Yes	1.0254	0.2570	2.788	1.721	4.737	0.0001
**Age**						
>71	**Ref**					
18-35	-0.0706	1.1034	0.932	0.149	18.009	0.9490
36-53	0.5991	1.1030	1.821	0.291	35.170	0.5870

54-71	0.2741	1.1388	1.315	0.191	26.364	0.8098

5. DISCUSSION

This is the first study to investigate the knowledge, attitude, and practices toward rabies disease among respondents in the study area. Few respondents were aware that rabies is caused by a virus. Dog bites was identified by a large proportion of the respondents as the main transmission mode for rabies to humans. But on the other hand, just over half of the respondents interviewed mentioned that washing of the wound following a dog bite with soap and running water was important for preventing rabies disease. Very few respondents were aware that licking of an open wound by a suspected rabid dog is one of ways in which rabies spreads from dogs to humans. Similarly, a very low number of respondents in the present study indicated that they knew that rabies disease was fatal. However, many respondents were aware of the importance of receiving anti-rabies vaccine immediately after being bitten by a dog suspected of having rabies to protect the bite victims from developing rabies. Only pet ownership was significantly associated with a high knowledge score for rabies.

5.1 Demographic profile of respondents

This study found that over half of respondents (53.26%) were 18-35 years of age. This the age group that is likely to be more active when it comes to pet management in the homes. Given that more females (63.54%) agreed to participate in the study compared to males, it is possible that females take more keen interest in matters that relate to their pets. However, this observation contrasts with findings by Bihon et al., [6] and Abdela and Teshome [2], who observed that male respondents constituted 71.4% and 59.3% of the study population respectively.

The findings of the present study suggest a high dog/pet ownership in the study area. For example, in comparison to the 72.79% who owned dogs in the present study, only 53% of the respondents owned dogs in a study done in Shirsuphal in western India [39]. Findings from the present study also contrast with those from a study conducted in Cameroon in which less than half of the respondents owned pets [9].

In this study, just below half (47.66%) of respondents were Christians, contrary to findings of studies conducted in Kombolcha and Dedo districts which reported Muslims proportion of 56.8% and 94.8% respectively [1, 20]. The observed difference could be attributed to differences in the dominant religions in the different study areas.

The number of people who had not completed secondary education (35.81%) or had completed matric (high school) (30.47%) in the study population was lower than the number of people who had formal education. However, this is much higher than what Abdela et al. [1] reported. The later observed that half of their study population (51.1%) did not have a formal education. Similarly, the number of people with education higher than primary schooling in the current study was higher than the 52,5% reported in the survey done in Berhampur, Odisha that had only primary school level education [41]. The high number of people who could read and write in the present study is an encouraging finding because it points to a target population that is amiable to training given that a lot of training material on rabies is in English.

5.2 Knowledge of the disease rabies

The very low number (26.95%; n=207) of respondents who were aware that a virus was the agent responsible for rabies, is in line with results from the study conducted in Dessie City, where only 18% (n=25) of respondents regarded a virus to be the cause of rabies [19]. However, in a study conducted in Kombolcha, Ethiopia, a higher number (76.6%; n=294) of respondents were aware that rabies is caused by a

virus [20]. This difference suggests a need to improve on the training of residents in the study area and particularly make it clear what causes rabies.

A very high number of respondents (86.98%) who mentioned dog bite as a mode of transmission for rabies to humans is an encouraging finding from a public health point of view. This aligns with findings from a study by Fesseha et al. [18], which reported almost a similar proportion (85%) of respondents who mentioned that rabies virus was transferred through a bite from an infected animal. However, a slightly lower number from the Philippines (79.5%) than those observed in the present study, were aware that dog bites play a crucial role in the transfer of rabies to people [5]. Similarly, a study conducted in Haiti also observed that 75.6% of respondents knew that rabies can be transmitted via a dog bite [17]. The findings of this study shows that the knowledge about the transmission of rabies in the study is comparable to that observed in other developing countries such as Ethiopia and the Philippines. On the other hand, results of this study indicate more people than what has been reported in other countries were knowledgeable about the transmission of rabies.

Licking of an open wound by a dog as mode of transmitting rabies, was mentioned by a mere 11.46% of the respondents in the present study. Similarly, a very low (1.8%) number of community members in a study conducted in Haiti were aware that rabies could be transmitted to people through contact of saliva with an open wound [17].

While most respondents mentioned biting as one of the ways the virus gains entry to the victim, licking of the open wound by the suspected animal was not considered important in the spread of rabies. This suggest that if the dog licked humans in the study area, they would probably not attempt to seek medical care. This would hence put them at risk of rabies infection.

Just over half (53.52%) of this study's respondents considered washing the wound with soap and running water as the first aid to prevent rabies. This low number, is of concern, given the importance of post exposure prophylaxis that includes washing the wound immediately with soap and water to prevent infection gaining entry to the victim. In contrast, a higher number (92.4%), of respondents from the study in and around South Gondor in Northwest Ethiopia indicated that washing the wound with soap was the first measure to be implement following a bite by a suspected rabid dog before seeking other preventative measures [20]. Findings reported here also contrasted with those reported by Bihon et al [6], who observed that a very low number of respondents (8.9%) regarded wound washing as an immediate response to prevent rabies. In view of the findings of the present study, while the number of people who acknowledge that washing the bite wound was important as the first step in the prevention of rabies is higher than what Bihon et al. [6] observed in South Gondor. Therefore, there is room for improvement on understanding how to prevent rabies in case of a bite among residents in the study area.

In this study, only 16% of respondents considered rabies a fatal disease. This figure is much lower than the findings from Bihon et al [6] and Abedela and Teshome [2], who observed that 84.6% and 82% of people, respectively, were aware that rabies cannot be cured once clinical signs appear. The fact that only a few respondents in this study were aware that rabies is not treatable, is significant and suggests people are most likely not to seek medical care early. It is critical that this kind of information be included in campaigns to educate the community about the seriousness of the disease rabies.

A considerable number of respondents (78%) in the present study knew that they need to obtain anti-rabies vaccine immediately after being bitten by dog

suspected of having rabies. A slightly lower response (65.2%) was reported in Dedo district of Jimma zone, Ethiopia [1]. Even a much lower number was reported by Abdela and Teshome [2] who observed that only 32.7% of the respondents in their study indicated that anti-rabies should be administered immediately after exposure. Although a much higher number of respondents in this study were aware of the importance of receiving anti-rabies vaccines immediately after being bitten by a suspect dog, there is room for improvement to bring the number closer to 100%. The authors believe that everyone should know that receiving anti-rabies treatment immediately after one has been bitten by a dog suspected of having rabies is necessary to eradicate rabies related deaths. The difference observed between the different studies suggest a difference in the effectiveness of extension services in the different study areas. It is evident from the findings reported here that there is room for improvement in the study area when it comes to the importance of receiving anti-rabies vaccine immediately following a dog bite.

Lack of knowledge among the general public on the role other species besides dogs play in the spread of rabies, seems to be widespread. For example, although in this study a large number (90%) of community members mentioned dogs as being susceptible to rabies, other animal species were mentioned less frequently. Similarly, a high proportion of respondents in other studies conducted in Kansas (96.8%) and Gondar Zuria District (100%), largely pointed to dogs as being the one that is susceptible to rabies [11, 27]. Only a study conducted in Harare Metropolitan reported few respondents (37.59%) who identified dogs as the only animal susceptible to rabies [36]. The widespread lack of knowledge of the role played by other species besides dogs in the spread of rabies, points to a gap in the education programmes communities

are exposed to. Therefore, there is a need to place emphasis on the role other species play in the spread of rabies during extension to communities.

The over three quarters (79.75%) of the respondents in this study who were aware that vaccinating pets was significant to keep humans safe from rabies, is consistent with findings of other studies. For example, most respondents (68%) from Jamma also believed that vaccination against rabies in dogs helps prevent the disease in humans [38]. Dubey et al. [12] reported a slightly higher proportion of respondents who believed that dog vaccination was a good practice in a study conducted in Jabalpur central India. The high number of respondents who were aware of the importance of vaccinating dogs to control rabies is good news. This is because vaccination of pets creates a barrier of diseases shared among pets and humans given that they occupy the same space.

The slightly less than half (42.19%) of the respondents in the present study who favoured vaccinating their pets at 3 months old, was a bit higher than the finding of a study conducted in Kigali city, Rwanda, were only 20.6% of respondents vaccinated their puppies by the age 3 months [31]. On the other hand, Bouaddi et al. [7] reported a much lower percentage of respondents (14.7%) who favoured having their dogs vaccinated at 3 months of age. However, in the Democratic Republic of Congo up to 14% of respondents believed that young puppies should not be vaccinated [27]. The differences observed in studies conducted elsewhere and the present study, the higher figures observed in this study, could be attributed to the rigour with which rabies eradication campaigns are conducted in the study area. In fact, in South Africa, rabies campaigns target puppies from 3 months of age and older as part of the eradication scheme aimed at eliminating rabies within the country.

The very high number of respondents (87.24%) in this study who preferred to be treated from a human clinic or hospital after exposure to a dog bite, is a welcome observation. It is important for people to know where they can be treated following exposure for effective rabies prevention. This result is consistent with what Edukugho et al. [14] reported. The latter observed that more than 86% of respondents would report to the hospital or see a doctor after being bitten by dogs. However, a report by Fesseha et al. [18], observed that only 55.6% of respondents would seek medical attention after a dog bite. This low number of people who would seek medical care reported by Fesseha et al. [18] in comparison to what was observed in the present study, suggests that the education of the public regarding how to treat exposed people is very impactful in the study area.

Although up to 83.33% of the respondents in this study knew that rabies can be transmitted from animals to humans, the number that did not know, even though small, cannot be ignored. However, these findings agree with those of studies done in Munesa district and Tigray regions of Ethiopia, in which 88.7% and 89.5%, respectively, indicated that humans were likely to contract rabies from animals [2,13].

The high number of respondents (79.04%) in the present study who were aware of the importance of obtaining anti-rabies vaccination following a bite from a suspected rabid dog, contrasts with findings from Odisha, India [41] and Ethiopia [11], which reported low numbers of respondents (14% and 38.8% respectively) who were aware of the importance of receiving anti-rabies vaccination after being bitten by a dog. This could be attributed to differences in the proximity to health care facilities in the different study areas. In instances where health facilities are readily accessed, although some of the bite victims may go to a health facility with the intention of getting anti-rabies,

they also end up receiving wound treatment. This could contribute to the high numbers seeking medical care observed in some studies as opposed to others.

The 71.74% of the respondents in the present study that were aware that if vaccinated against rabies following a bite by a rabid dog, a person is protected from developing rabies, is lower than the 87% of respondents from a study conducted in Abuja, Nigeria, that had visited health care facilities to obtain treatment following a dog bite [14]. Nonetheless, the high numbers of people in this study, who were aware that receiving the anti-rabies vaccine is important in preventing the disease in humans, further points to the effectiveness of the education campaign in the study area. This contrasts with finds of another study conducted in the Tigray region of Ethiopia, in which just over half (54.9%) of respondents preferred to go to traditional healers instead of hospitals following a dog bite incident [13].

It was encouraging to observe that majority of the respondents (74.61%) in this study were aware that a change in behaviour including dogs biting people were major clinical sign of rabies. This is in line with the findings reported in Dedo district, Southwest Ethiopia [1], Gondor Zuria, Ethiopia [11] and Gondar, Northwest [6], where up to 50.4%, 63.5% and 75.8% of the respondents respectively, knew that behaviour change is one of the signs of rabies.

On the other hand, paralysis as a clinical sign of rabies did not feature predominantly. Therefore, paralysis was likely to be overlooked as a sign of rabies by the study population. This is worth noting because, when such people get bitten by a rabid dog even if it showed paralytic signs, they are likely not to seek post exposure prophylaxis.

5.3 Attitude and practices of community members towards rabies

The considerable proportion of respondents (81.77%) in this study who showed a willingness to receive anti-rabies vaccine to help prevent the disease in case of dog bite, is a positive finding. This implies that people are willing to obtain anti-rabies treatment as soon as possible after exposure to prevent rabies, given that currently there is no available treatment once the disease has developed. The number of people willing to received anti-rabies in the current study was higher than 51% from Berhampur, Odisha, in India, that reported were willing to obtain anti-rabies as a control measure rabies following a dog bite, a country that is also endemic for rabies, and accounts for 36% of the world’s rabies deaths [41]. An even lower percentage (38.8%) of people from Gondar Zuria District, Ethiopia, were willing to obtain anti-rabies in response to dog bite [11]. Just like India and South Africa, Ethiopia is endemic to rabies. The difference observed with respect to the number of people with a positive attitude towards receiving anti-rabies vaccine in the different countries that are endemic to rabies, could be attributed to the nature of education programmes implemented in the different countries. It could also be attributed to the differences in the level of education in the different study areas.

The overwhelming majority of respondents (90.10%) in this study were willing to seek medical attention following exposure to a dog bite victim. This is consistent with results of a survey done in Kombolcha, Ethiopia where 89.3% of respondents would seek medical care if they were bitten by an animal suspected to have rabies [20]. However, results from the present study widely contrasted with findings of a survey conducted in and around South Gondar, Ethiopia, in which it was observed that 79.4% of respondents were not in favor of visiting health care centers following dog bites [6].

The 52.47% of respondents who indicated that they would allow their dogs to be quarantined for observation after biting someone, contrasts with 69% of the respondents in a study from Kigali City, Rwanda, who were not in favor of isolating their animals if they bit people [31]. However, the number of people willing to have their dogs quarantined in the present study were far higher than those reported in a study conducted in Cameroon, where only 2.6% favored confining their dogs for observation when they start behaving unusually, including biting people [9].

While a low percentage of respondents (39.45%) in the present study that indicated that they would put down their dogs if they were suspected to be rabid, many more, either did not think that putting down the dog suspected of rabies was appropriate or did not know what to do with such animals (60,29%. This sharply contrasts with results of studies conducted in South Gondar and Kombolcha where 64.3% and 84.1%, respectively, indicated that they would kill the dog suspected to have rabies [6,20]. The low numbers of people willing to have their animals put down if they are suspected to be rabid in this study, could be influenced by the belief that rabies can be treated. It could also be influenced by religious belief among certain ethnic or religious groups that an animal should die naturally without human interference.

The sterilization rate in the study area was low with just over half (53.39%) of the respondents in the study population having not sterilised their pets. The low sterilization rate of pets suggests that many dogs in the study area can breed. The problem with this, is the potential to increase the risk of rabies due to the resultant high number of dogs in the study area. The larger the population of dogs in the area, the more dogs are likely to roam freely, especially during mating season when they want to reproduce. This is why, dog population control is likely to curtail the spread of rabies.

The number of people willing to sterilise their pets in the present study contrasts with the numbers reported by a study conducted in Sri Lanka by Ubeyratne et al. [43], who reported that up to 86.41% had not sterilised their pets. But findings of the present study are consistent with those of a study conducted in El Jadida, Morocco, that reported that just under half (45%) of respondents did not believe in the practice of sterilizing pets [7].

A copy of the rabies vaccination certificate was requested from respondents as a confirmation of pets in the study areas having been vaccinated. A vaccination certificate was recorded as valid if the pet had received the rabies vaccine the previous year or in the same year the study was conducted. Only 58.77% of the respondents in the present study were in position of a vaccination certificate as proof of vaccination of their pets. This finding is suggests that the vaccination coverage in the study area is below the 70% vaccination coverage recommended by the World Health Organization (WHO) [7] to prevent rabies outbreaks in humans. This finding is a serious public health concern because it shows that the area is susceptible to experiencing a rabies outbreak. However, findings reported here, contrast with reports of studies conducted elsewhere. For example, in the El Jadida Region, Morocco [7], it was observed that 76.6% of the respondents had rabies vaccination certificate for their pets. Ntampaka et al. [31], observed that the majority (81.8%) of respondents in their study had vaccinated their animals to prevent them from getting rabies and 81.2% of the respondents from Southern Wollo, Ethiopia indicated that vaccination was significant to maintain a society free from rabies [20].

However, a low vaccination coverage, has also been observed in other parts of the continent. For example, Costa et al. [9], reported a low vaccination coverage (30.8%) of pets in their study conducted in Cameroon. It is evident that the study area

is doing better than other areas parts of other countries. But that notwithstanding, there is room for improvement in the study area to ensure that the vaccination coverage is at the level recommended by the WHO.

The 75.91% of respondents in this study that showed a willingness to vaccinate their pets following guidelines provided by the State to vaccinate dogs, is higher than the findings reported in El Jadida, Morocco, where only 67.8% of the respondents appreciated annual dog vaccination [7]. An even a lower response was observed among respondents in Northwest Ethiopia, where only 28.1% knew that dogs should be vaccinated repeatedly to prevent rabies [6]. Adhering to repeated and regular rabies vaccination helps boost the immune level of pets and thus minimize outbreaks of rabies.

The 50.26% in this study who mentioned that to avoid getting rabies from animals, one must avoid any contact with stray dogs, sharply contrasts with the 81.77% who advocated for animals getting vaccinated. The 50.26% in the present study also contrasts with only 33.3% of respondents in Kansas who expressed the view that avoiding contact with animals one is not familiar with, is key to preventing rabies [37].

Over half (58.07%) of respondents in the present study, kept their dogs behind a fence, which is far less than the number of respondents (84%) in Shirsuphal who kept their dogs in a fenced place to limit them from going outside the households [39]. On the other hand, a lower percentage of respondents (25%) in Filipinos kept their animals in an enclosed environment to prevent them from roaming freely [10]. Results reported here contrast with those in Bobo Dioulasso, Burkina Faso, where up to 85% of the participants kept their animals in closed places to prevent them from roaming in the streets [35]. Keeping dogs from contact with other stray animals limits the chance

of rabies infection. Therefore, the lack of fences around homes with dogs increases the contact of animals which may lead to the rapid spread of rabies. Thus, confinement of dogs is very important and needs to be improved on in the study area.

Over one quarter (29.56%) of respondents in the present study indicated that they occasionally allowed their dogs to move freely out of the yard unsupervised. However, this figure is lower than what was reported by Costa et al. [9] and Corfmat et al. [8], who reported a significantly higher percentage of respondents (61% and 59%, respectively), who indicated that they occasionally allowed their dogs to move out of the yard freely. Dogs that move freely risk contracting the rabies virus from other infected animals.

Just as was observed that 64% of the respondents in the Filipinos study consistently vaccinated their dogs to prevent rabies [10], up to 64.32% of respondents in the current study regularly presented their dogs for vaccination during vaccination campaigns. Similarly, a study conducted in south Gondar also reported that 69.8% of respondents showed commitment to dog vaccination [6]. However, although the number of people who consistently vaccinated their dogs in the present study was high, it was below the recommended 70% vaccination coverage by WHO [7]. The low number could be because some people are not available during the campaigns due to work or other commitments, while others are unable to handle their dogs and end up not presenting their dogs for vaccination. It is also possible that the high number of dogs that are not confined that was observed in this study explains the low numbers of people who consistently present their dogs for vaccination. This is because such dogs are likely not to be available to be immunized during the time of the vaccination campaign.

It was encouraging to note that 62.50% of respondents in the present study indicated that dog vaccination is necessary even for dogs that are kept in closed yards all the time. This finding has potential to boost vaccination as a tool to drive elimination off dog-mediated human rabies.

A very lower response (17.84%) was noted with respect to submitting the carcass of suspected animals to the laboratory to confirm if it had rabies. The results of the study conducted in the Democratic Republic of the Congo about submitting carcasses for confirmation of rabies, was worse, with only 2% of respondents indicating that they would take the carcass of a suspected rabid animal to veterinary services for laboratory testing [27]. Effort is needed to increase awareness of the need to submit carcasses of animals that have died for laboratory confirmation. This has the potential to assist in early outbreak detection and improving surveillance efforts.

A mere 53.52% of the respondents in the present study who would report animals suspected of being rabies positive to the authorities, contrasts with a higher percentage (73%) of respondents from Shirsuphal village in western India, who would report the presence of rabid dogs to the municipal authorities if they ever encountered a case of rabies [40]. However, in Debark Worenda, Ethiopia 84.1% of the respondents said that they would kill the animal if they suspected it had rabies [45]. In the study by Mbilo et al [27], only 20% of respondents indicated that they would alert the veterinary services if they recognized a suspected rabid animal. This completely contrasts with the findings of the present study, and this difference could be attributed to the impact of the rabies eradication campaigns in the different areas. Behavioral change in a suspected rabid animal was reported by a higher number of respondents, while other signs of rabies were mentioned less. These findings are of public health importance in that respondents are likely to report to the authorities when they observe

behavior change, which is an important sign of rabies. However, studies show that aggressive animals are likely to become tame when rabid. As a result, it might be difficult to identify when the animal is a stray.

5.4 Factors significantly associated with knowledge

The finding that respondents who owned pets had significantly higher odds (AOR: 2.788; P=0.0001) of being knowledgeable about rabies in comparison to respondents who did not own pets, is consistent with the findings by Abdela et al. [1]. The later also observed that respondents who owned dogs had significantly (P=0.029) higher odds of obtaining a good KAP score than those who did not own dogs. Similar results were observed among residents of Abuja municipal area, where it was found that people who owned dogs were 7.8 times more likely to have a better knowledge of rabies than those who had no dogs [14]. This is explained by the fact that people who own pets are more likely to receive information during rabies campaigns when they take their dogs for vaccination.

6. CONCLUSION AND RECOMMENDATIONS

There are knowledge gaps with respect to certain aspects such as rabies being a viral disease, how one can protect oneself from contracting rabies, precautionary measures to adopt following exposure to rabies and how to protect susceptible animals from being exposed to rabies. The role of measures such erecting fences around homesteads to restrict animal movements and neutering of pets to control dog population need to be emphasized during extension. The low number of people who had vaccination certificates, although worth noting, needs further investigate to establish whether respondents do not keep certificates, or if it is a true reflection of the status of the vaccination coverage.

The study identified that dog ownership was the only socio-demographic factor that was a significant predictor of knowledge on rabies. In view of this, information dissemination needs to be inclusive to include non-pet owners in the area who also constitute the population at risk. On a positive side, the study demonstrated that there was widespread knowledge on some aspects like knowledge of the role of dog bites in the spreading of rabies in the study area, which suggests that people are likely to seek medical attention in case of a dog bite incidence. In addition, a good attitude towards receiving PEP following a dog bite and reporting a suspected cases were widespread in the study.

To bridge the gaps in knowledge that were identified in this study, implementation of global strategies such as awareness and vaccination campaigns need to be stepped up. Information to sensitize people can be found from government distributed pamphlets or by watching “If Only I Knew” video and by also consulting Government Veterinary Services (GVS). In addition, programs to enforce the positive aspects identified in this study should continuously be implemented. This will help improve the control and prevention efforts directed towards minimizing and eradicating rabies outbreaks including associated human fatalities. A one health approach that involves collaboration between veterinary services with other public health institutions and local authorities to expand and strengthen rabies awareness should be adopted in the study areas to achieve 2030 goal of rabies elimination in rabies endemic areas.

7. Limitations

This study was subject to limitations of cross-sectional surveys such as recall biases. Furthermore, the study was limited to Verulam and Embo in eThekwini District; hence, the results cannot be generalised to other areas in South Africa. The study was conducted during the lockdown due to the COVID-19 pandemic, when movement of

people was restricted and people were reluctant to meet strangers, which could have led to selection bias, which thus calls for caution when interpreting the results. Nonetheless, this study presents baseline information on the knowledge, attitude, and practices towards rabies, which was previously unavailable in the study area. It also provides baseline information upon which future research can be developed.

8. ACKNOWLEGEMENTS

The authors would like to appreciate the ~~funding from the~~ University of South Africa for partially funding this study through the Post-graduate Bursary, as well as the participants for consenting to participate in this study, and their cooperation throughout the study. We would like to acknowledge the Department of Agriculture and Rural Development, Veterinary services section, KZN, for permitting us to carry-out the study within eThekwini district. We would also like to extend gratitude to UNISA ethics committee for approving this study to be conducted.

REFERENCES

1. Abdela, N., Midekso, B., Jabir, J. and Abdela, W. (2017) ‘Knowledge, attitudes and practices towards rabies in Dedo district of Jimma zone, southwestern Ethiopia: A community based cross - sectional study’, 9(May), pp. 61–71. Available at: https://doi.org/10.5897/IJMMS2017.1302.
2. Abdela, N. and Teshome, E. (2017) ‘Community based cross-sectional study on knowledge, attitudes and practices towards rabies in Munesa District, Arsi Zone, Southeastern Ethiopia’, *Journal of Public Health and Epidemiology*, 9(6), pp. 161–170. Available at: https://doi.org/10.5897/jphe2017.0931.
3. Ali, A., Ahmed, E.Y. and Sifer, D. (2014) ‘A Study on Knowledge, Attitude and Practice of rabies among residents in Addis Ababa, Ethiopia’, *Ethiopian*

Veterinary Journal, 17(2), p. 19. Available at: https://doi.org/10.4314/evj.v17i2.2.

4. Ba, M.F., Kane, N.M., Diallo, M.K.K., Bassoum, O., Boh, O.K., Mboup, F.Z.M. *et al.* (2021) 'Knowledge, attitudes and practices on rabies among human and animal health professionals in senegal', *Pathogens*, 10(10), pp. 1–15. Available at: https://doi.org/10.3390/pathogens10101282.
5. Barroga, T.R.M., Basitan, I.S., Lobete, T.M., Bernales, R.P., Gordoncillo, M.J.N., Lopez, E.L. *et al.* (2018) 'Community Awareness on Rabies Prevention and Control in Bicol, Philippines: Pre- and Post-Project Implementation', *Tropical Medicine and Infectious Disease*, 3(1), pp. 1–14. Available at: https://doi.org/10.3390/tropicalmed3010016
6. Bihon, A., Meresa, D. and Tesfaw, A. (2020) 'Rabies: Knowledge, Attitude and Practices in and Around South Gondar, North West Ethiopia', *Diseases*, 8(1), p. 5. Available at: https://doi.org/10.3390/diseases8010005
7. Bouaddi, K., Bitar, A., Bouslikhane, M., Ferssiwi, A., Fitani, A. and Mshelbwala, P.P. (2020) 'Knowledge, attitudes, and practices regarding rabies in El jadida region, Morocco', *Veterinary Sciences*, 7(1). Available at: https://doi.org/10.3390/vetsci7010029
8. Corfmat, J. Gibson, A.D., Mellanby, R.J, Watson, W., Appupillai, M., Yale, G. *et al.* (2022) 'Community attitudes and perceptions towards free-roaming dogs in Goa, India', *Journal of Applied Animal Welfare Science*, 00(00), pp. 1–17. Available at: https://doi.org/10.1080/10888705.2021.2014839.
9. Costa, G.B., Gilbert, A., Monroe, B., Blanton, J., Ngam, S.N., Recuenco, S. *et al.* (2018) 'The influence of poverty and rabies knowledge on healthcare

seeking behaviors and dog ownership, Cameroon', *PLoS ONE*, 13(6). Available at: https://doi.org/10.1371/journal.pone.0197330.

10. Davlin, S.L. Lapiz, S.M., Miranda, M.E. and Murray, K.O. (2014) 'Knowledge, attitudes, and practices regarding rabies in Filipinos following implementation of the Bohol Rabies Prevention and Elimination Programme', *Epidemiology and Infection*, 142(7), pp. 1476–1485. Available at: https://doi.org/10.1017/S0950268813002513.
11. Digafe, R.T., Kifelew, L.G. and Mechesso, A.F. (2015) 'Knowledge, attitudes and practices towards rabies: Questionnaire survey in rural household heads of Gondar Zuria District, Ethiopia', *BMC Research Notes*, 8(1). Available at: https://doi.org/10.1186/s13104-015-1357-8.
12. Dubey, S., Singh, R.V., Gupta, B., Gupta, D.K., Nyak, A., Rai, A. *et al.* (2022) 'Study on rabies control and prevention using knowledge attitude and practice in Jabalpur central India', 11(7), pp. 2353–2359.
13. Ebuy, Y., Alemayehu, T., Reda, M., Berhe, M. and Bsrat, A. (2019) 'Community Knowledge, Attitude and Practice on Rabies, Incidence in Humans and Animals and Risk Factors to Rabies in Selected Districts of Tigray Region, Ethiopia', *Nigerian Veterinary Journal*, 40(2), pp. 147–163.
14. Edukugho, A.A., Umoh, J.U., Diem, M., Ajani, O., Uba, B., Okeke, L. *et al.* (2018) 'Knowledge, attitudes and practices towards rabies prevention among residents of Abuja municipal area council, Federal Capital Territory, Nigeria', *Pan African Medical Journal*, 31, pp. 1–11. Available at: https://doi.org/10.11604/pamj.2018.31.21.15120.
15. eThekwini Municipality (2017) Integrated Development Plan (IDP) Annual Review - *2017/2018*; Durban eThekwini Municipality; 2017.

16. Fazeli, M., Golahdouz, M., Bashar, R., Bafrrani, M.A., Doosti-Irani, A., Pourhossein, B. *et al.* (2018) 'Design and Validation of a Persian Knowledge, Attitude, and Practice Questionnaire for Rabies (PKAP-Rabies) in the General Population of Iran', *Journal of Medical Microbiology and Infectious Diseases*, 6(2), pp. 67–71. Available at: https://doi.org/10.29252/jommid.6.2.3.67
17. Fenelon, N., Dely, P., Katz, M.A., Schaad, N.D., Dismer, A., Moran, D. *et al.* (2017) 'Knowledge, attitudes and practices regarding rabies risk in community members and healthcare professionals: Pétionville, Haiti, 2013', *Epidemiology and Infection*, 145(8), pp. 1624–1634. Available at: https://doi.org/10.1017/S0950268816003125
18. Fesseha, H. and Hadgu, A. (2020) 'Assessment of Knowledge, Attitude and Practices on Rabies among Communities of Atsbi Wonberta District, Tigray, Northern Ethiopia', *Current Research in Agriculture and Farming*, 1(1), pp. 21–31. Available at: https://doi.org/10.18782/2582-7146.104
19. Gebeyaw, S. and Teshome, D. (2016) 'Study on Community Knowledge, Attitude and Practice of Rabies in and Around Dessie City', *Austin Journal of Veterinary Science & Animimal Husbandry*, 3(1), p. 5.
20. Gebremeskel, K.A., Tanga, B.M., Getachew, A. and Eshetu, Y. (2019) 'Assessment of public knowledge, attitude and practices towards rabies in the community of Kombolcha, Southern Wollo, Amhara Reginal State, Ethiopia', *Journal of Public Health and Epidemiology*, 11(1), pp. 38–48. Available at: https://doi.org/10.5897/jphe2017.0965
21. Guadu, T., Shite, A., Chanie, M., Bogale, B. and Fentahun, T. (2014) 'Assessment of Knowledge, Attitude and Practices about Rabies and

Associated Factors: In the Case of Bahir Dar Town', *Global Veterinaria*, 13(3), pp. 348–354. Available at: https://doi.org/10.5829/idosi.gv.2014.13.03.8579.

22. Hampson, K., Coudeville, L., Lembo, T., Sambo, M., Kieffer, A., Attlan, M. *et al*. (2015) 'Estimating the Global Burden of Endemic Canine Rabies', *Plos Neglected Tropical Diseases,* 9(4): e0003709. Available at: https://doi.org/10.1371/journal.pntd.0003709

23. Hergert, M., LeRoux, K. and Nel, L.H. (2016) 'Risk factors associated with nonvaccination rabies status of dogs in KwaZulu-Natal, South Africa', *Veterinary Medicine: Research and Reports*, Volume 7, pp. 75–83. Available at: https://doi.org/10.2147/vmrr.s103859

24. Hergert, M. and Nel, L.H. (2013) 'Dog Bite Histories and Response to Incidents in Canine Rabies-Enzootic KwaZulu-Natal, South Africa', *PLoS Neglected Tropical Diseases*, 7(4), pp. 1–11. Available at: https://doi.org/10.1371/journal.pntd.0002059

25. Kiratitana-olan, K., Chaisowwong, W., Thongkorn, K. and Kreausukon, K. (2021) 'Keys to Good Knowledge, Attitude and Practice for Rabies Prevention in Disease-Free Communities in Thailand', *Veterinary Intergrative Sciences,* 19(3): 407-422

26. Lankester, F.J., Wouters, P.A.W.M., Czupryna, A., Palmer, G.H., Mzimbiri, I., Cleaveland, S. *et al.* (2016) 'Thermotolerance of an inactivated rabies vaccine for dogs', *Vaccine*, 34(46), pp. 5504–5511. Available at: https://doi.org/10.1016/j.vaccine.2016.10.015

27. Mbilo, C., Kabongo, J., Pyana, P.P., Nlonda, L., Nzita, R.W., Luntadila, B. *et al.* (2019) 'Dog ecology, bite incidence, and disease awareness: A cross-sectional survey among a rabies-affected community in the democratic

republic of the congo’, *Vaccines*, 7(3). Available at: https://doi.org/10.3390/vaccines7030098

28. Morters, M.K., McNabb, S., Horton, D.L., Fooks, A.R., Schoeman, J.P., Whay, H.R. *et al.* (2015) ‘Effective vaccination against rabies in puppies in rabies endemic regions’, *Veterinary Record*, 177(6), p. 150. Available at: https://doi.org/10.1136/vr.102975
29. Ngah Osoe Bouli, F.P., Awah-Ndukum, J., Tejiokem, C.M. and Mingoas Kilekoung, J. (2018) ‘Knowledge, attitudes and practices (KAP) evaluation regarding rabies in the Centre, Adamawa and North Regions of Cameroon’, *Journal of Animal Science and Veterinary Medicine*, 3(6), pp. 178–183. Available at: https://doi.org/10.31248/jasvm2018.114
30. Ngugi, J.N., Maza, A.K., Omolo, O.J. and Obonyo, M. (2018) ‘Epidemiology and surveillance of human animal-bite injuries and rabies post-exposure prophylaxis, in selected counties in Kenya, 2011-2016’, *BMC Public Health*, 18(1). Available at: https://doi.org/10.1186/s12889-018-5888-5
31. Ntampaka, P., Nyaga, P.N., Niragire, F., Gathumbi, J.F. and Tukei, M. (2019) ‘Knowledge, attitudes and practices regarding rabies and its control among dog owners in Kigali city, Rwanda’, pp. 1–16. Available at: https://doi.org/10.1371/journal.pone.0210044
32. Pal, P., Yawongsa, A., Bhusal, T.N., Bashyal, R. and Rukkwamsuk, T. (2021) ‘Knowledge, attitude, and practice about rabies prevention and control: A community survey in Nepal’, *Veterinary World*, 14(4), pp. 923–942. Available at: https://doi.org/10.14202/vetworld.2021.933-942
33. Rine, R.C., Dogara, G.S. and Pam, M.D. (2017) ‘Knowledge, Attitude and Practice of Rabies in and Around Lafia Metropolis, Nigeria’, *Central African*

Journal of Public Health, 3(3), pp 27-33. Available at: https://doi.org/10.11648/j.cajph.20210303.11

34. Sabeta, C.T., Weyer, J., Geertsma, P., Mohale, D., Miyen, J., Blumberg, L.H. *et al.* (2013) 'Emergence of rabies in the Gauteng Province, South Africa: 2010-2011', *Journal of the South African Veterinary Association*, 84(1), pp. 2010–2011. Available at: https://doi.org/10.4102/jsava.v84i1.923
35. Savadogo, M., Tialla, D., Ouattara, B., Dahourou, L.D., Ossebi, W., Ilboudo, S.G. *et al.* (2021) 'Factors associated with owned-dogs' vaccination against rabies: A household survey in Bobo Dioulasso, Burkina Faso', *Veterinary Medicine and Science*, 7(4), pp. 1096–1106. Available at: https://doi.org/10.1002/vms3.468
36. Spargo, R.M., Coetzer, A., Makuvadze, F.T., Chikerema, S.M., Chiwerere, V., Bhara, E. *et al*. (2021) 'Knowledge, attitudes and practices towards rabies: A survey of the general population residing in the Harare Metropolitan Province of Zimbabwe' *PLos ONE* 16(1): e0246103. Available at: https://doi.org/10.1371/journal.pone.0246103
37. Straily, A. and Trevino-Garrison, I. (2017) 'Knowledge, Attitudes and Practices of Law Enforcement Officers on Rabies and Animal Control Issues in Kansas', pp. 111–117. Available at: https://doi.org/10.1111/zph.12287
38. Tandon, S., Kotwal, S.K., Malik, M.A., Singh, M., Kumar, D., Shafiq, M. *et al.* (2017) 'A Community Based Survey on Rabies Control and Prevention using KAP in', (December), pp. 1019–1028. Available at: https://doi.org/10.5958/2277-940X.2017.00153.X
39. Tiwari, H.K., Robertson, I.D., O'Dea, M. and Vanak, A.T. (2019) 'Knowledge, attitudes and practices (KAP) towards rabies and free roaming dogs (FRD) in

Panchkula district of north India: A cross sectional study of urban residents', *PLoS Neglected Tropical Diseases*, 13(4), pp. 1–17. Available at: https://doi.org/10.1371/journal.pntd.0007384

40. Tiwari, H.K., O'Dea, M., Robertson, I.D., and Vanak, A.T. (2019) 'Knowledge, attitudes and practices (KAP) towards rabies and free-roaming dogs (FRD) in Shirsuphal village in western India: A community based cross-sectional study', *PLoS Neglected Tropical Diseases*, 13(1). Available at: https://doi.org/10.1371/journal.pntd.0007120
41. Tripathy, R.M., Satapathy, S.P. and Karmee, N. (2017) 'Assessment of knowledge, attitude and practice regarding rabies and its prevention among construction workers: a cross-sectional study in Berhampur, Odisha', *International Journal of Research in Medical Sciences*, 5(9), p. 3971. Available at: https://doi.org/10.18203/2320-6012.ijrms20173964
42. Tschopp, R., Bekele, S. and Aseffa, A. (2016) 'Dog Demography, Animal Bite Management and Rabies Knowledge-Attitude and Practices in the Awash Basin, Eastern Ethiopia', *PLoS Neglected Tropical Diseases*, 10(2). Available at: https://doi.org/10.1371/journal.pntd.0004471
43. Ubeyratne, K., Srikitjakarn, L., Pfeiffer, D., Kong, F., Sunil-Chandra, N., Chaisowwong, W. *et al.* (2020) 'A Knowledge, Attitudes and Practices (KAP) survey on canine rabies prevention and control in four rural areas of Sri Lanka', *Transboundary and Emerging Diseases*, (November), pp. 1–15. Available at: https://doi.org/10.1111/tbed.13940
44. Weyer, J., Dermaux-Msimang, V., Grobbelaar, A., LeRoux, C., Moola, N., Paweska, J. *et al.* (2020) 'Epidemiology of human rabies in South Africa, 2008

- 2018’, *South African Medical Journal*, 110(9), pp. 877–881. Available at: https://doi.org/10.7196/SAMJ.2020.v110i9.14324

45. Yalemebrat, N., Bekele, T. and Melaku, M. (2016) ‘Journal of Veterinary Medicine and Animal Health Assessment of public knowledge, attitude and practices towards rabies in Debark Woreda, North Gondar, Ethiopia’, *Journal of Veterinary Medicine and Animal Health*, 8(11), pp. 183–192. Available at: https://doi.org/10.5897/JVMAH2016.0504

Supporting information

S1 Fig. Fig 1: Distribution of respondents by responses to questions regarding animals susceptible to rabies.

S2 Fig. Fig 2: The percentage of respondents based on how they responded to the question about the clinical signs of rabies in dogs.

S3 Fig. Fig 3: A bar graph showing the proportion of respondents based on their attitude towards rabies in eThekwini District

***Knowledge, attitude, and practices towards rabies: a community survey in selected areas of KwaZulu-Natal Province, South Africa*.**

Mohube Titus Letsoalo[1,2¶], Daniel Nenene Qekwana[3&], Christian. A. Mbajiorgu[1&], Ishmael Festus Jaja[1&], James Wabwire Oguttu[1¶*]

[1]Department of Agriculture and Animal Health, College of Agriculture and Environmental Sciences, University of South Africa, Johannesburg, South Africa

[2]KwaZulu-Natal Department of Agriculture and Rural Development, Durban, KZN, South Africa

[3]Department of Paraclinical Science, Faculty of Veterinary Science, University of Pretoria, Pretoria, South Africa.

*Correspondence

Email: joguttu@unisa.ac.za

Abstract

Knowledge, attitude, and practices (KAP) studies have been widely used to assess gaps towards rabies. However, there is no evidence of baseline studies that have assessed the KAP towards rabies among selected communities in KwaZulu-Natal Province, South Africa. A cross-sectional questionnaire-based study design was adopted for this study. Participants (n=768) were selected using systematic random sampling. Data from respondents (≥18 years) was collected using a pretested questionnaire and analysed using descriptive and inferential statistics. Significance was set at $\alpha \leq 0.05$. Twenty seven percent (26.95%) of the respondents could name the cause of rabies, while just over half (53.52%) of the respondents indicated that they would adhere to post exposure prophylaxis (PEP) which, includes wound washing, a dose of human rabies immune globulin (HRIG), and rabies vaccine given at the time of the first medical visit, and two booster doses . Most respondents (90.10%) mentioned that they would seek medical attention following a dog bite. Only

59.77% of respondents were able to produce a vaccination certificate for their pets. Allowing dogs to roam without supervision was widely reported by the respondents. Dog ownership was the only factor significantly associated with knowledge of rabies. Lack of knowledge of the cause of rabies and the need to receive PEP immediately after exposure to a dog bite was widespread in the study area. However, gaps in some aspects related to knowledge about rabies were observed that require awareness creation for dog owners, including those who do not own pets.

Keywords: Rabies, vaccination, rabies disease, rabies awareness, dog-bite, post-exposure prophylaxis, PEP, Risk factors

1. INTRODUCTION

Domestic dogs play a significant role in the lives of humans. But on the other hand, they pose the greatest threat of transmitting rabies to humans [9]. Rabies is an important infectious viral disease [4], caused by the lyssavirus, from the family of *Rhabdoviridae* [16, 20]. All warm-blooded mammals are susceptible to the rabies virus infection generally transmitted through the bite of a rabid animal [29].

Dog mediated rabies infection remains a significant disease from a public health perspective [33] and annually it is responsible for an estimated 60 000 human deaths worldwide [22, 28]. Of these, Asia has the highest burden of rabies, followed by Africa. Making the two the most affected continents. In Africa, over 90% of human rabies deaths are associated with dog bites [33, 42]. In Asia alone, approximately 31,539 human deaths are reported annually suggesting a high burden of rabies in the continent ([3]. Furthermore, globally, around 15 million people seek rabies post-exposure prophylaxis (PEP) due to the threat of rabies infection following exposure [30].

Once symptoms of rabies develop, victims usually succumb to the disease [2]. This is especially true if the victim fails to obtain PEP early following a dog bite [14]. In humans potentially exposed to the rabies virus, PEP usually includes thorough wound cleaning and a combination of treatment with rabies immunoglobulin and vaccination [44]. Without proper health care, the loss of life of untreated victims is inevitable. Moreover, the disease is reported to have the highest case fatality of any disease [33].

Rabies is a preventable disease, and vaccination of dogs through mass campaigns is one of the effective ways to control the disease at its source [22]. According to the World Health Organization (WHO), a minimum of 70% dog vaccination coverage can drastically reduce the number of human cases within countries where rabies is endemic [26]. However, in most developing countries, rabies continues to be a challenge resulting in high mortalities in humans and animals [25]. This is despite existence of effective human and animal rabies vaccines [33]. This could be attributed to inadequate efforts to control and prevent the spread of the disease in these countries [14].

In South Africa, an estimated 40 human rabies related deaths from dog bite contacts occur yearly [44]. This points to the fact that measures needed to successfully prevent and control the spread of rabies within the dog population are inadequate. This is especially true in the province of KwaZulu-Natal (KZN), which has the highest cases of dog rabies in the country [23]. In addition, rabies is well established and remains endemic in the province [34]. This is supported by the recent increase in the number of laboratory confirmed positive animal cases in KZN [44]. Moreover, most of such cases of rabies have been confirmed in dogs [23]. Besides KZN Province, the Eastern Cape and Limpopo provinces are also considered by the Department of

Agriculture, Land Reform and Rural Development (DALLRD) as having high numbers of rabies.

Knowledge, attitude and practices studies are significant in obtaining information on the subject of concern [32]. Therefore, questions on the cause, source and mode of transmission, clinical signs and prevention practices and treatment measures are important in determining participants' knowledge, attitude and practices (KAP) regarding rabies [21]. In addition, knowledge of how to handle a suspected rabid dog is important [10], and measures that must be adopted when bitten by a rabid dog, need to be assessed if measures to prevent and control rabies are to be effective [45].

In fact, good knowledge, attitude and practices about rabies have been shown to reduce the spread of the disease and the fatalities associated with the disease [16]. In view of this, the present study investigated the KAP of communities in two areas (Embo and Verulam) of KwaZulu-Natal Province of South Africa.

2. METHODS

2.1 Study area

The study was conducted within eThekwini District in KwaZulu-Natal Province of South Africa. Kwazulu-Natal is one of the nine provinces in South Africa and is a multi-racial community. The population of KZN is approximately 10,819,130 living on an area of 94,361 km². Its annual population growth is estimated to be 1, 2 % [24].

The eThekwini District is occupied by about 3.7 million people on a land division of approximately 2,555 km² [15]. Embo (Latitude: 29° 54' 34'' S, Longitude: 30° 40' 29'' E) is a rural area situated in the inlands of eThekwini in the western region of the district, with an estimated population size of approximately 40,924 people, and an estimated 8,184 households. Verulam (Latitude: -29° 38' 59.99'' S; Longitude: 31° 02'

60.00" E) on the other hand is an urban area located in the upper part of the northern region [15]. Verulam has an estimated 64,950 households (Unpublished data, Verulam Environmental Health Manager), and approximately 184,114 occupants on a land of 18.13 km² ([15].

2.2 Study design

A cross-sectional study design was adopted to investigate the knowledge, attitude and practices of community members towards rabies in eThekwini District in KZN province of South Africa.

2.3 Target population

The target population for this study was the residents of Embo (a rural area) and Verulam (an urban area). The study included people of different age groups, religious beliefs, and educational backgrounds. People of both sex (male or female) and both pet owners and non-pet owners were also included in the study.

2.4 Sampling strategy

A systematic random sampling method was used to sample from the two selected areas (Embo and Verulam). In both areas, a sampling interval was predetermined before the selection of households commenced. The first household was selected randomly, thereafter, the successive households were selected following the adopted interval for each of the areas until the number required was reached. However, if an adult was absent at the time of interviewing, the next house with an adult (≥18 years) was chosen to be interviewed [40].

2.5 Sample size determination

This sample size from the two study areas (Embo and Verulam) was determined using the formula described by Gaudu et al. [21] :

$$N = \frac{1.96^2 \times Pexp(1 - Pexp)}{(d)^2}$$

N= Sample size

P_{exp}- Estimate or expected proportion of knowledge about rabies from community was considered at 50%, given that there is no baseline study regarding KAP in the study area.

d^2- Desired absolute precision (5%).

A total of 768 respondents (384 from each of the two areas) were recruited and agreed to voluntarily participate in the study.

2.6 Data collection

The questionnaire developed by Fenelon et al. [17] was adapted for this study. Data was collected using a structured questionnaire during face-to-face interviews. The questionnaire had the following sections: respondents' demographic data, Knowledge of rabies, Attitude towards rabies and Practices.

The questionnaire was translated into the local language (isiZulu) that is most widely spoken to cater for respondents who could not communicate in English. In addition, the questionnaire was pre-tested on fifteen participants from each area to test its fitness for purpose. Each interview session lasted 30-45 minutes and was conducted in English or IsiZulu. Administration of the questionnaire took place from 01/08/2019 to 31/03/2021.

2.7 Data management and data analysis

2.7.1 Data management

Each questionnaire was reviewed carefully after each data collection session to ensure that it was filled in correctly, and afterwards each question was coded. All raw data was immediately captured into the computer using Microsoft Excel 365

(Microsoft Corp., Redmond 137, USA). The correct response to each of the questions on knowledge was awarded a score of 1, while 0 was given for the incorrect answer. Thereafter, the total score for each respondent was computed and presented as a percentage (knowledge score). A binary variable was then created from the knowledge score (knowledgeable=1 and not knowledgeable=0).

2.7.2 Data analysis

Data was analysed using the Statistical Package for Social Science (SPSS) Version 27 (SPSS for Windows, version 27, SPSS, Inc, Chicago, Illinois, the USA). Descriptive statistics such as frequencies and percentages of all categorical variables were computed and presented in graphs and tables. Inferential analysis involved first fitting a series of univariate binary logistic regression models to test for simple associations between the predictor variables and the outcome variable (Knowledge). Variables with alpha level ≤ 0.20 in the univariable model were included in the multivariable model. Significance in the multivariable model was assessed at $p \leq$ ~~>~~ 0.05.

A multivariable logistic regression model was then fit to the data to assess the factors that were significantly associated with being knowledgeable about rabies. Using the backward selection method, variables to retain in the final regression model were identified. If removal of a variable from the model resulted in a ≥20% change in the effects measure of any of the variables in the model, the variable was considered a confounder and was thus retained in the final effects model, regardless of whether it was significantly associated with the outcome variable or not.

Models were compared using the Log likelihood ratio test. The model fit of the final effects model was tested using a Hosmer-Lemeshow test. Based on the results, there was a good model fit ($p > 0.05$).

3. Ethical consideration

The State Veterinary services of KwaZulu-Natal Department of Agriculture and rural development granted approval to conduct the study. In addition, permission to carry out the study was granted by the Ethics committee of the College of Agriculture and Environmental Science, University of South Africa (REC Reference #: 2019/CAES_HREC/105). The identity of the respondents was protected by ensuring that they remained anonymous. The objectives of the study were first explained to the respondents, and thereafter each participant signed the consent form to indicate that they voluntarily accepted to participate in the study. All respondents had the right to withdraw from the study at any time during the study. Findings of this study will be disseminated to the residents of the study areas during rabies vaccination campaigns and presented at conferences to the broader scientific community and researchers.

4. RESULTS

4.1 Descriptive statistics

4.1.1 Demographic profile of respondents

An equal number of respondents (384) was drawn from each of the identified areas (Embo and Verulam). Therefore, a total of 768 community members were invited to participate in the study.

Majority of the respondents were females (63.54%), aged between 18 – 35 years (53.21%), belonged to the Christian faith (47.66%), and had not completed secondary education (35.81%). There was a high pet ownership (72.79%) in the study area (Table 1).

Table 1: **Demographic profile of people who agreed to participate in the study**

Variables	Level	Frequency (n)	Percentage %	95% Confidence Interval	
				Lower interval	Upper interval
Age	18 – 35	409	53.26	49.65	56.83
	36 -53	288	37.50	34.06	41.03
	54 - 71	64	8.33	6.48	10.52
	>71	7	0.91	0.37	1.87
Sex	Male	280	36.46	33.05	39.97
	Female	488	63.54	60.03	66.95
Residence	Verulam	384	50.00	46.40	53.60
	Embo	384	50.00	46.40	53.60
Own a pet dog and/ or cat	Yes	559	72.79	69.49	75.91
	No	209	27.21	24.09	30.51
Religion	Christian	366	47.66	44.07	51.26
	African religion	140	18.23	15.56	21.15
	Hindu	204	26.56	23.47	29.84
	Muslim	31	4.04	2.76	5.68
	Other	27	3.52	2.33	5.07
Education	No education	43	5.60	4.08	7.47
	Completed primary	101	13.15	10.84	15.75
	Secondary not completed	275	35.81	32.41	39.31
	Completed matric/High school Education	234	30.47	27.23	33.86
	Tertiary completed	115	14.97	12.52	17.70

4.1.2 Knowledge of rabies disease

Results of the assessment of knowledge of rabies are presented in Table 2 and Table 3. Half (50.52%; n= 388) of respondents indicated that they did not know the cause of rabies, with only 26.95% (n=207) of the participants indicating that rabies is caused by a virus. Although in the minority, it was interesting to note that some respondents (2.47%, n=19) thought that rabies is caused by a chemical substance.

Table 2: The results of the assessment of the knowledge of rabies among respondents

Variables	Level	Frequency (n)	Percentage %
What is the cause of rabies?	Chemical substances	19	2.47
	Virus	207	26.95
	Insufficient intake of feed and water	77	10.03
	Psychological problem	77	10.03
	Don't know	388	50.52

How can human be infected with rabies?	Dog-bite	668	86.98
	Playing with the dog	15	1.95
	Feeding the dog	0	0
	Don’t know	85	11.07
What is the first thing to be done after dog-bite while at home?	Cover the wound with bandage	96	12.50
	Cleanse the wound with soap and running water	411	53.52
	Apply any topical medication	134	17.45
	Don’t know	127	16.54
Can rabies be treated beyond appearance of clinical signs?	Yes	387	50.39
	No	123	16.02
	Don’t know	258	33.59
When is it appropriate to receive anti-rabies injection after being bitten by a dog suspected of having rabies?	Later after weeks	27	3.52
	Immediately	601	78.26
	Any time after months	41	5.34
	Don’t know	99	12.89
Is vaccination of pets important for preventing human rabies?	Yes	611	79.56
	No	13	1.69
	Don’t know	144	18.75

Dog-bite was identified by most respondents (86.98%, n=668) as the likely rout for the transmission of rabies. In response to the question on the first thing to be done after a dog-bite while at home, slightly over half of the respondents (53.52%, n=411) indicated that they would clean the wound with soap and running water (Table 2).

Although 50.39% (n=387) of respondents were not aware that rabies can be fatal beyond appearance of clinical signs. It was also noted that most respondents (78.26%, n=601) would immediately seek the provision of anti-rabies injections following a bite by a suspect rabid animal. However, 12.89% (n=99) did not know that they should get anti-rabies injections immediately, while 5.34% (n=41) indicated that it could be given later; say after weeks or even months later (3.52%, n=27).

Majority of respondents indicated that human clinics or hospitals (87.24%, n=670) are the places they can obtain anti-rabies injection in case they were bitten by a suspected rabid dog. A few indicated the veterinary clinic or veterinary hospital (5.08%, n=39) or supermarkets (1.30%, n=10) as possible sources where anti-rabies injections could be obtained following a dog bite.

The results show that 79.56% (n=611) of the respondents were aware that vaccination of pets is important for controlling rabies among humans. This was followed by 18.75% (n=144) who did not know that vaccination of pets is important to prevent humans contracting the disease. Meanwhile, a very small percentage (1.69% (n=13) indicated that vaccination of pets was not important for controlling rabies in humans.

Although a high number of respondents (83.33%, n=640) were aware that rabies could be transmitted to humans from animals, up to 12.76% (n=98) of the study population, expressed ignorance about rabies being transmitted from animals to humans. In addition, some respondents said that rabies could not be transmitted from animals to humans. However, these were very few (3.91%, n=30) (Table 3).

Most respondents (79.04%, n=607) were aware the need for someone to seek medical attention after a bite from a suspected rabid dog. However, the number of positive responses slightly dropped to 71.74% (n=551) when respondents were asked if vaccination against rabies after being bitten by a suspected rabid dog offers protection against development of rabies (Table 3).

Less than half (42.19%; n=324) of the respondents indicated that a 3-month-old dog was eligible to receive the start-up rabies vaccination. This was followed by 28.65% (n=220) who did not know the age at which puppies can get their start-up rabies vaccination and 15.89% (n=122) who thought that puppies can get their start-up rabies vaccination immediately after birth (Table 3).

Table 3: The results of the assessment of the knowledge of rabies

Knowledge variables	Level	Frequency (n)	Percentage %
At what age do pets (dogs or cats) start to receive their vaccination?	Immediately from birth	122	15.89
	From 3 months	324	42.19
	From 12 months	100	13.02

	Never	2	0.26
	Don’t know	220	28.65
Where can you obtain anti-rabies injection if you are a victim of dog bite?	Veterinary clinic or Hospital	39	5.08
	Any supermarket	10	1.30
	Human clinic or Hospital	670	87.24
	Don’t know	49	6.38
Is it possible for rabies to be transmitted from animals to humans?	Yes	640	83.33
	No	30	3.91
	Don’t know	98	12.76
Is it advisable for someone to get anti-rabies after a bite from a suspected rabid dog?	Yes	607	79.04
	No	25	3.26
	Don’t know	135	17.58
	Did not answer	1	0.13
If you are vaccinated against rabies after being bitten by a rabid dog, will that protect you from developing rabies?	Yes	551	71.74
	No	44	5.73
	Don’t know	170	22.14
	Did not answer	3	0.39

In response to the question on the species that are “susceptible to rabies?”. Most respondents (92,2%) mentioned dogs. This was followed by 72,53% who indicated that human beings could contract rabies and just over half (57,42%) who mentioned cats were susceptible to rabies (Fig 1).

Fig 1: Distribution of respondents by responses to questions regarding animals susceptible to rabies.

In response to the question on clinical signs that can make them suspect rabies in dogs, most respondents mentioned (74,61%) signs such as change in behaviour, followed by the inability of a dog to drink and eat (20,83%), and salivation (16,28%) (Fig 2).

Fig 2: The percentage of respondents based on how they responded to the question about the clinical signs of rabies in dogs.

4.1.3 Attitude and practices towards the disease rabies

Results of the attitudes and practices towards rabies are summarised below. Majority of respondents (81.77%, n=628) indicated that it was necessary to receive anti-rabies injection after a dog bite. This was followed by 4.97%; n=115) who did not know that it was necessary to receive anti-rabies, and 3.13% (n=24) did not think that it was necessary to receive anti-rabies injection after a dog bite.

With regards to what should be done to a dog that has bitten someone, 52.47% (n=403) were of the view that such animal should be quarantined. This was followed by 23.83% (n=183) who were of the view that noting should be done to the dog and 13.54% (n=104) and 10.03% (n=77) who indicated that the dog should be killed or chased away respectively.

On the question of whether putting-down a dog suspected of having rabies was appropriate, only 39.45% (n=303), agreed that putting down the dog was the appropriate measure in such circumstances. This was followed by 34.51% (n=265) who did not think that putting down a dog in such circumstances was appropriate, and 25.78% (n=198) who did not know if putting down a dog in such circumstances was appropriate (Table 4).

Under thirty percent (27.21%; n=209) of the study population did not have pets. But among those who owned pets, slightly over half (53.39%, n=410) did not sterilise their pets.

In response to the question how often their dogs get vaccinated 75.91% (n=583) vaccinate their dogs at different intervals. This was followed by 19.14% (n=147), who were not aware of the frequency with which their dogs get vaccinated. Some respondents vaccinated their dogs only once in its lifetime (3.91%; n=30), or never

vaccinate their dogs (1.04%; n=8) (Table 4). Meanwhile, only 59.77% (n=459) of the respondents were able to produce the vaccination certificate as proof that their pets had been vaccinated against rabies.

Sixty-three percent (62.50%; n=408) of the respondents were of the view that vaccination of pets (dog or cat) was important even if the pet is always restrained. In contrast, 10.29% (n=79) did not think it was worthwhile vaccinating their dogs if they are always restraint.

With regards to allowing dogs going out of the yard unsupervised, 43.23% (n=332) did not allow their dogs to go out of the yard unsupervised, while 43.23% (n=332) indicated that they let their dogs go out of the yard unsupervised. But regarding keeping dogs in a fenced place, just over half (58.07%; n=446) indicated that they kept their dogs in a fenced premises to prevent the dogs from going outside unsupervised. The majority (64.32%, n=494) of respondents indicated that they always vaccinated their pets during annual vaccination campaigns, while 8.46% (n=65), indicated that they did not always take their dogs for vaccination during the rabies vaccination campaigns (Table 4).

As indicated in Table 4 below, only 17.84% (n=137) respondents were aware that only laboratory results can be used to declare the animal positive for rabies. However, a higher number (35.42%; n=272) indicated that animals that bite people after confrontation, should be presumed to be rabid. In response to the question what to do when a suspect rabies stray dog is encountered, just over half (53.52%; n=411) said that they would report the dog as a rabies suspect to the department of Agriculture/ or SPCA.

Table 4: Results of the attitude and practices among respondents towards rabies disease

Variables	Levels	Frequency (n)	Percentage %
Do you think it is necessary to receive anti-rabies injection after a dog bite?	Yes	628	81.77
	No	24	3.13
	Don’t know	115	14.97
	Did not answer	1	0.13
What should someone who has been bitten by a suspect rabid dog do following a bite?	Nothing	30	3.91
	Leave the wound to heal	20	2.60
	Seek medical attention	692	90.10
	Purchase medication for the wound	25	3.26
	Did not answer	1	0.13
What should be done to dog that has bitten someone?	Nothing	183	23.83
	Kill the dog	104	13.54
	Chase the dog away	77	10.03
	Quarantine the dog	403	52.47
	Did not answer	1	0.13
Do you think it is appropriate to put-down the dog if it is suspected to have rabies?	Yes	303	39.45
	No	265	34.51
	Don’t know	198	25.78
	Did not answer	2	0.26
Did you sterilise your pet?	Yes	149	19.40
	No	410	53.39
	Don’t have	209	27.21
Do you have a copy of certificate of vaccination for your pet, to produce?	Yes	459	59.77
	No	100	13.02
	Don’t have a pet	209	27.21
How often can you vaccinate your dog?	Only once in a lifetime	30	3.91
	Never	8	1.04
	Different interval	583	75.91
	Don’t know	147	19.14
Do you keep your dog in a fenced place?	Yes	446	58.07
	No	113	14.71
	Don’t have	209	27.21
Do you ever allow your dog to go out of the yard unsupervised?	Yes	227	29.56
	No	332	43.23
	Don’t have	209	27.21
Do you always vaccinate your dog when there are annual vaccination campaigns?	Yes	494	64.32
	No	65	8.46
	Don’t have a pet	209	27.21
Do you vaccinate your dog even though it is always restrained?	Yes	480	62.50
	No	79	10.29
	Don’t have	209	27.21
How do you know if an animals has rabies?	When the animal is lazy	102	13.28
	You only know by laboratory results	137	17.84
	Wait until it suddenly dies	13	1.69
	If it bites people when confronted	272	35.42
	Don’t know	244	31.77
What do you do when you see a suspect rabies stray dog?	Nothing	227	29.56
	Kill the dog and throw or bury	80	10.42
	Chase the dog away	50	6.51
	Report to Department of Agriculture/ SPCA)	411	53.52

Respondents were asked a question on how they keep safe from contracting rabies. Majority of respondents (81,77%) indicated they always vaccinate their animals to avoid rabies. Slightly over half (50,26%) of the respondents indicated that they would stay away from stray animals to avoid contracting rabies. Only 12,11% did not know what should be done to remain safe from rabies (Fig 3).

Figure 3: A bar graph showing the proportion of respondents based on their attitude towards rabies in eThekwini District.

4.2 Inferential statistics

Age, owning a pet, religion and educational level were the only variables that were significantly associated with the outcome in the univariable analysis at a generous P-value of ≤ 0.2. These were thus included in the multivariable logistic regression model to identify the factors that were significantly associated with being knowledgeable about rabies.

Only two variables (owning a pet and age of the respondents) were retained in the final multivariable logistic regression model. The respondents who owned pets compared to those who did not, were 3 times (AOR = 2.788; p-value=0.0001) as likely to be knowledgeable about rabies compared to those who did not own pets (reference group) (Table 5).

Age was not a significant (p-value >0.05) predictor of knowledge among the respondents. However, it was retained in the final effects model because it was a confounder.

Table 5: The results of multivariable analysis showing factors that were associated with being knowledge about rabies

Variable	Coefficient estimate	Standard error	Adjusted Odds Ratio	95 % CI		P-value
**Own Pet?**				Lower	Upper	
No	**Ref**					
Yes	1.0254	0.2570	2.788	1.721	4.737	0.0001
**Age**						
>71	**Ref**					
18-35	-0.0706	1.1034	0.932	0.149	18.009	0.9490
36-53	0.5991	1.1030	1.821	0.291	35.170	0.5870
54-71	0.2741	1.1388	1.315	0.191	26.364	0.8098

5. DISCUSSION

This is the first study to investigate the knowledge, attitude, and practices toward rabies disease among respondents in the study area. Few respondents were aware that rabies is caused by a virus. Dog bites was identified by a large proportion of the respondents as the main transmission mode for rabies to humans. But on the other hand, just over half of the respondents interviewed mentioned that washing of the wound following a dog bite with soap and running water was important for preventing rabies disease. Very few respondents were aware that licking of an open wound by a suspected rabid dog is one of ways in which rabies spreads from dogs to humans. Similarly, a very low number of respondents in the present study indicated that they knew that rabies disease was fatal. However, many respondents were aware of the importance of receiving anti-rabies vaccine immediately after being bitten by a dog suspected of having rabies to protect the bite victims from developing rabies. Only pet ownership was significantly associated with a high knowledge score for rabies.

5.1 Demographic profile of respondents

This study found that over half of respondents (53.26%) were 18-35 years of age. This the age group that is likely to be more active when it comes to pet management in the homes. Given that more females (63.54%) agreed to participate in the study compared to males, it is possible that females take more keen interest in matters that relate to their pets. However, this observation contrasts with findings by

Bihon et al., [6] and Abdela and Teshome [2], who observed that male respondents constituted 71.4% and 59.3% of the study population respectively.

The findings of the present study suggest a high dog/pet ownership in the study area. For example, in comparison to the 72.79% who owned dogs in the present study, only 53% of the respondents owned dogs in a study done in Shirsuphal in western India [39]. Findings from the present study also contrast with those from a study conducted in Cameroon in which less than half of the respondents owned pets [9].

In this study, just below half (47.66%) of respondents were Christians, contrary to findings of studies conducted in Kombolcha and Dedo districts which reported Muslims proportion of 56.8% and 94.8% respectively [1, 20]. The observed difference could be attributed to differences in the dominant religions in the different study areas.

The number of people who had not completed secondary education (35.81%) or had completed matric (high school) (30.47%) in the study population was lower than the number of people who had formal education. However, this is much higher than what Abdela et al. [1] reported. The later observed that half of their study population (51.1%) did not have a formal education. Similarly, the number of people with education higher than primary schooling in the current study was higher than the 52,5% reported in the survey done in Berhampur, Odisha that had only primary school level education [41]. The high number of people who could read and write in the present study is an encouraging finding because it points to a target population that is amiable to training given that a lot of training material on rabies is in English.

5.2 Knowledge of the disease rabies

The very low number (26.95%; n=207) of respondents who were aware that a virus was the agent responsible for rabies, is in line with results from the study

conducted in Dessie City, where only 18% (n=25) of respondents regarded a virus to be the cause of rabies [19]. However, in a study conducted in Kombolcha, Ethiopia, a higher number (76.6%; n=294) of respondents were aware that rabies is caused by a virus [20]. This difference suggests a need to improve on the training of residents in the study area and particularly make it clear what causes rabies.

A very high number of respondents (86.98%) who mentioned dog bite as a mode of transmission for rabies to humans is an encouraging finding from a public health point of view. This aligns with findings from a study by Fesseha et al. [18], which reported almost a similar proportion (85%) of respondents who mentioned that rabies virus was transferred through a bite from an infected animal. However, a slightly lower number from the Philippines (79.5%) than those observed in the present study, were aware that dog bites play a crucial role in the transfer of rabies to people [5]. Similarly, a study conducted in Haiti also observed that 75.6% of respondents knew that rabies can be transmitted via a dog bite [17]. The findings of this study shows that the knowledge about the transmission of rabies in the study is comparable to that observed in other developing countries such as Ethiopia and the Philippines. On the other hand, results of this study indicate more people than what has been reported in other countries were knowledgeable about the transmission of rabies.

Licking of an open wound by a dog as mode of transmitting rabies, was mentioned by a mere 11.46% of the respondents in the present study. Similarly, a very low (1.8%) number of community members in a study conducted in Haiti were aware that rabies could be transmitted to people through contact of saliva with an open wound [17].

While most respondents mentioned biting as one of the ways the virus gains entry to the victim, licking of the open wound by the suspected animal was not

considered important in the spread of rabies. This suggest that if the dog licked humans in the study area, they would probably not attempt to seek medical care. This would hence put them at risk of rabies infection.

Just over half (53.52%) of this study's respondents considered washing the wound with soap and running water as the first aid to prevent rabies. This low number, is of concern, given the importance of post exposure prophylaxis that includes washing the wound immediately with soap and water to prevent infection gaining entry to the victim. In contrast, a higher number (92.4%), of respondents from the study in and around South Gondor in Northwest Ethiopia indicated that washing the wound with soap was the first measure to be implement following a bite by a suspected rabid dog before seeking other preventative measures [20]. Findings reported here also contrasted with those reported by Bihon et al [6], who observed that a very low number of respondents (8.9%) regarded wound washing as an immediate response to prevent rabies. In view of the findings of the present study, while the number of people who acknowledge that washing the bite wound was important as the first step in the prevention of rabies is higher than what Bihon et al. [6] observed in South Gondor. Therefore, there is room for improvement on understanding how to prevent rabies in case of a bite among residents in the study area.

In this study, only 16% of respondents considered rabies a fatal disease. This figure is much lower than the findings from Bihon et al [6] and Abedela and Teshome [2], who observed that 84.6% and 82% of people, respectively, were aware that rabies cannot be cured once clinical signs appear. The fact that only a few respondents in this study were aware that rabies is not treatable, is significant and suggests people are most likely not to seek medical care early. It is critical that this kind of information

be included in campaigns to educate the community about the seriousness of the disease rabies.

A considerable number of respondents (78%) in the present study knew that they need to obtain anti-rabies vaccine immediately after being bitten by dog suspected of having rabies. A slightly lower response (65.2%) was reported in Dedo district of Jimma zone, Ethiopia [1]. Even a much lower number was reported by Abdela and Teshome [2] who observed that only 32.7% of the respondents in their study indicated that anti-rabies should be administered immediately after exposure. Although a much higher number of respondents in this study were aware of the importance of receiving anti-rabies vaccines immediately after being bitten by a suspect dog, there is room for improvement to bring the number closer to 100%. The authors believe that everyone should know that receiving anti-rabies treatment immediately after one has been bitten by a dog suspected of having rabies is necessary to eradicate rabies related deaths. The difference observed between the different studies suggest a difference in the effectiveness of extension services in the different study areas. It is evident from the findings reported here that there is room for improvement in the study area when it comes to the importance of receiving anti-rabies vaccine immediately following a dog bite.

Lack of knowledge among the general public on the role other species besides dogs play in the spread of rabies, seems to be widespread. For example, although in this study a large number (90%) of community members mentioned dogs as being susceptible to rabies, other animal species were mentioned less frequently. Similarly, a high proportion of respondents in other studies conducted in Kansas (96.8%) and Gondar Zuria District (100%), largely pointed to dogs as being the one that is susceptible to rabies [11, 27]. Only a study conducted in Harare Metropolitan reported

few respondents (37.59%) who identified dogs as the only animal susceptible to rabies [36]. The widespread lack of knowledge of the role played by other species besides dogs in the spread of rabies, points to a gap in the education programmes communities are exposed to. Therefore, there is a need to place emphasis on the role other species play in the spread of rabies during extension to communities.

The over three quarters (79.75%) of the respondents in this study who were aware that vaccinating pets was significant to keep humans safe from rabies, is consistent with findings of other studies. For example, most respondents (68%) from Jamma also believed that vaccination against rabies in dogs helps prevent the disease in humans [38]. Dubey et al. [12] reported a slightly higher proportion of respondents who believed that dog vaccination was a good practice in a study conducted in Jabalpur central India. The high number of respondents who were aware of the importance of vaccinating dogs to control rabies is good news. This is because vaccination of pets creates a barrier of diseases shared among pets and humans given that they occupy the same space.

The slightly less than half (42.19%) of the respondents in the present study who favoured vaccinating their pets at 3 months old, was a bit higher than the finding of a study conducted in Kigali city, Rwanda, were only 20.6% of respondents vaccinated their puppies by the age 3 months [31]. On the other hand, Bouaddi et al. [7] reported a much lower percentage of respondents (14.7%) who favoured having their dogs vaccinated at 3 months of age. However, in the Democratic Republic of Congo up to 14% of respondents believed that young puppies should not be vaccinated [27]. The differences observed in studies conducted elsewhere and the present study, the higher figures observed in this study, could be attributed to the rigour with which rabies eradication campaigns are conducted in the study area. In fact, in South Africa, rabies

campaigns target puppies from 3 months of age and older as part of the eradication scheme aimed at eliminating rabies within the country.

The very high number of respondents (87.24%) in this study who preferred to be treated from a human clinic or hospital after exposure to a dog bite, is a welcome observation. It is important for people to know where they can be treated following exposure for effective rabies prevention. This result is consistent with what Edukugho et al. [14] reported. The latter observed that more than 86% of respondents would report to the hospital or see a doctor after being bitten by dogs. However, a report by Fesseha et al. [18], observed that only 55.6% of respondents would seek medical attention after a dog bite. This low number of people who would seek medical care reported by Fesseha et al. [18] in comparison to what was observed in the present study, suggests that the education of the public regarding how to treat exposed people is very impactful in the study area.

Although up to 83.33% of the respondents in this study knew that rabies can be transmitted from animals to humans, the number that did not know, even though small, cannot be ignored. However, these findings agree with those of studies done in Munesa district and Tigray regions of Ethiopia, in which 88.7% and 89.5%, respectively, indicated that humans were likely to contract rabies from animals [2,13].

The high number of respondents (79.04%) in the present study who were aware of the importance of obtaining anti-rabies vaccination following a bite from a suspected rabid dog, contrasts with findings from Odisha, India [41] and Ethiopia [11], which reported low numbers of respondents (14% and 38.8% respectively) who were aware of the importance of receiving anti-rabies vaccination after being bitten by a dog. This could be attributed to differences in the proximity to health care facilities in the different study areas. In instances where health facilities are readily accessed, although some

of the bite victims may go to a health facility with the intention of getting anti-rabies, they also end up receiving wound treatment. This could contribute to the high numbers seeking medical care observed in some studies as opposed to others.

The 71.74% of the respondents in the present study that were aware that if vaccinated against rabies following a bite by a rabid dog, a person is protected from developing rabies, is lower than the 87% of respondents from a study conducted in Abuja, Nigeria, that had visited health care facilities to obtain treatment following a dog bite [14]. Nonetheless, the high numbers of people in this study, who were aware that receiving the anti-rabies vaccine is important in preventing the disease in humans, further points to the effectiveness of the education campaign in the study area. This contrasts with finds of another study conducted in the Tigray region of Ethiopia, in which just over half (54.9%) of respondents preferred to go to traditional healers instead of hospitals following a dog bite incident [13].

It was encouraging to observe that majority of the respondents (74.61%) in this study were aware that a change in behaviour including dogs biting people were major clinical sign of rabies. This is in line with the findings reported in Dedo district, Southwest Ethiopia [1], Gondor Zuria, Ethiopia [11] and Gondar, Northwest [6], where up to 50.4%, 63.5% and 75.8% of the respondents respectively, knew that behaviour change is one of the signs of rabies.

On the other hand, paralysis as a clinical sign of rabies did not feature predominantly. Therefore, paralysis was likely to be overlooked as a sign of rabies by the study population. This is worth noting because, when such people get bitten by a rabid dog even if it showed paralytic signs, they are likely not to seek post exposure prophylaxis.

5.3 Attitude and practices of community members towards rabies

The considerable proportion of respondents (81.77%) in this study who showed a willingness to receive anti-rabies vaccine to help prevent the disease in case of dog bite, is a positive finding. This implies that people are willing to obtain anti-rabies treatment as soon as possible after exposure to prevent rabies, given that currently there is no available treatment once the disease has developed. The number of people willing to received anti-rabies in the current study was higher than 51% from Berhampur, Odisha, in India, that reported were willing to obtain anti-rabies as a control measure rabies following a dog bite, a country that is also endemic for rabies, and accounts for 36% of the world's rabies deaths [41]. An even lower percentage (38.8%) of people from Gondar Zuria District, Ethiopia, were willing to obtain anti-rabies in response to dog bite [11]. Just like India and South Africa, Ethiopia is endemic to rabies. The difference observed with respect to the number of people with a positive attitude towards receiving anti-rabies vaccine in the different countries that are endemic to rabies, could be attributed to the nature of education programmes implemented in the different countries. It could also be attributed to the differences in the level of education in the different study areas.

The overwhelming majority of respondents (90.10%) in this study were willing to seek medical attention following exposure to a dog bite victim. This is consistent with results of a survey done in Kombolcha, Ethiopia where 89.3% of respondents would seek medical care if they were bitten by an animal suspected to have rabies [20]. However, results from the present study widely contrasted with findings of a survey conducted in and around South Gondar, Ethiopia, in which it was observed that 79.4% of respondents were not in favor of visiting health care centers following dog bites [6].

The 52.47% of respondents who indicated that they would allow their dogs to be quarantined for observation after biting someone, contrasts with 69% of the respondents in a study from Kigali City, Rwanda, who were not in favor of isolating their animals if they bit people [31]. However, the number of people willing to have their dogs quarantined in the present study were far higher than those reported in a study conducted in Cameroon, where only 2.6% favored confining their dogs for observation when they start behaving unusually, including biting people [9].

While a low percentage of respondents (39.45%) in the present study that indicated that they would put down their dogs if they were suspected to be rabid, many more, either did not think that putting down the dog suspected of rabies was appropriate or did not know what to do with such animals (60,29%. This sharply contrasts with results of studies conducted in South Gondar and Kombolcha where 64.3% and 84.1%, respectively, indicated that they would kill the dog suspected to have rabies [6,20]. The low numbers of people willing to have their animals put down if they are suspected to be rabid in this study, could be influenced by the belief that rabies can be treated. It could also be influenced by religious belief among certain ethnic or religious groups that an animal should die naturally without human interference.

The sterilization rate in the study area was low with just over half (53.39%) of the respondents in the study population having not sterilised their pets. The low sterilization rate of pets suggests that many dogs in the study area can breed. The problem with this, is the potential to increase the risk of rabies due to the resultant high number of dogs in the study area. The larger the population of dogs in the area, the more dogs are likely to roam freely, especially during mating season when they want to reproduce. This is why, dog population control is likely to curtail the spread of rabies.

The number of people willing to sterilise their pets in the present study contrasts with the numbers reported by a study conducted in Sri Lanka by Ubeyratne et al. [43], who reported that up to 86.41% had not sterilised their pets. But findings of the present study are consistent with those of a study conducted in El Jadida, Morocco, that reported that just under half (45%) of respondents did not believe in the practice of sterilizing pets [7].

A copy of the rabies vaccination certificate was requested from respondents as a confirmation of pets in the study areas having been vaccinated. A vaccination certificate was recorded as valid if the pet had received the rabies vaccine the previous year or in the same year the study was conducted. Only 58.77% of the respondents in the present study were in position of a vaccination certificate as proof of vaccination of their pets. This finding is suggests that the vaccination coverage in the study area is below the 70% vaccination coverage recommended by the World Health Organization (WHO) [7] to prevent rabies outbreaks in humans. This finding is a serious public health concern because it shows that the area is susceptible to experiencing a rabies outbreak. However, findings reported here, contrast with reports of studies conducted elsewhere. For example, in the El Jadida Region, Morocco [7], it was observed that 76.6% of the respondents had rabies vaccination certificate for their pets. Ntampaka et al. [31], observed that the majority (81.8%) of respondents in their study had vaccinated their animals to prevent them from getting rabies and 81.2% of the respondents from Southern Wollo, Ethiopia indicated that vaccination was significant to maintain a society free from rabies [20].

However, a low vaccination coverage, has also been observed in other parts of the continent. For example, Costa et al. [9], reported a low vaccination coverage (30.8%) of pets in their study conducted in Cameroon. It is evident that the study area

is doing better than other areas parts of other countries. But that notwithstanding, there is room for improvement in the study area to ensure that the vaccination coverage is at the level recommended by the WHO.

The 75.91% of respondents in this study that showed a willingness to vaccinate their pets following guidelines provided by the State to vaccinate dogs, is higher than the findings reported in El Jadida, Morocco, where only 67.8% of the respondents appreciated annual dog vaccination [7]. An even a lower response was observed among respondents in Northwest Ethiopia, where only 28.1% knew that dogs should be vaccinated repeatedly to prevent rabies [6]. Adhering to repeated and regular rabies vaccination helps boost the immune level of pets and thus minimize outbreaks of rabies.

The 50.26% in this study who mentioned that to avoid getting rabies from animals, one must avoid any contact with stray dogs, sharply contrasts with the 81.77% who advocated for animals getting vaccinated. The 50.26% in the present study also contrasts with only 33.3% of respondents in Kansas who expressed the view that avoiding contact with animals one is not familiar with, is key to preventing rabies [37].

Over half (58.07%) of respondents in the present study, kept their dogs behind a fence, which is far less than the number of respondents (84%) in Shirsuphal who kept their dogs in a fenced place to limit them from going outside the households [39]. On the other hand, a lower percentage of respondents (25%) in Filipinos kept their animals in an enclosed environment to prevent them from roaming freely [10]. Results reported here contrast with those in Bobo Dioulasso, Burkina Faso, where up to 85% of the participants kept their animals in closed places to prevent them from roaming in the streets [35]. Keeping dogs from contact with other stray animals limits the chance

of rabies infection. Therefore, the lack of fences around homes with dogs increases the contact of animals which may lead to the rapid spread of rabies. Thus, confinement of dogs is very important and needs to be improved on in the study area.

Over one quarter (29.56%) of respondents in the present study indicated that they occasionally allowed their dogs to move freely out of the yard unsupervised. However, this figure is lower than what was reported by Costa et al. [9] and Corfmat et al. [8], who reported a significantly higher percentage of respondents (61% and 59%, respectively), who indicated that they occasionally allowed their dogs to move out of the yard freely. Dogs that move freely risk contracting the rabies virus from other infected animals.

Just as was observed that 64% of the respondents in the Filipinos study consistently vaccinated their dogs to prevent rabies [10], up to 64.32% of respondents in the current study regularly presented their dogs for vaccination during vaccination campaigns. Similarly, a study conducted in south Gondar also reported that 69.8% of respondents showed commitment to dog vaccination [6]. However, although the number of people who consistently vaccinated their dogs in the present study was high, it was below the recommended 70% vaccination coverage by WHO [7]. The low number could be because some people are not available during the campaigns due to work or other commitments, while others are unable to handle their dogs and end up not presenting their dogs for vaccination. It is also possible that the high number of dogs that are not confined that was observed in this study explains the low numbers of people who consistently present their dogs for vaccination. This is because such dogs are likely not to be available to be immunized during the time of the vaccination campaign.

It was encouraging to note that 62.50% of respondents in the present study indicated that dog vaccination is necessary even for dogs that are kept in closed yards all the time. This finding has potential to boost vaccination as a tool to drive elimination off dog-mediated human rabies.

A very lower response (17.84%) was noted with respect to submitting the carcass of suspected animals to the laboratory to confirm if it had rabies. The results of the study conducted in the Democratic Republic of the Congo about submitting carcasses for confirmation of rabies, was worse, with only 2% of respondents indicating that they would take the carcass of a suspected rabid animal to veterinary services for laboratory testing [27]. Effort is needed to increase awareness of the need to submit carcasses of animals that have died for laboratory confirmation. This has the potential to assist in early outbreak detection and improving surveillance efforts.

A mere 53.52% of the respondents in the present study who would report animals suspected of being rabies positive to the authorities, contrasts with a higher percentage (73%) of respondents from Shirsuphal village in western India, who would report the presence of rabid dogs to the municipal authorities if they ever encountered a case of rabies [40]. However, in Debark Worenda, Ethiopia 84.1% of the respondents said that they would kill the animal if they suspected it had rabies [45]. In the study by Mbilo et al [27], only 20% of respondents indicated that they would alert the veterinary services if they recognized a suspected rabid animal. This completely contrasts with the findings of the present study, and this difference could be attributed to the impact of the rabies eradication campaigns in the different areas. Behavioral change in a suspected rabid animal was reported by a higher number of respondents, while other signs of rabies were mentioned less. These findings are of public health importance in that respondents are likely to report to the authorities when they observe

behavior change, which is an important sign of rabies. However, studies show that aggressive animals are likely to become tame when rabid. As a result, it might be difficult to identify when the animal is a stray.

5.4 Factors significantly associated with knowledge

The finding that respondents who owned pets had significantly higher odds (AOR: 2.788; P=0.0001) of being knowledgeable about rabies in comparison to respondents who did not own pets, is consistent with the findings by Abdela et al. [1]. The later also observed that respondents who owned dogs had significantly (P=0.029) higher odds of obtaining a good KAP score than those who did not own dogs. Similar results were observed among residents of Abuja municipal area, where it was found that people who owned dogs were 7.8 times more likely to have a better knowledge of rabies than those who had no dogs [14]. This is explained by the fact that people who own pets are more likely to receive information during rabies campaigns when they take their dogs for vaccination.

6. CONCLUSION AND RECOMMENDATIONS

There are knowledge gaps with respect to certain aspects such as rabies being a viral disease, how one can protect oneself from contracting rabies, precautionary measures to adopt following exposure to rabies and how to protect susceptible animals from being exposed to rabies. The role of measures such erecting fences around homesteads to restrict animal movements and neutering of pets to control dog population need to be emphasized during extension. The low number of people who had vaccination certificates, although worth noting, needs further investigate to establish whether respondents do not keep certificates, or if it is a true reflection of the status of the vaccination coverage.

The study identified that dog ownership was the only socio-demographic factor that was a significant predictor of knowledge on rabies. In view of this, information dissemination needs to be inclusive to include non-pet owners in the area who also constitute the population at risk. On a positive side, the study demonstrated that there was widespread knowledge on some aspects like knowledge of the role of dog bites in the spreading of rabies in the study area, which suggests that people are likely to seek medical attention in case of a dog bite incidence. In addition, a good attitude towards receiving PEP following a dog bite and reporting a suspected cases were widespread in the study.

To bridge the gaps in knowledge that were identified in this study, implementation of global strategies such as awareness and vaccination campaigns need to be stepped up. Information to sensitize people can be found from government distributed pamphlets or by watching “If Only I Knew” video and by also consulting Government Veterinary Services (GVS). In addition, programs to enforce the positive aspects identified in this study should continuously be implemented. This will help improve the control and prevention efforts directed towards minimizing and eradicating rabies outbreaks including associated human fatalities. A one health approach that involves collaboration between veterinary services with other public health institutions and local authorities to expand and strengthen rabies awareness should be adopted in the study areas to achieve 2030 goal of rabies elimination in rabies endemic areas.

7. Limitations

This study was subject to limitations of cross-sectional surveys such as recall biases. Furthermore, the study was limited to Verulam and Embo in eThekwini District; hence, the results cannot be generalised to other areas in South Africa. The study was conducted during the lockdown due to the COVID-19 pandemic, when movement of

people was restricted and people were reluctant to meet strangers, which could have led to selection bias, which thus calls for caution when interpreting the results. Nonetheless, this study presents baseline information on the knowledge, attitude, and practices towards rabies, which was previously unavailable in the study area. It also provides baseline information upon which future research can be developed.

8. ACKNOWLEGEMENTS

The authors would like to appreciate the University of South Africa for partially funding this study through the Post-graduate Bursary, as well as the participants for consenting to participate in this study, and their cooperation throughout the study. We would like to acknowledge the Department of Agriculture and Rural Development, Veterinary services section, KZN, for permitting us to carry-out the study within eThekwini district. We would also like to extend gratitude to UNISA ethics committee for approving this study to be conducted.

REFERENCES

1. Abdela, N., Midekso, B., Jabir, J. and Abdela, W. (2017) ‘Knowledge, attitudes and practices towards rabies in Dedo district of Jimma zone, southwestern Ethiopia: A community based cross - sectional study’, 9(May), pp. 61–71. Available at: https://doi.org/10.5897/IJMMS2017.1302.
2. Abdela, N. and Teshome, E. (2017) ‘Community based cross-sectional study on knowledge, attitudes and practices towards rabies in Munesa District, Arsi Zone, Southeastern Ethiopia’, *Journal of Public Health and Epidemiology*, 9(6), pp. 161–170. Available at: https://doi.org/10.5897/jphe2017.0931.
3. Ali, A., Ahmed, E.Y. and Sifer, D. (2014) ‘A Study on Knowledge, Attitude and Practice of rabies among residents in Addis Ababa, Ethiopia’, *Ethiopian*

Veterinary Journal, 17(2), p. 19. Available at: https://doi.org/10.4314/evj.v17i2.2.

4. Ba, M.F., Kane, N.M., Diallo, M.K.K., Bassoum, O., Boh, O.K., Mboup, F.Z.M. *et al.* (2021) 'Knowledge, attitudes and practices on rabies among human and animal health professionals in senegal', *Pathogens*, 10(10), pp. 1–15. Available at: https://doi.org/10.3390/pathogens10101282.
5. Barroga, T.R.M., Basitan, I.S., Lobete, T.M., Bernales, R.P., Gordoncillo, M.J.N., Lopez, E.L. *et al.* (2018) 'Community Awareness on Rabies Prevention and Control in Bicol, Philippines: Pre- and Post-Project Implementation', *Tropical Medicine and Infectious Disease*, 3(1), pp. 1–14. Available at: https://doi.org/10.3390/tropicalmed3010016
6. Bihon, A., Meresa, D. and Tesfaw, A. (2020) 'Rabies: Knowledge, Attitude and Practices in and Around South Gondar, North West Ethiopia', *Diseases*, 8(1), p. 5. Available at: https://doi.org/10.3390/diseases8010005
7. Bouaddi, K., Bitar, A., Bouslikhane, M., Ferssiwi, A., Fitani, A. and Mshelbwala, P.P. (2020) 'Knowledge, attitudes, and practices regarding rabies in El jadida region, Morocco', *Veterinary Sciences*, 7(1). Available at: https://doi.org/10.3390/vetsci7010029
8. Corfmat, J. Gibson, A.D., Mellanby, R.J, Watson, W., Appupillai, M., Yale, G. *et al.* (2022) 'Community attitudes and perceptions towards free-roaming dogs in Goa, India', *Journal of Applied Animal Welfare Science*, 00(00), pp. 1–17. Available at: https://doi.org/10.1080/10888705.2021.2014839.
9. Costa, G.B., Gilbert, A., Monroe, B., Blanton, J., Ngam, S.N., Recuenco, S. *et al.* (2018) 'The influence of poverty and rabies knowledge on healthcare

seeking behaviors and dog ownership, Cameroon', *PLoS ONE*, 13(6). Available at: https://doi.org/10.1371/journal.pone.0197330.

10. Davlin, S.L. Lapiz, S.M., Miranda, M.E. and Murray, K.O. (2014) 'Knowledge, attitudes, and practices regarding rabies in Filipinos following implementation of the Bohol Rabies Prevention and Elimination Programme', *Epidemiology and Infection*, 142(7), pp. 1476–1485. Available at: https://doi.org/10.1017/S0950268813002513.
11. Digafe, R.T., Kifelew, L.G. and Mechesso, A.F. (2015) 'Knowledge, attitudes and practices towards rabies: Questionnaire survey in rural household heads of Gondar Zuria District, Ethiopia', *BMC Research Notes*, 8(1). Available at: https://doi.org/10.1186/s13104-015-1357-8.
12. Dubey, S., Singh, R.V., Gupta, B., Gupta, D.K., Nyak, A., Rai, A. *et al.* (2022) 'Study on rabies control and prevention using knowledge attitude and practice in Jabalpur central India', 11(7), pp. 2353–2359.
13. Ebuy, Y., Alemayehu, T., Reda, M., Berhe, M. and Bsrat, A. (2019) 'Community Knowledge, Attitude and Practice on Rabies, Incidence in Humans and Animals and Risk Factors to Rabies in Selected Districts of Tigray Region, Ethiopia', *Nigerian Veterinary Journal*, 40(2), pp. 147–163.
14. Edukugho, A.A., Umoh, J.U., Diem, M., Ajani, O., Uba, B., Okeke, L. *et al.* (2018) 'Knowledge, attitudes and practices towards rabies prevention among residents of Abuja municipal area council, Federal Capital Territory, Nigeria', *Pan African Medical Journal*, 31, pp. 1–11. Available at: https://doi.org/10.11604/pamj.2018.31.21.15120.
15. eThekwini Municipality (2017) Integrated Development Plan (IDP) Annual Review - *2017/2018*; Durban eThekwini Municipality; 2017.

16. Fazeli, M., Golahdouz, M., Bashar, R., Bafrrani, M.A., Doosti-Irani, A., Pourhossein, B. *et al.* (2018) ‘Design and Validation of a Persian Knowledge, Attitude, and Practice Questionnaire for Rabies (PKAP-Rabies) in the General Population of Iran’, *Journal of Medical Microbiology and Infectious Diseases*, 6(2), pp. 67–71. Available at: https://doi.org/10.29252/jommid.6.2.3.67
17. Fenelon, N., Dely, P., Katz, M.A., Schaad, N.D., Dismer, A., Moran, D. *et al.* (2017) ‘Knowledge, attitudes and practices regarding rabies risk in community members and healthcare professionals: Pétionville, Haiti, 2013’, *Epidemiology and Infection*, 145(8), pp. 1624–1634. Available at: https://doi.org/10.1017/S0950268816003125
18. Fesseha, H. and Hadgu, A. (2020) ‘Assessment of Knowledge, Attitude and Practices on Rabies among Communities of Atsbi Wonberta District, Tigray, Northern Ethiopia’, *Current Research in Agriculture and Farming*, 1(1), pp. 21–31. Available at: https://doi.org/10.18782/2582-7146.104
19. Gebeyaw, S. and Teshome, D. (2016) ‘Study on Community Knowledge, Attitude and Practice of Rabies in and Around Dessie City’, *Austin Journal of Veterinary Science & Animimal Husbandry*, 3(1), p. 5.
20. Gebremeskel, K.A., Tanga, B.M., Getachew, A. and Eshetu, Y. (2019) ‘Assessment of public knowledge, attitude and practices towards rabies in the community of Kombolcha, Southern Wollo, Amhara Reginal State, Ethiopia’, *Journal of Public Health and Epidemiology*, 11(1), pp. 38–48. Available at: https://doi.org/10.5897/jphe2017.0965
21. Guadu, T., Shite, A., Chanie, M., Bogale, B. and Fentahun, T. (2014) ‘Assessment of Knowledge, Attitude and Practices about Rabies and

Associated Factors: In the Case of Bahir Dar Town', *Global Veterinaria*, 13(3), pp. 348–354. Available at: https://doi.org/10.5829/idosi.gv.2014.13.03.8579.

22. Hampson, K., Coudeville, L., Lembo, T., Sambo, M., Kieffer, A., Attlan, M. *et al*. (2015) 'Estimating the Global Burden of Endemic Canine Rabies', *Plos Neglected Tropical Diseases,* 9(4): e0003709. Available at: https://doi.org/10.1371/journal.pntd.0003709
23. Hergert, M., LeRoux, K. and Nel, L.H. (2016) 'Risk factors associated with nonvaccination rabies status of dogs in KwaZulu-Natal, South Africa', *Veterinary Medicine: Research and Reports*, Volume 7, pp. 75–83. Available at: https://doi.org/10.2147/vmrr.s103859
24. Hergert, M. and Nel, L.H. (2013) 'Dog Bite Histories and Response to Incidents in Canine Rabies-Enzootic KwaZulu-Natal, South Africa', *PLoS Neglected Tropical Diseases*, 7(4), pp. 1–11. Available at: https://doi.org/10.1371/journal.pntd.0002059
25. Kiratitana-olan, K., Chaisowwong, W., Thongkorn, K. and Kreausukon, K. (2021) 'Keys to Good Knowledge, Attitude and Practice for Rabies Prevention in Disease-Free Communities in Thailand', *Veterinary Intergrative Sciences,* 19(3): 407-422
26. Lankester, F.J., Wouters, P.A.W.M., Czupryna, A., Palmer, G.H., Mzimbiri, I., Cleaveland, S. *et al.* (2016) 'Thermotolerance of an inactivated rabies vaccine for dogs', *Vaccine*, 34(46), pp. 5504–5511. Available at: https://doi.org/10.1016/j.vaccine.2016.10.015
27. Mbilo, C., Kabongo, J., Pyana, P.P., Nlonda, L., Nzita, R.W., Luntadila, B. *et al.* (2019) 'Dog ecology, bite incidence, and disease awareness: A cross-sectional survey among a rabies-affected community in the democratic

republic of the congo', *Vaccines*, 7(3). Available at: https://doi.org/10.3390/vaccines7030098

28. Morters, M.K., McNabb, S., Horton, D.L., Fooks, A.R., Schoeman, J.P., Whay, H.R. *et al.* (2015) 'Effective vaccination against rabies in puppies in rabies endemic regions', *Veterinary Record*, 177(6), p. 150. Available at: https://doi.org/10.1136/vr.102975
29. Ngah Osoe Bouli, F.P., Awah-Ndukum, J., Tejiokem, C.M. and Mingoas Kilekoung, J. (2018) 'Knowledge, attitudes and practices (KAP) evaluation regarding rabies in the Centre, Adamawa and North Regions of Cameroon', *Journal of Animal Science and Veterinary Medicine*, 3(6), pp. 178–183. Available at: https://doi.org/10.31248/jasvm2018.114
30. Ngugi, J.N., Maza, A.K., Omolo, O.J. and Obonyo, M. (2018) 'Epidemiology and surveillance of human animal-bite injuries and rabies post-exposure prophylaxis, in selected counties in Kenya, 2011-2016', *BMC Public Health*, 18(1). Available at: https://doi.org/10.1186/s12889-018-5888-5
31. Ntampaka, P., Nyaga, P.N., Niragire, F., Gathumbi, J.F. and Tukei, M. (2019) 'Knowledge, attitudes and practices regarding rabies and its control among dog owners in Kigali city, Rwanda', pp. 1–16. Available at: https://doi.org/10.1371/journal.pone.0210044
32. Pal, P., Yawongsa, A., Bhusal, T.N., Bashyal, R. and Rukkwamsuk, T. (2021) 'Knowledge, attitude, and practice about rabies prevention and control: A community survey in Nepal', *Veterinary World*, 14(4), pp. 923–942. Available at: https://doi.org/10.14202/vetworld.2021.933-942
33. Rine, R.C., Dogara, G.S. and Pam, M.D. (2017) 'Knowledge, Attitude and Practice of Rabies in and Around Lafia Metropolis, Nigeria', *Central African*

Journal of Public Health, 3(3), pp 27-33. Available at: https://doi.org/10.11648/j.cajph.20210303.11

34. Sabeta, C.T., Weyer, J., Geertsma, P., Mohale, D., Miyen, J., Blumberg, L.H. *et al.* (2013) 'Emergence of rabies in the Gauteng Province, South Africa: 2010-2011', *Journal of the South African Veterinary Association*, 84(1), pp. 2010–2011. Available at: https://doi.org/10.4102/jsava.v84i1.923

35. Savadogo, M., Tialla, D., Ouattara, B., Dahourou, L.D., Ossebi, W., Ilboudo, S.G. *et al.* (2021) 'Factors associated with owned-dogs' vaccination against rabies: A household survey in Bobo Dioulasso, Burkina Faso', *Veterinary Medicine and Science*, 7(4), pp. 1096–1106. Available at: https://doi.org/10.1002/vms3.468

36. Spargo, R.M., Coetzer, A., Makuvadze, F.T., Chikerema, S.M., Chiwerere, V., Bhara, E. *et al*. (2021) 'Knowledge, attitudes and practices towards rabies: A survey of the general population residing in the Harare Metropolitan Province of Zimbabwe' *PLos ONE* 16(1): e0246103. Available at: https://doi.org/10.1371/journal.pone.0246103

37. Straily, A. and Trevino-Garrison, I. (2017) 'Knowledge, Attitudes and Practices of Law Enforcement Officers on Rabies and Animal Control Issues in Kansas', pp. 111–117. Available at: https://doi.org/10.1111/zph.12287

38. Tandon, S., Kotwal, S.K., Malik, M.A., Singh, M., Kumar, D., Shafiq, M. *et al.* (2017) 'A Community Based Survey on Rabies Control and Prevention using KAP in', (December), pp. 1019–1028. Available at: https://doi.org/10.5958/2277-940X.2017.00153.X

39. Tiwari, H.K., Robertson, I.D., O'Dea, M. and Vanak, A.T. (2019) 'Knowledge, attitudes and practices (KAP) towards rabies and free roaming dogs (FRD) in

Panchkula district of north India: A cross sectional study of urban residents', *PLoS Neglected Tropical Diseases*, 13(4), pp. 1–17. Available at: https://doi.org/10.1371/journal.pntd.0007384

40. Tiwari, H.K., O'Dea, M., Robertson, I.D., and Vanak, A.T. (2019) 'Knowledge, attitudes and practices (KAP) towards rabies and free-roaming dogs (FRD) in Shirsuphal village in western India: A community based cross-sectional study', *PLoS Neglected Tropical Diseases*, 13(1). Available at: https://doi.org/10.1371/journal.pntd.0007120
41. Tripathy, R.M., Satapathy, S.P. and Karmee, N. (2017) 'Assessment of knowledge, attitude and practice regarding rabies and its prevention among construction workers: a cross-sectional study in Berhampur, Odisha', *International Journal of Research in Medical Sciences*, 5(9), p. 3971. Available at: https://doi.org/10.18203/2320-6012.ijrms20173964
42. Tschopp, R., Bekele, S. and Aseffa, A. (2016) 'Dog Demography, Animal Bite Management and Rabies Knowledge-Attitude and Practices in the Awash Basin, Eastern Ethiopia', *PLoS Neglected Tropical Diseases*, 10(2). Available at: https://doi.org/10.1371/journal.pntd.0004471
43. Ubeyratne, K., Srikitjakarn, L., Pfeiffer, D., Kong, F., Sunil-Chandra, N., Chaisowwong, W. *et al.* (2020) 'A Knowledge, Attitudes and Practices (KAP) survey on canine rabies prevention and control in four rural areas of Sri Lanka', *Transboundary and Emerging Diseases*, (November), pp. 1–15. Available at: https://doi.org/10.1111/tbed.13940
44. Weyer, J., Dermaux-Msimang, V., Grobbelaar, A., LeRoux, C., Moola, N., Paweska, J. *et al.* (2020) 'Epidemiology of human rabies in South Africa, 2008

- 2018’, *South African Medical Journal*, 110(9), pp. 877–881. Available at: https://doi.org/10.7196/SAMJ.2020.v110i9.14324

45. Yalemebrat, N., Bekele, T. and Melaku, M. (2016) ‘Journal of Veterinary Medicine and Animal Health Assessment of public knowledge, attitude and practices towards rabies in Debark Woreda, North Gondar, Ethiopia’, *Journal of Veterinary Medicine and Animal Health*, 8(11), pp. 183–192. Available at: https://doi.org/10.5897/JVMAH2016.0504

Supporting information

S1 Fig. Fig 1: Distribution of respondents by responses to questions regarding animals susceptible to rabies.

S2 Fig. Fig 2: The percentage of respondents based on how they responded to the question about the clinical signs of rabies in dogs.

S3 Fig. Fig 3: A bar graph showing the proportion of respondents based on their attitude towards rabies in eThekwini District

Response to editor

Comment: Line 163-164: Significance in the multivariable model was assessed at $p > 0.05$. Please correct this.

Response: corrected as advised, and now reads as follows: Significance in the multivariable model was assessed at $p< 0.05$ (see line 161

Response to editor

Please delete "prospective" from line 18, "of the respondents." from line 23 and "treatment" from line 25.

Response: corrected as advised

PEP includes wound washing, a dose of human rabies immune globulin (HRIG), and rabies vaccine given at the time of the first medical visit, and two booster doses. Please correct the statement at line 25.

Response: Corrected as advised-see lines 25-27

Please delete "rate" from line 56 as it is a proportion.

Response: “rate” deleted from line 58

Please replace all "Univariate" and "multivariate" with "univariable" and "multivariable" throughout the manuscript.

Response: corrected as advised.

Line 160: Please delete "binary".

Response: Corrected as advised-see line 162

Please mention the software used for the logistic regression analysis. Lines 318-322: Univariable results are not final, so please don't describe them.

Response: The following statement has been added under data analysis (lines 156-157)

Data was analysed using the Statistical Package for Social Science (SPSS) Version 27 (SPSS for Windows, version 27, SPSS, Inc, Chicago, Illinois, the USA).

Results of univariable regression have been removed as advised.
