# Supplementary material for: Knowledge, attitude, and practices towards rabies: A community survey in selected areas of KwaZulu-Natal Province, South Africa
Source: PLoS One. 2026 Jul 9;21(7):e0352279. doi: 10.1371/journal.pone.0352279 (PMC13349125; doi:10.1371/journal.pone.0352279)
Supplement: Attachment — Submitted filename: Response to Reviewers.docx [file pone.0352279.s004.docx]

Response to Reviewers

Minor:

1. Throughout the document, the authors need to be congisant and specify rabies as a disease and rabies virus as the aetiologic agent of the disease.

**Response**: The authors have specified rabies as either virus or disease as suggested by the reviewer(s)

ii. The third line in the introduction should be amended to read "All warm-blooded mammals are susceptible to rabies virus infection generally transmitted through the bite of an infected rabid animal",

**Response**: As suggested by the reviewer (s), the authors have amended the sentence to the above mentioned.

iii. Also amend the sentence "Rabies infection is attributable to domestic dogs and remains .........perspective",.

**Response**: The sentence was amended according to the reviewer’s suggestion to: Dog mediated rabies infection remains a significant disease from a public health perspective.

iv. The estimated number of 60 000 human deaths was cited by Hampson et al., 2015 and not Morters et al., 2015.

**Response**: Morters et al., 2015 ‘Effective vaccination against rabies in puppies in rabies endemic regions’ has estimated that 60 000 human deaths occur yearly, while Hampson et al., ‘Estimating the Global Burden of Endemic Canine Rabies’ estimated mortalities in human annually to be around 59 000.

v. Combine the two sentences "Almost all (98%) ............and In Africa" as both state the same issue.

**Response**: The sentences have been combined to; In Africa, over 90% of human rabies deaths are associated with dog-bite.

vi. The sentence that starts "Rabies can only be prevented but not cured" - there are a few cases of human who were cured - please refer to the Willoughby and Milwakee protocol for this.

**Response**: Thank you for referring us to Willoughby and Milwaukee protocol. The authors have rephrased the statement.

vii. On page 3, a combination of rabies immunoglobulin and vaccine is given depending on the vategory of the bite - please qualify this.

**Response**: For example, a dog bite victim determined to be a category 3, is a patient who has been bitten by an animal suspected of having rabies and the victim presents with open wounds and bleeding on the bite site.

viii. Please provide a reference for the sentence that talks about rabies having the highest case fatality rate of any disease,

**Response**: The authors cited Rine *et al*., 2017, who reported that rabies has highest case fatality rate of any disease

ix. Replace "reduce rabies disease" with "control the disease at its source",. second para page

**Response:** Accordingly, the authors replaced “reduce rabies disease” with “control at its source”

3. Secondly, replace "outbreaks occur" with "rabies is endemic".

**Response**: As suggested by the reviewers, the authors replaced “outbreak occur” with “rabies is endemic”.

x. What is a "high burden of rabies mortality persists",

**Response**: "high burden of rabies mortality persists” means that high number of people dying due to rabies remain unchecked. However, the sentence was rephrased to read as follows: “However, in most developing countries, rabies continues to be a challenge resulting in high mortalities in humans and animals”.

xi. Rewrite the sentence to read "In South Africa, an estimated 40 human rabies rleated deaths from dog bite contacts occur yearly", In the same para, include Limpopo and the Eastern Cape provinces in addition to KZN. You should also include records and illustrations from the Department of Agriculture Land Reform and Rural Development to depict the current rabies situation in the country.

**Response:** The authors have highlighted Limpopo and Eastern cape as other provinces that experience rabies as reported by Department of Agriculture, Land Reform and Rural Development.

xii. In the last para, delete disease so that it reads "rabies" only,

**Response:** Disease was deleted

xiii. First sentence on page 4 - specify the selected rural and urban areas.

**Response:** The areas have been specified as rural (Embo) and urban (Verulam).

xiv. Please provide a map of the country and the study areas to guide the reader of the geographical location of this area.

**Response:** We failed to secure a good clear map that shows the two areas. The few maps we managed to obtain, although not good, also require applying for copyright which we have failed to secure. We have thus opted to provide the coordinates for the two areas.

Embo (Latitude: 29° 54' 34" S, Longitude: 30° 40' 29" E)

Verulam (Latitude: -29° 38' 59.99" S; Longitude: 31° 02' 60.00" E

xv. Second para, replace "populated by different races" by "a mulit-racial community", and delete ""for different races" at the end of the sentence.

**Response:** The words in the sentence have been revised according to what the reviewer(s) suggested.

xvi. Third para, rewrite to read "Verulam has an estimated 64,950 households".

**Response:** The sentence has been rephrased to what the reviewer has suggested

xvii. Under data collection - insert "a" after "using",

**Response:** “a” was inserted

xix. Under section 4 - replace reported with disseminated.

**Response:** The authors replaced “reported” with “disseminated”

xx. Under 5.1.1 - replace among with in.

**Response:** Among was replaced with “in”

xxi. In Table 1, I suggest replace matric with "high school education",

**Response**: Thanks for the suggestion. We have amended accordingly.

xxii. Under Table 2, replace "being responsible to" with "the likely risk for",

**Response:** The sentence was revised by addition of “the likely risk for”

xxiii. On page 11 - rewrite to read "The inability of a dog to drink and eat as signs of rabies were mentioned".

**Response:** The authors have rephrased the sentence as suggested

xxiv. Rewrite in para 5 - "indicated that their pets were not sterilised", on page 14 - was there a follow up on why >50% of the respondents were not in favour of sterilisation?

**Response:** Cross-sectional study design was employed in this study, which was only conducted once. We agree it would have been good to follow up. We will take this into consideration in future or follow up studies.

xxv. On page 13, does ongoing campaigns refer to annual vaccination campaigns?

**Response:** The authors have rephrased the sentence to specify that this refers to annual vaccination campaigns

xxvi. On page 15, replace referent with reference,

**Response:** Referent was replaced with reference

xxvii. Second sentence of the discussion - delate the "causes" at the end of the snetence.,

**Response:** Causes was removed from the sentence

xxviii. Under 6.1 - rewrite to read ".... when it comes to pet management in the homes",.

**Response:** The authors amended as recommended and added: “when it comes to pet management in the homes” to the sentence

xxix. First para on page 17 - rewrite to read "Findings from the present study also contrast those from a study conducted in Cameroon in which less than half of the respondents owned pets",.

**Response:** The authors have rephrased the sentence as advised to read as: Findings from the present study also contrast those from a study conducted in Cameroon in which less than half of the respondents owned pets.

xxx. second para - "In this study, just below half (47.66%) of respondents were Christians, contrary to findings in studies conducted in ...........distrcits which reported Muslim populations of 56.8% and 94.8% respectively".,

**Response:** The authors have rephrased the sentence to the one suggested by the reviewers

xxxi. Under 6.2 - how are the results better?

**Response:** Thanks for pointing this out, we have rephrsed the sentence to read as follows: **“**However, in a study conducted in Kombolcha, Ethiopia, a higher number (76.6%; n=294) of respondents were aware.

xxxii. Under references - is Abedela Nehash (2017) and Abdela et al., 2017 - the same reference? Please check whether the following are included in the text (I may have missed them) - Lankester et al., 2016; Sabeta et al., 2013; Report CF 2010; Tiwari, Vanak et al., 2019a; Ubeyratne et al., 2020;

**Response:** The authors have checked and ensured that all cited references are listed in the bibliography

**Major:**

i. Sample size determination - Was the 50% prevalence based on previous research studies e.g. Hergert and Nel?

**Response:** The authors have not found studies that looked at the “prevalence” of knowledge that were previously done in the selected areas of study and therefore assumed prevalence of knowledge to be 50%. Available literature refers to the prevalence of rabies which was not the focus of this study.

ii. On page 9, it is indicated that playing with the dog could lead to contraction of rabies - during the discussions was there any implications that other host species could be included, e.g. cats, willdife?

**Response:** We thank the reviewer for the comment. Unfortunately, it is not clear to us what the reviewer wanted us to do, or which change to make.

iii. Third para - please rewrite "... that vaccination of pets is important to prevent humans contracting the disease".

**Response:** The sentence was rephrased to the suggested

iv. One major weakness in the manuscript and in the discussion is the repetition of results,

**Response:** The authors have reviewed the discussion to avoid repetition of results.

v. On page 19, 3rd para - discuss the differences in the results?

**Response:** The authors have discussed the differences in results

vi. Under 6.3 - is the endemicity of rabies in KZN and India and Ethiopia comparable - give the reader something to think about.

**Response:** We appreciate this comment-we have added something on India and Ethiopia to the effect that like SA it is a rabies endemic area.

vii. In the discussion, there are very few references being disucssed in the context of the results obtained here - Gebremeskei et al., 2019; Bihon et al., 2020. The authors should expand and compare the findings to other regions.

**Response:** we take note of the reviewer’s concern. We would like to reiterate that we have endeavoured to discussion our findings in the context of what is known and also to explain our findings. We refer to studies from Rwanda, Morocco, india, Ethiopia, Filipions etc. It is possible, we missed what the reviewer intended us to do. We will gladly attend to it if further clarification is provided**.**

viii. The authors on page 24 indicated that adhering to repeated and regular vaccinations helps boost the immune level of pets - what important question is what the turnover rate of the dog population we are dealing with here?

**Response:** The question was intended to reveal whether the respondent would be keen to another vaccination when the campaigns are conducted. That notwithstanding we agree with the reviewer that it would be good to consider the turnover rate of the dog population in the study area to establish the “herd immunity”. However, this was outside the scope of this work.

ix. On page 25 - the authors allude to rabies eradiation campaigns? Is this achievable - I suggest that the authors use elimination of dog-mediated human rabies as other wildife vectors bats, jackals have a potential to spill over into the dog population.

**Response:** The authors appreciate the suggestion and therefore made the necessary changes.

x. Under conclusions, the authors highlighted the identified gaps. But what is important is an intervention. How will these gaps fit into the national rabies control programme of South Africa as we move towards 2030? There is currently a one health group - how will these gaps help strengthen the current collaboration in that province?

**Response:** The 2030 agenda aims to eliminate rabies, through mass dog vaccination campaigns that achieve 70%vaccination rate per annum (Mbilo *et al*., 2019). The authors recommended that to bridge the gaps, implementation of global strategies such as awareness and vaccination campaigns to address such should be effective in order to eradicate rabies in South Africa.

xi. Coertse et al., 2017 - refers to rabies-related Mokola and not to dog rabies. Please include an appropriate reference for dog rabies in South Africa and there are many such references.

**Response: corrected.** Reference has been added.

Reviewer #2: The authors conducted a descriptive analysis of rabies Knowledge, Attitudes, and Practices (KAP) in KwaZulu-Natal Province, South Africa, involving 768 participants. Based on the responses obtained, the authors concluded that a lack of knowledge regarding the cause of rabies and the importance of receiving Post-Exposure Prophylaxis (PEP) immediately after a dog bite was prevalent in the study area. However, they also identified certain gaps in awareness that require targeted interventions for the general population, including those who do not own pets.

**Response:** We are appreciative of the positive comment on our work by the reviewer. However, we would we would like to point out that our work also had an analytical component; hence we were able to make inferences.

While studies like this are informative and valuable for understanding the current state of knowledge and practices, there is room for advancement to make the research more substantial. The approach taken in this study, in its current form, may be considered too simplistic for publication in a high-impact journal, as it does not significantly contribute to advancing the field of research. Simply summarizing data is considered a basic step, and the authors are encouraged to perform more advanced analyses to provide deeper insights and contribute to the existing body of knowledge on this topic.

**Response:** The authors have analysed data using descriptive statistics and logistic regression model (univariate and multivariate analysis) to determine significant predictors of knowledge. We would appreciate if the reviewer was kind as to be specific on the type of advanced analysis he or she would have loved to see performed. But that aside, we believe that we were able to achieve the objectives for which we set out to do in this work. Last but least, we believe that this study contributes to improved understanding of the disease in the study area that happens to be endemic to rabies.

The authors are urged to consider conducting more in-depth research, incorporating sophisticated methodologies or analyses that can offer novel perspectives and meaningful contributions to the field of rabies knowledge and prevention.

**Response:** The approach used in this study is widely used in KAP studies. Therefore, it was adapted, and we feel it is suited to this study. However, we are keen to learn more about what we could have done, if the reviewer was kind as to advise on the kind of analysis, he or she would like to see us perform to enrich this study.
