# Supplementary material for: Knowledge, attitude, and practices towards rabies: A community survey in selected areas of KwaZulu-Natal Province, South Africa
Source: PLoS One. 2026 Jul 9;21(7):e0352279. doi: 10.1371/journal.pone.0352279 (PMC13349125; doi:10.1371/journal.pone.0352279)
Supplement: Attachment — Submitted filename: Review.docx [file pone.0352279.s008.docx]

I was asked to review the manuscript along with the previous reviewers’ comments. Overall, I believe that the authors present a well conducted study which presents important information for policy makers in South Africa. Additionally, they highlight important knowledge gaps in individuals, and I believe this manuscript to include important information.

However, the manuscript is lengthy, and I recommend to the authors that they cut down a lot of what is presented in the results and discussion. Specifically, the results are presented both in Tables/Figures and they reiterated in the text. It is fairly redundant, and the authors need to have more brevity in the results section. The discussion section is a lot of reiteration of the results which should be removed. The discussion section is to interpret the results only. In my opinion, for this manuscript to be published, there needs to be substantial re-organization of the results and discussion sections

In addition, I have line-by-line comments to be addressed.

- Line 16 – To assess what type of gap? Are you referring to a knowledge gap here?
- L20 –‘years of age’ should be used instead of ‘years’
- L20 – ‘validated’ would be more appropriate to use instead of ‘pre-tested’ here.
- L21 – you can remove the alpha here to save space in the abstract. It generally is not needed in an abstract.
- L22 and L23 and L27, L28– Please include the numerator and denominator when providing percentages.
- L27 – ‘said’ vs. ‘mentioned’. The word mention indicates an afterthought – that of which your participants didn’t respond with.
- L29 – Please include percentage, numerator and denominator.
- L28 – please remove ‘on the other hand’ as this is not scientific language. I also recommend removing ‘greatest’ as this is subjective qualifier. I recommend rephrasing to ‘However, they pose the threat of transmitting rabies’.
- L41 – the category ‘warm-blooded’ is not a scientific category and should not be used. I recommend just keeping it as ‘mammals’ (since all mammals are susceptible to rabies). I know a previously reviewer asked for the inclusion of this – but I highly disagree with it as it is not scientifically correct.
- L45 and L46 – Please combine these two sentences, they should not be separate.
- L47 – 31,539 is not an approximate number (you are giving an exact number). I recommend either remove the word approximate or rounding the value to 31,500 to give an approximate. I recommend instead using a value from Africa (since that is where your study is taking place). Additionally, can you confirm that all of these deaths were directly attributed to rabies? If not, then this does not suggest a high rate of rabies. Additionally, ‘high burden’ is not scientific – please change to ‘high rate’.
- L52 – It would be more scientific to state mortality rates here – please also find a citation for this
- L55 - you should mention doses of human rabies immune globulin (HRIG) here.
- L57- please give explicit values.
- L 74 – 77 – This needs to be referenced.
- L78 – Please use the acronym KAP here since you’ve already introduced it.
- L81 – Again, please use the acronym KAP here since you’ve already introduced it.
- L105 – ‘was used’ instead of ‘was adopted’
- L105 – please use the acronym KAP here.
- L135 - please use the acronym KAP here.
- L147 – ‘was recorded using Microsoft Excel’
- L148 – How is there a ‘correct/incorrect response’ for each answer? I recommend rephrasing.
- L182-184 – I don’t believe it’s necessary to mention this. Plans for dissemination is not relevant to the study.
- Results – with all percentages given, the associated numerator and denominator need to be given in parathesis directly following the percentage.
- L207 – ‘route’ not ‘rout’
- Results – A lot of the results that are presented in the tables, are reiterated in the text. This fatigues the reader making the manuscript hard to read overall since it is very repetitive, and the authors need to cut the information down. Please reconsider what is in the text and what is presented in the tables.
- L322 – please remove the word ‘generous’ – this is not to be used in scientific writing.
- L221 – please give the specific p-value instead of stating that it is > 0.05
- Discussion – The discussion needs to be cut down considerably and the authors need to reconsider what information is pertinent to the study. At present, the discussion is currently 14 pages long, which is a lot. Overall, there is a lot of re-presenting the results which should not occur. The discussion should only be used to interpret the results and not presenting the results again. Additionally, there is too many instances where the authors are comparing their results to other study. It’s great to compare results, but the authors should consider judicious use of this and should instead focus on interpreting their own results.
- L338 – Please use the acronym KAP
- L379 – I would argue that 27% is not a very low number – restate to something along the lines of ‘minority’
- L383 – If you are stating results from another study, please also include the numerator and denominator so that the reader can know how many participants answered the questionnaire. Please fix here and throughout the rest of the manuscript.
- L400 – 12% is not a ‘mere’ amount – re-state to say ‘minority’
- L411 – Please use the acronym PEP
- L460 - remove ‘the’ at the start of the sentence.
- L494 – “The number that did not know, even though small, cannot be ignored.” – This sentence is confusing, and I believe there is a typo. Please rephrase.
- L639 – “Just as was observed” is a confusing way to start a sentence – please rephrase.
- L684 – ‘latter’ vs ‘later’
- Figures and table captions – I recommend to the authors that they modify the captions for the figures and captions so that they can be understood without reference to the text
- Figures – I highly recommend to the authors that they include a map of their study site. This would be helpful for the reader to understand.
