# Supplementary material for: Knowledge, attitude, and practices towards rabies: A community survey in selected areas of KwaZulu-Natal Province, South Africa
Source: PLoS One. 2026 Jul 9;21(7):e0352279. doi: 10.1371/journal.pone.0352279 (PMC13349125; doi:10.1371/journal.pone.0352279)
Supplement: Attachment — Submitted filename: Rebuttal.docx [file pone.0352279.s009.docx]

Response to the reviewer’s comments and suggestions

Reviewer: Overall, I believe that the authors present a well conducted study which presents important information for policy makers in South Africa. Additionally, they highlight important knowledge gaps in individuals, and I believe this manuscript to include important information.

Response: We would like to thank the reviewer for the positive comment with respect to our work

Reviewer: However, the manuscript is lengthy, and I recommend to the authors that they cut down a lot of what is presented in the results and discussion. Specifically, the results are presented both in Tables/Figures and they reiterated in the text. It is redundant, and the authors need to have more brevity in the results section.

Response: We acknowledge the reviewer’s concern regarding how we presented the results. Consequently, we have cut down on the reiteration of the results in the text.

Reviewer: The discussion section is a lot of reiteration of the results which should be removed. The discussion section is to interpret the results only. In my opinion, for this manuscript to be published, there needs to be substantial re-organization of the results and discussion sections

Response: We are grateful to the reviewer for pointing this out. We have consequently removed most of the results that were appearing in the discussion. Only where we felt that it was necessary to reiterate the results have we left the results in.

Reviewer: Line 16 – To assess what type of gap? Are you referring to a knowledge gap here?

Response: Thank you for picking this up, we have provided clarification, and it now reads as follows: “gaps in the KAP towards rabies”

Reviewer: L20 –‘years of age’ should be used instead of ‘years’

Response: Change has been implemented as recommended

Reviewer: L20 – ‘validated’ would be more appropriate to use instead of ‘pre-tested’ here.

Response: pre-tested has been replaced with validated

Reviewer: L21 – you can remove the alpha here to save space in the abstract. It generally is not needed in an abstract.

Response: Alpha has been removed as recommended.

Reviewer: L22 and L23 and L27, L28– Please include the numerator and denominator when providing percentages.

Response: We were not sure whether to respond to this suggestion given that the reviewer wanted us to limit reiterating the results in the text. Since the numerator is in the table, we thought it was not necessary to repeat each one of them it in the text. But nonetheless, we have adopted the recommendation made by the reviewer.

Comment: L27 – ‘said’ vs. ‘mentioned’. The word mention indicates an afterthought – that of which your participants didn’t respond with.

Response: Mentioned was replaced by “said”.

Comment: L29 – Please include percentage, numerator and denominator.

Response: We have included the percentages, numerator and denominator as requested.

Reviewer: L28 – please remove ‘on the other hand’ as this is not scientific language. I also recommend removing ‘greatest’ as this is subjective qualifier. I recommend rephrasing to ‘However, they pose the threat of transmitting rabies’.

Response: Both ‘on the other hand’ and ‘greatest’, have been removed as advised.

Reviewer: L41 – the category ‘warm-blooded’ is not a scientific category and should not be used. I recommend just keeping it as ‘mammals’ (since all mammals are susceptible to rabies). I know a previously reviewer asked for the inclusion of this – but I highly disagree with it as it is not scientifically correct.

Response: the change has been made, and ‘warm-blooded’ has been replaced by “mammals”

Reviewer: L45 and L46 – Please combine these two sentences, they should not be separate.

Response: The two sentences have been combined.

Reviewer: L47 – 31,539 is not an approximate number (you are giving an exact number). I recommend either remove the word approximate or rounding the value to 31,500 to give an approximate. I recommend instead using a value from Africa (since that is where your study is taking place). Additionally, can you confirm that all of these deaths were directly attributed to rabies? If not, then this does not suggest a high rate of rabies. Additionally, ‘high burden’ is not scientific – please change to ‘high rate’.

Response: the word ‘approximate has been removed. However, we do not agree with the reviewer on the word burden not being scientific. In Epidemiology, the word burden is used to describe the impact of a disease on the population, and this includes morbidity and mortality. The word “rate” in Epidemiology has an element of time. So if we talk of rate of rabies, it means the number of rabies cases occurring over a specific period of time. As a result we decided not to replace burden with rate.

Reviewer: L52 – It would be more scientific to state mortality rates here – please also find a citation for this

Response: Reference has been added

Reviewer: L55 - you should mention doses of human rabies immune globulin (HRIG) here.

Response: We appreciate the reviewer’s suggestion on the number of injections. However, we would like to note that the number of injections of rabies immune globulin and vaccinations depend on the categorization of wound. It is not just one fixed number of injections. We feel that dwelling on the treatment of rabies cases will not improve on the paper. Since this is not the focus of the paper, we decided to refer the reader to documents where details of treatment of rabies in humans is described in detail. The following sentence has been added in line: “Details of the treatment that is prescribed for victims of dog bites to prevent development of the disease rabies, is described in details by Bishop et al (2010).”

Reviewer: L57- please give explicit values.

Response: We are not sure which figures the reviewer is referring to here. But if it is the number of vaccinations the victims received, refer to the comment above on treatment of rabies. The number of injections given depend on the category of the dog bite wound.

Reviewer: L 74 – 77 – This needs to be referenced.

Response: reference has been provided.

Reviewer: L78 – Please use the acronym KAP here since you’ve already introduced it.

Response: We acknowledge the suggestion by the reviewer. However, we are of the view that it is not good practice to begin a sentence with an abbreviation and/or acronym. As a result, we did not change to acronym KAP here.

Reviewer: L81 – Again, please use the acronym KAP here since you’ve already introduced it.

Response: the change has been implemented.

Reviewer: L105 – ‘was used’ instead of ‘was adopted’

Response: the change has been made

Reviewer: L105 – please use the acronym KAP here.

Response: Change implemented.

Reviewer: L135 - please use the acronym KAP here.

Response: Done

Reviewer: L147 – ‘was recorded using Microsoft Excel’

Response: implemented

Reviewer: L148 – How is there a ‘correct/incorrect response’ for each answer? I recommend rephrasing.

Response: Yes, there is a correct/incorrect response for each answer. But this specifically refers to the section on Knowledge. It would not have been possible to score the knowledge if we did not do it this way.

Reviewer: L182-184 – I don’t believe it’s necessary to mention this. Plans for dissemination is not relevant to the study.

Response: Thank you for the suggestion. We have removed the plans for dissemination.

Reviewer: Results – with all percentages given, the associated numerator and denominator need to be given in parathesis directly following the percentage.

Response: The numerator and denominator associated with percentage has been added where needed.

Reviewer: L207 – ‘route’ not ‘rout’

Response: Thanks for picking this up. We have implemented the suggestion.

Reviewer: Results – A lot of the results that are presented in the tables, are reiterated in the text. This fatigues the reader making the manuscript hard to read overall since it is very repetitive, and the authors need to cut the information down. Please reconsider what is in the text and what is presented in the tables.

Response: As mentioned earlier we have tried to minimise

Reviewer: L322 – please remove the word ‘generous’ – this is not to be used in scientific writing.

Response: We take note of the reviewer’s comment. However, in epidemiology, it is acceptable to use the phrase ‘generous’. We have used the word in several work we have published in this journal and others in the past.

Reviewer: L221 – please give the specific p-value instead of stating that it is > 0.05

Response: We acknowledge the reviewer’s comment. However, given that the variable is categorical with several levels, stating the exact p-value would look clumsy. The reader can refer to the table if in need of the exact p-value for each level.

Reviewer: Discussion – The discussion needs to be cut down considerably and the authors need to reconsider what information is pertinent to the study. At present, the discussion is currently 14 pages long, which is a lot. Overall, there is a lot of re-presenting the results which should not occur. The discussion should only be used to interpret the results and not presenting the results again. Additionally, there is too many instances where the authors are comparing their results to other study. It’s great to compare results, but the authors should consider judicious use of this and should instead focus on interpreting their own results.

Response: We have cut down the discussion, particularly by limiting reiterating the results in the discussion section to the extent possible without the paper being watered down. However, on the issue of limiting comparison of our findings and those of other authors, we are of the view that unlike the results section, the discussion should in addition to interpreting the findings, focus on locating findings of the study in the larger body of existing research. In view of this, we are of the view that compare results with those of other peer-reviewed papers is an important component of the discussion. We are thus reluctant to cut down on the comparison.

Reviewer: L338 – Please use the acronym KAP

Response: Done

Reviewer: L379 – I would argue that 27% is not a very low number – restate to something along the lines of ‘minority’

Response: The statement has been reviewed and changed

Reviewer: L383 – If you are stating results from another study, please also include the numerator and denominator so that the reader can know how many participants answered the questionnaire. Please fix here and throughout the rest of the manuscript.

Response: We have implemented the suggestion throughout the discussion.

Reviewer: L400 – 12% is not a ‘mere’ amount – re-state to say ‘minority’

Response: suggestion implemented as recommended.

Reviewer: L411 – Please use the acronym PEP

Response: Implemented as recommended.

Reviewer: L460 - remove ‘the’ at the start of the sentence.

Response: implemented as recommended.

Reviewer: L494 – “The number that did not know, even though small, cannot be ignored.” – This sentence is confusing, and I believe there is a typo. Please rephrase.

Response: The sentence has been rephrased to make it clearer. It now reads as follows: “From a public health standpoint, the fact that the overwhelming majority of respondents in this study knew that rabies can be transmitted from animals to humans is a significant finding. Likewise, even though the number of people who did not know that rabies can be transmitted from animals to humans was small, it should be considered a serious public health implication.’

Reviewer: L639 – “Just as was observed” is a confusing way to start a sentence – please rephrase.

Response: The sentence as been rephrased and reads as follows: “In a Filipinos study it was observed that 64% of the respondents consistently vaccinated their dogs to prevent rabies [10].

Reviewer: L684 – ‘latter’ vs ‘later’

Response: Thanks for picking this -the correction has been made

Reviewer: Figures and table captions – I recommend to the authors that they modify the captions for the figures and captions so that they can be understood without reference to the text.

Response: We have modified the captions of figures and tables.

Reviewer: Figures – I highly recommend to the authors that they include a map of their study site. This would be helpful for the reader to understand.

Response: We would like to thank the reviewer for the suggestion. We had planned to include a map from the onset, did an extensive search for a suitable map and failed to get one. That is why we provide the GPS coordinates of the two areas where the study was conducted.
