# Supplementary material for: Knowledge, attitude, and practices towards rabies: A community survey in selected areas of KwaZulu-Natal Province, South Africa
Source: PLoS One. 2026 Jul 9;21(7):e0352279. doi: 10.1371/journal.pone.0352279 (PMC13349125; doi:10.1371/journal.pone.0352279)
Supplement: Attachment — Submitted filename: Review_declet_4.docx [file pone.0352279.s010.docx]

Thank you for editing your manuscript. It has improved with the edits made. I do have some edits remaining.

L22 – Please remove ‘twenty seven percent’ since you include 26.95 in parenthesis. Please rephrase this sentence to fix the issue.

L28 – Remove ‘only’ since 59% is a majority – not minority.

L80 – please introduce the acronym ‘KAP’ here.

L87 – since the KAP acronym has already been used (and should be introduced as per my previous comment), please use it consistently throughout the entire manuscript.

L115 - please elaborate how random was selected.

Results – There is still a lot of repetition of results. There’s no need to repeat the results in paragraph form if they are presented in the table.

Discussion – There is still too many discussions on the results of other studies. The authors should cut down using other studies and instead only talk about their results and what it means for KwaZulu-Natal Province.
