# Supplementary material for: Knowledge, attitude, and practices towards rabies: A community survey in selected areas of KwaZulu-Natal Province, South Africa
Source: PLoS One. 2026 Jul 9;21(7):e0352279. doi: 10.1371/journal.pone.0352279 (PMC13349125; doi:10.1371/journal.pone.0352279)
Supplement: Attachment — Submitted filename: Rebuttal letter (1).docx [file pone.0352279.s011.docx]

Rebuttal letter

Reviewer 4

**Reviewer:** Thank you for editing your manuscript. It has improved with the edits made. I do have some edits remaining.

Response: Thank you for this comment and for appreciating our effort

**Reviewer:** L22 – Please remove ‘twenty seven percent’ since you include 26.95 in parenthesis. Please rephrase this sentence to fix the issue.

Response: The reason ‘twenty seven percent was included was to avoid starting a sentence with a figure. Some journals do not accept it. However, on your advice we have removed it, and now the sentence starts with 26.95%.

**Reviewer:** L28 – Remove ‘only’ since 59% is a majority – not minority.

Response: Many thanks for the observation-the change has been implemented.

**Reviewer:** L80 – please introduce the acronym ‘KAP’ here.

Response: suggestion implemented

**Reviewer:** L87 – since the KAP acronym has already been used (and should be introduced as per my previous comment), please use it consistently throughout the entire manuscript.

Response: suggestion implemented

**Reviewer:** L115 - please elaborate how random was selected.

Response: We described how systematic random sampling was implemented; we are therefore not certain what the reviewer would like to see us add on the current description. Nonetheless, we have added the following statement about the sampling interval used in the two places and it reads as follows: (169 and 21 in Embo and Verulam respectively)-see line 119

**Reviewer:** Results – There is still a lot of repetition of results. There’s no need to repeat the results in paragraph form if they are presented in the table.

Response: We note the reviewer’s concern, however, we are not sure what the reviewer wants us to do, especially since the results and discussion are separated. Is the reviewer expecting us to remove the results and just include a statement referring to the table?

Using the example below, please advise how we should write the results, and we will gladly adopt based on your example.

Half (50.52%; 388/768) of respondents indicated that they did not know the cause of rabies, with only 26.95% (n=207/768) of the participants indicating that rabies is caused by a virus.

If we replaced the above with the following; “Respondents indicated that they did not know the cause of the rabies, while some of the participants indicated that rabies is caused by a virus.”

Will the above be acceptable to the reviewer since we will have removed the results?

Besides that the other reviewer advised that we be consistent and include results throughout the manuscript. This would mean putting back some of the results we removed so as to minimise repeating results in the text.

**Reviewer:** Discussion – There is still too many discussions on the results of other studies. The authors should cut down using other studies and instead only talk about their results and what it means for KwaZulu-Natal Province.

Response: We would love to implement the suggestion of the reviewer; however, we find it is at odds with the principle of placing our study in the broader context of existing literature. In fact, the other reviewer advised that we should compare our results with results from SA and/or neighbouring countries. He or she has provided several articles to help with this. Given these conflicting suggestions, we have reworked the discussion to accommodate both views.

Reviewer #6:

**Reviewer**: Overall, I believe the authors present a well conducted a study on the knowledge, attitudes and practices towards rabies study on two villages of the KwaZulu-Natal Province, South Africa. The study identified some gaps in the knowledge, attitudes and practices towards rabies from local populations. This information can be useful for policy makers and for the implementation of rabies control programs.

Response: Thanks for appreciating the effort put into this study- your kind words are highly appreciated.

**Reviewer**: I consider the manuscript to be lengthy, namely the results and discussion parts, especially the last one. I would especially recommend a full review of the discussion part. I consider that there is some repetition of the results in the discussion and for the manuscript to be published there is a need to re-organize results and discussion. As of special importance to the results – the percentage of vaccinated dogs – I think some clarifications are needed on the way the percentage was calculated. I suggest also to include the limitations within the discussion part and to provide the questionnaire in the supplementary materials.

Response: We note the comment by the reviewer regarding the length of the paper more so the results and the discussion. We respectfully point the reviewer to the fact that the paper is results-heavy (i.e., it has a lot of results). We thus not sure if the reviewer is suggesting that we drop some of the results and shorten the paper, or does the reviewer advise that we leave out the results in the text as shown below:

“Dog-bite was identified by most respondents (86.98%, 668/768) as the likely route for the transmission of rabies. In response to the question on the first thing to be done after a dog-bite while at home, slightly over half of the respondents (53.52%, 411/768) indicated that they would clean the wound with soap and running water (Table 2).”

We could rewrite the above paragraph in the following way and remove all the results:

Dog-bite was identified by most respondents as the likely route for the transmission of rabies. The first thing to be done after a dog-bite while at home according to some of the respondents, would be to clean the wound with soap and running water (Table 2).”

Would this be acceptable to the reviewer?

Reviewer further says: “re-organize results and discussion”;

Response: It is not clear what the reviewer would like us to do with this comment. Does the reviewer have a certain way or order in which the results should be presented? We have nonetheless reworked the results section to be best of our ability.

The review also indicated the following: “As of special importance to the results – the percentage of vaccinated dogs – I think some clarifications are needed on the way the percentage was calculated.

Response: We thank the reviewer for this comment and as a results we have used the number of people with dogs to calculate the vaccination coverage instead of using the total number of respondents as a proximate measure of the number of dogs vaccinated in the study area.

The reviewer said; “ I suggest also to include the limitations within the discussion part and to provide the questionnaire in the supplementary materials.

Response: We have provided the questionnaire as recommended. However, on the suggestions of including limitations for the study, we have a whole section dedicated to this under section 7, entitled “ **Limitations”.** It is not clear if the reviewer expects us to remove the section on limitation, and instead add limitations in the discussion part. Most journals require a section on the limitations to be included at the end of the paper as we have done. We thus respectfully decided to have a section on limitations.

**Reviewer:** L 24: I suggest putting the comma before “which”

Response: It looks like the reviewer might have reviewed the older version of the manuscript, because in the latest version, the coma is already in place where he or she suggested it be added.
**Reviewer:** L 25 and 26: PEP includes also the vaccines given on the days 3, 7, 14 and 28 and not just the one given at the first medical visit.

Response: We would like to thank the reviewer for this comment. We do agree that PEP also includes the vaccines given on the days 3, 7, 14 and 28 and not just the one given at the first medical visit. However, we would like to bring to the attention of the reviewer that in case of Category I, vaccine is not given on the first day. That is why reference is made to the category of the wound. This being the abstract, we could not include all those details.

**Reviewer:** L 33, 34 and 35: I suggest rephrasing; especially the last part, I do not think it does a lot of sense the way it is phrased, as it seems that those who do not own pets are included in the dog owners.

Response: We have changed the sentence, and it now reads as follows: “However, gaps in some respects related to knowledge about rabies were observed that requires awareness creation among residents of the study area irrespective of whether they own or do not own pets.”

**Reviewer:** L 42: I suggest adding a comma between “infection” and “generally”.

Response: Implemented.

**Reviewer:** L 47: I suggest removing the comma after “making the two”

Response: coma removed.

**Reviewer:** L 48: I suggest removing “While”

Response: Implemented

**Reviewer:** L 49: I suggest adding a comma after “annually”

Response: coma added

**Reviewer:** L 49: I suggest adding “infection” after “rabies”.

Response: infection added

**Reviewer:** L 53 and 54: I would suggest that you rephrase both, as it is especially true that symptoms of rabies can develop in case victims fail to obtain PEP and not so much that victims succumb to the disease after symptoms develop in case, they failed to obtain PEP.

Response: We note the reviewer’s concerns, however we are of the view that both statements are factually correct and are backed by references. What we convey here, is that once symptoms appear the patients usually succumb to the disease, and that the risk of developing symptoms is high if PEP is not obtained.

**Reviewer:** L 57: I suggest rephrasing. Although the development of symptoms will have much higher chances to occur in those who did not take PEP, an exposure does not always lead to infection and disease, even without the PEP. Therefore, I suggest changing it to “the loss of life of untreated victims might occur”.

Response: Thank you to the reviewer for this suggestion, the sentence has been changed and reads as follows: “Without proper health care, the loss of life of untreated victims might occur.”

**Reviewer:** L 61: I am not sure if “mass campaigns” is the correct English expression. Perhaps “mass vaccination of dogs against rabies”

Response: Thank you for picking this up. We have corrected it, by adding the word vaccination-it now reads “…mas vaccination campaigns…”

**Reviewer:** L 65: I would suggest changing the expression “developing countries” to “low-and-middle income countries”.

Response: We appreciate the suggestion of the reviewer; however, we would like to respectfully bring to the attention of the reviewer that the term low and middle-income country (LMIC) is often used interchangeably with "developing country" but LMIC refers only to the economy of the countries. We thus decided to retain developing countries.

**Reviewer:** L 75, 76 and 77: Perhaps it can be merged in one sentence

Response: Many thanks for the suggestion. We have merged the sentences, and they now read as follows:” This is supported by the recent increase in the number of laboratory confirmed positive animal cases in KZN, most of which have been confirmed in dogs [23, 44].”

**Reviewer:** L 80: Add the acronym “KAP” after “Knowledge, attitude and practices”. Also, do you consider the word “attitude” or “attitudes” to be the most correct?

Response: Acronym KAP has been added as suggested. However, “attitude” “attitudes” is used interchangeably in literature. Either of the two is correct, but we prefer attitude.

**Reviewer:** L 82: I suggest changing the “clinical signs and” to “clinical signs, “

Response: Change has been implemented (line 85)

**Reviewer:** L 84, 85 and 86: this sentence is not clear to me; could you please rephrase it?

Response: Noted and the following change has been made: “In addition, for prevention and control measures for rabies to be effective, knowledge of the community on how to handle a suspected rabid dog [10], plus measures that must be adopted when bitten by a rabid dog, need to be assessed.” (Lines 86-90).

**Reviewer:** L 87: Perhaps it can be added that KAP studies can be used also to identify gaps in knowledge, attitudes and practices, allowing for the design of targeted campaigns to fill those gaps

Response: Thanks for the suggestion, the sentence has been added (Lines 93-94).

**Reviewer:** L 93: Perhaps you can refer here that the study was conducted in Embo and Verulam, located in eThekwini District of KwaZulu-Natal province.

Response: The sentence has been modified to read as follows: “In view of this, the present study investigated the KAP in Embo and Verulam located in eThekwini District of KwaZulu-Natal Province of South Africa (Line 94-96).

**Reviewer:** L 110: In my opinion you should state here that just those with 18 years old or more were part of the target population. You can also justify the choice behind Embo and Verulam (or do it in the discussion).

Response: Line 117 -aged 18 years and older has been added. There have been rabies suspect cases in both Embo and Verulam in the recent past hence they were chosen for the study.

**Reviewer:** L 116: Can you please report the value of the sampling interval? Was it calculated by dividing the population size by the sample size?

Response: Line 126 the sampling interval has been mentioned. The sampling interval in systematic random sampling is determined by dividing the size of the target population and sample size determined using the sample determination formula.

**Reviewer:** L 118 and 119: Could you please explain a bit more of the sampling strategy here? Would you count the houses following a road? In which direction? And what did you do when the road finished? How was the first point chosen?

Response: How the first point was chosen is explained in Line 125 (i.e. first household is determined randomly). Here, you take the number of the first houses that make up the sampling interval and allocate a random number to each. One number is then selected randomly. Thereafter, the sampling interval is instituted. A reference (reference 40) is provided at the end of the paragraph, which describes the methodology in detail.

**Reviewer:** L 120 and 121: Were there any criteria – such as the household head – besides age of the selection of the respondent in each house?

Response: Yes, the criteria was that the household head was targeted, but in the absence of the household head, any adult (>18 yrs) was interviewed.

**Reviewer:** L 123: I suggest changing to “The sample size for the two study areas”

Response: Thanks for the suggestion- change has been implemented.

**Reviewer:** L 136: I suggest changing the comma after “data” to an “and”

Response: Thanks for the suggestion-implemented

**Reviewer:** L 138: Was there any process of translation and back translation or was it a simple translation from English to isiZulu?

Response: It was a simple translation from English to isiZulu during the interview.

**Reviewer:** L 146: Would the word “answer” be more appropriate than “questionnaire”?

Response: Thanks for the question. Since all the questions were reviewed for completeness, Questionnaire is more appropriate.

**Reviewer:** L 147: Was each question or answer that was coded?

Response: We have changed to “..each response was coded”

**Reviewer:** L 160: I suggest having “Knowledge score”

Response: Thanks for picking this up. We have added “score” as suggested.

**Reviewer:** L 161: are you talking about alpha or p value?

Response: Alpha and p value are used interchangeably.

**Reviewer:** L 170: were the models compared at each removal step?

Response: Yes, the models where compared - refer to the last paragraph of that section.

**Reviewer:** L 203 and 204: I do not understand well the beginning of the sentence. I suggest removing “Although in the minority”. I also suggest to change to “by a chemical substance (2.47%, xx/yy) or by insufficient…” . Furthermore, I can see in some cases where you kept “n=” and in others where you do not have the denominator. Please standardize all.

Response: The paragraph has been changed by removing some of the results, and now it read as follows: “Only 26.95% (n=207/768) of the participants were aware that rabies is caused by a virus. Some respondents thought that rabies is caused by a chemical substance (2.47%, n=19/768) or that it was caused by insufficient intake of feed and water (10.03%, 77/768).

**Reviewer:** L 217: I suggest changing “were okay” to “thought it was appropriate to get”

Response: Thanks for the suggestion-change has been implemented

**Reviewer:** L 218: I suggest changing the “;” to a comma

Response: We have taken note of the advice to change “;” to coma. However, we do not agree with the suggestion of the reviewer. A coma would be appropriate if we were listing items. In this case we are explaining what we mean by later.

**Reviewer:** L 220: add “where” after “are the places” and I suggest changing “injection” to “injections”

Response: Thanks for picking this up-change has been implemented.

**Reviewer:** L 226: change the parenthesis after “%” to comma, as previously done.

Response: Thanks for picking this up-change implemented.

**Reviewer:** L 230: I suggest removing the comma after “population”

Response: Noted -the coma has been removed.

**Reviewer:** L 233: I think it should be “were aware of the need”

Response: Thanks for picking this up-we have made the necessary changes

**Reviewer:** L 246: change the comma to point before the decimal number. Check the text please, because there are other situations where this happens.

Response: Thanks for picking this up-we have made the changes and reviewed the whole document to ensure it is corrected.

**Reviewer:** L 252 on: From this line on you reduced the number of times the percentage of respondents and absolute values are presented and used much more adjectives that express relative dimensions, such as “majority”, “small” or “some”. This contrasts with the previous section and seems to be a lack of consistency; furthermore, the stating of the numbers is much more precise and objective. For these two reasons I would prefer to see those numbers stated instead of the relative adjectives. Please always consider what should be presented in the text, as you do not need to report everything in written format and can make use of tables and graphs.

Response: Thanks for picking up the change from using percentages to using adjectives that express relative dimensions, such as “majority”, “small” or “some. This was a recommendation by one of the previous reviewers. Please note that this is the 5^th^ review of this manuscript. How you want it now, is how it was in the beginning. The change was implemented to reduce the amount of results reported in the text, which you also have raised. We are thus conflicted as what we should do given the conflicting suggestions.

**Reviewer:** L 254 to 267: same comment as previous

Response: Noted but this was done to reduce the number of times we cite results in the text. This was a recommendation by one of the reviewers during the 4^th^ review round. Furthermore, reviewer 4 in this review round has asked us to further reduce the results in the text. We therefore feel that putting back the results will be undoing what we did in the previous reviews.

**Reviewer:** L 269: I suggest removing the comma after “(303/768)”

Response: Coma has been removed

**Reviewer:** L 273: I would prefer to see a number instead of “under 30%”, as I do not know what this really means – it can go from 0 to 29.9%. As well, I think it would be better to see the number of those who have pets instead of those who have not.

Response: We note the concern of the reviewer, but this was done as part of minimizing repeating of the results in the text as advised by yourself earlier on and one of the reviewers during the 4^th^ review. Therefore, if we are to implement this suggestion, it will mean undoing what we did in 4^th^ review round.

**Reviewer:** L 274: Is the percentage 53.9% correct or meaningful? If I understand correctly, the entire number of respondents was used to calculate it, correct? As we are just talking about those who have pets – and therefore the only ones who could neuter them – could you please explain the rationale behind using the entire sample size as denominator?

Response: The rational of using the whole population to compute those who indicated that they had neutered their dogs, was that we wanted to compute it as a function of the proportion of the total study population and not just those who had dogs. However, we have adopted your suggestion and recomputed the proportions using the number of participants that had dogs.

**Reviewer:** L 275: I suggest adding “on” – “In response to the question on how often”

Response: noted-added.

**Reviewer:** L276: I think it would be better to present the precise numbers instead of relative adjectives.

Response: We note the concern of the reviewer. As we have stated above, this is a result of recommendations from previous reviewers who advised us to reduce on the results in the text. We are therefore torn between your recommendations and theirs. We have opted to leave it as it is.

**Reviewer:** L 279: I have doubts regarding the calculation of the percentage 59.77. The calculation used the entire number of respondents as denominator, correct? If so, it does not make sense to me. If part of the respondents did not have dogs, it could not be expected to get vaccination certificates from them. So, the percentage of those who had certificates should be in relation to those who have dogs, not to the entire number of respondents. As an example, if just 1% had dogs, but all of them had a vaccination certificate, using the entire number of respondents as denominator would result on stating that just 1% was able to produce a vaccination certificate, while in fact 100% of those who could produce a vaccination certificate had done it. But perhaps I am understanding it incorrectly.

Response: Thanks for your observation. We totally agree with your argument. We have adopted your suggestion and computed the proportions using the number of those who had dogs as the denominator.

**Reviewer:** L 282 and 283: Was this question asked to all the respondents?

Respondents: We have computed the proportions using the number that had dogs as the denominator.

**Reviewer:** L 284 and 285: Same question; Because it is written "their dogs", which leads me to think that this question was just for pet owners

Response: We have computed the proportions based on the total number that had dogs.

**Reviewer:** L 286 and 287: Same question; was this just asked to pet owners? If so, why is the total
number of respondents used as denominator?

Response: The questions were posed to all the respondents. But we agree that it equally makes sense to compute the percentages based on those who had dogs and not the total population.

**Reviewer:** L 294: I suggest removing the word “bellow”

Response: The word “bellow” is not in the last version we submitted. It is possible the reviewer had an older version of the manuscript.

**Reviewer:** L 295 to 298: this sentence is not very clear to me; could you please rephrase it?

Response: We have rephrased it and it now reads as follows: “In comparison to those who were aware that only laboratory results can be used to declare the animal positive to rabies, the number of people who indicated that animals that bite people after confrontation should be presumed to be rabid, was higher (35.42%, 272/768).

**Reviewer:** L 299 and 300: Perhaps "when asked what they would do in case of an encounter with a suspected rabid stray dog just 53.5 (411/768) said that they would..."

Response: Done
**Reviewer:** Table 4: I am not sure if presenting the percentages calculated with the total number of respondents as denominator are the best way to present the results from the questions directed to those who had pets.

Response: See responses to earlier similar questions.

**Reviewer:** Table 4: I do not understand to where does the word “throw” refers too in the last question

Response: throw as in disposing off by throwing in the nearest bush or open spaces etc.

**Reviewer:** L 307: I would prefer to see numbers instead of adjectives with relative meanings

Response: As indicated earlier, we opted for this to reduce on the results cited in the text. This was on recommendation of one of the reviewers in review round 4. We have this opted to leave it to the editor to advise on the same to avoid to and fro.

**Reviewer:** L 317: p without capital; throughout the text and tables (like table 5) sometimes you have the p value written in capital, sometimes not in capital. Please standardize.

Response: Noted and implemented.

**Reviewer:** L 322: On the value of the Odds ratio you use three decimal houses, while in the rest of the cases you use two; please standardize.

Response: Noted and implemented.

**Reviewer:** L 322: Should it be “three times as likely” or “three times more likely”?

Response: three times as likely is the correct way to present the odds ratio. If you say three times more, it means you have subtracted the odds ratio of the reference value from the odds ration for the variable. For example, in the case of AOR = 2.788, if you subtract one from 2.788, you get 1.788, then it becomes 2 times more likely.

**Reviewer:** Discussion – I would suggest removing redundant or not so relevant information or comparisons from the discussion. The authors compared their results with existing literature, but I think it would be beneficial to choose to mainly discuss results that can be of special relevance and to choose comparisons that are closer to the situation in KwaZulu-Natal Province, such as other studies from South Africa. Furthermore, the authors often present differences on findings, but without offering possible explanations for those differences. It is good to compare the results with other studies, but frequently I do not understand why those specific studies were chosen and, in many instances, I fail to understand what the main reasons for these differences could be.

Response: We note the reviewer’s suggestions, however we are of the view that choosing to mainly discuss results that can be of special relevance and to choose comparisons that are closer to the situation in KwaZulu-Natal Province, would make this study purely local. Our objective was to place this study in the context of available literature both locally and internationally. Secondly placing a study in the context of available literature, does not mean all differences can be explained. Where it is possible to explain the differences between our findings and those of other studies, we have done so. We do not think that just because we are not able to explain the observed differences, we cannot point out the differences between findings of our study and of studies done elsewhere.

**Reviewer:** L 344: I have some doubts on discussing of the demographics on a section separately, as done here. I think it would make more sense to be integrated in the discussion of the differences in findings as possible explanations for those findings. Could you please explain why did you opt for this way?

Response: We do take note of the reviewer’s suggestion. However, it is not clear what the reviewer wants us to do. Is reviewer suggesting that we integrate differences observed in the socio-demographic profile of respondents in our study and that of others in the discussion of other results? The suggestion is not clear, but the way we have discussed results here is how we have always done it past publications.

**Reviewer:** L 346 and 347: Could you please explain or give a reference why this age group is more active regarding pet management?

Response: We are not able to explain nor provide a reference as requested. As a result, we have rephrased the sentence and provided possible explanations for the observed results.

**Reviewer:** L 347 to 349: Could this be influenced by the way respondents were selected? As there is no information on how respondents within the household were selected, it is difficult to evaluate the reasons behind this finding. If the person available would be the respondent, could it be that woman were generally more at home than man, for example? Could you please explain your statement?

Response: This is an interest question. However, the target, was household heads. But in event that the household was not available, any adult that was available was asked to complete the questionnaire. This approached is commonly used in surveys. As to what influenced the gender distribution, we are not able to explain. We are simply presenting what we saw. It possible that majority of households are headed by women or there are more educated women in the areas than men and hence women are more comfortable to complete the questionnaire. We are not able to tell as this was outside the scope of the study. However, based on this question, this is something we will take into consideration in future studies.

**Reviewer:** L 354: Is it a high rate?

Response: Rate in Epidemiology has an element of time. Hence it is not appropriate to use rate in this instance.

**Reviewer:** L 355: Perhaps you can compare it with studies from South Africa or neighbor countries -
[https://protect.checkpoint.com/v2/r02/___https://pmc.ncbi.nlm.nih.gov/articles/PMC8929695/%23sec015___.YzJlOnVuaXNhbW9iaWxlOmM6bzo1YTIxMjY2OGUxZTY1ZjA3MGNkYjM5MzViZjMzZTJjNzo3OmJkOTM6YzY5OTZiNzMwZThmYzFlODM0ZjA1YjZjOThlODBjNWI1ZGQxM2UyNzY4MTQzYzEzMTA2MDljZTBkYzYxODllZDp0OlQ6Tg](https://protect.checkpoint.com/v2/r02/___https:/pmc.ncbi.nlm.nih.gov/articles/PMC8929695/%23sec015___.YzJlOnVuaXNhbW9iaWxlOmM6bzo1YTIxMjY2OGUxZTY1ZjA3MGNkYjM5MzViZjMzZTJjNzo3OmJkOTM6YzY5OTZiNzMwZThmYzFlODM0ZjA1YjZjOThlODBjNWI1ZGQxM2UyNzY4MTQzYzEzMTA2MDljZTBkYzYxODllZDp0OlQ6Tg),
[https://protect.checkpoint.com/v2/r02/___https://journals.plos.org/plosntds/article?id=10.1371/journal.pntd.0002059%23s3___.YzJlOnVuaXNhbW9iaWxlOmM6bzo1YTIxMjY2OGUxZTY1ZjA3MGNkYjM5MzViZjMzZTJjNzo3OmRhYTE6ZWZlOThiYmMyOWU0OWU0ZTMzMDU4NGJmOTk4MGMwZTg3MTFiYTg0MDQzNWI5NjFjZDZjYWRjNWZjMmViYzY5YTp0OlQ6Tg](https://protect.checkpoint.com/v2/r02/___https:/journals.plos.org/plosntds/article?id=10.1371/journal.pntd.0002059%23s3___.YzJlOnVuaXNhbW9iaWxlOmM6bzo1YTIxMjY2OGUxZTY1ZjA3MGNkYjM5MzViZjMzZTJjNzo3OmRhYTE6ZWZlOThiYmMyOWU0OWU0ZTMzMDU4NGJmOTk4MGMwZTg3MTFiYTg0MDQzNWI5NjFjZDZjYWRjNWZjMmViYzY5YTp0OlQ6Tg),
[https://protect.checkpoint.com/v2/r02/___https://www.authorea.com/users/889337/articles/1266757-knowledge-attitudes-andpractices-kap-of-rabies-prevention-among-communities-living-in-proximity-to-game-reservesin-the-eastern-cape-of-south-africa___.YzJlOnVuaXNhbW9iaWxlOmM6bzo1YTIxMjY2OGUxZTY1ZjA3MGNkYjM5MzViZjMzZTJjNzo3OjI5ZTE6NTYyZTQ1N2Q2OGU3ZDcyYTI5YTE1ZWYxYzVkOTYzMTNiNmJjMzBhYjY1NmIwOWQ0ZGQ5ODg1YTMxY2Y4ZmE4NTp0OlQ6Tg](https://protect.checkpoint.com/v2/r02/___https:/www.authorea.com/users/889337/articles/1266757-knowledge-attitudes-andpractices-kap-of-rabies-prevention-among-communities-living-in-proximity-to-game-reservesin-the-eastern-cape-of-south-africa___.YzJlOnVuaXNhbW9iaWxlOmM6bzo1YTIxMjY2OGUxZTY1ZjA3MGNkYjM5MzViZjMzZTJjNzo3OjI5ZTE6NTYyZTQ1N2Q2OGU3ZDcyYTI5YTE1ZWYxYzVkOTYzMTNiNmJjMzBhYjY1NmIwOWQ0ZGQ5ODg1YTMxY2Y4ZmE4NTp0OlQ6Tg),
[https://protect.checkpoint.com/v2/r02/___https://journals.plos.org/plosone/article?id=10.1371/journal.pone.0246103%23sec011___.YzJlOnVuaXNhbW9iaWxlOmM6bzo1YTIxMjY2OGUxZTY1ZjA3MGNkYjM5MzViZjMzZTJjNzo3OmVhNDY6ZGJkOTJiMTc4ODMzY2QxYTJkZTQ0ODRhNWNkZmZmMzhmZDY3MWQzZWQ1YzhlMmJhMzE2YzJiYzZlYzAyNjRmNjp0OlQ6Tg](https://protect.checkpoint.com/v2/r02/___https:/journals.plos.org/plosone/article?id=10.1371/journal.pone.0246103%23sec011___.YzJlOnVuaXNhbW9iaWxlOmM6bzo1YTIxMjY2OGUxZTY1ZjA3MGNkYjM5MzViZjMzZTJjNzo3OmVhNDY6ZGJkOTJiMTc4ODMzY2QxYTJkZTQ0ODRhNWNkZmZmMzhmZDY3MWQzZWQ1YzhlMmJhMzE2YzJiYzZlYzAyNjRmNjp0OlQ6Tg),
[https://protect.checkpoint.com/v2/r02/___https://journals.plos.org/plosntds/article?id=10.1371/journal.pntd.0011631___.YzJlOnVuaXNhbW9iaWxlOmM6bzo1YTIxMjY2OGUxZTY1ZjA3MGNkYjM5MzViZjMzZTJjNzo3OmE4ZDY6YWZjOWFlZGNkMmZjNWY1M2NjOGViYzNjODZiODg4NjQyMDdkNjlmNzRiMzJmYjg0ZTIzM2JjYmU0N2M5NWIxNjp0OlQ6Tg](https://protect.checkpoint.com/v2/r02/___https:/journals.plos.org/plosntds/article?id=10.1371/journal.pntd.0011631___.YzJlOnVuaXNhbW9iaWxlOmM6bzo1YTIxMjY2OGUxZTY1ZjA3MGNkYjM5MzViZjMzZTJjNzo3OmE4ZDY6YWZjOWFlZGNkMmZjNWY1M2NjOGViYzNjODZiODg4NjQyMDdkNjlmNzRiMzJmYjg0ZTIzM2JjYmU0N2M5NWIxNjp0OlQ6Tg),
[https://protect.checkpoint.com/v2/r02/___https://link.springer.com/article/10.1186/s12917-018-1604-z%23Sec6___.YzJlOnVuaXNhbW9iaWxlOmM6bzo1YTIxMjY2OGUxZTY1ZjA3MGNkYjM5MzViZjMzZTJjNzo3OjMwOTI6YWUwMWI3NmUzNzkzZGMzNWJjMDU5YTExNTczN2FiMjE5OWU4NTYzY2JkMTNiZDQxNTdlYWI3NDY5YzI5Zjk3Njp0OlQ6Tg](https://protect.checkpoint.com/v2/r02/___https:/link.springer.com/article/10.1186/s12917-018-1604-z%23Sec6___.YzJlOnVuaXNhbW9iaWxlOmM6bzo1YTIxMjY2OGUxZTY1ZjA3MGNkYjM5MzViZjMzZTJjNzo3OjMwOTI6YWUwMWI3NmUzNzkzZGMzNWJjMDU5YTExNTczN2FiMjE5OWU4NTYzY2JkMTNiZDQxNTdlYWI3NDY5YzI5Zjk3Njp0OlQ6Tg) - especially this one, from the same province

Response: we would like to thank the reviewer for the suggested references. We have considered them.

**Reviewer:** L 359: Perhaps “high dog ownership rate”

Response: We note the suggestion, and we are aware that the word “rate” is sometimes used to mean percentages, however in epidemiology, “rate” has an element of time. Which we did not consider in computing the percentage of people who own dogs. We have thus changed it to read as follows: “In light of the high number of people who own dogs…..”

**Reviewer:** L 362 and 363: Could you please explain why you chose these two articles for the comparison?

Response: Our objective was to place our study in the wider context based on available literature, particularly literature cited in the literature review. That is what informed our choice of articles that have been cited.

**Reviewer:** L 362 to 365: I am not sure if these paragraph makes much sense. You had not previously used these two references as comparison, or did I miss it? If you did not, in my opinion it would make more sense to point out the differences and then offer possible explanations for the differences.

Response: it is not clear what the review would like us to do in this case. Is reviewer suggesting that we remove the possible explanation we have provided and only indicate that findings of our study were different from those of the other two studies? We would appreciate if the review made it clearer more so why providing a possible explanation is not appropriate.

**Reviewer:** L 376: I suggest removing the comma after “study”

Response: done

**Reviewer:** L 381: Should it be “on the causes of rabies”?

Response: Noted and corrected to “cause of rabies”

**Reviewer:** L 391 to 395: Could you please explain if there was any particular reason to choose Ethiopia and the Philippines as comparison?

Response: Like indicated earlier on, the objective was to place our findings in the broader context of existing literature by citing studies done elsewhere such as countries in Africa and Asia. As indicated in the literature review, these are regions that also have a high rabies burden.

**Reviewer:** L 391: “show” instead of “shows”

Response: done

**Reviewer:** L 396: I suggest removing the comma after “rabies”

Response: done
**Reviewer:** L 397: for consistency, please add the numerator and denominator.

Response: done

**Reviewer:** L 397: I suggest to change “Which” to “This is”

Response: done

**Reviewer:** L 401 to 405: Perhaps you can cut the first sentence of the paragraph and move the second one to the previous paragraph, following the sentence that finishes with "low number of respondents (11.46%)".

Response: by “cut the first sentence of the paragraph” , we are not sure if the reviewer meant that we should delete the sentence.

We have nonetheless moved the second line to the previous paragraph.

**Reviewer:** L 406 to 421: Are the comparisons with these studies relevant? Are you trying to explain why the differences exist, or just reporting them? In case of just reporting them, is this relevant for the objective of this study

Response: The intention is to show how our results compare with what others have reported elsewhere, and in addition (as explained earlier), to place our findings in the broader context of existing literature locally and internationally.

**Reviewer:** L 422: Please change “diseases” to “disease”

Response: done

**Reviewer:** L 430: Again, I fail see how this comparison would be important. As I understand it, what is important is that there are people who do not know that they should take the vaccine immediately and therefore there would be important to have campaigns, for example, focused on this. Could you please explain why do you think that having these comparisons is important?

Response: We take cognisant of the reviewer’s insistence on this issue, but we would like to respectfully differ. A discussion cannot be without comparison and placing of the findings of the study in the broader context of existing literature. We have had manuscripts rejected by international journals on the premise that they are too locally focused and hence unsuitable for international journals.

**Reviewer:** L 450: Please change the comma on the percentage to a point

Response: Done

**Reviewer:** L 507 to 512: This is a good point of comparison between the results in different studies.

Response: Note, and many thanks to the reviewer.

**Reviewer:** L 547 to 551: It would be good to have data supporting these statements

Response: We note the reviewer’s concern. However, it is not clear what the reviewer would like us to do. Nonetheless, we are of the view that reasonably conceived and logically presented, authors' speculation can add to the value of a research report. Speculation or a suggestion can serve as a pointer to where further research is needed.

**Reviewer:** L 594 to 599: If I understood correctly, only 559 respondents had dogs, correct? If 459 were able to produce a vaccination certificate, this means that 82% of the dogs were vaccinated. Or did I understand it incorrectly? If it is as I understood it, could you please re-write the paragraph?

Responses: Yes the reviewer is correct that 559 had dogs. We do agree that 82% among those who had dogs during the study period were able to produce a vaccination certificate. We have considered the reviewer’s suggestions and made the relevant changes.

**Reviewer:** L 646 to 648: Here is the same problem with using the entire sample; if some of the
respondents do not have pets, it does not make sense to include them in the calculation.

Response: We have adopted the reviewer’s suggestion and recomputed the proportions of respondents who presented their dogs in vaccination campaigns using the number of respondents that had dogs as the denominator.

**Reviewer:** L 651 to 653: I seem to me repeated, as you had already referred this before.

Response: We note the reviewer’s concern. It is not a repetition. However, we have rephrased it to read as follows: “The number of people who indicated they would consistently present their dogs for vaccination in the present study was high, which could explain the more than 70% vaccination coverage recommended by WHO [7] that was recorded in the present study.

**Reviewers:** L 690: Several studies point to education as a factor. Other studies included considerably more factors in the analysis. In case you can think of any reasons, I would suggest that you discuss the reason behind education not being a significant factor in this study and how other potential factors could also have an influence in levels of knowledge.

Response: We do take note of the reviewer’s comment to the effect that several studies point to education as a factor. Respectfully we cannot think of any reason as to why education in our study was not a significant predictor, while in other studies it was. Even a thing like the type of analysis could give different results. For example, it is possible that at univariate level a variable could be significantly associated with the outcome, but when confounders are accounted for in a multivariate model the variable ceases to be significantly associated with the outcome.

**Reviewer:** L 731 to 740: Unless stated otherwise, I think the limitations should be included in the discussion.

Response: We note the reviewer’s suggestion. We are used to presenting the limitations as a separate section. This is because many journals now ask you to place this “limitations section” at the very end of your article. We stand to be corrected, but we are of the view that this is how PLOS one recommends that manuscripts be structured. In view of this we have respectfully opted to leave the section as it is.

**Reviewer:** Figure 2: I suggest changing the title of the y axis to “Percentage of respondents” as in Figure 3.

Response: Thanks for picking this up-correction has been effected.

**Reviewer:** Figure 2 and 3: could you pleas explain why is the color of the text different between both graphs?

Response: We note the reviewer’s comment, but unfortunately, we are not able to make out what the reviewer is referring to. Has it got to do with the fact that the captions are bolded? We kindly request the reviewer to help and provide more details of what he or she is asking us to attend to. We do not see any difference in the colour of the text referred to.

**Reviewer:** Figure 1: I do not understand the title of one of the bars “Equine and…”

Response. The two bars show the proportion of respondents who indicated that horses (Equines) and pigs are susceptible to rabies. It should be clear now in the new figure.
