# Supplementary material for: Knowledge, attitude, and practices towards rabies: A community survey in selected areas of KwaZulu-Natal Province, South Africa
Source: PLoS One. 2026 Jul 9;21(7):e0352279. doi: 10.1371/journal.pone.0352279 (PMC13349125; doi:10.1371/journal.pone.0352279)
Supplement: Attachment — Submitted filename: Rebuttal letter.docx [file pone.0352279.s012.docx]

Reviewer #6: Dear authors, thank you for the answers to my queries and for editing the manuscript following the suggestions. I believe it has improved and, apologizing because I know that it has been a lengthy process of review, I still have some comments, questions and suggestions. I am mainly concerned with the discussion and how lengthy it is.

Response: Thanks for the positive feedback. We value your comments and believe they helped improve the manuscript. We have tried to cut down on the discussion by removing the opening paragraph, which is a summary of the results and also removed the discussion on the socio-demographic characteristics of the study participants or respondents.

L 21: I suggest to remove the comma after “26.95% (207/768)”

Response: comma has been removed.

L22: I had suggested to change the comma to before “Which”, not to after

Response: thanks for pointing this out-we have made the relevant changes.

L 23-25: Thank you for the explanation. I understand now the idea of the sentence; to make it clearer I would suggest having it as: “which can include, depending on the category of the bite wound, wound washing, a dose of human rabies immune globulin (HRIG), and rabies vaccine given at the time of the first medical visit.”

Response: Many thanks for the suggestion. The sentence has been changed accordingly

L 27: I suggest removing either “of” or “of respondents”. It does not make much sense to me to have “much dog owners of respondents”.

Response: change has been implemented and “of respondents” has been removed.

L 29-32: Thank you for rephrasing it; I would suggest changing “that” to “,which”

Response: thanks for the suggestion-the change has been made and “that” has been changed to “which”.

L 57: I suggest changing “is” to “are”, as the sentence starts with “Details” and remove “in details”, as it is already at the beginning of the sentence.

Response: Thank you for picking this up- we have made as suggested.

L 64: Perhaps change “mortalities” to “mortality”.

Response: change has been made with “mortalities” changed to “mortality”.

L 65: I suggest adding “the” before existence

Response: Thank you for the suggestion. The change has been implemented.

L 75: I do not know if this is also present on the original version, but the pdf version I have has “Click or tap here to enter text.” written in the middle of this line.

Response: It was a reference holder, and it has been deleted.

L 71-77: Throughout the text province is sometimes with capital P and sometimes without capital (like also in lines 92 and 107). Please standardize.

Response: Where “province is a name, we used capital “P”, but where it is not used as a name, we use small “p”.

L82: There are two commas after “effective”

Response: Noted and one coma removed

L 82: I believe it should be “prevention and control measures of rabies”, instead of “prevention and control measures for rabies”.

Response: Change has been made as suggested

L 86: I would suggest changing the second “the disease”, for “with it”.

Response: change has been made accordingly

L 88: Perhaps you can add “towards rabies” after “KAP”

Response: the word “towards” has been added as recommended.

L 89: I might be wrong, but it seems to me that there is a double space after “located” Response: The reviewer is correct there was a double space, and a change has been made.

L 188: Thank you for the explanation. Yes, I understand that the first point was selected randomly, but I think it would be useful to have a more specific explanation on how the point was selected randomly (ex: GPS point randomly generated, list of households, etc.). In case someone would like to repeat the study, it would be useful to explain clearly and in detail all the steps which will allow the repetition. Did the authors have the houses numbered like in reference 40 and drew the first number randomly? If so, I understand which directions were taken after the first house. If not, I would like to know please how you decided on the direction to take. In case the method was the same as in reference 40, I would suggest adding something like “we followed exactly the same approach as in the study by Kumar et al.”, just to make it more explicit that exactly the same approach was followed and had the houses numbered. Personally, when I read an article, I like to fully and clearly understand all the methods followed.

Response: Thanks for raising this up again-we had over assumed I guess regarding the procedure for systematic random sampling. We have thus provided more details on how we executed systematic random sampling.

L 122: Just to confirm, in case the household head was not present, the interviewers would just ask for whoever would like to or would be available to answer the questionnaire, is that?

Response: Not really- the person had to be resident in the household.

L 155: I think it would be useful to describe what the threshold level was to consider each respondent knowledgeable or not knowledgeable. I assume the knowledge score would be calculated as the proportion of the score obtained over the total possible points obtained, correct?

Response: you are correct - the knowledge score was calculated as the proportion of the score obtained over the total possible points that could be obtained. You are also correct on the issue of establishing the threshold. The following adjustment has been made on the data analysis: “The knowledge score of each respondent was computed and presented as a percentage. Thereafter, a binary variable was created from the knowledge score using 60% as the cut-off for being knowledgeable (≥ 60%) or not knowledgeable (< 60%) using the criteria described by Alam et al. (2021).”

L 164: I respectfully disagree, as I consider alpha and p value different and should not be used interchangeably. The value of alpha is the threshold defined by the authors as the level of significance, and therefore the defined threshold for rejecting the null hypothesis, while p is a probability resulting from the test done. Therefore, variables will be included from the univariable model to the multivariable model if p ≤ α (in this case, α= 0.2).

Response: Thanks for the explanation.

L 209: Yes, this formulation would be acceptable from my perspective. I suggest to keep the precise numbers for results considered more relevant and refer to the table in the case of those not so important.

Response: Many thanks for the suggestion

L 218: On (5.34% (41/768) was used a second parenthesis (before 41), when in remaining similar cases, a comma was used. Please standardize.

Response: Noted with thanks – it was a typo, otherwise a comma is preferred. Consequently, changes have been made in line with the suggestion.

L 221: I suggest adding “where” after the word “places”

Response: thanks for picking this up-the word where has been added.

L 253 on: I understand the effort to reduce the length of the results. I also believe that this is needed. Perhaps citing numbers for important results and referring to the ones considered less important to the tables might help.

Response: thanks for the suggestion- we have reviewed the manuscript to suit the suggested style.

L262: The percentage seems to be in a different font size

Response-thanks for picking this up-we have made changes on the font size

L 281: 147/559 is 26.2%. Why is the text just “twenty percent”?

Response: Thanks for picking this up- we have changed it to twenty six percent (26.2, 147/559)

L 279 on: I am puzzled by the number 559 used when referring to “only dogs” in many of the paragraphs. From what I understand, 559 would include houses that could have just a cat, correct (as it seems to me from the formulation on the table “Own a pet dog and/ or cat”) ? Therefore, it does not seem correct to me to constantly refer uniquely to dogs when the number 559 can include also households just with cats. Could also be interesting – if the number of households just owning cats is considerable – to see the differences between the percentage of cats and dogs vaccinated. I suppose the percentage of dogs vaccinated is higher.

Response: The reviewer is right that the 559 could include houses that could have just a cat. Dog and pet in this study is used interchangeably. We did not aim to distinguish between cats and dogs so we did not investigate differences in percentages of cats and dogs that were vaccinated. It is something we have noted for future studies.

L304: Please change “no” to “not”

Response: Change implemented

L 306: I think the “rabid” would be more appropriate than “rabies”

Response-we agree with the suggestion and change has been made.

Table 4: Why is the sum of the answers to question “How often can you vaccinate your dog?” not 100%?

Response: thanks for picking this up-we had wrongly calculated the percentage that said Don’t know as 19.14% when it is 26.30%. Correction has been made.

Table 4: I suggest that to mark in thicker the lines separating the answers to each question, as it will make reading the table much easier.

Response: thanks for the suggestions, but we think that the journal will do that when they format the paper, should it be accepted for publication.

Table 4: I understand the 27.21 percent value for those who do not have a pet on the table. As the sum of all is above 100%, it seems a bit strange at first sight, but I understand why is like that. Personally, I think that if you opt to remove that line from the questions just asked to pet owners it would be fine, but it is also ok from my side if it stays like that.

Response: thanks for the suggestion-we agree that removing the line removes the confusion, and that is what we have opted to do.

L 311-312: Could the authors please indicate in which table it is possible to see the percentage figure?

Response: we have clarified by changing the sentence to read as follows: “As shown in Fig 3, the overwhelming majority interviewed indicated that animals should always be vaccinated to avoid rabies outbreaks.”

L 338: As in the line above is “rabies is caused” I would suggest to change “was” to “is”

Response: many thanks for picking it up-we have made the change as suggested.

L 336-349: It seems mainly to be a summary of the results. Being the paper lengthy, unless it was recommended by another reviewer or you really have a strong reason to keep it, I would just remove these lines, to avoid repetition and reduce the length. As pointed by other reviewers

Response: you are right the whole section is a summary of the results. A couple of journals we have published with in the past prefer starting the discussion with a summary of the main findings. However, in the interest of reducing the length the paper, we have decided to remove the section as advised.

L 351-353: Could you please explain why do you do this statement? Being that you do not state any specific criteria for the selection of the respondents, why do you associate the highest participation of this group to being more related to the household pets? Could there be other reasons, such as visiting time or day, which could have influenced this?

Response: The criteria used in selection of respondents was random sampling. The purpose of random sampling is to get a study population that is a reflection of the target population. In view of this, we assume that the findings of this study is a reflection of the target population. There is evidence as per the reference we cite to the effect that gender plays in a role in matters of caring for pets, albeit that our results contradict what others have reported elsewhere.

L 360-362: Could this difference be due to differences in occupation? For example, in areas inhabited mainly by farmers whose farm is close to the house or during non-farming season could be that males are more at home than in other seasons or occupations.

Response: thanks for this thought-provoking suggestion. However, the two areas are not farming communities and hence the thinking might not apply. Most communities in SA, including rural areas close to cities do not depend on farming but travel to the cities for employment and return in the evening. It is nonetheless something we could give thought in future studies.

L 351-383: I understand my previous comment regarding the section “Demographic profile of respondents” was not very clear. I completely understand the will of the authors to place the study in the context of available literature. On my side, I do not see a big problem with having a more local focus. Results of KAP surveys and identified gaps can be dependent on so many factors, which are mainly locally based. Furthermore, as gaps are local, interventions to address these gaps will also be locally focused. I tend to believe that results from a KAP study are in a high degree related to the local context. We cannot identify a gap between a certain region or country and automatically assume that the same gap will be present in a very different country and region. KAP constructs are highly related to local contexts and therefore, as a tool that can be used as a first step to devise interventions to control rabies in that specific context, the results of the KAP survey are mainly locally dependent and focused. KAP methodology – this is, how to carry out or improve KAP surveys – could be something more relevant on a global context eventually. In my opinion, KAP results are generally very locally dependent and therefore its importance is mainly local. Surely there can be comparisons with other regions and countries, and in this situation my opinion is that it is more valuable to compare more similar situations to understand what the reasons could be in case there are differences than just comparing results from very different contexts. Overall, I would suggest cutting several of the comparisons with other countries and focusing more on the relevance of the results to rabies control in the province. Surely, comparisons can be relevant for certain results and in that case, I would suggest using more similar situations to the study area. That is why I suggested some of the references in my previous comment.

Respectfully, I fail to see the relevance of a considerable part of the text within the section “Demographic profile of respondents”. Unless these differences could be used in the discussion of different findings in terms of KAP levels, or practices, or attitudes, or specific knowledge, etc. For example, knowing that rabies is a viral disease, could it be that in the study carried out in Kombolcha there was a greater proportion of correct answers because the level of education could also be higher (at least the percentage of respondents with college diplomas is higher than in this study the percentage of respondents who finished tertiary education). This is what I meant by integrating the demographic profiles of respondents on the discussion about differences in KAP instead of doing separate comparisons for the demographic profiles. For example, is it very relevant to state uniquely that in this study the majority of the respondents is Christian and, in another study, done in Ethiopia most of the respondents was Muslim? Or comparing the percentage of respondents who owned dogs compared with the percentage of dog owners from a study in Cameroon, done on an area where a considerable part of the population is Muslim and therefore, who religious attitudes might have an impact on having a dog or not? As I said above, in my opinion the manuscript is lengthy, and I believe that referring uniquely to some of the different demographic characteristics when they can be potential factors influencing the differences registered in results would be more beneficial, instead of having an entire section of differences between the demographic characteristics of this study and some other studies done in another country.

Response: The reviewer is of the view that KAP studies should have a local focus and not be placed in the context of available literature. If the target is the local audience, then we would have gone that route. Otherwise, we do not think that a study with a local focus is of any benefit to the wider body of scientists and is not likely to be accepted by international journals. This has been our experience over the years.

We respectfully disagree with the reviewer on the issue of summarising the demographic characteristics of the study population. Our disagreement is premised on the fact that in an empirical manuscript, a more detailed set of demographic information (and any other background information) about the sample and context of the study are relevant for understanding and interpreting the study's findings, including its generalizability especially in quantitative studies like our study and limitations thereof. For example, the reviewer asks if it is very relevant to state uniquely that in this study the majority of the respondents is Christian and, in another study, done in Ethiopia most of the respondents were Muslim. The answer in our view is yes, it is important and necessary. The reason being that both the study area in the present study and the one in Ethiopia are endemic to rabies, and in that way they are similar. So providing a detailed report of the demographics and how they compare to those of a study done in another area is relevant to understanding the findings of the study and generalizability and the limit thereof. The reviewer suggests integrating the demographic profiles of respondents on the discussion about differences in KAP instead of doing separate comparisons for the demographic profiles. We are of the view that the two are not mutually exclusive-there can be a detailed description of the demographics for purposes explained above, and at the same time integrate aspects of the demographics in the discussion of the knowledge, attitude and practices.

However, the above notwithstanding, we have decided to remove the section that discusses the demographics and only left the summary of the results of the demographics. We have also endeavoured to integrate the demographic profile of the respondents in the discussion as recommended by the reviewer.

L 384: I suggest changing to “Knowledge rabies disease”

Response: we have changed it to “Knowledge of rabies disease” instead of “knowledge rabies disease”

L 423-434: In this case the authors compare the study results with two studies from Ethiopia, and even from the same region – both studies were carried out in different areas of the Amhara region. And they present very disparate results in terms of the percentage of respondents following bite wound washing practices. Probably due to different demographic, cultural or educational characteristics between both areas. But that is precisely why I have difficulties in seeing a high value of constant comparison of all the results with the results of a few studies done in a few other countries, some of them probably quite different from South Africa. This leads to a discussion with 15 pages, which I consider to be quite lengthy and more difficult to read.

I understand the will of the authors to place the results in a broader context, but as stated at the end of the introduction, KAP studies can be useful to identify gaps and to inform information campaigns or other control interventions. Therefore, I believe that a discussion more focused on the meaning of the results for the control of rabies in KwaZulu-Natal, with a couple of comparisons with the results of other studies, would be of higher value for the overall manuscript. I believe that for a policy maker or for someone wishing to implement control activities, a focus on what the results might mean to KwaZulu-Natal and some of the aspects that might need to be address would be more important and might get lost in the middle of so lengthy constant comparisons with results from a few studies from countries such as Ethiopia, Cameroon or Morocco.

Response: From the passage the reviewer refers to (Lines 423-434), it becomes clear that South Africa and KZN particularly is not doing well in terms of educating the populace about how to handle rabies when compared to other areas that are endemic for rabies. That is the point we drive home in this passage and has implications for control. The challenge from these findings is that how can you control the disease when you are lagging behind other countries on some basics things about the disease? Another person would have probably had another focus and would have presented the same findings with a different focus. But that is not to mean that our approach is wrong. On the contrary, unlike the reviewer’s view that our findings are of no benefit to policy makers, we think that policymakers can draw on our findings and implement policies that will ensure that there is an increase in knowledge of a thing as basic as washing the wound as the first aid to be administered by dog bite victims. Realising that they are lagging behind other countries can be a motivating factor. That is the value we have in comparing with findings of other studies.

We do a lot of explaining of the meaning of the results for the study area. For example, the paragraph below clearly shows this and there are many such throughout the manuscript. We think the best motivation for change is when you compare yourself to others.

The figure of 16% in this study of the proportion of people who considered rabies to be a fatal disease is much lower than what was that reported by Bihon et al. [6] and Abedela and Teshome [2], who observed that 84.6% (325/384) and 82% (123/150) of people, respectively, were aware that rabies cannot be cured once clinical signs appear. The fact that only a few respondents in this study were aware that rabies is not treatable is significant and points to the fact that people are likely not to seek medical care early following a dog bite. This kind of information must be included in campaigns to educate the community about the seriousness of the disease rabies.

L: 433: While sometimes there are two suggestions for the translation of the name of the city (Gondar or Gonder), “Gondar” would be the most commonly used and this is also the form the article title used. This instead of “Gondor”.

Response: We agree that the correct name is Gondar. Many thanks for picking it up.

L 447: I suggest not having a paragraph here.

Response: Paragraph removed.
