# Supplementary material for: Knowledge, attitude, and practices towards rabies: A community survey in selected areas of KwaZulu-Natal Province, South Africa
Source: PLoS One. 2026 Jul 9;21(7):e0352279. doi: 10.1371/journal.pone.0352279 (PMC13349125; doi:10.1371/journal.pone.0352279)
Supplement: Attachment — Submitted filename: Rebuttal Letter -Revision 8.docx [file pone.0352279.s013.docx]

## Response to reviewers

**Reframe the Title of Table 5 to make it more scientific**

**Response:**

The title has been reframed and now reads as follows: “: Results of the analysis of factors correlated with being knowledgeable about rabies”

**Add years in bracket where Age groups are given in tables.**

**Response:**

“Year” has been against age groups in tables

**Check for consistency in mentioning p value in main text, tables and in abstract**

**Response:**

*p* value has been corrected to ensure consistency.

**The table or its caption should explicitly state that only the variables retained in the final model are shown.**

**Response:**

A footnote has been added below the table to reflect that only the variables retained in the final model are shown.

**The abstract mentions a p-value of <0.005 for dog ownership, but Table 5 provides the more precise value of <0.0001. The abstract should be updated to reflect the specific significance found in the final model.**

**Response:**

The abstract has been updated to reflect the specific significance found in the final model.

**Reviewer #7:**

**I commend the authors for their effort in improving the article through multiple revisions. I encourage them to further address the remaining shortcomings and refine the manuscript to meet academic standards. While most of the reviewers’ suggestions have been incorporated, some issues remain unresolved. My general comments pertain to the discussion section, particularly the use of phrases and sentence construction. Several sentences could be tightened for clarity and grammatical accuracy, for example, by avoiding repetition, correcting fragments, and smoothing transitions. While the manuscript is generally understandable but requires refinement into academic style. As it is not feasible to pinpoint all instances, the authors are advised to conduct a thorough review and implement corrections. I would advise authors to consider seeking professional proofreading support so that the review process is completed in time.**

**Response:**

We have reviewed the discussion section, with the use of phrases and sentence construction to try and tighten clarity and grammatical accuracy. We also engaged the professional proof English language editor to assist in this regard.

**Line 54-55: Without proper health care, the loss of life of untreated victims might occur.**

**It may be written as: In the absence of appropriate healthcare interventions, untreated individuals are at increased risk of death.**

**Response:**

We appreciate the reviewer’s suggestion, and the sentence has been change to read as suggested, and the sentence now reads as suggested by the reviewer.

**Line 56-58: Details of the treatment prescribed for victims of dog bites to prevent the development of the disease rabies are described by Bishop et al…**

**The word disease rabies is not correct. Pl check for these words and correct it accordingly.**

**It can be written as: “Details of the treatment prescribed for dog-bite victims to prevent the development of rabies have been described by Bishop et al.”.**

**Response:**

We have rephrased the sentence, and it now reads as suggested by the reviewer.

**In addition, WHO Expert Consultation on rabies, third edition 2018 provides clear guideline on prevention of rabies in humans.**

**Response:**

The sentence has been changed accordingly as advised by the reviewer.

**Line 77-78: Knowledge, attitude and practices (KAP) studies are significant in obtaining**

**information on the subject of concern. What is significant?**

**It can be written as : Knowledge, Attitude, and Practices (KAP) studies constitute an important methodological approach for acquiring insights into the subject of concern.**

**Response:**

Thanks for the suggestion-we have rephrased the sentence to read as recommended by the reviewer.

**Line 274-277: The expression “pulling down a dog” conveys meaning or may be understandable, but it does not reflect an academic style of writing. I have commented this in my previous review but was not corrected.**

**It should be replaced with either “euthanizing a dog suspected of having rabies” or “destroying a dog suspected of rabies infection”. Please change this in the discussion section if mentioned.**

**Response:**

Thanks for the recommendation. The phrase “put down a dog”, has been rephrased and wherever it appears in the document has been corrected. It has been replaced with “euthanizing” or “destroying the dog”

**Line 353-356- This is a serious public health given that washing ….**

**Please qualify after public health. ---is it public health concern or public health issue**

**….virus from gaining entry to the victim and …..**

**It should be the victim’s body**

**Please restructure the sentence.**

**Rephrase**: the sentence has been restructured to qualify after public health by adding “concern” after public health.

**Line 367=368 -- if we are to eradicate rabies-related deaths.**

**Please change as: to prevent death**

**I have pointed out in my previous review that the word "eradicate/eradication" is used inappropriately in the manuscript. There is a difference between eradication, elimination, prevention and control. It will be difficult to eradicate rabies virus. We should be aiming to eliminate dog-mediated human rabies deaths. Please replace the word eradication with elimination or prevention or control wherever appropriate.**

**Response:** The reviewer’s concern is noted and wherever the word eradicate appears it has been replaced with “prevent”

**Line 372-378 – the word other species. Please add …other species of animals or wildlife**

**….extension to communities ---it should be extension education programe or awareness program.**

**Responses:**

The sentence has been restructured by adding “other species of animals” as recommended by the reviewer.

**Line 379-390- …The high number of respondents who were aware of the importance of vaccinating dogs to control rabies is good news. The expression good news is clear but is not a formal expression.**

**It can be written as: The high number of respondents who were aware of the importance of vaccinating dogs to control rabies is encouraging**

**Response:**

The recommendation has been adopted, and the sentence now reads as follows: “The high number of respondents who were aware of the importance of vaccinating dogs to control rabies is encouraging.”

**Line 380-382. Vaccination of pets against rabies creates a barrier to diseases shared among pets and humans, given that they occupy the same space. …The word that creates a barrier is correct but slightly vague. In public health writing, terms like “reduces transmission” or “prevents spillover” are clearer.**

**It can be written as: Rabies vaccination in pets enhances herd immunity and disrupts viral transmission within animal populations and from animals to humans. OR**

**Vaccination of pets against rabies enhances herd immunity and helps interrupt the transmission chain of the rabies virus among animals, as well as between animals and humans.**

**Response:**

Thanks for the suggestion. The sentence has been restructured by adopting the recommendation by the reviewer.

**Line 394-396 : the word welcome observation is not an academic style of writing, replace with an encouraging finding**

**It can be expressed as: The very high number of respondents (87.24%) who preferred to be treated at a human clinic or hospital after exposure to a dog bite in this study is an encouraging finding**

**Response:**

The sentence has been changed to read as the reviewer has recommended.

**Line 416-418: incomplete sentences. Please complete the sentence**

**Response:**

The sentence has been rewritten and now reads as follows: “This means that dogs presenting with paralysis are likely to be overlooked as rabies suspected cases. Therefore, if a rabid dog showing paralytic signs, was to bite a person, such a person is likely not to seek PEP, which increases the risk of contracting rabies among the respondents.”

**Line 432-434: the words odd behaviour should be replaced with abnormal behaviours or unusual behaviours.**

**Maybe it can be expressed as: The high number of respondents who favoured isolating their animals or placing them under quarantine if they displayed abnormal behaviours, such as biting someone, is a very significant finding.**

**Response:**

The reviewer’s suggestion has been adopted.

**Line 437-438: putting down the dog ; and plus many . please correct these.**

**Response:**

The phrase putting down the dog has been changed through out the document.

**Line 446-451: Please revise**

**Lune 455: ….if the goal of eradicating dog mediated rabies death in humans is to be attained.**

**The goal is elimination of dog-mediated human rabies death by 2030, and not eradication. Please change**

**Response:**

The sentence has been restructured, and eradicating has been replaced elimination.

**Line 446-459: …..which according to WHO is the minimum vaccination coverage needed to prevent rabies outbreaks in humans.**

**It is not about rabies outbreak in humans but increase herd immunity in dogs and then prevent rabies virus transmission within dogs or animals and prevent transmission to humans. Please correct this.**

**Response:**

Thanks for the suggestion-we have made the relevant changes to reflect the reviewer’s suggestion.

**Line 466-470: please correct**

**Response:**

**The paragraph has been reviewed and now reads as follows:**

While in the study by Shirsuphal [41], 84% (56/67) of participants did not let their dogs to go outside the homestead, very few participants in the present study indicated that they kept their dogs in an area that is fenced off and does not allow dogs to go outside the compound. This observation is a serious public health concern, because when domestic dogs are let to roam freely, they frequently encounter stray animals that might rabid. This heightens the risk of these dogs contracting the rabies virus. Therefore, confinement of dogs to minimize domestic dogs encountering stray dogs or wild animals in the study area, needs to be improved on.

**Line 474: Filipino should be corrected as the Philippines**

**Response:**

The change has been implemented as suggested by the reviewer.

**Line 481-486: the word problematic is not an academic style of expression and also the sentence is long.**

**It can be written as: Given that the study area is endemic for rabies, it is concerning that some people did not present their dogs for vaccination during each campaign. This may be due to individuals being unavailable because of work or other commitments, while others were unable to handle their dogs and therefore did not present them for vaccination.**

**Response:**

The sentence has been modified by adopting the suggestion by the reviewer.

**Line 495-499: ….Much of the …..this sentence is not structured correctly.**

**I think it can be written as : “Although the findings are encouraging, more effort is required to ensure that carcasses of animals suspected of rabies are submitted for laboratory confirmation.”**

**Response:**

The reviewer’s suggestion has been implemented and now the sentence reads as suggested by the reviewer.

**Line 517-518: The study area has attained over 70% vaccination coverage recommended by the WHO to minimize the risk of rabies outbreaks in humans/communities.**

**As stated above, 70% vaccination coverage in dogs is to break the rabies virus transmission chain within dogs and prevent transmission to humans and not rabies outbreaks in humans.**

**Response:**

**The sentence as been changed accordingly and now reads as follows: “**The study area has attained over 70% vaccination coverage, which according to the WHO is needed to break the rabies virus transmission chain within dogs and prevent transmission to humans.”

**Line 523-524—The role of measures Please restructure the sentence**

**Response:**

The whole paragraph has been restructured and now reads as follows: “The identified knowledge gaps include poor knowledge of the cause of rabies, how one can protect oneself from contracting rabies, precautionary measures to adopt following exposure to rabies and how to protect susceptible animals from being exposed to rabies. Measures such erecting fences around homesteads to restrict animal movements and neutering of pets to control dog population need to be emphasized during awareness programs.”

We would like to thank the reviewers for all their input that have helped improve on the paper. We have attended to all the comments and suggestions and how we responded is outlined above. In addition, the manuscript was sent for editing by a professional English language editor. In the version with track changes the words in blue is what the editor suggested and the paragraph highlight yellow are paragraphs on which we as authors worked on while implementing the comments and suggestions from reviewer 7 and the editor.
