# Supplementary material for: Knowledge, attitude, and practices towards rabies: A community survey in selected areas of KwaZulu-Natal Province, South Africa
Source: PLoS One. 2026 Jul 9;21(7):e0352279. doi: 10.1371/journal.pone.0352279 (PMC13349125; doi:10.1371/journal.pone.0352279)
Supplement: Peer review history

# Peer review: Decision Letter 0

**DOI:** 10.1371/journal.pone.0352279.r001

**Authors:** A. K. M. Anisur Rahman

7 Nov 2023

PONE-D-23-27745

Knowledge, attitude and practices towards rabies: A community survey in selected areas of KwaZulu-Natal Province, South Africa

PLOS ONE

Dear Dr. Oguttu,

Thank you for submitting your manuscript to PLOS ONE. After careful consideration, we feel that it has merit but does not fully meet PLOS ONE’s publication criteria as it currently stands. Therefore, we invite you to submit a revised version of the manuscript that addresses the points raised during the review process.

Please submit your revised manuscript by Dec 22 2023 11:59PM. If you will need more time than this to complete your revisions, please reply to this message or contact the journal office at plosone@plos.org. When you're ready to submit your revision, log on to https://www.editorialmanager.com/pone/ and select the 'Submissions Needing Revision' folder to locate your manuscript file.

Please include the following items when submitting your revised manuscript:

- A rebuttal letter that responds to each point raised by the academic editor and reviewer(s). You should upload this letter as a separate file labeled 'Response to Reviewers'.
- A marked-up copy of your manuscript that highlights changes made to the original version. You should upload this as a separate file labeled 'Revised Manuscript with Track Changes'.
- An unmarked version of your revised paper without tracked changes. You should upload this as a separate file labeled 'Manuscript'.

If you would like to make changes to your financial disclosure, please include your updated statement in your cover letter. Guidelines for resubmitting your figure files are available below the reviewer comments at the end of this letter.

We look forward to receiving your revised manuscript.

Kind regards,

A. K. M. Anisur Rahman, Ph.D.

Academic Editor

PLOS ONE

Journal Requirements:

When submitting your revision, we need you to address these additional requirements.

1. Please ensure that your manuscript meets PLOS ONE's style requirements, including those for file naming. The PLOS ONE style templates can be found at

https://journals.plos.org/plosone/s/file?id=wjVg/PLOSOne_formatting_sample_main_body.pdf and

https://journals.plos.org/plosone/s/file?id=ba62/PLOSOne_formatting_sample_title_authors_affiliations.pdf

2.  We note that you have stated that you will provide repository information for your data at acceptance. Should your manuscript be accepted for publication, we will hold it until you provide the relevant accession numbers or DOIs necessary to access your data. If you wish to make changes to your Data Availability statement, please describe these changes in your cover letter and we will update your Data Availability statement to reflect the information you provide.

[Note: HTML markup is below. Please do not edit.]

Reviewers' comments:

Reviewer's Responses to Questions

**Comments to the Author**

1. Is the manuscript technically sound, and do the data support the conclusions?

The manuscript must describe a technically sound piece of scientific research with data that supports the conclusions. Experiments must have been conducted rigorously, with appropriate controls, replication, and sample sizes. The conclusions must be drawn appropriately based on the data presented.

Reviewer #1: Partly

Reviewer #2: No

**********

2. Has the statistical analysis been performed appropriately and rigorously?

Reviewer #1: Yes

Reviewer #2: No

**********

3. Have the authors made all data underlying the findings in their manuscript fully available?

The PLOS Data policy requires authors to make all data underlying the findings described in their manuscript fully available without restriction, with rare exception (please refer to the Data Availability Statement in the manuscript PDF file). The data should be provided as part of the manuscript or its supporting information, or deposited to a public repository. For example, in addition to summary statistics, the data points behind means, medians and variance measures should be available. If there are restrictions on publicly sharing data—e.g. participant privacy or use of data from a third party—those must be specified.

Reviewer #1: Yes

Reviewer #2: No

**********

4. Is the manuscript presented in an intelligible fashion and written in standard English?

PLOS ONE does not copyedit accepted manuscripts, so the language in submitted articles must be clear, correct, and unambiguous. Any typographical or grammatical errors should be corrected at revision, so please note any specific errors here.

Reviewer #1: No

Reviewer #2: Yes

**********

5. Review Comments to the Author

Please use the space provided to explain your answers to the questions above. You may also include additional comments for the author, including concerns about dual publication, research ethics, or publication ethics. (Please upload your review as an attachment if it exceeds 20,000 characters)

Reviewer #1: The manuscript by Oguttu et al on "Knowledge, attitude and practices toward rabies" bears reference. This is an important for investigations as it identifies gaps which can be used for intervention to reduce the number of dog-mediated human rabies deaths. The investigation in question was undertaken in a perfect setting considering the number of dog rabies outbreaks and human deaths confirmed from this southern African country. I have included specific issues that the authors need to address.

Minor:

1. Throughout the document, the authors need to be congisant and specify rabies as a disease and rabies virus as the aetiologic agent of the disease.

ii. The third line in the introduction should be amended to read "All warm-blooded mammals are susceptible to rabies virus infection generally transmitted through the bite of an infected rabid animal",

iii. Also amend the sentence "Rabies infection is attributable to domestic dogs and remains .........perspective",.

iv. The estimated number of 60 000 human deaths was cited by Hampson et al., 2015 and not Morters et al., 2015.

v. Combine the two snetences "Almost all (98%) ............and In Africa" as both state the same issue.

vi. The sentence that starts "Rabies can only be prevented but not cured" - there are a few cases of human who were cured - please refer to the Willoughby and Milwakee protocol for this.

vii. On page 3, a combination of rabies immunoglobulin and vaccine is given dpending on the vategory of the bite - please qualify this.

viii. Please provide a reference for the sentence that talks about rabies having the highest case fatality rate of any disease,

ix. Replace "reduce rabies disease" with "control the disease at its source",. second para page 3. Secondly, replace "outbreaks occur" with "rabies is endemic".

x. What is a "high burden of rabies mortality persists",

xi. Rewrite the sentence to read "In South Africa, an estimated 40 human rabies rleated deaths from dog bite contacts occur yearly", In the same para, include Limpopo and the Eastern Cape provinces in addition to KZN. You should also include records and illustrations from the Department of Agricuture Land Reform and Rural Development to depict the current rabies situation in the country.

xii. In the last para, delete disease so that it reads "rabies" only,

xiii. First sentence on page 4 - specify the selected rural and urban areas.

xiv. Please provide a map of the country and the study areas to guide the reader of the geographical location of this sarea.

xv. Second para, replace "populated by different races" by "a mulit-racial community", and delete ""for different races" at the end of the sentence.

xvi. Third para, rewrite to read "Verulam has an estimated 64,950 households".

xvii. Under data colelction - insert "a" after "using",

xix. Under section 4 - replace reported with disseminated.

xx. Under 5.1.1 - replace among with in.

xxi. In Table 1, I suggest replace matric with "high school education",

xxii. Under Table 2, replace "being responsible to" with "the likely risk for",

xxiii. On page 11 - rewrite to read "The inability of a dog to drink and eat as signs of rabies were mentioned".

xxiv. Rewrite in para 5 - "indicated that their pets were not sterilised", on page 14 - was there a follow up on why >50% of the respondents were not in favour of sterilisation?

xxv. On page 13, does ongoing campaigns refer to annual vaccination campaigns?

xxvi. On page 15, replace referent with reference,

xxvii. Seconf sentence of the discussion - delate the "causes" at the end of the snetence.,

xxviii. Under 6.1 - rwrite to read ".... when it comes to pet management in the homes",.

xxix. First para on page 17 - rewrite to read "Findings from the present study also contrast those from a study conducted in Cameroon in which less than half of the reposndents owned pets",.

xxx. second para - "In this study, just below half (47.66%) of respondents were Christians, contrary to fings in studies conducted in ...........distrcits which reported Muslim populations of 56.8% and 94.8% respectively".,

xxxi. Under 6.2 - how are the results bettter?

xxxii. Under references - is Abedela Nehash (2017) and Abdela et al., 2017 - the same reference? Please check whether the following are included in the text (I may have missed them) - Lankester et al., 2016; Sabeta et al., 2013; Report CF 2010; Tiwari, Vanak et al., 2019a; Ubeyratne et al., 2020;

Major:

i. Sample size determination - Was the 50% prevalence based on previous research studies e.g. Hergert and Nel?

ii. On page 9, it is indicated that playing with the dog could lead to contraction of of rabies - during the discussions was there any implicationsthat other host species could be included, e.g. cats, willdife?

iii. Third para - please rewrite "... that vaccination of pets is important to prevent humans contracting the disease".

iv. One major weakness in the mnauscript and in the discussion is the repetition of results,

v. On page 19, 3rd para - discuss the differences in the results?

vi. Under 6.3 - is the endemicity of rabies in KZN and India and Ethiopia comparable - give the reader soemthong to think about.

vii. In the discussion, there are very few references being disucssed in the context of the results obtained here - Gebremeskei et al., 2019; Bihon et al., 2020. The authors should expand and compare the findings to other regions.

viii. The authors on page 24 indicated that adhering to repeated and regular vaccinations helps boost the immune level of pets - what important question is what the turnover rate of the dog popualtion we are dealing with here?

ix. On page 25 - the authors allude to rabies eradiation campaigns? Is this achievable - I suggest that the authors use elimination of dog-mediated human rabies as other wildife vectors bats, jackals have a potential to spill over into the dog population.

x. Under conlcusions, the authors highlighted the identified gaps. But what is important is an intervention. How will these gaps fit into the national rabies control programme of South Africa as we move towards 2030? There is currently a one health group - how will these gaps help strengthen the current collaboration in that province?

xi. Coertse et al., 2017 - refers to rabies-related Mokola and not to dog rabies. Please include an appropriate reference for dog rabies in South Africa and there are many such references.

Reviewer #2: The authors conducted a descriptive analysis of rabies Knowledge, Attitudes, and Practices (KAP) in KwaZulu-Natal Province, South Africa, involving 768 participants. Based on the responses obtained, the authors concluded that a lack of knowledge regarding the cause of rabies and the importance of receiving Post-Exposure Prophylaxis (PEP) immediately after a dog bite was prevalent in the study area. However, they also identified certain gaps in awareness that require targeted interventions for the general population, including those who do not own pets.

While studies like this are informative and valuable for understanding the current state of knowledge and practices, there is room for advancement to make the research more substantial. The approach taken in this study, in its current form, may be considered too simplistic for publication in a high-impact journal, as it does not significantly contribute to advancing the field of research. Simply summarizing data is considered a basic step, and the authors are encouraged to perform more advanced analyses to provide deeper insights and contribute to the existing body of knowledge on this topic.

The authors are urged to consider conducting more in-depth research, incorporating sophisticated methodologies or analyses that can offer novel perspectives and meaningful contributions to the field of rabies knowledge and prevention. See a few papers as a guide https://www.ncbi.nlm.nih.gov/pmc/articles/PMC9649959/,https://bmcpublichealth.biomedcentral.com/articles/10.1186/s12889-020-8145-7, https://www.sciencedirect.com/science/article/pii/S2352771421000549

**********

6. PLOS authors have the option to publish the peer review history of their article (what does this mean?). If published, this will include your full peer review and any attached files.

If you choose “no”, your identity will remain anonymous but your review may still be made public.

**Do you want your identity to be public for this peer review?**  For information about this choice, including consent withdrawal, please see our Privacy Policy.

Reviewer #1: No

Reviewer #2: No

**********

[NOTE: If reviewer comments were submitted as an attachment file, they will be attached to this email and accessible via the submission site. Please log into your account, locate the manuscript record, and check for the action link "View Attachments". If this link does not appear, there are no attachment files.]

While revising your submission, please upload your figure files to the Preflight Analysis and Conversion Engine (PACE) digital diagnostic tool, https://pacev2.apexcovantage.com/. PACE helps ensure that figures meet PLOS requirements. To use PACE, you must first register as a user. Registration is free. Then, login and navigate to the UPLOAD tab, where you will find detailed instructions on how to use the tool. If you encounter any issues or have any questions when using PACE, please email PLOS at figures@plos.org. Please note that Supporting Information files do not need this step.

# Author comment: Author response to Decision Letter 1

**DOI:** 10.1371/journal.pone.0352279.r002

21 Apr 2024

Minor:

1. Throughout the document, the authors need to be congisant and specify rabies as a disease and rabies virus as the aetiologic agent of the disease.

Response: The authors have specified rabies as either virus or disease as suggested by the reviewer(s)

ii. The third line in the introduction should be amended to read "All warm-blooded mammals are susceptible to rabies virus infection generally transmitted through the bite of an infected rabid animal",

Response: As suggested by the reviewer (s), the authors have amended the sentence to the above mentioned.

iii. Also amend the sentence "Rabies infection is attributable to domestic dogs and remains .........perspective",.

Response: The sentence was amended according to the reviewer’s suggestion to: Dog mediated rabies infection remains a significant disease from a public health perspective.

iv. The estimated number of 60 000 human deaths was cited by Hampson et al., 2015 and not Morters et al., 2015.

Response: Morters et al., 2015 ‘Effective vaccination against rabies in puppies in rabies endemic regions’ has estimated that 60 000 human deaths occur yearly, while Hampson et al., ‘Estimating the Global Burden of Endemic Canine Rabies’ estimated mortalities in human annually to be around 59 000.

v. Combine the two sentences "Almost all (98%) ............and In Africa" as both state the same issue.

Response: The sentences have been combined to; In Africa, over 90% of human rabies deaths are associated with dog-bite.

vi. The sentence that starts "Rabies can only be prevented but not cured" - there are a few cases of human who were cured - please refer to the Willoughby and Milwakee protocol for this.

Response: Thank you for referring us to Willoughby and Milwaukee protocol. The authors have rephrased the statement.

vii. On page 3, a combination of rabies immunoglobulin and vaccine is given depending on the vategory of the bite - please qualify this.

Response: For example, a dog bite victim determined to be a category 3, is a patient who has been bitten by an animal suspected of having rabies and the victim presents with open wounds and bleeding on the bite site.

viii. Please provide a reference for the sentence that talks about rabies having the highest case fatality rate of any disease,

Response: The authors cited Rine et al., 2017, who reported that rabies has highest case fatality rate of any disease

ix. Replace "reduce rabies disease" with "control the disease at its source",. second para page

Response: Accordingly, the authors replaced “reduce rabies disease” with “control at its source”

3. Secondly, replace "outbreaks occur" with "rabies is endemic".

Response: As suggested by the reviewers, the authors replaced “outbreak occur” with “rabies is endemic”.

x. What is a "high burden of rabies mortality persists",

Response: "high burden of rabies mortality persists” means that high number of people dying due to rabies remain unchecked. However, the sentence was rephrased to read as follows: “However, in most developing countries, rabies continues to be a challenge resulting in high mortalities in humans and animals”.

xi. Rewrite the sentence to read "In South Africa, an estimated 40 human rabies rleated deaths from dog bite contacts occur yearly", In the same para, include Limpopo and the Eastern Cape provinces in addition to KZN. You should also include records and illustrations from the Department of Agriculture Land Reform and Rural Development to depict the current rabies situation in the country.

Response: The authors have highlighted Limpopo and Eastern cape as other provinces that experience rabies as reported by Department of Agriculture, Land Reform and Rural Development.

xii. In the last para, delete disease so that it reads "rabies" only,

Response: Disease was deleted

xiii. First sentence on page 4 - specify the selected rural and urban areas.

Response: The areas have been specified as rural (Embo) and urban (Verulam).

xiv. Please provide a map of the country and the study areas to guide the reader of the geographical location of this area.

Response: We failed to secure a good clear map that shows the two areas. The few maps we managed to obtain, although not good, also require applying for copyright which we have failed to secure. We have thus opted to provide the coordinates for the two areas.

Embo (Latitude: 29° 54' 34" S, Longitude: 30° 40' 29" E)

Verulam (Latitude: -29° 38' 59.99" S; Longitude: 31° 02' 60.00" E

xv. Second para, replace "populated by different races" by "a mulit-racial community", and delete ""for different races" at the end of the sentence.

Response: The words in the sentence have been revised according to what the reviewer(s) suggested.

xvi. Third para, rewrite to read "Verulam has an estimated 64,950 households".

Response: The sentence has been rephrased to what the reviewer has suggested

xvii. Under data collection - insert "a" after "using",

Response: “a” was inserted

xix. Under section 4 - replace reported with disseminated.

Response: The authors replaced “reported” with “disseminated”

xx. Under 5.1.1 - replace among with in.

Response: Among was replaced with “in”

xxi. In Table 1, I suggest replace matric with "high school education",

Response: Thanks for the suggestion. We have amended accordingly.

xxii. Under Table 2, replace "being responsible to" with "the likely risk for",

Response: The sentence was revised by addition of “the likely risk for”

xxiii. On page 11 - rewrite to read "The inability of a dog to drink and eat as signs of rabies were mentioned".

Response: The authors have rephrased the sentence as suggested

xxiv. Rewrite in para 5 - "indicated that their pets were not sterilised", on page 14 - was there a follow up on why >50% of the respondents were not in favour of sterilisation?

Response: Cross-sectional study design was employed in this study, which was only conducted once. We agree it would have been good to follow up. We will take this into consideration in future or follow up studies.

xxv. On page 13, does ongoing campaigns refer to annual vaccination campaigns?

Response: The authors have rephrased the sentence to specify that this refers to annual vaccination campaigns

xxvi. On page 15, replace referent with reference,

Response: Referent was replaced with reference

xxvii. Second sentence of the discussion - delate the "causes" at the end of the snetence.,

Response: Causes was removed from the sentence

xxviii. Under 6.1 - rewrite to read ".... when it comes to pet management in the homes",.

Response: The authors amended as recommended and added: “when it comes to pet management in the homes” to the sentence

xxix. First para on page 17 - rewrite to read "Findings from the present study also contrast those from a study conducted in Cameroon in which less than half of the respondents owned pets",.

Response: The authors have rephrased the sentence as advised to read as: Findings from the present study also contrast those from a study conducted in Cameroon in which less than half of the respondents owned pets.

xxx. second para - "In this study, just below half (47.66%) of respondents were Christians, contrary to findings in studies conducted in ...........distrcits which reported Muslim populations of 56.8% and 94.8% respectively".,

Response: The authors have rephrased the sentence to the one suggested by the reviewers

xxxi. Under 6.2 - how are the results better?

Response: Thanks for pointing this out, we have rephrsed the sentence to read as follows: “However, in a study conducted in Kombolcha, Ethiopia, a higher number (76.6%; n=294) of respondents were aware.

xxxii. Under references - is Abedela Nehash (2017) and Abdela et al., 2017 - the same reference? Please check whether the following are included in the text (I may have missed them) - Lankester et al., 2016; Sabeta et al., 2013; Report CF 2010; Tiwari, Vanak et al., 2019a; Ubeyratne et al., 2020;

Response: The authors have checked and ensured that all cited references are listed in the bibliography

Major:

i. Sample size determination - Was the 50% prevalence based on previous research studies e.g. Hergert and Nel?

Response: The authors have not found studies that looked at the “prevalence” of knowledge that were previously done in the selected areas of study and therefore assumed prevalence of knowledge to be 50%. Available literature refers to the prevalence of rabies which was not the focus of this study.

ii. On page 9, it is indicated that playing with the dog could lead to contraction of rabies - during the discussions was there any implications that other host species could be included, e.g. cats, willdife?

Response: We thank the reviewer for the comment. Unfortunately, it is not clear to us what the reviewer wanted us to do, or which change to make.

iii. Third para - please rewrite "... that vaccination of pets is important to prevent humans contracting the disease".

Response: The sentence was rephrased to the suggested

iv. One major weakness in the manuscript and in the discussion is the repetition of results,

Response: The authors have reviewed the discussion to avoid repetition of results.

v. On page 19, 3rd para - discuss the differences in the results?

Response: The authors have discussed the differences in results

vi. Under 6.3 - is the endemicity of rabies in KZN and India and Ethiopia comparable - give the reader something to think about.

Response: We appreciate this comment-we have added something on India and Ethiopia to the effect that like SA it is a rabies endemic area.

vii. In the discussion, there are very few references being disucssed in the context of the results obtained here - Gebremeskei et al., 2019; Bihon et al., 2020. The authors should expand and compare the findings to other regions.

Response: we take note of the reviewer’s concern. We would like to reiterate that we have endeavoured to discussion our findings in the context of what is known and also to explain our findings. We refer to studies from Rwanda, Morocco, india, Ethiopia, Filipions etc. It is possible, we missed what the reviewer intended us to do. We will gladly attend to it if further clarification is provided.

viii. The authors on page 24 indicated that adhering to repeated and regular vaccinations helps boost the immune level of pets - what important question is what the turnover rate of the dog population we are dealing with here?

Response: The question was intended to reveal whether the respondent would be keen to another vaccination when the campaigns are conducted. That notwithstanding we agree with the reviewer that it would be good to consider the turnover rate of the dog population in the study area to establish the “herd immunity”. However, this was outside the scope of this work.

ix. On page 25 - the authors allude to rabies eradiation campaigns? Is this achievable - I suggest that the authors use elimination of dog-mediated human rabies as other wildife vectors bats, jackals have a potential to spill over into the dog population.

Response: The authors appreciate the suggestion and therefore made the necessary changes.

x. Under conclusions, the authors highlighted the identified gaps. But what is important is an intervention. How will these gaps fit into the national rabies control programme of South Africa as we move towards 2030? There is currently a one health group - how will these gaps help strengthen the current collaboration in that province?

Response: The 2030 agenda aims to eliminate rabies, through mass dog vaccination campaigns that achieve 70%vaccination rate per annum (Mbilo et al., 2019). The authors recommended that to bridge the gaps, implementation of global strategies such as awareness and vaccination campaigns to address such should be effective in order to eradicate rabies in South Africa.

xi. Coertse et al., 2017 - refers to rabies-related Mokola and not to dog rabies. Please include an appropriate reference for dog rabies in South Africa and there are many such references.

Response: corrected. Reference has been added.

Reviewer #2: The authors conducted a descriptive analysis of rabies Knowledge, Attitudes, and Practices (KAP) in KwaZulu-Natal Province, South Africa, involving 768 participants. Based on the responses obtained, the authors concluded that a lack of knowledge regarding the cause of rabies and the importance of receiving Post-Exposure Prophylaxis (PEP) immediately after a dog bite was prevalent in the study area. However, they also identified certain gaps in awareness that require targeted interventions for the general population, including those who do not own pets.

Response: We are appreciative of the positive comment on our work by the reviewer. However, we would we would like to point out that our work also had an analytical component; hence we were able to make inferences.

While studies like this are informative and valuable for understanding the current state of knowledge and practices, there is room for advancement to make the research more substantial. The approach taken in this study, in its current form, may be considered too simplistic for publication in a high-impact journal, as it does not significantly contribute to advancing the field of research. Simply summarizing data is considered a basic step, and the authors are encouraged to perform more advanced analyses to provide deeper insights and contribute to the existing body of knowledge on this topic.

Response: The authors have analysed data using descriptive statistics and logistic regression model (univariate and multivariate analysis) to determine significant predictors of knowledge. We would appreciate if the reviewer was kind as to be specific on the type of advanced analysis he or she would have loved to see performed. But that aside, we believe that we were able to achieve the objectives for which we set out to do in this work. Last but least, we believe that this study contributes to improved understanding of the disease in the study area that happens to be endemic to rabies.

The authors are urged to consider conducting more in-depth research, incorporating sophisticated methodologies or analyses that can offer novel perspectives and meaningful contributions to the field of rabies knowledge and prevention.

Response: The approach used in this study is widely used in KAP studies. Therefore, it was adapted, and we feel it is suited to this study. However, we are keen to learn more about what we could have done, if the reviewer was kind as to advise on the kind of analysis, he or she would like to see us perform to enrich this study.

Attachment Submitted filename: Response to Reviewers.docx

# Peer review: Decision Letter 1

**DOI:** 10.1371/journal.pone.0352279.r003

**Authors:** A. K. M. Anisur Rahman

28 May 2024

PONE-D-23-27745R1

Knowledge, attitude and practices towards rabies: A community survey in selected areas of KwaZulu-Natal Province, South Africa

PLOS ONE

Dear Dr. Oguttu,

Thank you for submitting your manuscript to PLOS ONE. After careful consideration, we feel that it has merit but does not fully meet PLOS ONE’s publication criteria as it currently stands. Therefore, we invite you to submit a revised version of the manuscript that addresses the points raised during the review process.

==============================

**ACADEMIC EDITOR:**

In addition:

Please delete "prospective" from line 18, "of the respondents." from line 23 and "treatment" from line 25.

PEP includes wound washing, a dose of human rabies immune globulin (HRIG), and rabies vaccine given at the time of the first medical visit, and two booster doses. Please correct the statement at line 25.

Please delete "rate" from line 56 as it is a proportion.

Please replace all "Univariate" and "multivariate" with "univariable" and "multivariable" throughout the manuscript.

Line 160: Please delete "binary".

Please mention the software used for the logistic regression analysis. Lines 318-322: Univariable results are not final, so please don't describe them.

-->.

==============================

Please submit your revised manuscript by Jul 12 2024 11:59PM. If you will need more time than this to complete your revisions, please reply to this message or contact the journal office at plosone@plos.org. When you're ready to submit your revision, log on to https://www.editorialmanager.com/pone/ and select the 'Submissions Needing Revision' folder to locate your manuscript file.

Please include the following items when submitting your revised manuscript:

- A rebuttal letter that responds to each point raised by the academic editor and reviewer(s). You should upload this letter as a separate file labeled 'Response to Reviewers'.
- A marked-up copy of your manuscript that highlights changes made to the original version. You should upload this as a separate file labeled 'Revised Manuscript with Track Changes'.
- An unmarked version of your revised paper without tracked changes. You should upload this as a separate file labeled 'Manuscript'.

We look forward to receiving your revised manuscript.

Kind regards,

A. K. M. Anisur Rahman, Ph.D.

Academic Editor

PLOS ONE

Journal Requirements:

Please review your reference list to ensure that it is complete and correct. If you have cited papers that have been retracted, please include the rationale for doing so in the manuscript text, or remove these references and replace them with relevant current references. Any changes to the reference list should be mentioned in the rebuttal letter that accompanies your revised manuscript. If you need to cite a retracted article, indicate the article’s retracted status in the References list and also include a citation and full reference for the retraction notice.

[Note: HTML markup is below. Please do not edit.]

Reviewers' comments:

Reviewer's Responses to Questions

**Comments to the Author**

1. If the authors have adequately addressed your comments raised in a previous round of review and you feel that this manuscript is now acceptable for publication, you may indicate that here to bypass the “Comments to the Author” section, enter your conflict of interest statement in the “Confidential to Editor” section, and submit your "Accept" recommendation.

Reviewer #1: (No Response)

Reviewer #2: (No Response)

**********

2. Is the manuscript technically sound, and do the data support the conclusions?

The manuscript must describe a technically sound piece of scientific research with data that supports the conclusions. Experiments must have been conducted rigorously, with appropriate controls, replication, and sample sizes. The conclusions must be drawn appropriately based on the data presented.

Reviewer #1: Yes

Reviewer #2: Yes

**********

3. Has the statistical analysis been performed appropriately and rigorously?

Reviewer #1: Yes

Reviewer #2: N/A

**********

4. Have the authors made all data underlying the findings in their manuscript fully available?

The PLOS Data policy requires authors to make all data underlying the findings described in their manuscript fully available without restriction, with rare exception (please refer to the Data Availability Statement in the manuscript PDF file). The data should be provided as part of the manuscript or its supporting information, or deposited to a public repository. For example, in addition to summary statistics, the data points behind means, medians and variance measures should be available. If there are restrictions on publicly sharing data—e.g. participant privacy or use of data from a third party—those must be specified.

Reviewer #1: Yes

Reviewer #2: Yes

**********

5. Is the manuscript presented in an intelligible fashion and written in standard English?

PLOS ONE does not copyedit accepted manuscripts, so the language in submitted articles must be clear, correct, and unambiguous. Any typographical or grammatical errors should be corrected at revision, so please note any specific errors here.

Reviewer #1: Yes

Reviewer #2: Yes

**********

6. Review Comments to the Author

Please use the space provided to explain your answers to the questions above. You may also include additional comments for the author, including concerns about dual publication, research ethics, or publication ethics. (Please upload your review as an attachment if it exceeds 20,000 characters)

Reviewer #1: Minor issues picked up:

Abstract:

Line 8 - delete "of the respondents"

Introduction":

Line 3: replace "the" with "a",

Insert "human or equine" before "rabies immunoglobulin,

Page 3, para 1 Replace "having high numbers of" with "endemic for dog",

Page 8, after the table, "rout" should read "route",

Under 4.1.3 - insert "vaccine" after anti-rabies, second para - animal should read "animals",

Page 12, Write SPCA in full,

Table 4: 5th question should read "Was your pet sterilised", question 7 - replace with "How often has your dog been vaccinated", 12th question - animals should read animal,

Page 16, rabies disease should read rabies,

Page 17, 2nd line - later should read latter,

section 5.2 - 2nd para, line 9 - shows should read show,

Page 18, line 1 - replace biting with bite contact, 2nd para, line 4 - replace infection with virus, replace "to the victim" with "into the peripheral sites", 2nd para, line 7 - implement should read "implemented", line 10 - insert "virus infection" after rabies,

Page 20, line 14 - replace "from" with "at",

Page 23, line 8 - delete "when they want to reproduce",

Page 24, para 2 - replace "thus minimise outbreaks of rabies" with " break dog rabies cycles",

Reviewer #2: I thank the authors for improving the manuscript. While the authors built a multivariable regression model, their outcome was not clear enough to allow for replication of the study. I therefore invite the authors to clarify how they obtained their outcome.

**********

7. PLOS authors have the option to publish the peer review history of their article (what does this mean?). If published, this will include your full peer review and any attached files.

If you choose “no”, your identity will remain anonymous but your review may still be made public.

**Do you want your identity to be public for this peer review?**  For information about this choice, including consent withdrawal, please see our Privacy Policy.

Reviewer #1: No

Reviewer #2: No

**********

[NOTE: If reviewer comments were submitted as an attachment file, they will be attached to this email and accessible via the submission site. Please log into your account, locate the manuscript record, and check for the action link "View Attachments". If this link does not appear, there are no attachment files.]

While revising your submission, please upload your figure files to the Preflight Analysis and Conversion Engine (PACE) digital diagnostic tool, https://pacev2.apexcovantage.com/. PACE helps ensure that figures meet PLOS requirements. To use PACE, you must first register as a user. Registration is free. Then, login and navigate to the UPLOAD tab, where you will find detailed instructions on how to use the tool. If you encounter any issues or have any questions when using PACE, please email PLOS at figures@plos.org. Please note that Supporting Information files do not need this step.

# Author comment: Author response to Decision Letter 2

**DOI:** 10.1371/journal.pone.0352279.r004

29 May 2024

Response to the Academic Editor

Please delete "prospective" from line 18, "of the respondents." from line 23 and "treatment" from line 25.

Response: corrected as advised

PEP includes wound washing, a dose of human rabies immune globulin (HRIG), and rabies vaccine given at the time of the first medical visit, and two booster doses. Please correct the statement at line 25.

Response: Corrected as advised-see lines 25-27

Please delete "rate" from line 56 as it is a proportion.

Response: “rate” deleted from line 58

Please replace all "Univariate" and "multivariate" with "univariable" and "multivariable" throughout the manuscript.

Response: corrected as advised.

Line 160: Please delete "binary".

Response: Corrected as advised-see line 162

Please mention the software used for the logistic regression analysis. Lines 318-322: Univariable results are not final, so please don't describe them.

Response: The following statement has been added under data analysis (lines 156-157)

Data was analysed using the Statistical Package for Social Science (SPSS) Version 27 (SPSS for Windows, version 27, SPSS, Inc, Chicago, Illinois, the USA).

Results of univariable regression have been removed as advised.

Attachment Submitted filename: Response to Reviewers.pdf

# Editor report: Decision Letter 2

**DOI:** 10.1371/journal.pone.0352279.r005

**Authors:** A. K. M. Anisur Rahman

3 Jun 2024

PONE-D-23-27745R2

Knowledge, attitude and practices towards rabies: A community survey in selected areas of KwaZulu-Natal Province, South Africa

PLOS ONE

Dear Dr. Oguttu,

Thank you for submitting your manuscript to PLOS ONE. After careful consideration, we feel that it has merit but does not fully meet PLOS ONE’s publication criteria as it currently stands. Therefore, we invite you to submit a revised version of the manuscript that addresses the points raised during the review process.

==============================

**ACADEMIC EDITOR:**

Line 163-164: Significance in the multivariable model was assessed at p ˃ 0.05.  Please correct this.

==============================

Please submit your revised manuscript by Jul 18 2024 11:59PM. If you will need more time than this to complete your revisions, please reply to this message or contact the journal office at plosone@plos.org. When you're ready to submit your revision, log on to https://www.editorialmanager.com/pone/ and select the 'Submissions Needing Revision' folder to locate your manuscript file.

Please include the following items when submitting your revised manuscript:

- A rebuttal letter that responds to each point raised by the academic editor and reviewer(s). You should upload this letter as a separate file labeled 'Response to Reviewers'.
- A marked-up copy of your manuscript that highlights changes made to the original version. You should upload this as a separate file labeled 'Revised Manuscript with Track Changes'.
- An unmarked version of your revised paper without tracked changes. You should upload this as a separate file labeled 'Manuscript'.

-->We look forward to receiving your revised manuscript.

Kind regards,

A. K. M. Anisur Rahman, Ph.D.

Academic Editor

PLOS ONE

Journal Requirements:

Please review your reference list to ensure that it is complete and correct. If you have cited papers that have been retracted, please include the rationale for doing so in the manuscript text, or remove these references and replace them with relevant current references. Any changes to the reference list should be mentioned in the rebuttal letter that accompanies your revised manuscript. If you need to cite a retracted article, indicate the article’s retracted status in the References list and also include a citation and full reference for the retraction notice.

[Note: HTML markup is below. Please do not edit.]

[NOTE: If reviewer comments were submitted as an attachment file, they will be attached to this email and accessible via the submission site. Please log into your account, locate the manuscript record, and check for the action link "View Attachments". If this link does not appear, there are no attachment files.]

While revising your submission, please upload your figure files to the Preflight Analysis and Conversion Engine (PACE) digital diagnostic tool, https://pacev2.apexcovantage.com/. PACE helps ensure that figures meet PLOS requirements. To use PACE, you must first register as a user. Registration is free. Then, login and navigate to the UPLOAD tab, where you will find detailed instructions on how to use the tool. If you encounter any issues or have any questions when using PACE, please email PLOS at figures@plos.org. Please note that Supporting Information files do not need this step.

# Author comment: Author response to Decision Letter 3

**DOI:** 10.1371/journal.pone.0352279.r006

11 Jul 2024

We have revised the manuscript and provided a point by point explanation of how we responded to the Academic Editor's comment and suggestions. We have also formated the paper according to the templated for PLOSONE. If the paper is accepted for publication we will make the data available on the institutional repository.

Attachment Submitted filename: Response_to_Reviewers_auresp_3.pdf

# Peer review: Decision Letter 3

**DOI:** 10.1371/journal.pone.0352279.r007

**Authors:** Aravindh Babu Ramasamy Parthiban

22 Oct 2024

PONE-D-23-27745R3

Knowledge, attitude and practices towards rabies: A community survey in selected areas of KwaZulu-Natal Province, South Africa

PLOS ONE

Dear Dr. Oguttu,

Thank you for submitting your manuscript to PLOS ONE. After careful consideration, we feel that it has merit but does not fully meet PLOS ONE’s publication criteria as it currently stands. Therefore, we invite you to submit a revised version of the manuscript that addresses the points raised during the review process.

Please submit your revised manuscript by Dec 06 2024 11:59PM. If you will need more time than this to complete your revisions, please reply to this message or contact the journal office at plosone@plos.org. When you're ready to submit your revision, log on to https://www.editorialmanager.com/pone/ and select the 'Submissions Needing Revision' folder to locate your manuscript file.

Please include the following items when submitting your revised manuscript:

- A rebuttal letter that responds to each point raised by the academic editor and reviewer(s). You should upload this letter as a separate file labeled 'Response to Reviewers'.
- A marked-up copy of your manuscript that highlights changes made to the original version. You should upload this as a separate file labeled 'Revised Manuscript with Track Changes'.
- An unmarked version of your revised paper without tracked changes. You should upload this as a separate file labeled 'Manuscript'.

If you would like to make changes to your financial disclosure, please include your updated statement in your cover letter. Guidelines for resubmitting your figure files are available below the reviewer comments at the end of this letter.

If applicable, we recommend that you deposit your laboratory protocols in protocols.io to enhance the reproducibility of your results. Protocols.io assigns your protocol its own identifier (DOI) so that it can be cited independently in the future. For instructions see: https://journals.plos.org/plosone/s/submission-guidelines#loc-laboratory-protocols. Additionally, PLOS ONE offers an option for publishing peer-reviewed Lab Protocol articles, which describe protocols hosted on protocols.io. Read more information on sharing protocols at https://plos.org/protocols?utm_medium=editorial-email&utm_source=authorletters&utm_campaign=protocols.

We look forward to receiving your revised manuscript.

Kind regards,

Aravindh Babu Ramasamy Parthiban, B.V.Sc, M.V.Sc, Ph.D.

Academic Editor

PLOS ONE

Additional Editor Comments:

Comments from academic editor: While the study design and data is acceptable with major revisions (reviewers comments attached), such isolated studies have limited applicability/ usefulness in the field. The authors need to justify the same.

Reviewers' comments:

Reviewer's Responses to Questions

**Comments to the Author**

1. If the authors have adequately addressed your comments raised in a previous round of review and you feel that this manuscript is now acceptable for publication, you may indicate that here to bypass the “Comments to the Author” section, enter your conflict of interest statement in the “Confidential to Editor” section, and submit your "Accept" recommendation.

Reviewer #3: All comments have been addressed

Reviewer #4: (No Response)

Reviewer #5: (No Response)

**********

2. Is the manuscript technically sound, and do the data support the conclusions?

The manuscript must describe a technically sound piece of scientific research with data that supports the conclusions. Experiments must have been conducted rigorously, with appropriate controls, replication, and sample sizes. The conclusions must be drawn appropriately based on the data presented.

Reviewer #3: Yes

Reviewer #4: Partly

Reviewer #5: Yes

**********

3. Has the statistical analysis been performed appropriately and rigorously?

Reviewer #3: Yes

Reviewer #4: Yes

Reviewer #5: Yes

**********

4. Have the authors made all data underlying the findings in their manuscript fully available?

The PLOS Data policy requires authors to make all data underlying the findings described in their manuscript fully available without restriction, with rare exception (please refer to the Data Availability Statement in the manuscript PDF file). The data should be provided as part of the manuscript or its supporting information, or deposited to a public repository. For example, in addition to summary statistics, the data points behind means, medians and variance measures should be available. If there are restrictions on publicly sharing data—e.g. participant privacy or use of data from a third party—those must be specified.

Reviewer #3: Yes

Reviewer #4: Yes

Reviewer #5: Yes

**********

5. Is the manuscript presented in an intelligible fashion and written in standard English?

PLOS ONE does not copyedit accepted manuscripts, so the language in submitted articles must be clear, correct, and unambiguous. Any typographical or grammatical errors should be corrected at revision, so please note any specific errors here.

Reviewer #3: Yes

Reviewer #4: No

Reviewer #5: Yes

**********

6. Review Comments to the Author

Reviewer #3: The manuscript is well written and provide valuable information about the knowlege, attitude and practices about rabies in an African community. Only major suggestion to the the authors would be to concise the discussion session. Instead of providing the results for all the findings and discussing each of the results in the discussion session, it would be easier to discuss the most signifcant of the results and therefore avoid redundancy. Other minor comments are listed as comments in the attachment.

Reviewer #4: I was asked to review the manuscript along with the previous reviewers’ comments. Overall, I believe that the authors present a well conducted study which presents important information for policy makers in South Africa. Additionally, they highlight important knowledge gaps in individuals, and I believe this manuscript to include important information.

However, the manuscript is lengthy, and I recommend to the authors that they cut down a lot of what is presented in the results and discussion. Specifically, the results are presented both in Tables/Figures and they reiterated in the text. It is fairly redundant, and the authors need to have more brevity in the results section. The discussion section is a lot of reiteration of the results which should be removed. The discussion section is to interpret the results only. In my opinion, for this manuscript to be published, there needs to be substantial re-organization of the results and discussion sections

In addition, I have line-by-line comments to be addressed in an attached document.

Reviewer #5: General comments – I’m satisfied that this study was carried out carefully and that the data has been processed in a methodical way. I have listed various minor matters below, mostly typographical or grammatical errors or points that I felt needed clarifying. I would also particularly draw the authors’ attention to the issue with their referencing, discussed in more detail below.

Overall, my main reservation is that, while the discussion very competently considers how their data on specific points of analysis compares with previous studies carried out by various other researchers, I am left with no clear overall understanding of how knowledge of rabies in this study area compares with knowledge in other parts of South Africa, other countries in Africa, or elsewhere in the world. Does this study region perform well, overall, or poorly, or is it a very mixed pattern? (which is what I have gathered from reading the paper – but perhaps I’ve misunderstood). Are there any likely social causes that the authors think drive overall patterns of KAP towards rabies in their study area? They have discussed the reasons for individual findings, but not for the overall picture. Or, if they think the findings are internally contradictory in some ways, why might that be? Also, as a non-African reader, I only have a basic understanding of how dogs are kept in the study areas – I don’t know whether the two study areas would have obviously different ways of keeping dogs, for example, and the discussion doesn’t compare the two study areas with each other at all. I would have been glad to read an introductory sentence or two that described typical dog ownership patterns in the study regions, to orientate me.

In general, therefore, I think this paper is a solid piece of work which certainly deserves to be published. However, it would be helpful to have a more focused overview that discussed the findings as a whole to contextualise this study within the wider field of social science work on rabies KAP. The conclusion does a good job of summarising the findings for the South African context, I think, but perhaps slightly overlooks also presenting the findings in a way that is easily relevant for a global reader.

Detailed comments

NB – I have not called attention to all grammatical and spelling errors, just the ones that had most impact. There are others, so it would be useful for the authors to go through the manuscript themselves again carefully to find these. However, they did not detract from my understanding of the paper.

Abstract – gap before the full stop in line 26 – needs removing. The last sentence (lines 32-34) reads awkwardly, both because the language is a bit convoluted and because it doesn’t quite make sense to me, since it seems to be saying that some dog owners don’t own pets. This may be a cultural issue, because the article is discussing dog ownership in Africa, whereas I live in the UK, where dog ownership and pet ownership refer to the same thing in regard to dogs, more or less. Perhaps you mean that some people technically own dogs which actually lead a free-roaming lifestyle, rather than living in the home? To be more understandable to a global audience, perhaps reword to say something like ‘However, some participants revealed gaps in some aspects of rabies knowledge, suggesting a need for more public education for dog owners.’

Introduction – there is a problem with your referencing. To comply with PLOS ONE guidelines and general academic practice, your in-text citations should start with 1 and continue in numerical order throughout the manuscript. Instead, you have ordered the bibliography alphabetically, so that the numerical citations don’t start at 1 but are mixed up throughout the text. This is confusing for the reader and will be refused by PLOS ONE’s staff anyway, so needs correcting.

Line 39 – greatest compared to what? – I think you mean ‘greatest risk of any species’.

Lines 45-46 don’t read very smoothly – suggest removing ‘Of these’, which doesn’t help clarity, and also removing ‘Making the two the most affected continents’, which is not a full sentence and is not needed, since you have just told the reader the same thing in the previous sentence.

Line 47 – you give a very precise number and say ‘approximately’, which reads a bit strangely – would suggest rounding to 31,500 or even ‘over 30,000’.

Methods

Line 94 – I don’t understand ‘1, 2%’ – is this 1.2% or 1-2% or something else?

Line 152 – how did you convert a percentage knowledge score to a binary variable? What was the cutoff and how was it chosen? Surely this will drastically affect the results, but you don’t seem to mention it?

Line 162 – fitted

Line 196, table 1 – I don’t understand some of the education categories. Again, I think this is probably a cultural difference. Does ‘completed primary’ imply ‘and did not progress to secondary’? If so, it might be more clear to make the next category ‘Secondary started but not completed’, or to make the primary category ‘primary education only’, because to me, it reads as if ‘completed primary’ and ‘secondary not completed’ might overlap, since someone could have completed primary and moved to secondary but not finished it, and would then be in both groups. I hope this makes sense.

Results

Line 206 – typo – ‘route’.

Line 211 – two sentences need joining together here, as the 210 ‘Although’ sentence doesn’t make sense as a stand-alone sentence.

Discussion

Line 417 – typo – ‘where’.

477 – previously the discussion has broadly been suggesting that your study area has less knowledge about rabies than some other studied areas, but here you point out that your area is performing better on canine vaccination (and knowledge re human vaccination, around line 500) than some other regions. It would be interesting to consider holistically why your area is doing better in some ways and worse in others than other comparable regions. At the moment, although your discussion is detailed and painstaking, it is a bit fragmented, so that it’s hard for someone who doesn’t know the region to get an overview of how the study area compares to other endemic areas.

Line 588 – typo – ‘in possession’, not ‘in position’.

Line 670 – where was Mbilo’s study carried out?

Conclusion

Line 713 – explain what this video is? I can guess it’s a government education video, but it would be good to know!

**********

7. PLOS authors have the option to publish the peer review history of their article (what does this mean?). If published, this will include your full peer review and any attached files.

If you choose “no”, your identity will remain anonymous but your review may still be made public.

**Do you want your identity to be public for this peer review?**  For information about this choice, including consent withdrawal, please see our Privacy Policy.

Reviewer #3: No

Reviewer #4: No

Reviewer #5: No

**********

[NOTE: If reviewer comments were submitted as an attachment file, they will be attached to this email and accessible via the submission site. Please log into your account, locate the manuscript record, and check for the action link "View Attachments". If this link does not appear, there are no attachment files.]

While revising your submission, please upload your figure files to the Preflight Analysis and Conversion Engine (PACE) digital diagnostic tool, https://pacev2.apexcovantage.com/. PACE helps ensure that figures meet PLOS requirements. To use PACE, you must first register as a user. Registration is free. Then, login and navigate to the UPLOAD tab, where you will find detailed instructions on how to use the tool. If you encounter any issues or have any questions when using PACE, please email PLOS at figures@plos.org. Please note that Supporting Information files do not need this step.

Attachment Submitted filename: PONE-D-23-27745_R3_reviewerComments.pdf

Attachment Submitted filename: Review.docx

# Author comment: Author response to Decision Letter 4

**DOI:** 10.1371/journal.pone.0352279.r008

28 Nov 2024

Reviewer: Overall, I believe that the authors present a well conducted study which presents important information for policy makers in South Africa. Additionally, they highlight important knowledge gaps in individuals, and I believe this manuscript to include important information.

Response: We would like to thank the reviewer for the positive comment with respect to our work

Reviewer: However, the manuscript is lengthy, and I recommend to the authors that they cut down a lot of what is presented in the results and discussion. Specifically, the results are presented both in Tables/Figures and they reiterated in the text. It is redundant, and the authors need to have more brevity in the results section.

Response: We acknowledge the reviewer’s concern regarding how we presented the results. Consequently, we have cut down on the reiteration of the results in the text.

Reviewer: The discussion section is a lot of reiteration of the results which should be removed. The discussion section is to interpret the results only. In my opinion, for this manuscript to be published, there needs to be substantial re-organization of the results and discussion sections

Response: We are grateful to the reviewer for pointing this out. We have consequently removed most of the results that were appearing in the discussion. Only where we felt that it was necessary to reiterate the results have we left the results in.

Reviewer: Line 16 – To assess what type of gap? Are you referring to a knowledge gap here?

Response: Thank you for picking this up, we have provided clarification, and it now reads as follows: “gaps in the KAP towards rabies”

Reviewer: L20 –‘years of age’ should be used instead of ‘years’

Response: Change has been implemented as recommended

Reviewer: L20 – ‘validated’ would be more appropriate to use instead of ‘pre-tested’ here.

Response: pre-tested has been replaced with validated

Reviewer: L21 – you can remove the alpha here to save space in the abstract. It generally is not needed in an abstract.

Response: Alpha has been removed as recommended.

Reviewer: L22 and L23 and L27, L28– Please include the numerator and denominator when providing percentages.

Response: We were not sure whether to respond to this suggestion given that the reviewer wanted us to limit reiterating the results in the text. Since the numerator is in the table, we thought it was not necessary to repeat each one of them it in the text. But nonetheless, we have adopted the recommendation made by the reviewer.

Comment: L27 – ‘said’ vs. ‘mentioned’. The word mention indicates an afterthought – that of which your participants didn’t respond with.

Response: Mentioned was replaced by “said”.

Comment: L29 – Please include percentage, numerator and denominator.

Response: We have included the percentages, numerator and denominator as requested.

Reviewer: L28 – please remove ‘on the other hand’ as this is not scientific language. I also recommend removing ‘greatest’ as this is subjective qualifier. I recommend rephrasing to ‘However, they pose the threat of transmitting rabies’.

Response: Both ‘on the other hand’ and ‘greatest’, have been removed as advised.

Reviewer: L41 – the category ‘warm-blooded’ is not a scientific category and should not be used. I recommend just keeping it as ‘mammals’ (since all mammals are susceptible to rabies). I know a previously reviewer asked for the inclusion of this – but I highly disagree with it as it is not scientifically correct.

Response: the change has been made, and ‘warm-blooded’ has been replaced by “mammals”

Reviewer: L45 and L46 – Please combine these two sentences, they should not be separate.

Response: The two sentences have been combined.

Reviewer: L47 – 31,539 is not an approximate number (you are giving an exact number). I recommend either remove the word approximate or rounding the value to 31,500 to give an approximate. I recommend instead using a value from Africa (since that is where your study is taking place). Additionally, can you confirm that all of these deaths were directly attributed to rabies? If not, then this does not suggest a high rate of rabies. Additionally, ‘high burden’ is not scientific – please change to ‘high rate’.

Response: the word ‘approximate has been removed. However, we do not agree with the reviewer on the word burden not being scientific. In Epidemiology, the word burden is used to describe the impact of a disease on the population, and this includes morbidity and mortality. The word “rate” in Epidemiology has an element of time. So if we talk of rate of rabies, it means the number of rabies cases occurring over a specific period of time. As a result we decided not to replace burden with rate.

Reviewer: L52 – It would be more scientific to state mortality rates here – please also find a citation for this

Response: Reference has been added

Reviewer: L55 - you should mention doses of human rabies immune globulin (HRIG) here.

Response: We appreciate the reviewer’s suggestion on the number of injections. However, we would like to note that the number of injections of rabies immune globulin and vaccinations depend on the categorization of wound. It is not just one fixed number of injections. We feel that dwelling on the treatment of rabies cases will not improve on the paper. Since this is not the focus of the paper, we decided to refer the reader to documents where details of treatment of rabies in humans is described in detail. The following sentence has been added in line: “Details of the treatment that is prescribed for victims of dog bites to prevent development of the disease rabies, is described in details by Bishop et al (2010).”

Reviewer: L57- please give explicit values.

Response: We are not sure which figures the reviewer is referring to here. But if it is the number of vaccinations the victims received, refer to the comment above on treatment of rabies. The number of injections given depend on the category of the dog bite wound.

Reviewer: L 74 – 77 – This needs to be referenced.

Response: reference has been provided.

Reviewer: L78 – Please use the acronym KAP here since you’ve already introduced it.

Response: We acknowledge the suggestion by the reviewer. However, we are of the view that it is not good practice to begin a sentence with an abbreviation and/or acronym. As a result, we did not change to acronym KAP here.

Reviewer: L81 – Again, please use the acronym KAP here since you’ve already introduced it.

Response: the change has been implemented.

Reviewer: L105 – ‘was used’ instead of ‘was adopted’

Response: the change has been made

Reviewer: L105 – please use the acronym KAP here.

Response: Change implemented.

Reviewer: L135 - please use the acronym KAP here.

Response: Done

Reviewer: L147 – ‘was recorded using Microsoft Excel’

Response: implemented

Reviewer: L148 – How is there a ‘correct/incorrect response’ for each answer? I recommend rephrasing.

Response: Yes, there is a correct/incorrect response for each answer. But this specifically refers to the section on Knowledge. It would not have been possible to score the knowledge if we did not do it this way.

Reviewer: L182-184 – I don’t believe it’s necessary to mention this. Plans for dissemination is not relevant to the study.

Response: Thank you for the suggestion. We have removed the plans for dissemination.

Reviewer: Results – with all percentages given, the associated numerator and denominator need to be given in parathesis directly following the percentage.

Response: The numerator and denominator associated with percentage has been added where needed.

Reviewer: L207 – ‘route’ not ‘rout’

Response: Thanks for picking this up. We have implemented the suggestion.

Reviewer: Results – A lot of the results that are presented in the tables, are reiterated in the text. This fatigues the reader making the manuscript hard to read overall since it is very repetitive, and the authors need to cut the information down. Please reconsider what is in the text and what is presented in the tables.

Response: As mentioned earlier we have tried to minimise

Reviewer: L322 – please remove the word ‘generous’ – this is not to be used in scientific writing.

Response: We take note of the reviewer’s comment. However, in epidemiology, it is acceptable to use the phrase ‘generous’. We have used the word in several work we have published in this journal and others in the past.

Reviewer: L221 – please give the specific p-value instead of stating that it is > 0.05

Response: We acknowledge the reviewer’s comment. However, given that the variable is categorical with several levels, stating the exact p-value would look clumsy. The reader can refer to the table if in need of the exact p-value for each level.

Reviewer: Discussion – The discussion needs to be cut down considerably and the authors need to reconsider what information is pertinent to the study. At present, the discussion is currently 14 pages long, which is a lot. Overall, there is a lot of re-presenting the results which should not occur. The discussion should only be used to interpret the results and not presenting the results again. Additionally, there is too many instances where the authors are comparing their results to other study. It’s great to compare results, but the authors should consider judicious use of this and should instead focus on interpreting their own results.

Response: We have cut down the discussion, particularly by limiting reiterating the results in the discussion section to the extent possible without the paper being watered down. However, on the issue of limiting comparison of our findings and those of other authors, we are of the view that unlike the results section, the discussion should in addition to interpreting the findings, focus on locating findings of the study in the larger body of existing research. In view of this, we are of the view that compare results with those of other peer-reviewed papers is an important component of the discussion. We are thus reluctant to cut down on the comparison.

Reviewer: L338 – Please use the acronym KAP

Response: Done

Reviewer: L379 – I would argue that 27% is not a very low number – restate to something along the lines of ‘minority’

Response: The statement has been reviewed and changed

Reviewer: L383 – If you are stating results from another study, please also include the numerator and denominator so that the reader can know how many participants answered the questionnaire. Please fix here and throughout the rest of the manuscript.

Response: We have implemented the suggestion throughout the discussion.

Reviewer: L400 – 12% is not a ‘mere’ amount – re-state to say ‘minority’

Response: suggestion implemented as recommended.

Reviewer: L411 – Please use the acronym PEP

Response: Implemented as recommended.

Reviewer: L460 - remove ‘the’ at the start of the sentence.

Response: implemented as recommended.

Reviewer: L494 – “The number that did not know, even though small, cannot be ignored.” – This sentence is confusing, and I believe there is a typo. Please rephrase.

Response: The sentence has been rephrased to make it clearer. It now reads as follows: “From a public health standpoint, the fact that the overwhelming majority of respondents in this study knew that rabies can be transmitted from animals to humans is a significant finding. Likewise, even though the number of people who did not know that rabies can be transmitted from animals to humans was small, it should be considered a serious public health implication.’

Reviewer: L639 – “Just as was observed” is a confusing way to start a sentence – please rephrase.

Response: The sentence as been rephrased and reads as follows: “In a Filipinos study it was observed that 64% of the respondents consistently vaccinated their dogs to prevent rabies [10].

Reviewer: L684 – ‘latter’ vs ‘later’

Response: Thanks for picking this -the correction has been made

Reviewer: Figures and table captions – I recommend to the authors that they modify the captions for the figures and captions so that they can be understood without reference to the text.

Response: We have modified the captions of figures and tables.

Reviewer: Figures – I highly recommend to the authors that they include a map of their study site. This would be helpful for the reader to understand.

Response: We would like to thank the reviewer for the suggestion. We had planned to include a map from the onset, did an extensive search for a suitable map and failed to get one. That is why we provide the GPS coordinates of the two areas where the study was conducted.

Attachment Submitted filename: Rebuttal.docx

# Peer review: Decision Letter 4

**DOI:** 10.1371/journal.pone.0352279.r009

**Authors:** Raúl Alejandro Alegría-Morán

3 Mar 2025

PONE-D-23-27745R4

Knowledge, attitude and practices towards rabies: A community survey in selected areas of KwaZulu-Natal Province, South Africa

PLOS ONE

Dear Dr. Oguttu,

Thank you for submitting your manuscript to PLOS ONE. After careful consideration, we feel that it has merit but does not fully meet PLOS ONE’s publication criteria as it currently stands. Therefore, we invite you to submit a revised version of the manuscript that addresses the points raised during the review process.

==============================

Please place particular emphasis on the comments and suggestions made by Reviewer 6 and the details in the attachment from Reviewer 4. Include any supplementary materials that were suggested by the reviewers, as well as consider the limitations of the study and include them in the manuscript.

==============================

Please submit your revised manuscript by Apr 17 2025 11:59PM. If you will need more time than this to complete your revisions, please reply to this message or contact the journal office at plosone@plos.org. When you're ready to submit your revision, log on to https://www.editorialmanager.com/pone/ and select the 'Submissions Needing Revision' folder to locate your manuscript file.

Please include the following items when submitting your revised manuscript:

- A rebuttal letter that responds to each point raised by the academic editor and reviewer(s). You should upload this letter as a separate file labeled 'Response to Reviewers'.
- A marked-up copy of your manuscript that highlights changes made to the original version. You should upload this as a separate file labeled 'Revised Manuscript with Track Changes'.
- An unmarked version of your revised paper without tracked changes. You should upload this as a separate file labeled 'Manuscript'.

If you would like to make changes to your financial disclosure, please include your updated statement in your cover letter. Guidelines for resubmitting your figure files are available below the reviewer comments at the end of this letter.

If applicable, we recommend that you deposit your laboratory protocols in protocols.io to enhance the reproducibility of your results. Protocols.io assigns your protocol its own identifier (DOI) so that it can be cited independently in the future. For instructions see: https://journals.plos.org/plosone/s/submission-guidelines#loc-laboratory-protocols. Additionally, PLOS ONE offers an option for publishing peer-reviewed Lab Protocol articles, which describe protocols hosted on protocols.io. Read more information on sharing protocols at https://plos.org/protocols?utm_medium=editorial-email&utm_source=authorletters&utm_campaign=protocols.

We look forward to receiving your revised manuscript.

Kind regards,

Raúl Alejandro Alegría-Morán, Ph.D.

Academic Editor

PLOS ONE

[Note: HTML markup is below. Please do not edit.]

Reviewers' comments:

Reviewer's Responses to Questions

**Comments to the Author**

1. If the authors have adequately addressed your comments raised in a previous round of review and you feel that this manuscript is now acceptable for publication, you may indicate that here to bypass the “Comments to the Author” section, enter your conflict of interest statement in the “Confidential to Editor” section, and submit your "Accept" recommendation.

Reviewer #4: (No Response)

Reviewer #6: (No Response)

**********

2. Is the manuscript technically sound, and do the data support the conclusions?

The manuscript must describe a technically sound piece of scientific research with data that supports the conclusions. Experiments must have been conducted rigorously, with appropriate controls, replication, and sample sizes. The conclusions must be drawn appropriately based on the data presented.

Reviewer #4: Yes

Reviewer #6: Yes

**********

3. Has the statistical analysis been performed appropriately and rigorously?

Reviewer #4: Yes

Reviewer #6: Yes

**********

4. Have the authors made all data underlying the findings in their manuscript fully available?

The PLOS Data policy requires authors to make all data underlying the findings described in their manuscript fully available without restriction, with rare exception (please refer to the Data Availability Statement in the manuscript PDF file). The data should be provided as part of the manuscript or its supporting information, or deposited to a public repository. For example, in addition to summary statistics, the data points behind means, medians and variance measures should be available. If there are restrictions on publicly sharing data—e.g. participant privacy or use of data from a third party—those must be specified.

Reviewer #4: No

Reviewer #6: Yes

**********

5. Is the manuscript presented in an intelligible fashion and written in standard English?

PLOS ONE does not copyedit accepted manuscripts, so the language in submitted articles must be clear, correct, and unambiguous. Any typographical or grammatical errors should be corrected at revision, so please note any specific errors here.

Reviewer #4: Yes

Reviewer #6: Yes

**********

6. Review Comments to the Author

Please use the space provided to explain your answers to the questions above. You may also include additional comments for the author, including concerns about dual publication, research ethics, or publication ethics. (Please upload your review as an attachment if it exceeds 20,000 characters)

Reviewer #4: Thank you for editing your manuscript. It has improved with the edits made. Howeverm I do have some edits remaining to be addressed. Thank you for the opportunity to review your manuscript.

Reviewer #6: Reviewer: Overall, I believe the authors present a well conducted a study on the knowledge,

attitudes and practices towards rabies study on two villages of the KwaZulu-Natal Province,

South Africa. The study identified some gaps in the knowledge, attitudes and practices

towards rabies from local populations. This information can be useful for policy makers and

for the implementation of rabies control programs.

Reviewer: I consider the manuscript to be lengthy, namely the results and discussion parts,

especially the last one. I would especially recommend a full review of the discussion part. I

consider that there is some repetition of the results in the discussion and for the manuscript

to be published there is a need to re-organize results and discussion. As of special

importance to the results – the percentage of vaccinated dogs – I think some clarifications

are needed on the way the percentage was calculated. I suggest also to include the

limitations within the discussion part and to provide the questionnaire in the supplementary

materials.

L 24: I suggest putting the comma before “which”

L 25 and 26: PEP includes also the vaccines given on the days 3, 7, 14 and 28 and not just

the one given at the first medical visit.

L 33, 34 and 35: I suggest rephrasing; especially the last part, I do not think it does a lot of

sense the way it is phrased, as it seems that those who do not own pets are included in the

dog owners.

L 42: I suggest adding a comma between “infection” and “generally”.

L 47: I suggest removing the comma after “making the two”

L 48: I suggest removing “While”

L 49: I suggest adding a comma after “annually”

L 49: I suggest adding “infection” after “rabies”.

L 53 and 54: I would suggest that you rephrase both, as it is especially true that symptoms of

rabies can develop in case victims fail to obtain PEP and not so much that victims succumb

to the disease after symptoms develop in case they failed to obtain PEP.

L 57: I suggest rephrasing. Although the development of symptoms will have much higher

chances to occur in those who did not take PEP, an exposure does not always lead to

infection and disease, even without the PEP. Therefore, I suggest changing it to “the loss of

life of untreated victims might occur”.

L 61: I am not sure if “mass campaigns” is the correct English expression. Perhaps “mass

vaccination of dogs against rabies”

L 65: I would suggest changing the expression “developing countries” to “low-and-middle

income countries”.

L 75, 76 and 77: Perhaps it can be merged in one sentence

L 80: Add the acronym “KAP” after “Knowledge, attitude and practices”. Also, do you consider

the word “attitude” or “attitudes” to be the most correct?

L 82: I suggest changing the “clinical signs and” to “clinical signs, “

L 84, 85 and 86: this sentence is not clear to me; could you please rephrase it?

L 87: Perhaps it can be added that KAP studies can be used also to identify gaps in

knowledge, attitudes and practices, allowing for the design of targeted campaigns to fill those

gaps

L 93: Perhaps you can refer here that the study was conducted in Embo and Verulam,

located in eThekwini District of KwaZulu-Natal province.

L 110: In my opinion you should state here that just those with 18 years old or more were part

of the target population. You can also justify the choice behind Embo and Verulam (or do it in

the discussion).

L 116: Can you please report the value of the sampling interval? Was it calculated by dividing

the population size by the sample size?

L 118 and 119: could you please explain a bit more of the sampling strategy here? Would you

count the houses following a road? In which direction? And what did you do when the road

finished? How was the first point chosen?

L 120 and 121: Were there any criteria – such as the household head – besides age of the

selection of the respondent in each house?

L 123: I suggest changing to “The sample size for the two study areas”

L 136: I suggest changing the comma after “data” to an “and”

L 138: Was there any process of translation and back translation or was it a simple

translation from English to isiZulu?

L 146: Would the word “answer” be more appropriate than “questionnaire”?

L 147: Was each question or answer that was coded?

L 160: I suggest having “Knowledge score”

L 161: are you talking about alpha or p value?

L 170: were the models compared at each removal step?

L 203 and 204: I do not understand well the beginning of the sentence. I suggest removing

“Although in the minority”. I also suggest to change to “by a chemical substance (2.47%,

xx/yy) or by insufficient…” . Furthermore, I can see in some cases where you kept “n=” and in

others where you do not have the denominator. Please standardize all.

L 217: I suggest changing “were okay” to “thought it was appropriate to get”

L 218: I suggest changing the “;” to a comma

L 220: add “where” after “are the places” and I suggest changing “injection” to “injections”

L 226: change the parenthesis after “%” to comma, as previously done.

L 230: I suggest removing the comma after “population”

L 233: I think it should be “were aware of the need”

L 246: change the comma to point before the decimal number. Check the text please,

because there are other situations where this happens.

L 252 on: From this line on you reduced the number of times the percentage of respondents

and absolute values are presented and used much more adjectives that express relative

dimensions, such as “majority”, “small” or “some”. This contrasts with the previous section

and seems to be a lack of consistency; furthermore, the stating of the numbers is much more

precise and objective. For these two reasons I would prefer to see those numbers stated

instead of the relative adjectives. Please always consider what should be presented in the

text, as you do not need to report everything in written format and can make use of tables

and graphs.

L 254 to 267: same comment as previous

L 269: I suggest removing the comma after “(303/768)”

L 273: I would prefer to see a number instead of “under 30%”, as I do not know what this

really means – it can go from 0 to 29.9%. As well, I think it would be better to see the number

of those who have pets instead of those who have not.

L 274: Is the percentage 53.9% correct or meaningful? If I understand correctly, the entire

number of respondents was used to calculate it, correct? As we are just talking about those

who have pets – and therefore the only ones who could neuter them – could you please

explain the rationale behind using the entire sample size as denominator?

L 275: I suggest adding “on” – “In response to the question on how often”

L276: I think it would be better to present the precise numbers instead of relative adjectives

L 279: I have doubts regarding the calculation of the percentage 59.77. The calculation used

the entire number of respondents as denominator, correct? If so, it does not make sense to

me. If part of the respondents did not have dogs, it could not be expected to get vaccination

certificates from them. So, the percentage of those who had certificates should be in relation

to those who have dogs, not to the entire number of respondents. As an example, if just 1%

had dogs, but all of them had a vaccination certificate, using the entire number of

respondents as denominator would result on stating that just 1% was able to produce a

vaccination certificate, while in fact 100% of those who could produce a vaccination

certificate had done it. But perhaps I am understanding it incorrectly.

L 282 and 283: Was this question asked to all the respondents?

L 284 and 285: Same question; Because it is written "their dogs", which leads me to think

that this question was just for pet owners

L 286 and 287: Same question; was this just asked to pet owners? If so, why is the total

number of respondents used as denominator?

L 294: I suggest to remove the word “bellow”

L 295 to 298: this sentence is not very clear to me; could you please rephrase it?

L 299 and 300: Perhaps "when asked what they would do in case of an encounter with a

suspected rabid stray dog just 53.5 (411/768) said that they would..."

Table 4: I am not sure if presenting the percentages calculated with the total number of

respondents as denominator are the best way to present the results from the questions

directed to those who had pets.

Table 4: I do not understand to where does the word “throw” refers too in the last question

L 307: I would prefer to see numbers instead of adjectives with relative meanings

L 317: p without capital; throughout the text and tables (like table 5) sometimes you have the

p value written in capital, sometimes not in capital. Please standardize.

L 322: On the value of the Odds ratio you use three decimal houses, while in the rest of the

cases you use two; please standardize.

L 322: Should it be “three times as likely” or “three times more likely”?

Reviewer: Discussion – I would suggest removing redundant or not so relevant information or

comparisons from the discussion. The authors compared their results with existing literature,

but I think it would be beneficial to choose to mainly discuss results that can be of special

relevance and to choose comparisons that are closer to the situation in KwaZulu-Natal

Province, such as other studies from South Africa. Furthermore, the authors often present

differences on findings, but without offering possible explanations for those differences. It is

good to compare the results with other studies, but frequently I do not understand why those

specific studies were chosen and, in many instances, I fail to understand what the main

reasons for these differences could be.

L 344: I have some doubts on discussing of the demographics on a section separately, as

done here. I think it would make more sense to be integrated in the discussion of the

differences in findings as possible explanations for those findings. Could you please explain

why did you opt for this way?

L 346 and 347: Could you please explain or give a reference why this age group is more

active regarding pet management?

L 347 to 349: Could this be influenced by the way respondents were selected? As there is no

information on how respondents within the household were selected, it is difficult to evaluate

the reasons behind this finding. If the person available would be the respondent, could it be

that woman were generally more at home than man, for example? Could you please explain

your statement?

L 354: Is it a high rate?

L 355: Perhaps you can compare it with studies from South Africa or neighbor countries -

https://pmc.ncbi.nlm.nih.gov/articles/PMC8929695/#sec015,

https://journals.plos.org/plosntds/article?,

https://www.authorea.com/users/889337/articles/1266757-knowledge-attitudes-andpractices-kap-of-rabies-prevention-among-communities-living-in-proximity-to-game-reservesin-the-eastern-cape-of-south-africa,

https://journals.plos.org/plosone/article?,

https://journals.plos.org/plosntds/article?id=10.1371/journal.pntd.0011631,

https://link.springer.com/article/10.1186/s12917-018-1604-z#Sec6 - especially this one, from

the same province

L 359: Perhaps “high dog ownership rate”

L 362 and 363: Could you please explain why you chose these two articles for the

comparison?

L 362 to 365: I am not sure if these paragraph makes much sense. You had not previously

used these two references as comparison, or did I miss it? If you did not, in my opinion it

would make more sense to point out the differences and then offer possible explanations for

the differences.

L 376: I suggest to remove the comma after “study”

L 381: Should it be “on the causes of rabies”?

L 391 to 395: Could you please explain if there was any particular reason to choose Ethiopia

and the Philippines as comparison?

L 391: “show” instead of “shows”

L 396: I suggest to remove the comma after “rabies”

L 397: for consistency, please add the numerator and denominator.

L 397: I suggest to change “Which” to “This is”

L 401 to 405: Perhaps you can cut the first sentence of the paragraph and move the second

one to the previous paragraph, following the sentence that finishes with "low number of

respondents (11.46%)".

L 406 to 421: Are the comparisons with these studies relevant? Are you trying to explain why

the differences exist, or just reporting them? In case of just reporting them, is this really

relevant for the objective of this study?

L 422: Please change “diseases” to “disease”

L 430: Again, I fail see how this comparison would be important. As I understand it, what is

important is that there are people who do not know that they should take the vaccine

immediately and therefore there would be important to have campaigns, for example,

focused on this. Could you please explain why do you think that having these comparisons is

important?

L 450: Please change the comma on the percentage to a point

L 507 to 512: This is a good point of comparison between the results in different studies.

L 547 to 551: It would be good to have data supporting these statements

L 594 to 599: If I understood correctly, only 559 respondents had dogs, correct? If 459 were

able to produce a vaccination certificate, this means that 82% of the dogs were vaccinated.

Or did I understand it incorrectly? If it is as I understood it, could you please re-write the

paragraph?

L 646 to 648: Here is the same problem with using the entire sample; if some of the

respondents do not have pets, it does not make sense to include them in the calculation.

L 651 to 653: I seems to me repeated, as you had already referred this before.

L 690: Several studies point education as a factor. Other studies included considerably more

factors in the analysis. In case you can think of any reasons, I would suggest that you

discuss the reason behind education not being a significant factor in this study and how other

potential factors could also have an influence in levels of knowledge.

L 731 to 740: Unless stated otherwise, I think the limitations should be included in the

discussion

Figure 2: I suggest changing the title of the y axis to “Percentage of respondents” as in

Figure 3.

Figure 2 and 3: could you pleas explain why is the color of the text different between both

graphs?

Figure 1: I do not understand the title of one of the bars “Equine and…”

**********

7. PLOS authors have the option to publish the peer review history of their article (what does this mean?). If published, this will include your full peer review and any attached files.

If you choose “no”, your identity will remain anonymous but your review may still be made public.

**Do you want your identity to be public for this peer review?**  For information about this choice, including consent withdrawal, please see our Privacy Policy.

Reviewer #4: No

Reviewer #6: **Yes:** Filipe Maximiano Sousa

**********

[NOTE: If reviewer comments were submitted as an attachment file, they will be attached to this email and accessible via the submission site. Please log into your account, locate the manuscript record, and check for the action link "View Attachments". If this link does not appear, there are no attachment files.]

While revising your submission, please upload your figure files to the Preflight Analysis and Conversion Engine (PACE) digital diagnostic tool, https://pacev2.apexcovantage.com/. PACE helps ensure that figures meet PLOS requirements. To use PACE, you must first register as a user. Registration is free. Then, login and navigate to the UPLOAD tab, where you will find detailed instructions on how to use the tool. If you encounter any issues or have any questions when using PACE, please email PLOS at figures@plos.org. Please note that Supporting Information files do not need this step.

Attachment Submitted filename: Review_declet_4.docx

# Author comment: Author response to Decision Letter 5

**DOI:** 10.1371/journal.pone.0352279.r010

1 Apr 2025

We would like to thank the reviewers for the detailed positive comments and suggestions. We have used them to enhance the quality of the paper. We have endeavoured to respond to each query and suggestion to the best of our ability. We hope you will find our responses acceptable.

Attachment Submitted filename: Rebuttal letter (1).docx

# Peer review: Decision Letter 5

**DOI:** 10.1371/journal.pone.0352279.r011

**Authors:** Raúl Alejandro Alegría-Morán

5 May 2025

PONE-D-23-27745R5

Knowledge, attitude and practices towards rabies: A community survey in selected areas of KwaZulu-Natal Province, South Africa

PLOS ONE

Dear Dr. Oguttu,

Thank you for submitting your manuscript to PLOS ONE. After careful consideration, we feel that it has merit but does not fully meet PLOS ONE’s publication criteria as it currently stands. Therefore, we invite you to submit a revised version of the manuscript that addresses the points raised during the review process.

==============================

**Please consider the comments and suggestion of both reviewers (4 and 6) in terms of length of results and discussion, and particularly on reviewer 6 in terms of the specific comments about discussion.**

==============================

Please submit your revised manuscript by Jun 19 2025 11:59PM. If you will need more time than this to complete your revisions, please reply to this message or contact the journal office at plosone@plos.org. When you're ready to submit your revision, log on to https://www.editorialmanager.com/pone/ and select the 'Submissions Needing Revision' folder to locate your manuscript file.

Please include the following items when submitting your revised manuscript:

- A rebuttal letter that responds to each point raised by the academic editor and reviewer(s). You should upload this letter as a separate file labeled 'Response to Reviewers'.
- A marked-up copy of your manuscript that highlights changes made to the original version. You should upload this as a separate file labeled 'Revised Manuscript with Track Changes'.
- An unmarked version of your revised paper without tracked changes. You should upload this as a separate file labeled 'Manuscript'.

If you would like to make changes to your financial disclosure, please include your updated statement in your cover letter. Guidelines for resubmitting your figure files are available below the reviewer comments at the end of this letter.

If applicable, we recommend that you deposit your laboratory protocols in protocols.io to enhance the reproducibility of your results. Protocols.io assigns your protocol its own identifier (DOI) so that it can be cited independently in the future. For instructions see: https://journals.plos.org/plosone/s/submission-guidelines#loc-laboratory-protocols. Additionally, PLOS ONE offers an option for publishing peer-reviewed Lab Protocol articles, which describe protocols hosted on protocols.io. Read more information on sharing protocols at https://plos.org/protocols?utm_medium=editorial-email&utm_source=authorletters&utm_campaign=protocols.

We look forward to receiving your revised manuscript.

Kind regards,

Raúl Alejandro Alegría-Morán, Ph.D.

Academic Editor

PLOS ONE

[Note: HTML markup is below. Please do not edit.]

Reviewers' comments:

Reviewer's Responses to Questions

**Comments to the Author**

1. If the authors have adequately addressed your comments raised in a previous round of review and you feel that this manuscript is now acceptable for publication, you may indicate that here to bypass the “Comments to the Author” section, enter your conflict of interest statement in the “Confidential to Editor” section, and submit your "Accept" recommendation.

Reviewer #4: (No Response)

Reviewer #6: (No Response)

**********

2. Is the manuscript technically sound, and do the data support the conclusions?

The manuscript must describe a technically sound piece of scientific research with data that supports the conclusions. Experiments must have been conducted rigorously, with appropriate controls, replication, and sample sizes. The conclusions must be drawn appropriately based on the data presented.

Reviewer #4: Yes

Reviewer #6: Yes

**********

3. Has the statistical analysis been performed appropriately and rigorously?

Reviewer #4: Yes

Reviewer #6: Yes

**********

4. Have the authors made all data underlying the findings in their manuscript fully available?

The PLOS Data policy requires authors to make all data underlying the findings described in their manuscript fully available without restriction, with rare exception (please refer to the Data Availability Statement in the manuscript PDF file). The data should be provided as part of the manuscript or its supporting information, or deposited to a public repository. For example, in addition to summary statistics, the data points behind means, medians and variance measures should be available. If there are restrictions on publicly sharing data—e.g. participant privacy or use of data from a third party—those must be specified.

Reviewer #4: No

Reviewer #6: Yes

**********

5. Is the manuscript presented in an intelligible fashion and written in standard English?

PLOS ONE does not copyedit accepted manuscripts, so the language in submitted articles must be clear, correct, and unambiguous. Any typographical or grammatical errors should be corrected at revision, so please note any specific errors here.

Reviewer #4: Yes

Reviewer #6: Yes

**********

6. Review Comments to the Author

Please use the space provided to explain your answers to the questions above. You may also include additional comments for the author, including concerns about dual publication, research ethics, or publication ethics. (Please upload your review as an attachment if it exceeds 20,000 characters)

Reviewer #4: Thank you for your edits - I have no further comments to the authors.

I still feel as though the results section is lengthy (as there is repetition of results in both the text and tables), and the discussion is also lengthy given that the authors overly refer to many other studies and fail to discuss the results in the context of their own study.

However, I understand that there are conflicting reviews, but I do maintain my position.

I will defer to the editor to proceed further however would be supportive of their decision

Reviewer #6: Dear authors, thank you for the answers to my queries and for editing the manuscript following the suggestions. I believe it has improved and, apologizing because I know that it has been a lengthy process of review, I still have some comments, questions and suggestions. I am mainly concerned with the discussion and how lengthy it is.

L 21: I suggest to remove the comma after “26.95% (207/768)”

L22: I had suggested to change the comma to before “Which”, not to after

L 23-25: Thank you for the explanation. I understand now the idea of the sentence; to make it clearer I would suggest having it as: “which can include, depending on the category of the bite wound, wound washing, a dose of human rabies immune globulin (HRIG), and rabies vaccine given at the time of the first medical visit.”

L 27: I suggest removing either “of” or “of respondents”. It does not make much sense to me to have “much dog owners of respondents”.

L 29-32: Thank you for rephrasing it; I would suggest changing “that” to “,which”

L 57: I suggest changing “is” to “are”, as the sentence starts with “Details” and remove “in details”, as it is already at the beginning of the sentence.

L 64: Perhaps change “mortalities” to “mortality”.

L 65: I suggest adding “the” before existence

L 75: I do not know if this is also present on the original version, but the pdf version I have has “Click or tap here to enter text.” written in the middle of this line.

L 71-77: Throughout the text province is sometimes with capital P and sometimes without capital (like also in lines 92 and 107). Please standardize.

L82: There are two commas after “effective”

L 82: I believe it should be “prevention and control measures of rabies”, instead of “prevention and control measures for rabies”.

L 86: I would suggest changing the second “the disease”, for “with it”.

L 88: Perhaps you can add “towards rabies” after “KAP”

L 89: I might be wrong, but it seems to me that there is a double space after “located”

L 188: Thank you for the explanation. Yes, I understand that the first point was selected randomly, but I think it would be useful to have a more specific explanation on how the point was selected randomly (ex: GPS point randomly generated, list of households, etc.). In case someone would like to repeat the study, it would be useful to explain clearly and in detail all the steps which will allow the repetition. Did the authors have the houses numbered like in reference 40 and drew the first number randomly? If so, I understand which directions were taken after the first house. If not, I would like to know please how you decided on the direction to take. In case the method was the same as in reference 40, I would suggest to add something like “we followed exactly the same approach as in the study by Kumar et al.”, just to make it more explicitly that exactly the same approach was followed and had the houses numbered. Personally, when I read an article, I like to fully and clearly understand all the methods followed.

L 122: Just to confirm, in case the household head was not present, the interviewers would just ask for whoever would like to or would be available to answer the questionnaire, is that?

L 155: I think it would be useful to describe what the threshold level was to consider each respondent knowledgeable or not knowledgeable. I assume the knowledge score would be calculated as the proportion of the score obtained over the total possible points obtained, correct?

L 164: I respectfully disagree, as I consider alpha and p value different and should not be used interchangeably. The value of alpha is the threshold defined by the authors as the level of significance, and therefore the defined threshold for rejecting the null hypothesis, while p is a probability resulting from the test done. Therefore, variables will be included from the univariable model to the multivariable model if p ≤ α (in this case, α= 0.2).

L 209: Yes, this formulation would be acceptable from my perspective. I suggest to keep the precise numbers for results considered more relevant and refer to the table in the case of those not so important.

L 218: On (5.34% (41/768) was used a second parenthesis (before 41), when in remaining similar cases, a comma was used. Please standardize.

L 221: I suggest adding “where” after the word “places”

L 253 on: I understand the effort to reduce the length of the results. I also believe that this is needed. Perhaps citing numbers for important results and referring to the ones considered less important to the tables might help.

L262: The percentage seems to be in a different font size

L 281: 147/559 is 26.2%. Why is the text just “twenty percent”?

L 279 on: I am puzzled by the number 559 used when referring to “only dogs” in many of the paragraphs. From what I understand, 559 would include houses that could have just a cat, correct (as it seems to me from the formulation on the table “Own a pet dog and/ or cat”) ? Therefore, it does not seem correct to me to constantly refer uniquely to dogs when the number 559 can include also households just with cats. Could also be interesting – if the number of households just owning cats is considerable – to see the differences between the percentage of cats and dogs vaccinated. I suppose the percentage of dogs vaccinated is higher.

L304: Please change “no” to “not”

L 306: I think the “rabid” would be more appropriate than “rabies”

Table 4: Why is the sum of the answers to question “How often can you vaccinate your dog?” not 100%?

Table 4: I suggest that to mark in thicker the lines separating the answers to each question, as it will make reading the table much easier.

Table 4: I understand the 27.21 percent value for those who do not have a pet on the table. As the sum of all is above 100%, it seems a bit strange at first sight, but I understand why is like that. Personally, I think that if you opt to remove that line from the questions just asked to pet owners it would be fine, but it is also ok from my side if it stays like that.

L 311-312: Could the authors please indicate in which table it is possible to see the percentage figure?

L 338: As in the line above is “rabies is caused” I would suggest to change “was” to “is”

L 336-349: It seems mainly to be a summary of the results. Being the paper lengthy, unless it was recommended by another reviewer or you really have a strong reason to keep it, I would just remove these lines, to avoid repetition and reduce the length. As pointed by other reviewers

L 351-353: Could you please explain why do you do this statement? Being that you do not state any specific criteria for the selection of the respondents, why do you associate the highest participation of this group to being more related to the household pets? Could there be other reasons, such as visiting time or day, which could have influenced this?

L 360-362: Could this difference be due to differences in occupation? For example, in areas inhabited mainly by farmers whose farm is close to the house or during non-farming season could be that males are more at home than in other seasons or occupations.

L 351-383: I understand my previous comment regarding the section “Demographic profile of respondents” was not very clear. I completely understand the will of the authors to place the study in the context of available literature. On my side, I do not see a big problem with having a more local focus. Results of KAP surveys and identified gaps can be dependent on so many factors, which are mainly locally based. Furthermore, as gaps are local, interventions to address these gaps will also be locally focused. I tend to believe that results from a KAP study are in a high degree related to the local context. We cannot identify a gap between a certain region or country and automatically assume that the same gap will be present in a very different country and region. KAP constructs are highly related to local contexts and therefore, as a tool that can be used as a first step to devise interventions to control rabies in that specific context, the results of the KAP survey are mainly locally dependent and focused. KAP methodology – this is, how to carry out or improve KAP surveys – could be something more relevant on a global context eventually. In my opinion, KAP results are generally very locally dependent and therefore its importance is mainly local. Surely there can be comparisons with other regions and countries, and in this situation my opinion is that it is more valuable to compare more similar situations to understand what the reasons could be in case there are differences than just comparing results from very different contexts. Overall, I would suggest cutting several of the comparisons with other countries and focusing more on the relevance of the results to rabies control in the province. Surely, comparisons can be relevant for certain results and in that case, I would suggest using more similar situations to the study area. That is why I suggested some of the references in my previous comment.

Respectfully, I fail to see the relevance of a considerable part of the text within the section “Demographic profile of respondents”. Unless these differences could be used in the discussion of different findings in terms of KAP levels, or practices, or attitudes, or specific knowledge, etc. For example, knowing that rabies is a viral disease, could it be that in the study carried out in Kombolcha there was a greater proportion of correct answers because the level of education could also be higher (at least the percentage of respondents with college diplomas is higher than in this study the percentage of respondents who finished tertiary education). This is what I meant by integrating the demographic profiles of respondents on the discussion about differences in KAP instead of doing separate comparisons for the demographic profiles. For example, is it very relevant to state uniquely that in this study the majority of the respondents is Christian and, in another study, done in Ethiopia most of the respondents was Muslim? Or comparing the percentage of respondents who owned dogs compared with the percentage of dog owners from a study in Cameroon, done on an area where a considerable part of the population is Muslim and therefore, who religious attitudes might have an impact on having a dog or not? As I said above, in my opinion the manuscript is lengthy, and I believe that referring uniquely to some of the different demographic characteristics when they can be potential factors influencing the differences registered in results would be more beneficial, instead of having an entire section of differences between the demographic characteristics of this study and some other studies done in another country.

L 384: I suggest changing to “Knowledge rabies disease”

L 423-434: In this case the authors compare the study results with two studies from Ethiopia, and even from the same region – both studies were carried out in different areas of the Amhara region. And they present very disparate results in terms of the percentage of respondents following bite wound washing practices. Probably due to different demographic, cultural or educational characteristics between both areas. But that is precisely why I have difficulties in seeing a high value of constant comparison of all the results with the results of a few studies done in a few other countries, some of them probably quite different from South Africa. This leads to a discussion with 15 pages, which I consider to be quite lengthy and more difficult to read.

I understand the will of the authors to place the results in a broader context, but as stated at the end of the introduction, KAP studies can be useful to identify gaps and to inform information campaigns or other control interventions. Therefore, I believe that a discussion more focused on the meaning of the results for the control of rabies in KwaZulu-Natal, with a couple of comparisons with the results of other studies, would be of higher value for the overall manuscript. I believe that for a policy maker or for someone wishing to implement control activities, a focus on what the results might mean to KwaZulu-Natal and some of the aspects that might need to be address would be more important and might get lost in the middle of so lengthy constant comparisons with results from a few studies from countries such as Ethiopia, Cameroon or Morocco.

L: 433: While sometimes there are two suggestions for the translation of the name of the city (Gondar or Gonder), “Gondar” would be the most commonly used and this is also the form the article title used. This instead of “Gondor”.

L 447: I suggest not having a paragraph here.

**********

7. PLOS authors have the option to publish the peer review history of their article (what does this mean?). If published, this will include your full peer review and any attached files.

If you choose “no”, your identity will remain anonymous but your review may still be made public.

**Do you want your identity to be public for this peer review?**  For information about this choice, including consent withdrawal, please see our Privacy Policy.

Reviewer #4: No

Reviewer #6: No

**********

[NOTE: If reviewer comments were submitted as an attachment file, they will be attached to this email and accessible via the submission site. Please log into your account, locate the manuscript record, and check for the action link "View Attachments". If this link does not appear, there are no attachment files.]

While revising your submission, please upload your figure files to the Preflight Analysis and Conversion Engine (PACE) digital diagnostic tool, https://pacev2.apexcovantage.com/. PACE helps ensure that figures meet PLOS requirements. To use PACE, you must first register as a user. Registration is free. Then, login and navigate to the UPLOAD tab, where you will find detailed instructions on how to use the tool. If you encounter any issues or have any questions when using PACE, please email PLOS at figures@plos.org. Please note that Supporting Information files do not need this step.

# Author comment: Author response to Decision Letter 6

**DOI:** 10.1371/journal.pone.0352279.r012

1 Jun 2025

Dear Reviewer

Many thanks for the comments-we found many of them to be relevant and helpful in improving the paper. We thus have tried to accomodate as many of your suggestions as possible and this has included: removing the discussion of the demographic profile of the respondents and integrating the demographic profiles in the discussion of KAP. We have also tried to cut down on the comparison but not entirely removed them because of our interest of placing the study in a wider international context as articulated in our response to your suggestion. This is of course in addition to the comments you provided for which we have submitted a rebuttal letter.

Attachment Submitted filename: Rebuttal letter.docx

# Peer review: Decision Letter 6

**DOI:** 10.1371/journal.pone.0352279.r013

**Authors:** Raúl Alejandro Alegría-Morán

22 Jul 2025

PONE-D-23-27745R6

Knowledge, attitude and practices towards rabies: A community survey in selected areas of KwaZulu-Natal Province, South Africa

PLOS ONE

Dear Dr. Oguttu,

Thank you for submitting your manuscript to PLOS ONE. After careful consideration, we feel that it has merit but does not fully meet PLOS ONE’s publication criteria as it currently stands. Therefore, we invite you to submit a revised version of the manuscript that addresses the points raised during the review process.

==============================

Please focus on the comments and suggestions highlighted by reviewers 6 and 7. It is understood that this has been a lengthy work and has gone through several rounds of review. However, it is still emphasized that the discussion is extremely lengthy and derivative, comparing each result with evidence from sites with very different contexts. It is recommended to focus on the most important results and provide context to sites with similar characteristics, in addition to the global perspective they have already provided. This, in addition to all the other suggestions from the reviewers.

==============================

Please submit your revised manuscript by Sep 05 2025 11:59PM. If you will need more time than this to complete your revisions, please reply to this message or contact the journal office at plosone@plos.org. When you're ready to submit your revision, log on to https://www.editorialmanager.com/pone/ and select the 'Submissions Needing Revision' folder to locate your manuscript file.

Please include the following items when submitting your revised manuscript:

- A rebuttal letter that responds to each point raised by the academic editor and reviewer(s). You should upload this letter as a separate file labeled 'Response to Reviewers'.
- A marked-up copy of your manuscript that highlights changes made to the original version. You should upload this as a separate file labeled 'Revised Manuscript with Track Changes'.
- An unmarked version of your revised paper without tracked changes. You should upload this as a separate file labeled 'Manuscript'.

If you would like to make changes to your financial disclosure, please include your updated statement in your cover letter. Guidelines for resubmitting your figure files are available below the reviewer comments at the end of this letter.

If applicable, we recommend that you deposit your laboratory protocols in protocols.io to enhance the reproducibility of your results. Protocols.io assigns your protocol its own identifier (DOI) so that it can be cited independently in the future. For instructions see: https://journals.plos.org/plosone/s/submission-guidelines#loc-laboratory-protocols. Additionally, PLOS ONE offers an option for publishing peer-reviewed Lab Protocol articles, which describe protocols hosted on protocols.io. Read more information on sharing protocols at https://plos.org/protocols?utm_medium=editorial-email&utm_source=authorletters&utm_campaign=protocols.

We look forward to receiving your revised manuscript.

Kind regards,

Raúl Alejandro Alegría-Morán, Ph.D.

Academic Editor

PLOS ONE

Journal Requirements:

If the reviewer comments include a recommendation to cite specific previously published works, please review and evaluate these publications to determine whether they are relevant and should be cited. There is no requirement to cite these works unless the editor has indicated otherwise.

[Note: HTML markup is below. Please do not edit.]

Reviewers' comments:

Reviewer's Responses to Questions

**Comments to the Author**

1. If the authors have adequately addressed your comments raised in a previous round of review and you feel that this manuscript is now acceptable for publication, you may indicate that here to bypass the “Comments to the Author” section, enter your conflict of interest statement in the “Confidential to Editor” section, and submit your "Accept" recommendation.

Reviewer #6: (No Response)

Reviewer #7: (No Response)

**********

2. Is the manuscript technically sound, and do the data support the conclusions?

The manuscript must describe a technically sound piece of scientific research with data that supports the conclusions. Experiments must have been conducted rigorously, with appropriate controls, replication, and sample sizes. The conclusions must be drawn appropriately based on the data presented.

Reviewer #6: Yes

Reviewer #7: Yes

**********

3. Has the statistical analysis been performed appropriately and rigorously?

Reviewer #6: Yes

Reviewer #7: Yes

**********

4. Have the authors made all data underlying the findings in their manuscript fully available?

The PLOS Data policy requires authors to make all data underlying the findings described in their manuscript fully available without restriction, with rare exception (please refer to the Data Availability Statement in the manuscript PDF file). The data should be provided as part of the manuscript or its supporting information, or deposited to a public repository. For example, in addition to summary statistics, the data points behind means, medians and variance measures should be available. If there are restrictions on publicly sharing data—e.g. participant privacy or use of data from a third party—those must be specified.

Reviewer #6: Yes

Reviewer #7: Yes

**********

5. Is the manuscript presented in an intelligible fashion and written in standard English?

PLOS ONE does not copyedit accepted manuscripts, so the language in submitted articles must be clear, correct, and unambiguous. Any typographical or grammatical errors should be corrected at revision, so please note any specific errors here.

Reviewer #6: Yes

Reviewer #7: Yes

**********

6. Review Comments to the Author

Please use the space provided to explain your answers to the questions above. You may also include additional comments for the author, including concerns about dual publication, research ethics, or publication ethics. (Please upload your review as an attachment if it exceeds 20,000 characters)

Reviewer #6: Dear authors, thank you for the answers to my queries and for editing the manuscript following the suggestions. I believe it has improved and, apologizing because I know that it has been a lengthy process of review, I still have some comments, questions and suggestions. I am mainly concerned with the discussion and how lengthy it is. Furthermore, I think it would be helpful to provide the questionnaire in the supplementary materials.

L 80-81: I suggest to change to “In addition, for rabies’ prevention and control measures to be effective…”

L 85: I would suggest changing the second “the disease”, for “with it”.

L 116: I suggest changing “this sample size” to “the sample size”.

L 120; I suggest to add “the” before “community” – so it would be “from the community”. In this case, as it is more than one, even maybe “from the communities”.

L 140: Thank you for a more precise explanation of the sample method used. I think it is much more understandable now. I would suggest to change the order between the sentence in lines 142-143 and the one in lines 140-142.

L 161: I suggest to change to “ was given to an incorrect answer”.

L173: I believe it should be “Variables with p value ≤ 0.20”, or “Variables with a p value equal or under the alpha value of 0.2”.

L 199: I think the sentence formulation should be different. We already know from the methods that 768 were invited to participate. As we are in the results section I would argue that “were included in the study” would be more appropriate.

L 207: Actually, I guess it is more than 50%, as those who stated it is a chemical substance or psychological problem also did not know that it is caused by a virus. So I suggest that either you add up those who did not know at all the cause or stated a wrong cause, or you change the formulation of the sentence to “half of the respondents were not aware of the cause of rabies”. I think that probably the first option is better as then you can remove the second part of the sentence. Or you just state that only 26.9% knew that rabies is caused by a virus and you remove the first part of the sentence.

L222: I suggest to change “(12.89%, 99/768).” to after “some respondents”, as generally in the text you indicate the percentage and number immediately after stating “most, some”, etc.

L308: I suggest to remove the comma after “confrontation”.

L311: Separating the percentage from the “411/768” you have a percentage, while in the other cases you have generally a comma. Please ensure you always have a comma.

L 296: I have to go back to the question of the 559, those who owned a dog or just a cat and how the questions were formulated in the questionnaire.

I start to ask how were the questions regarding vaccination, keeping on a fenced place, going out of the yard unsupervised, vaccinating during annual vaccination campaigns, asked to the respondents. Were they asked “do you this to your animal/pet” or were they asked as it appears on Table 4? If they were asked as it appears on Table 4, where one of the question refers to “pet” (about sterilization) and the remaining to “dogs”, then I would assume that 559 cannot be the total denominator for many of those questions. If I just have a cat and someone asks me if I leave my dog to go out unsupervised, I will either answer that does not make sense or will answer no, as I do not have a dog. So first, I would like to understand what was exactly asked to the respondents regarding the vaccination campaigns, going out of the yard, etc. In case the respondents were all asked about “their pets” and not just “their dog”, I would kindly request the authors to standardize it all over the text and tables. The way it reads now is confusing, as sometimes it refers to pets (cats or dogs) and sometimes just to dogs. If should be unequivocal to what the authors are referring always to both cats and dogs and currently is not, because sometimes the authors refer to pets and sometimes the authors refer to dogs, which are not the same. And we have to be careful about this, as often KAP studies on rabies will just focus on dogs. Therefore, it is necessary to state if both cats and dogs are being always considered in all the questions and have that in a uniform way throughout the paper.

As an example, Line 301-302 is very confusing – the authors start to refer to the owners who own dogs and then refer the percentage of dog owners who vaccinate their pets during annual campaigns. It is not clear to the reader if this question was just asked to dog owners or to owners of cats and dogs, but the way it reads would lead reads to conclude that it was asked just to owners of dogs.

Table 4: The authors stated having removed the 27.21% for those who did not have pets, but did it just for some questions and not to all. I would think that either should be removed to all of those questions just asked to pet owners or to keep it in all, but not to remove in some and keep it in others.

Discussion: I acknowledge the efforts the authors did to improve and address the comments regarding the discussion, but in my opinion the discussion is still too long. The paper has 33 pages, of which around 13 are discussion. I apologize, but I fail to see the value of just stating values from a couple of studies done in different countries and contexts. In my opinion, for the study to be published, it needs a thoroughly review. I would suggest choosing important findings and to place more importance on what they mean for the local context.

Reviewer #7: The manuscript has undergone multiple rounds of review, and the authors have made several revisions accordingly. However, not all reviewer comments have been fully addressed. As noted by other reviewers, the discussion section remains somewhat lengthy and could be further condensed to focus more directly on the study’s key findings. It is not always necessary to compare each point /finding with other studies. Instead, emphasis should be put on interpreting/discussing the current findings and providing relevant recommendations or suggestions based on them. However, you can compare it with few key studies.

I am selecting one paragraph as an example to guide the authors on how to shorten and focus their discussion accordingly. Please revisit the discussion section again.

Example: Line 369 – Licking of an open wound discussion. It is not necessary to compare the findings from Haiti. I would suggest to revise like this:

“Licking of open wounds by rabies suspected animals was not considered a significant route of transmission in the present study. This suggests that if a dog licked a person in the study area, the individual might not seek medical attention. Such behavior could increase the risk of rabies infection. We recommend educating the public that, although rare, rabies can also be transmitted through the licking of open wounds by infected animals”.

I have few other comments in general

• Table 1 delete 95% CI data/column and is not necessary

• The authors have used the word "transfer/transferred" in a few of the sentences to indicate the transmission of rabies virus. In scientific/medical writing, transmit/transmitted is the standard term used in epidemiology and virology to describe how diseases spread and transmitted.

Example: The rabies virus is transmitted through a bite from an infected animal and NOT rabies virus was transferred through a bite…. Please change the word transfer to transmit or transmitted accordingly in the manuscript.

• The word “spread” of rabies virus is also used inappropriately. Please correct it accordingly.

• The word "eradicate/eradication" is also used inappropriately in the text. There is a difference between eradication, elimination, prevention and control. It will be difficult to eradicate rabies virus. We should be aiming to eliminate dog-mediated human rabies deaths.

• Word “put down” –is it not appropriate to use humanely euthanize the animal.

doing a good job…please revise

• I can also see few sentences ….there is a room for improvement …..provide specific recommendations.

**********

7. PLOS authors have the option to publish the peer review history of their article (what does this mean?). If published, this will include your full peer review and any attached files.

If you choose “no”, your identity will remain anonymous but your review may still be made public.

**Do you want your identity to be public for this peer review?**  For information about this choice, including consent withdrawal, please see our Privacy Policy.

Reviewer #6: No

Reviewer #7: No

**********

[NOTE: If reviewer comments were submitted as an attachment file, they will be attached to this email and accessible via the submission site. Please log into your account, locate the manuscript record, and check for the action link "View Attachments". If this link does not appear, there are no attachment files.]

While revising your submission, please upload your figure files to the Preflight Analysis and Conversion Engine (PACE) digital diagnostic tool, https://pacev2.apexcovantage.com/. PACE helps ensure that figures meet PLOS requirements. To use PACE, you must first register as a user. Registration is free. Then, login and navigate to the UPLOAD tab, where you will find detailed instructions on how to use the tool. If you encounter any issues or have any questions when using PACE, please email PLOS at figures@plos.org. Please note that Supporting Information files do not need this step.

# Author comment: Author response to Decision Letter 7

**DOI:** 10.1371/journal.pone.0352279.r014

9 Dec 2025

A rebuttal letter with line by line response to the reviewers has been prepared and is attached as a file.

# Peer review: Decision Letter 7

**DOI:** 10.1371/journal.pone.0352279.r015

**Authors:** Venu Ramkrishna Shah

27 Apr 2026

PONE-D-23-27745R7

Knowledge, attitude and practices towards rabies: A community survey in selected areas of KwaZulu-Natal Province, South Africa

PLOS One

Dear Dr. Oguttu,

Thank you for submitting your manuscript to PLOS ONE. After careful consideration, we feel that it has merit but does not fully meet PLOS ONE’s publication criteria as it currently stands. Therefore, we invite you to submit a revised version of the manuscript that addresses the points raised during the review process.

Following corrections are required:

1. Reframe the Title of Table 5 to make it more scientific

2. Add years in bracket where Age groups are given in tables.

3. Check for consistency in mentioning p value in main text, tables and in abstract.

Also do revision as per the comments received from Reviewer 7 as mentioned below.

Please submit your revised manuscript by Jun 11 2026 11:59PM. If you will need more time than this to complete your revisions, please reply to this message or contact the journal office at plosone@plos.org. When you're ready to submit your revision, log on to https://www.editorialmanager.com/pone/ and select the 'Submissions Needing Revision' folder to locate your manuscript file.

Please include the following items when submitting your revised manuscript:

- A letter that responds to each point raised by the academic editor and reviewer(s). You should upload this letter as a separate file labeled 'Response to Reviewers'.
- A marked-up copy of your manuscript that highlights changes made to the original version. You should upload this as a separate file labeled 'Revised Manuscript with Track Changes'.
- An unmarked version of your revised paper without tracked changes. You should upload this as a separate file labeled 'Manuscript'.

If you would like to make changes to your financial disclosure, please include your updated statement in your cover letter. Guidelines for resubmitting your figure files are available below the reviewer comments at the end of this letter.

If applicable, we recommend that you deposit your laboratory protocols in protocols.io to enhance the reproducibility of your results. Protocols.io assigns your protocol its own identifier (DOI) so that it can be cited independently in the future. For instructions see: https://journals.plos.org/plosone/s/submission-guidelines#loc-laboratory-protocols. Additionally, PLOS ONE offers an option for publishing peer-reviewed Lab Protocol articles, which describe protocols hosted on protocols.io. Read more information on sharing protocols at https://plos.org/protocols?utm_medium=editorial-email&utm_source=authorletters&utm_campaign=protocols.

As the corresponding author, your ORCID iD is verified in the submission system and will appear in the published article. PLOS supports the use of ORCID, and we encourage all coauthors to register for an ORCID iD and use it as well. Please encourage your coauthors to verify their ORCID iD within the submission system before final acceptance, as unverified ORCID iDs will not appear in the published article. *Only*  the individual author can complete the verification step; PLOS staff *cannot*  verify ORCID iDs on behalf of authors.

We look forward to receiving your revised manuscript.

Kind regards,

Venu Ramkrishna Shah

Academic Editor

PLOS One

Journal Requirements:

If the reviewer comments include a recommendation to cite specific previously published works, please review and evaluate these publications to determine whether they are relevant and should be cited. There is no requirement to cite these works unless the editor has indicated otherwise.

Please review your reference list to ensure that it is complete and correct. If you have cited papers that have been retracted, please include the rationale for doing so in the manuscript text, or remove these references and replace them with relevant current references. Any changes to the reference list should be mentioned in the rebuttal letter that accompanies your revised manuscript. If you need to cite a retracted article, indicate the article’s retracted status in the References list and also include a citation and full reference for the retraction notice.

Additional Editor Comments (if provided):

Please include following corrections in the manuscript.

1. In the tables, where Age is mentioned, authors need to add (Years) in the bracket.

2. Table 5, Title can be reframed appropriately. Also the text states that religion and educational level were included in the multivariable model initially. However, they are not present in Table 5. This implies they were removed during the backward selection method. The table or its caption should explicitly state that only the variables retained in the final model are shown. The abstract mentions a p-value of <0.005 for dog ownership , but Table 5 provides the more precise value of <0.0001. The abstract should be updated to reflect the specific significance found in the final model.

[Note: HTML markup is below. Please do not edit.]

Reviewers' comments:

Reviewer's Responses to Questions

**Comments to the Author**

1. If the authors have adequately addressed your comments raised in a previous round of review and you feel that this manuscript is now acceptable for publication, you may indicate that here to bypass the “Comments to the Author” section, enter your conflict of interest statement in the “Confidential to Editor” section, and submit your "Accept" recommendation.

Reviewer #7: (No Response)

**********

2. Is the manuscript technically sound, and do the data support the conclusions?

The manuscript must describe a technically sound piece of scientific research with data that supports the conclusions. Experiments must have been conducted rigorously, with appropriate controls, replication, and sample sizes. The conclusions must be drawn appropriately based on the data presented.

Reviewer #7: Yes

**********

3. Has the statistical analysis been performed appropriately and rigorously?

Reviewer #7: Yes

**********

4. Have the authors made all data underlying the findings in their manuscript fully available?

The PLOS Data policy requires authors to make all data underlying the findings described in their manuscript fully available without restriction, with rare exception (please refer to the Data Availability Statement in the manuscript PDF file). The data should be provided as part of the manuscript or its supporting information, or deposited to a public repository. For example, in addition to summary statistics, the data points behind means, medians and variance measures should be available. If there are restrictions on publicly sharing data—e.g. participant privacy or use of data from a third party—those must be specified.

Reviewer #7: Yes

**********

5. Is the manuscript presented in an intelligible fashion and written in standard English?

PLOS ONE does not copyedit accepted manuscripts, so the language in submitted articles must be clear, correct, and unambiguous. Any typographical or grammatical errors should be corrected at revision, so please note any specific errors here.

Reviewer #7: Yes

**********

6. Review Comments to the Author

Please use the space provided to explain your answers to the questions above. You may also include additional comments for the author, including concerns about dual publication, research ethics, or publication ethics. (Please upload your review as an attachment if it exceeds 20,000 characters)

Reviewer #7: I commend the authors for their effort in improving the article through multiple revisions. I encourage them to further address the remaining shortcomings and refine the manuscript to meet academic standards. While most of the reviewers’ suggestions have been incorporated, some issues remain unresolved. My general comments pertain to the discussion section, particularly the use of phrases and sentence construction. Several sentences could be tightened for clarity and grammatical accuracy, for example, by avoiding repetition, correcting fragments, and smoothing transitions. While the manuscript is generally understandable but requires refinement into academic style. As it is not feasible to pinpoint all instances, the authors are advised to conduct a thorough review and implement corrections. I would advise authors to consider seeking professional proofreading support so that the review process is completed in time.

Few of my specific comments that I could pinpoint are outlined below.

Line 54-55: Without proper health care, the loss of life of untreated victims might occur.

It may be written as: In the absence of appropriate healthcare interventions, untreated individuals are at increased risk of death.

Line 56-58: Details of the treatment prescribed for victims of dog bites to prevent the development of the disease rabies are described by Bishop et al…

The word disease rabies is not correct. Pl check for these words and correct it accordingly.

It can be written as: “Details of the treatment prescribed for dog-bite victims to prevent the development of rabies have been described by Bishop et al.”.

In addition, WHO Expert Consultation on rabies, third edition 2018 provides clear guideline on prevention of rabies in humans.

Line 77-78: Knowledge, attitude and practices (KAP) studies are significant in obtaining

information on the subject of concern. What is significant?

It can be written as : Knowledge, Attitude, and Practices (KAP) studies constitute an important methodological approach for acquiring insights into the subject of concern.

Line 274-277: The expression “pulling down a dog” conveys meaning or may be understandable, but it does not reflect an academic style of writing. I have commented this in my previous review but was not corrected.

It should be replaced with either “euthanizing a dog suspected of having rabies” or “destroying a dog suspected of rabies infection”. Please change this in the discussion section if mentioned.

Line 353-356- This is a serious public health given that washing ….

Please qualify after public health. ---is it public health concern or public health issue

….virus from gaining entry to the victim and …..

It should be the victim’s body

Please restructure the sentence.

Line 367=368 -- if we are to eradicate rabies-related deaths.

Please change as: to prevent death

I have pointed out in my previous review that the word "eradicate/eradication" is used inappropriately in the manuscript. There is a difference between eradication, elimination, prevention and control. It will be difficult to eradicate rabies virus. We should be aiming to eliminate dog-mediated human rabies deaths. Please replace the word eradication with elimination or prevention or control wherever appropriate.

Line 372-378 – the word other species. Please add …other species of animals or wildlife

….extension to communities ---it should be extension education programe or awareness program.

Line 379-390- …The high number of respondents who were aware of the importance of vaccinating dogs to control rabies is good news. The expression good news is clear but is not a formal expression.

It can be written as: The high number of respondents who were aware of the importance of vaccinating dogs to control rabies is encouraging

Line 380-382. Vaccination of pets against rabies creates a barrier to diseases shared among pets and humans, given that they occupy the same space. …The word that creates a barrier is correct but slightly vague. In public health writing, terms like “reduces transmission” or “prevents spillover” are clearer.

It can be written as: Rabies vaccination in pets enhances herd immunity and disrupts viral transmission within animal populations and from animals to humans. OR

Vaccination of pets against rabies enhances herd immunity and helps interrupt the transmission chain of the rabies virus among animals, as well as between animals and humans.

Line 394-396 : the word welcome observation is not an academic style of writing, replace with an encouraging finding

It can be expressed as: The very high number of respondents (87.24%) who preferred to be treated at a human clinic or hospital after exposure to a dog bite in this study is an encouraging finding

Line 416-418: incomplete sentences. Please complete the sentence

Line 432-434: the words odd behaviour should be replaced with abnormal behaviours or unusual behaviours.

May be it can be expressed as: The high number of respondents who favoured isolating their animals or placing them under quarantine if they displayed abnormal behaviours, such as biting someone, is a very significant finding.

Line 437-438: putting down the dog ; and plus many . please correct these.

Line 446-451: Please revise

Lune 455: ….if the goal of eradicating dog mediated rabies death in humans is to be attained.

The goal is elimination of dog-mediated human rabies death by 2030, and not eradication. Please change

Line 446-459: …..which according to WHO is the minimum vaccination coverage needed to prevent rabies outbreaks in humans.

It is not about rabies outbreak in humans but increase herd immunity in dogs and then prevent rabies virus transmission within dogs or animals and prevent transmission to humans. Please correct this.

Line 466-470: please correct

Line 474: Filipino should be corrected as the Philippines

Line 481-486: the word problematic is not an academic style of expression and also the sentence is long.

It can be written as: Given that the study area is endemic for rabies, it is concerning that some people did not present their dogs for vaccination during each campaign. This may be due to individuals being unavailable because of work or other commitments, while others were unable to handle their dogs and therefore did not present them for vaccination.

Line 495-499: ….Much of the …..this sentence is not structured correctly.

I think it can be written as : “Although the findings are encouraging, more effort is required to ensure that carcasses of animals suspected of rabies are submitted for laboratory confirmation.”

Line 517-518: The study area has attained over 70% vaccination coverage recommended by the WHO to minimize the risk of rabies outbreaks in humans/communities.

As stated above, 70% vaccination coverage in dogs is to break the rabies virus transmission chain within dogs and prevent transmission to humans and not rabies outbreaks in humans.

Line 523-524—The role of measures Please restructure the sentence

**********

7. PLOS authors have the option to publish the peer review history of their article (what does this mean?). If published, this will include your full peer review and any attached files.

If you choose “no”, your identity will remain anonymous but your review may still be made public.

**Do you want your identity to be public for this peer review?**  For information about this choice, including consent withdrawal, please see our Privacy Policy.

Reviewer #7: No

**********

[NOTE: If reviewer comments were submitted as an attachment file, they will be attached to this email and accessible via the submission site. Please log into your account, locate the manuscript record, and check for the action link "View Attachments". If this link does not appear, there are no attachment files.]

To ensure your figures meet our technical requirements, please review our figure guidelines: https://journals.plos.org/plosone/s/figures

You may also use PLOS’s free figure tool, NAAS, to help you prepare publication quality figures: https://journals.plos.org/plosone/s/figures#loc-tools-for-figure-preparation

NAAS will assess whether your figures meet our technical requirements by comparing each figure against our figure specifications.

# Author comment: Author response to Decision Letter 8

**DOI:** 10.1371/journal.pone.0352279.r016

2 Jun 2026

Response to reviewers

Reframe the Title of Table 5 to make it more scientific

Response:

The title has been reframed and now reads as follows: “: Results of the analysis of factors correlated with being knowledgeable about rabies”

Add years in bracket where Age groups are given in tables.

Response:

“Year” has been against age groups in tables

Check for consistency in mentioning p value in main text, tables and in abstract

Response:

p value has been corrected to ensure consistency.

The table or its caption should explicitly state that only the variables retained in the final model are shown.

Response:

A footnote has been added below the table to reflect that only the variables retained in the final model are shown.

The abstract mentions a p-value of <0.005 for dog ownership, but Table 5 provides the more precise value of <0.0001. The abstract should be updated to reflect the specific significance found in the final model.

Response:

The abstract has been updated to reflect the specific significance found in the final model.

Reviewer #7:

I commend the authors for their effort in improving the article through multiple revisions. I encourage them to further address the remaining shortcomings and refine the manuscript to meet academic standards. While most of the reviewers’ suggestions have been incorporated, some issues remain unresolved. My general comments pertain to the discussion section, particularly the use of phrases and sentence construction. Several sentences could be tightened for clarity and grammatical accuracy, for example, by avoiding repetition, correcting fragments, and smoothing transitions. While the manuscript is generally understandable but requires refinement into academic style. As it is not feasible to pinpoint all instances, the authors are advised to conduct a thorough review and implement corrections. I would advise authors to consider seeking professional proofreading support so that the review process is completed in time.

Response:

We have reviewed the discussion section, with the use of phrases and sentence construction to try and tighten clarity and grammatical accuracy. We also engaged the professional proof English language editor to assist in this regard.

Line 54-55: Without proper health care, the loss of life of untreated victims might occur.

It may be written as: In the absence of appropriate healthcare interventions, untreated individuals are at increased risk of death.

Response:

We appreciate the reviewer’s suggestion, and the sentence has been change to read as suggested, and the sentence now reads as suggested by the reviewer.

Line 56-58: Details of the treatment prescribed for victims of dog bites to prevent the development of the disease rabies are described by Bishop et al…

The word disease rabies is not correct. Pl check for these words and correct it accordingly.

It can be written as: “Details of the treatment prescribed for dog-bite victims to prevent the development of rabies have been described by Bishop et al.”.

Response:

We have rephrased the sentence, and it now reads as suggested by the reviewer.

In addition, WHO Expert Consultation on rabies, third edition 2018 provides clear guideline on prevention of rabies in humans.

Response:

The sentence has been changed accordingly as advised by the reviewer.

Line 77-78: Knowledge, attitude and practices (KAP) studies are significant in obtaining

information on the subject of concern. What is significant?

It can be written as : Knowledge, Attitude, and Practices (KAP) studies constitute an important methodological approach for acquiring insights into the subject of concern.

Response:

Thanks for the suggestion-we have rephrased the sentence to read as recommended by the reviewer.

Line 274-277: The expression “pulling down a dog” conveys meaning or may be understandable, but it does not reflect an academic style of writing. I have commented this in my previous review but was not corrected.

It should be replaced with either “euthanizing a dog suspected of having rabies” or “destroying a dog suspected of rabies infection”. Please change this in the discussion section if mentioned.

Response:

Thanks for the recommendation. The phrase “put down a dog”, has been rephrased and wherever it appears in the document has been corrected. It has been replaced with “euthanizing” or “destroying the dog”

Line 353-356- This is a serious public health given that washing ….

Please qualify after public health. ---is it public health concern or public health issue

….virus from gaining entry to the victim and …..

It should be the victim’s body

Please restructure the sentence.

Rephrase: the sentence has been restructured to qualify after public health by adding “concern” after public health.

Line 367=368 -- if we are to eradicate rabies-related deaths.

Please change as: to prevent death

I have pointed out in my previous review that the word "eradicate/eradication" is used inappropriately in the manuscript. There is a difference between eradication, elimination, prevention and control. It will be difficult to eradicate rabies virus. We should be aiming to eliminate dog-mediated human rabies deaths. Please replace the word eradication with elimination or prevention or control wherever appropriate.

Response: The reviewer’s concern is noted and wherever the word eradicate appears it has been replaced with “prevent”

Line 372-378 – the word other species. Please add …other species of animals or wildlife

….extension to communities ---it should be extension education programe or awareness program.

Responses:

The sentence has been restructured by adding “other species of animals” as recommended by the reviewer.

Line 379-390- …The high number of respondents who were aware of the importance of vaccinating dogs to control rabies is good news. The expression good news is clear but is not a formal expression.

It can be written as: The high number of respondents who were aware of the importance of vaccinating dogs to control rabies is encouraging

Response:

The recommendation has been adopted, and the sentence now reads as follows: “The high number of respondents who were aware of the importance of vaccinating dogs to control rabies is encouraging.”

Line 380-382. Vaccination of pets against rabies creates a barrier to diseases shared among pets and humans, given that they occupy the same space. …The word that creates a barrier is correct but slightly vague. In public health writing, terms like “reduces transmission” or “prevents spillover” are clearer.

It can be written as: Rabies vaccination in pets enhances herd immunity and disrupts viral transmission within animal populations and from animals to humans. OR

Vaccination of pets against rabies enhances herd immunity and helps interrupt the transmission chain of the rabies virus among animals, as well as between animals and humans.

Response:

Thanks for the suggestion. The sentence has been restructured by adopting the recommendation by the reviewer.

Line 394-396 : the word welcome observation is not an academic style of writing, replace with an encouraging finding

It can be expressed as: The very high number of respondents (87.24%) who preferred to be treated at a human clinic or hospital after exposure to a dog bite in this study is an encouraging finding

Response:

The sentence has been changed to read as the reviewer has recommended.

Line 416-418: incomplete sentences. Please complete the sentence

Response:

The sentence has been rewritten and now reads as follows: “This means that dogs presenting with paralysis are likely to be overlooked as rabies suspected cases. Therefore, if a rabid dog showing paralytic signs, was to bite a person, such a person is likely not to seek PEP, which increases the risk of contracting rabies among the respondents.”

Line 432-434: the words odd behaviour should be replaced with abnormal behaviours or unusual behaviours.

Maybe it can be expressed as: The high number of respondents who favoured isolating their animals or placing them under quarantine if they displayed abnormal behaviours, such as biting someone, is a very significant finding.

Response:

The reviewer’s suggestion has been adopted.

Line 437-438: putting down the dog ; and plus many . please correct these.

Response:

The phrase putting down the dog has been changed through out the document.

Line 446-451: Please revise

Lune 455: ….if the goal of eradicating dog mediated rabies death in humans is to be attained.

The goal is elimination of dog-mediated human rabies death by 2030, and not eradication. Please change

Response:

The sentence has been restructured, and eradicating has been replaced elimination.

Line 446-459: …..which according to WHO is the minimum vaccination coverage needed to prevent rabies outbreaks in humans.

It is not about rabies outbreak in humans but increase herd immunity in dogs and then prevent rabies virus transmission within dogs or animals and prevent transmission to humans. Please correct this.

Response:

Thanks for the suggestion-we have made the relevant changes to reflect the reviewer’s suggestion.

Line 466-470: please correct

Response:

The paragraph has been reviewed and now reads as follows:

While in the study by Shirsuphal [41], 84% (56/67) of participants did not let their dogs to go outside the homestead, very few participants in the present study indicated that they kept their dogs in an area that is fenced off and does not allow dogs to go outside the compound. This observation is a serious public health concern, because when domestic dogs are let to roam freely, they frequently encounter stray animals that might rabid. This heightens the risk of these dogs contracting the rabies virus. Therefore, confinement of dogs to minimize domestic dogs encountering stray dogs or wild animals in the study area, needs to be improved on.

Line 474: Filipino should be corrected as the Philippines

Response:

The change has been implemented as suggested by the reviewer.

Line 481-486: the word problematic is not an academic style of expression and also the sentence is long.

It can be written as: Given that the study area is endemic for rabies, it is concerning that some people did not present their dogs for vaccination during each campaign. This may be due to individuals being unavailable because of work or other commitments, while others were unable to handle their dogs and therefore did not present them for vaccination.

Response:

The sentence has been modified by adopting the suggestion by the reviewer.

Line 495-499: ….Much of the …..this sentence is not structured correctly.

I think it can be written as : “Although the findings are encouraging, more effort is required to ensure that carcasses of animals suspected of rabies are submitted for laboratory confirmation.”

Response:

The reviewer’s suggestion has been implemented and now the sentence reads as suggested by the reviewer.

Line 517-518: The study area has attained over 70% vaccination coverage recommended by the WHO to minimize the risk of rabies outbreaks in humans/communities.

As stated above, 70% vaccination coverage in dogs is to break the rabies virus transmission chain within dogs and prevent transmission to humans and not rabies outbreaks in humans.

Response:

The sentence as been changed accordingly and now reads as follows: “The study area has attained over 70% vaccination coverage, which according to the WHO is needed to break the rabies virus transmission chain within dogs and prevent transmission to humans.”

Line 523-524—The role of measures Please restructure the sentence

Response:

The whole paragraph has been restructured and now reads as follows: “The identified knowledge gaps include poor knowledge of the cause of rabies, how one can protect oneself from contracting rabies, precautionary measures to adopt following exposure to rabies and how to protect susceptible animals from being exposed to rabies. Measures such erecting fences around homesteads to restrict animal movements and neutering of pets to control dog population need to be emphasized during awareness programs.”

We would like to thank the reviewers for all their input that have helped improve on the paper. We have attended to all the comments and suggestions and how we responded is outlined above. In addition, the manuscript was sent for editing by a professional English language editor. In the version with track changes the words in blue is what the editor suggested and the paragraph highlight yellow are paragraphs on which we as authors worked on while implementing the comments and suggestions from reviewer 7 and the Editor.

Attachment Submitted filename: Rebuttal Letter -Revision 8.docx

# Editor report: Decision Letter 8

**DOI:** 10.1371/journal.pone.0352279.r017

**Authors:** Venu Ramkrishna Shah

8 Jun 2026

Knowledge, attitude and practices towards rabies: A community survey in selected areas of KwaZulu-Natal Province, South Africa

PONE-D-23-27745R8

Dear Dr. Oguttu,

We’re pleased to inform you that your manuscript has been judged scientifically suitable for publication and will be formally accepted for publication once it meets all outstanding technical requirements.

Within one week, you’ll receive an e-mail detailing the required amendments. When these have been addressed, you’ll receive a formal acceptance letter and your manuscript will be scheduled for publication.

An invoice will be generated when your article is formally accepted. Please note, if your institution has a publishing partnership with PLOS and your article meets the relevant criteria, all or part of your publication costs will be covered. Please make sure your user information is up-to-date by logging into Editorial Manager at Editorial Manager® and clicking the ‘Update My Information' link at the top of the page. For questions related to billing, please contact billing support.

If your institution or institutions have a press office, please notify them about your upcoming paper to help maximize its impact. If they’ll be preparing press materials, please inform our press team as soon as possible -- no later than 48 hours after receiving the formal acceptance. Your manuscript will remain under strict press embargo until 2 pm Eastern Time on the date of publication. For more information, please contact onepress@plos.org.

Kind regards,

Venu Ramkrishna Shah

Academic Editor

PLOS One

Additional Editor Comments (optional):

Reviewers' comments:

# Editor report: Acceptance letter

**DOI:** 10.1371/journal.pone.0352279.r018

**Authors:** Venu Ramkrishna Shah

PONE-D-23-27745R8

PLOS One

Dear Dr. Oguttu,

I'm pleased to inform you that your manuscript has been deemed suitable for publication in PLOS One. Congratulations! Your manuscript is now being handed over to our production team.

At this stage, our production department will prepare your paper for publication. This includes ensuring the following:

* All references, tables, and figures are properly cited

* All relevant supporting information is included in the manuscript submission,

* There are no issues that prevent the paper from being properly typeset

You will receive further instructions from the production team, including instructions on how to review your proof when it is ready. Please keep in mind that we are working through a large volume of accepted articles, so please give us a few days to review your paper and let you know the next and final steps.

Lastly, if your institution or institutions have a press office, please let them know about your upcoming paper now to help maximize its impact. If they'll be preparing press materials, please inform our press team within the next 48 hours. Your manuscript will remain under strict press embargo until 2 pm Eastern Time on the date of publication. For more information, please contact onepress@plos.org.

You will receive an invoice from PLOS for your publication fee after your manuscript has reached the completed accept phase. If you receive an email requesting payment before acceptance or for any other service, this may be a phishing scheme. Learn how to identify phishing emails and protect your accounts at https://explore.plos.org/phishing.

If we can help with anything else, please email us at customercare@plos.org.

Thank you for submitting your work to PLOS ONE and supporting open access.

Kind regards,

PLOS ONE Editorial Office Staff

on behalf of

Dr. Venu Ramkrishna Shah

Academic Editor

PLOS One
